# Access to SCF_3_‑Substituted Indolizines via a Photocatalytic Late-Stage Functionalization Protocol

**DOI:** 10.1021/acs.orglett.5c02079

**Published:** 2025-07-21

**Authors:** Kevin Klaus Stefanoni, René Wilhelm

**Affiliations:** Institute of Organic Chemistry, 26534 Clausthal University of Technology, Leibnizstr. 6, 38678 Clausthal-Zellerfeld, Germany

## Abstract

A mild, scalable, and highly chemoselective photocatalytic method was developed for direct indolizine functionalization with *N*-((trifluoromethyl)­thio)­saccharin, yielding high amounts of desired products while tolerating various functional groups. The photoredox catalyst employed offers a straightforward and inexpensive alternative to commercially available catalysts. Additionally, a 3-SCF_3_ analogue of a histamine H3 receptor antagonist was synthesized with excellent yield, demonstrating the strategy’s potential in developing biologically relevant molecules.

## Graphical abstract

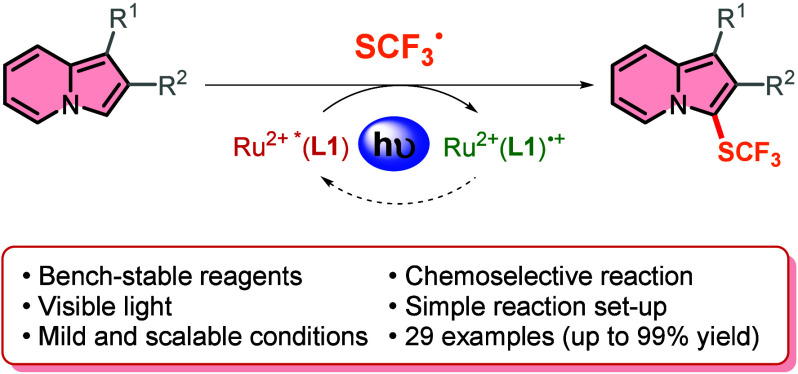

Indolizines are an important class of nitrogen-containing heterocycles that have a wide range of biologically relevant properties, being bioisosteres of indoles.^1–3^ Indolizine-containing molecules also find applications in dyes and fluorescent materials, with one of the most notable examples being Seoul-Fluor.^4^ Due to their relevance, numerous groups have been working on indolizine synthesis and also their late-stage functionalization over the last decades.^5–13^ The trifluoromethylthio (SCF_3_) group is an important pharmacophore found in numerous pharmaceutical and agrochemical products, including TAK-243 and Valiniprol (Figure 1).^14,15^

**Figure 1:**
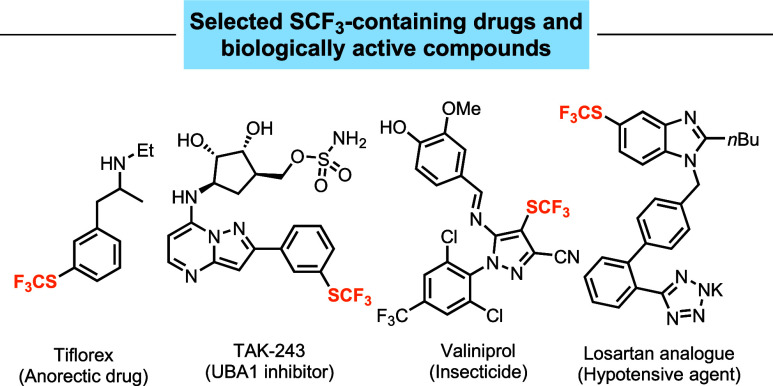
Drugs and bioactive molecules bearing a SCF_3_ group.

Incorporating a SCF_3_ group into pharmaceuticals is known to significantly enhance their metabolic stability and cell membrane permeability.^16–20^ Consequently, the direct functionalization of small molecules with the SCF_3_ group has been actively explored in synthetic chemistry. Early work in the field of electrophilic trifluoromethylthiolation employed gaseous trifluoromethylsulfenyl chloride (CF_3_SCl) as an electrophilic reagent.^21,22^ However, its significant toxicity and corrosive nature restrict its application, and thus, shelf-stable electrophilic CF_3_S reagents have been developed to overcome limitations associated with handling CF_3_SCl.

The first example of indolizine trifluoromethylthiolation was reported by Mirek et al. in 1981 (Scheme 1a).^23^ Despite the overall process yielding good results, indolizines were unselectively functionalized at both positions 1 and 3, irrespective of the presence of substituents on those positions (e.g., an acetyl group), upon reaction with gaseous trifluoromethylsulfenyl chloride (Cl-SCF_3_). Alongside the discovery of *N*-trifluoromethylthiosaccharin in 2014,^24,25^ Wang et al. proposed an FeCl_3_-catalyzed trifluoromethylthiolation of indoles and related heterocycles. Among those, two examples of indolizine trifluoromethylthiolation were also showcased, in which the desired 3-functionalized products were successfully prepared in excellent yields (Scheme 1b).^26^

**Scheme 1:**
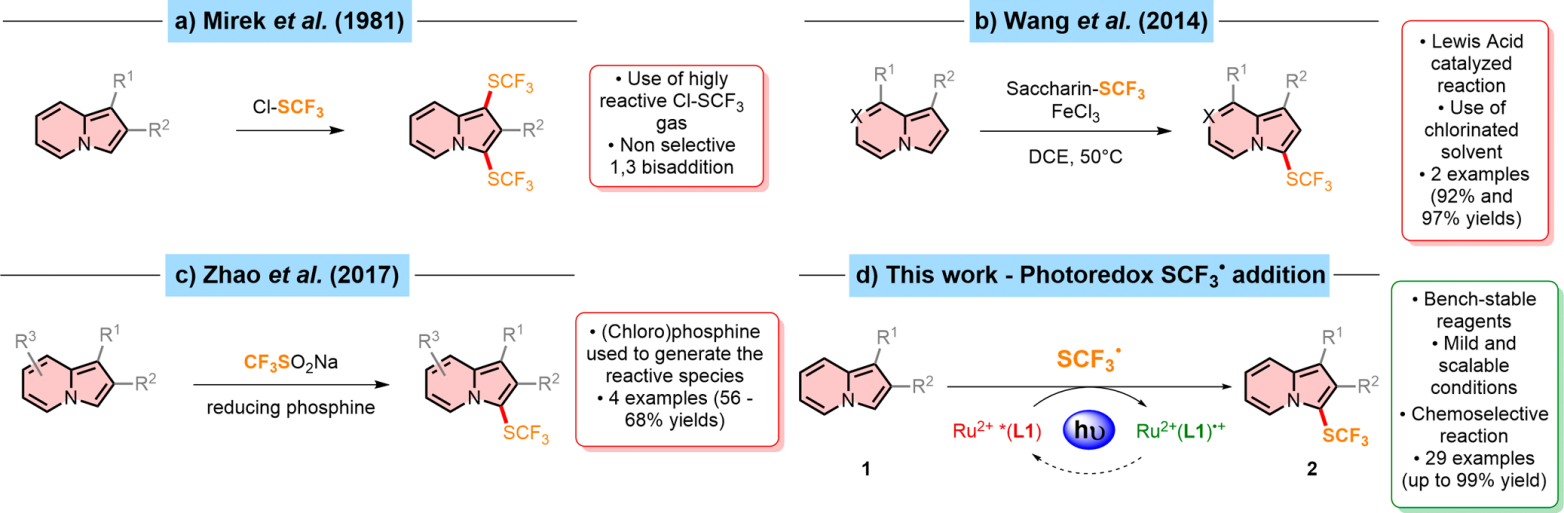
Previous and Current Protocols for the Synthesis of 3-((Trifluoromethyl)­thio)­indolizines

Immediately after, a transition-metal-free approach was reported by Honeker et al. for the *N*-heteroarene trifluoromethylthiolation employing *N*-trifluoromethylthiosaccharin.^27^ Despite the promising yields and the protocol operational simplicity, only one example of a 3-trifluoromethylthiolated indolizine was shown. With the development of photoredox catalysis, *N*-trifluoromethylthiosaccharin has increasingly been employed as an electrophilic radical source.^28,29^ More recently, alternative transition-metal-free electrophilic SCF_3_ sources were described by Chachignon et al. and Zhao et al.^30–32^ They reported that either CF_3_SO_2_Cl or CF_3_SO_2_Na reacted with a (chloro)­phosphine in order to generate the reactive species. These conditions, designed and optimized specifically for indole functionalization, proved to be challenging for indolizine substrates, as reflected in the lower yields (56–68%) (Scheme 1c).

Up to date, no synthetic procedure for the direct radical trifluoromethylthiolation of indolizines has been proposed, despite the continuous development of both radical sources and photoredox protocols. Herein, in our ongoing effort to create effective and straightforward photocatalysts and methods for the direct functionalization of heterocycles,^33,34^ we present a photocatalyzed radical trifluoromethylthiolation strategy to achieve a range of substituted 3-(trifluoromethylthio)­indolizine derivatives **2**, mediated by *N*-((trifluoromethyl)­thio)­saccharin as the SCF_3_ radical source in the benign solvent acetone and the new dyad-like complex [Ru­(dpp)_2_(dMeOTPA-Tz-bpy)]­(PF_6_)_2_ (**PC1**)^33^ as the photoredox catalyst with up to 99% yield (Scheme 1d).

For the optimization, 2-phenylindolizine-1-carbonitrile **1a** was chosen as the model substrate. Common photocatalysts were able to afford the desired trifluoromethylthiolated product in average to good yields (i.e., 35–70%) (entries 2–5, Table 1), with homoleptic [Ru­(dpp)_3_]­(PF_6_)_2_ as the best performing, despite leaving a considerable amount of unreacted indolizine **1a**. Conversely, our new reported photocatalyst **PC1** ^33^ was able to afford the desired product **2a** in an excellent yield of 92% while converting all of the starting material **1a** (entry 1, Table 1). As discussed previously,^33^ from an academic perspective, **PC1** is significantly more cost-effective than other commercially available photoredox catalysts, such as Ir­(ppy)_3_ and 4-CzIPN, reaffirming its superior value. The reaction without any photocatalyst afforded indolizine **2a** in a 16% yield (entry 6, Table 1). For further optimization studies toward the influence of solvent and wavelength of the applied light, see page S3 of the Supporting Information.

**Table 1:**
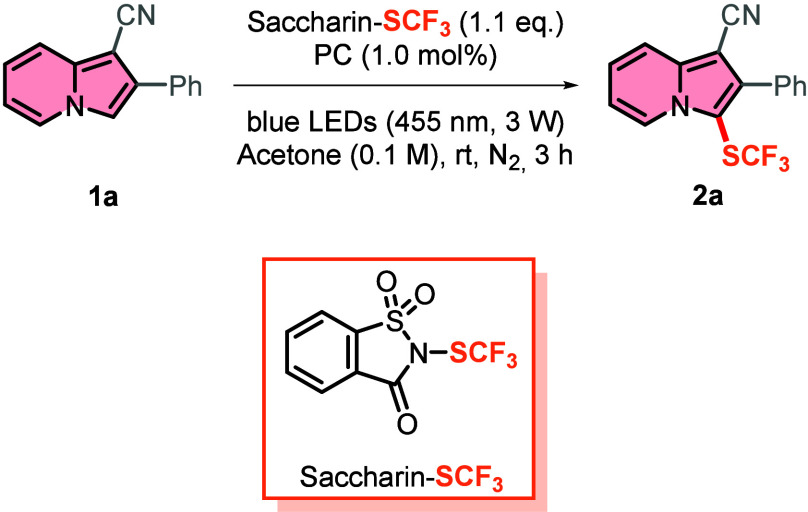
Optimization Studies for the Synthesis of Indolizine **2a** ^a^

entry	photoredox catalyst	yield (%)
1	PC1	96 (92)
2	[Ru(bpy)_3_](PF_6_)_2_	50
3	[Ru(dpp)_3_](PF_6_)_2_	70
4	Ir(ppy)_3_	59
5	4-CzIPN	35
6	—	16

a Reactions were carried out under a N_2_ atmosphere at 25 °C using compound **1a** (0.2 mmol), SCF_3_^•^ source (1.1 equiv), PC (1.0 mol %), and acetone (0.1 M of compound **1a**), irradiated by blue LEDs (455 nm, 3 W) for 3 h. Isolated yields after column chromatography are given in parentheses.

With the optimized conditions in hand, the scope and limitations of the protocol were investigated. A variety of different indolizine scaffolds were examined under the aforementioned conditions. Compared to indolizine **1a**, similar indolizine-1-carbonitriles afforded generally low yields. 2-(4-Methoxyphenyl)­indolizine-1-carbonitrile **1b** afforded the desired trifluoromethylthiolated product with 26% yield. 2-(2-Methoxyphenyl)­indolizine-1-carbonitrile **1c** resulted in a yield of 22%, which could be improved up to 51% if the reaction time was increased to 16 h.

This suggests that electron-donating groups have a positive effect on the yield, while steric factors control the kinetics of the CF_3_S radical addition to the indolizine core. However, no change in yields was noticed for 2-(4-methoxyphenyl)­indolizine-1-carbonitrile **1b** when the latter strategy was applied. Upon changing substituents on the aromatic ring at position 2 of the indolizine core to more electron-withdrawing groups, the yields dropped drastically. Indolizine **2d** was prepared from 2-(4-hydroxyphenyl)­indolizine-1-carbonitrile **1d** in a 30% yield, proving the chemoselectivity of the designed protocol even when free OH groups are present in indolizine. Among the tested indolizine-1-carbonitriles, 2-(thiophen-2-yl)­indolizine-1-carbonitrile **1w** and 2-methylindolizine-1-carbonitrile **1y** showed the best activity, affording indolizines **2w** and **2y** with yields of 77 and 84%, respectively. This result suggests how the designed trifluoromethylthiolation reaction is affected by the electronic nature of the starting materials **1**: strong electron-donating groups on position 2 are crucial to balance the pull effect of the −CN group on position 1 and to ultimately afford better yields. When the −CN group was exchanged to a carboxylic acid ester (i.e., −CO_2_Me or −CO_2_Et), an increase in yields was observed. Both methyl 2-(4-methoxyphenyl)-3-((trifluoromethyl)­thio)­indolizine-1-carboxylate **2o** and ethyl 2-(4-methoxyphenyl)-3-((trifluoromethyl)­thio)­indolizine-1-carboxylate **2q** were isolated with good yields of 61 and 55%, respectively, which are more than twice the yield of 2-(4-methoxyphenyl)­indolizine-1-carbonitrile **1b**. In analogy to the result obtained with indolizine-1-carbonitriles, these results further point out the critical role of electron-donating groups at position 2 in achieving optimal reaction outcomes. 2-Phenylindolizine analogues behaved differently compared to indolizine **1a**: while ethyl 2-phenyl-3-((trifluoromethyl)­thio)­indolizine-1-carboxylate **2p** was still isolated with a 73% yield, an average diminished yield of 53% was observed for methyl 2-phenyl-3-((trifluoromethyl)­thio)­indolizine-1-carboxylate **2n**. Excellent yields were instead achieved with methyl 2-(thiophen-2-yl)­indolizine-1-carboxylate **1v** and ethyl 2-(thiophen-2-yl)­indolizine-1-carboxylate **1t**, which were converted to the desired products **2v** and **2t** in yields of 78 and 94%, respectively. Ethyl 2-methylindolizine-1-carboxylate **1x** stood out as the best example of the reaction scope, as indolizine **2x** was isolated in a quantitative yield after the reaction. These last results finally show how carboxylic acid ester groups, particularly −CO_2_Et, have a less pronounced electron-withdrawing effect on the indolizine core than the −CN group, which, in turn, allows one to achieve excellent yields for products **2**. Removal of a substituent at position 1 resulted in general good to excellent yields. Simple 2-phenylindolizine **1e** was converted into the desire product **2e** with 75% yield. With the addition of a methoxy group on the phenyl ring, an increase in the yield was observed if the substituent is on the 4 position as for 2-(4-methoxyphenyl)­indolizine **1f** (i.e., 85%), while the desired 2-(2-methoxyphenyl)-3-((trifluoromethyl)­thio)­indolizine **2g** was obtained in a slightly diminished yield of 72%. Electron-donating substituents on the phenyl ring, like a methyl for 2-(4-methylphenyl)­indolizine **1h** and a bromine for 2-(4-bromophenyl)­indolizine **1j**, have a positive effect on the synthesis of the desired products **2h** and **2j**, which were isolated with 80 and 75% yields, respectively. Substituents like chlorine or trifluoromethyl had a larger impact on the electronic density of the indolizine core, resulting in diminished yields for the preparation of 2-(4-chlorophenyl)-3-((trifluoromethyl)­thio)­indolizine **2k** and 2-(4-(trifluoromethyl)­phenyl)-3-((trifluoromethyl)­thio)­indolizine **2l** (i.e., 50 and 65%, respectively).

An −OH group was also tolerated during the preparation of 2-(4-hydroxyphenyl)-3-((trifluoromethyl)­thio)­indolizine **2i**, and the desired product was isolated with 30% yield. Product **2i** was recovered in a mixture with indolizine **2i′** (2:1 ratio), which is 1,3-bis trifluoromethylthiolated indolizine. 2-(Thiophen-2-yl)­indolizine **1u** was successfully converted to the desired trifluoromethylthiolated indolizine **2u** with an excellent yield of 86%. Conversely to the related indolizine (**2x**, **2y**, and **2z**) bearing a methyl at the 2 position, 2-methyl-3-((trifluoromethyl)­thio)­indolizine **2aa** was isolated with a 53% yield. This latter average yield may be caused by the intrinsic sensitivity of the parent compound 2-methyl-indolizine **1aa** to light, heat, and air, an attribute that is also observed in its functionalized analogues. At last, the presence of a methyl group on the 6 position of the indolizine core did not significantly affect the outcome of the reaction. 6-Methyl-2-(*p*-tolyl)-3-((trifluoromethyl)­thio)­indolizine **2m** was synthesized with 80% yield, which is comparable to that of indolizine **2m**. 1-Sulfonylindolizines were also tested under the optimized conditions. Indolizine bearing a phenyl ring on the 2 position yielded in poor yields the desired product **2ab** (i.e., 20%) when the sulfone substituent was −SO_2_Me. The 2-methyl analogue instead stood as the best example among 1-sulfonylindolizines, being able to afford 2-methyl-1-(phenylsulfonyl)-3-((trifluoromethyl)­thio)­indolizine **2z** with a 74% yield. If a larger substituent group was present at position 2, the yield of the desired product decreased markedly. This indicates that steric factors play a significant role in governing the formation of the desired indolizines **2**. When another electron-withdrawing group is placed on the indolizine core at position 2 (e.g., −CO_2_Et), the synthesis of desired indolizines **2** was more challenging.

In fact, diethyl 3-((trifluoromethyl)­thio)­indolizine-1,2-dicarboxylate **2ac** was isolated in 22% yield. This result points out how electron-poor indolizines, such as **1ac**, are less prone to react under the optimized reaction conditions. As an important core unit for the preparation of novel organic fluorophores,^35^ indolizino­[1,2-*c*]­quinolin-6­(5*H*)-one **1s** was also applied under the optimized conditions. Notably, the desired product 12-((trifluoromethyl)­thio)­indolizino­[1,2-*c*]­quinolin-6­(5*H*)-one **2s** directly precipitated from the reaction media, and it was isolated with 79% yield. 2-(4-Methoxyphenyl)-1-phenylindolizine **1r** afforded the desired indolizine **2r** with a 47% yield.

In order to increase the yield for selected indolizines, the strategy of diminishing the volume of solvent used for the reaction by 4-fold, from 0.1 to 0.4 M, was adopted. By this means, the yield of 2-(4-methoxyphenyl)-3-((trifluoromethyl)­thio)­indolizine-1-carbonitrile **2b** increased from 26 to 52%, the yield of 1-(methylsulfonyl)-2-phenyl-3-((trifluoromethyl)­thio)­indolizine **2ab** increased from 20 to 53%, and the yield of diethyl 3-((trifluoromethyl)­thio)­indolizine-1,2-dicarboxylate **2ac** increased from 22 to 34%.

Additionally, a Hammett study was performed to investigate the effects of substituents on the indolizine core. The analysis yielded a ρ value of less than zero, indicating that the reaction involves the development of a positive charge in the transition state. For detailed information, see Table S1 and Figure S3 on pages S7 and S8 of the Supporting Information.

To demonstrate the scalability and effectiveness of the designed protocol, a scale-up experiment was performed using 1.8 mmol of indolizine **1a**, a 9-fold increase compared to previous conditions. Slight adjustments to the reaction parameters were made from the conditions outlined in Table 2: the solvent volume was reduced to a concentration of 0.2 M for indolizine **1a**, the loading of photocatalyst **PC1** was decreased to 0.5 mol %, and 1.08 equiv of saccharin–SCF_3_ was added. Under these modified conditions, the desired product **2a** was isolated with an excellent yield of 90%, closely comparable to the 92% yield obtained in the smaller batch experiment (footnote d, Table 2).

**Table 2:**
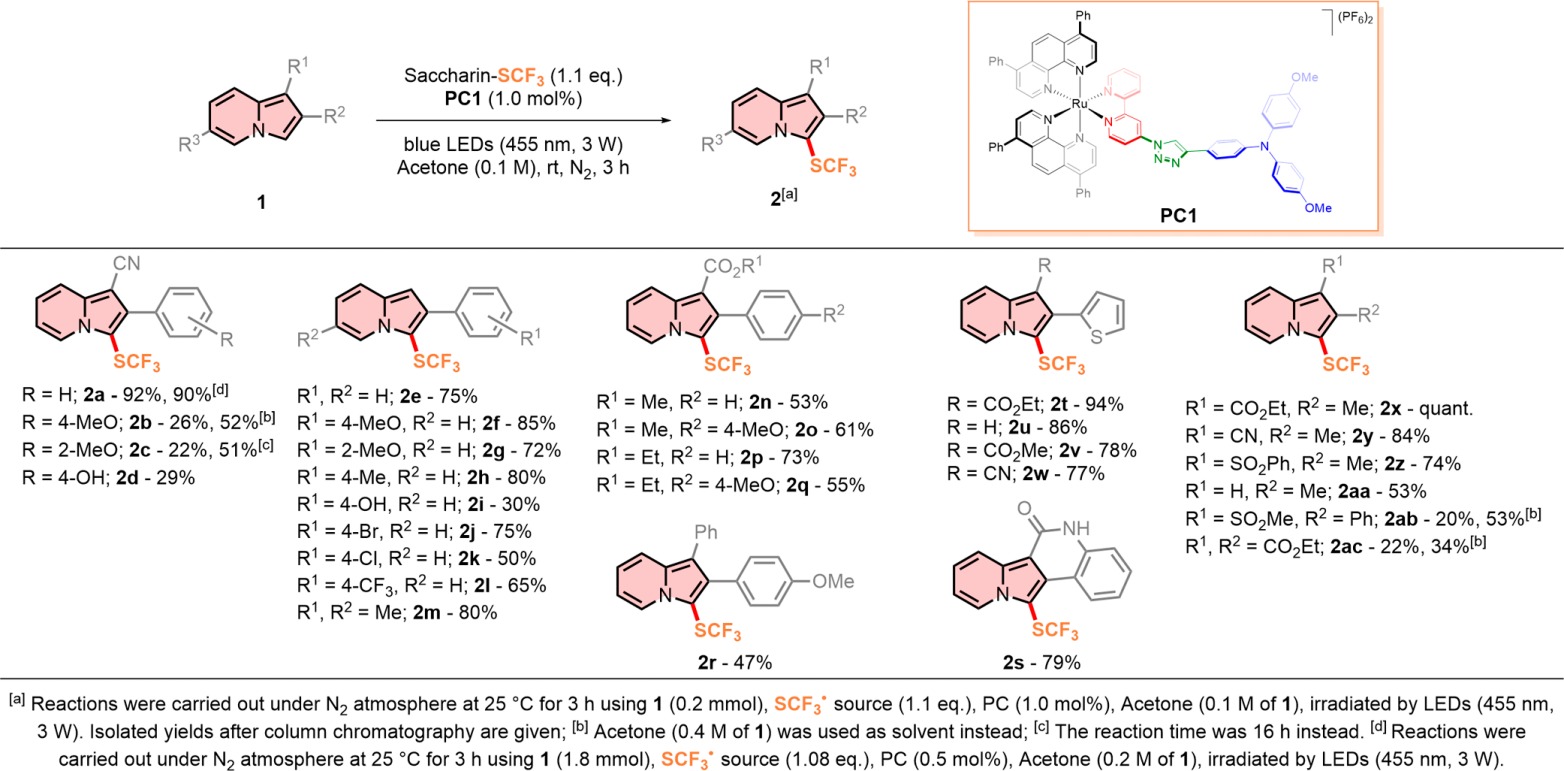
Photocatalytic Trifluoromethythiolation of Indolizines **1**

Given the significance of the indolizine scaffold in medicinal chemistry,^1,3^ a 3-SCF_3_ analogue of a biologically active indolizine has been synthesized. The straightforward alkylation of indolizine **2i** led to the isolation of product **3** in a quantitative yield (Scheme 2). Since trifluoromethylthiolated compounds often possess distinct and unique biological properties compared to their non-functionalized counterparts,^16,18^ we consider compound **3** a promising candidate for further medicinal applications. The non-trifluoromethylthiolated analogue of compound **3** has already been reported and evaluated as histamine H3 receptor antagonists.^36^

**Scheme 2:**
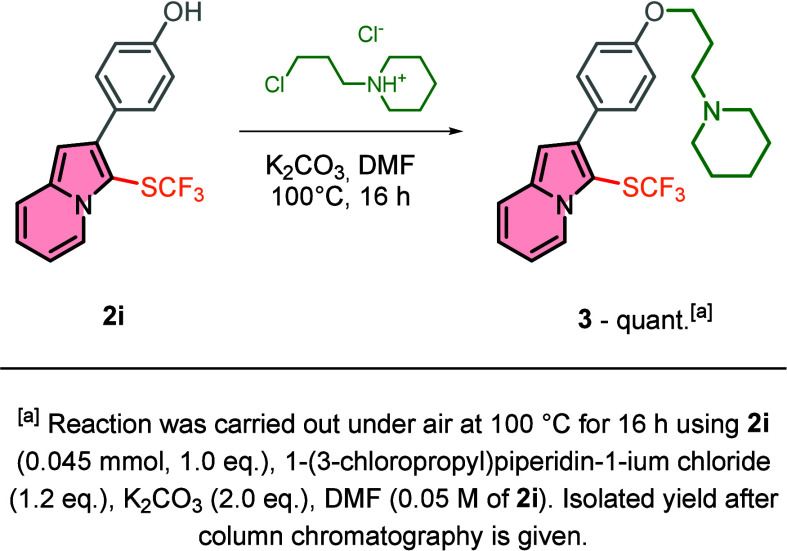
Synthesis of a Biologically Active Compound Analogue

A broad range of SCF_3_-subtituted indolizines were obtained chemoselectively via a photocatalytic late-state functionalization protocol. Simple, air-insensitive, and inexpensive reagents were employed. The newly reported catalyst **PC1** exceeds the performance of established commercially available photoredox catalysts, proving again its utility. The target 3-trifluoromethylthiolated indolizines were synthesized efficiently, achieving good to excellent yields. Furthermore, the protocol demonstrated excellent scalability, maintaining consistent yields upon scale-up without any significant loss in efficiency. To further highlight the significance of trifluoromethylthiolated compounds, a 3-SCF_3_ analogue of a histamine H3 receptor antagonist was prepared with an excellent yield.

## Supplementary Material





## Data Availability

The data underlying this study are available in the published article and its Supporting Information, and the FID NMR files are openly available in the ACS Chemistry Databank at 10.5061/dryad.v6wwpzh7x.

## Notes

Full experimental description, spectral data of the compounds, and additional spectroscopy data (PDF)
FAIR data, including the primary NMR FID files, for compounds **2** and **3** (ZIP)
