# Supplementary material for: Access to SCF3‑Substituted Indolizines via a Photocatalytic Late-Stage Functionalization Protocol
Source: Org Lett. 2025 Jul 21;27(31):8389–93. doi: 10.1021/acs.orglett.5c02079 (PMC12340967; doi:10.1021/acs.orglett.5c02079)
Supplement: Supplementary file 1 [file ol5c02079_si_001.pdf]

Supporting Information

Access to SCF_3 Substituted Indolizines via a Photocatalytic Late-Stage Functionalization Protocol

Kevin Klaus Stefanoni[a] and René Wilhelm[a]*

[a] Institute of Organic Chemistry, Leibnizstr. 6, Clausthal University of Technology, Germany
phone +495323722040, fax number: +495323722834, e-mail: rene.wilhelm@tu-clausthal.de

Table of contents

1. General experimental information S2

2. Photocatalytic reaction conditions S2

3. Starting materials preparation S3

4. Hammett-plot study S7

5. Reaction optimization S9

6. Trifluoromethylation of indolizines S10

6.1. General procedure for screening experiments S10

6.2. General procedure A for trifluoromethylthiolation of indolizines S10

6.3. Scale-up experiment for trifluoromethylthiolation of **1a** S25

6.4. Synthesis of analogues of biologically active compounds S26

7. Absorption, emission and electrochemical properties S27

8. Proposed reaction mechanism S29

9. NMR spectra S30

10. References S81

1. General experimental information

All reactions were carried out under an atmosphere of nitrogen in oven-dried glassware, unless otherwise stated. All reactions that require heating were conducted using a steel heat-on block. All chemicals were purchased and used without further purification unless otherwise mentioned. Anhydrous solvents were dried according to standard procedures before usage and stored in a glovebox. All NMR-Spectra have been measured using either BRUKER Digital AVANCE 400 MHz FTNMR or a BRUKER Digital AVANCE III 600 MHz FT-NMR. The chemical shifts are reported in ppm, the coupling constants in Hz. All mass spectra have been measured using a Hewlett-Packard Agilent LC/MSD-System Series HP 1100 with API-ES and the detector is TOF. All UV-vis spectra have been measured using a JASCO V-650 spectrophotometer or a JASCO V-760 spectrophotometer. The reactions were traced by thin layer chromatography with silica gel 60 (F254, MERCK KGAA). For the detection of substances, quenching was used at either 254 nm or 366 nm with a UV lamp. The preparative column chromatography was conducted through silica gel 60 (230–400 mesh).

All cyclic voltammetry experiments were performed in 0.1 M $[Bu_4N][PF_6]$ MeCN solution with an Autolab PGSTAT204 potentiostat/galvanostat (Metrohm). A cell with a three-electrode configuration was used. The glassy carbon working electrode (*d*=2 mm) was polished before each measurement with a 0.03 µm Al_2O_3 slurry and then rinsed thoroughly with deionized water and MeCN. A platinum sheet was used as counter electrode, while the reference electrode was Ag/AgCl (3.0 M KCl). The measurements were performed at a temperature of 22 °C under a nitrogen atmosphere after bubbling the solutions with the same gas for 10 min. A pre-bubbler is also included in the experimental set-up, to prevent excessive evaporation of the solvent. The IUPAC plotting convention was used to plot voltammograms. The initial potential was set to 0.0 V, and the scan proceeded in the oxidation direction up to the potential shown in the plot.

2. Photocatalytic reaction conditions

Reactions were performed in open round-bottom-flasks illuminated from below with Avonec 3 W High Power LEDs (https://www.avonec.de/3w-high-power-led/3w-high-power-led-10000k-20000k-kaltweiss-46-51-52-53.html) affixed to a cooling block and the setup was prevented from heating through a water-cooled metal case, as shown in **Figure S1** as an example. No filters were used during the irradiation process. The emission spectrum of the used blue LED is shown in **Figure S2** below.

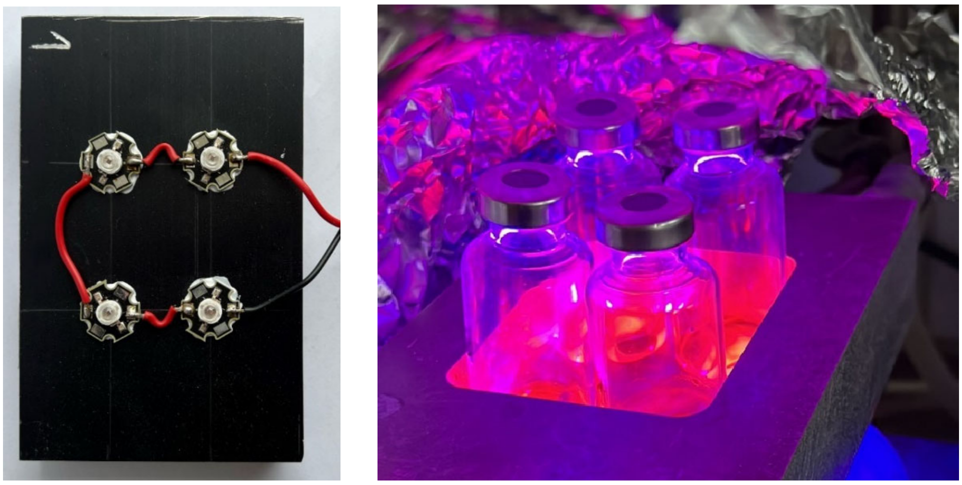

Figure S1. Custom LED photoreactor for reaction optimization and scale-up processes.

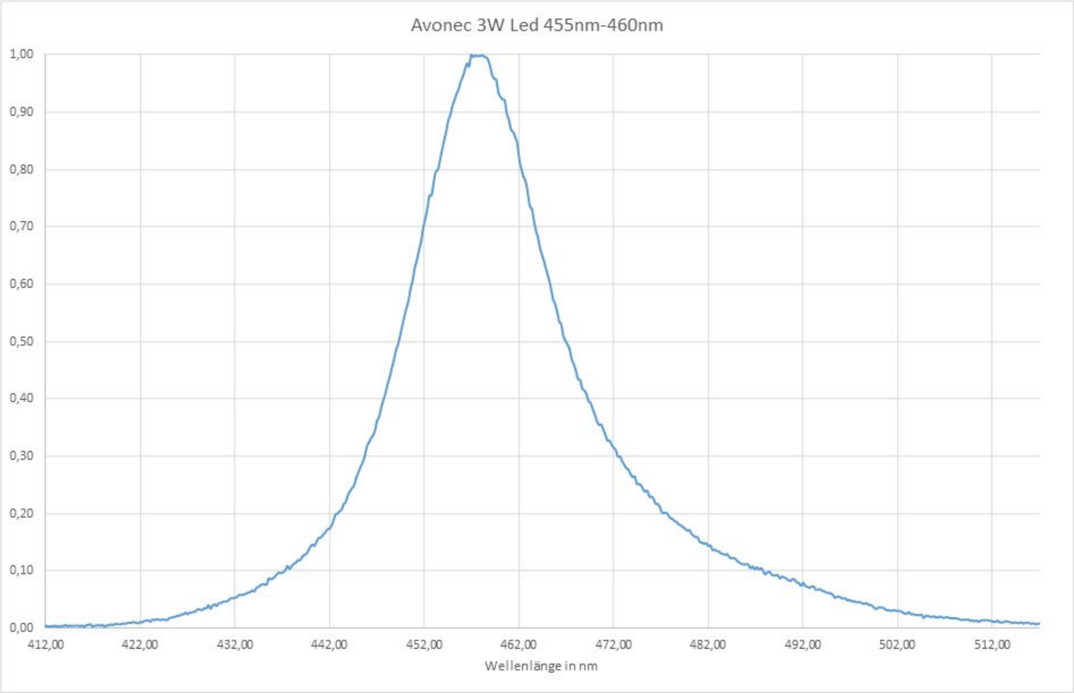

Figure S2. Emission spectrum of the used 455 nm LED lamp, as provided by the manufacturer.

3. Starting materials preparation

Indolizines **1a – 1d, 1n – 1q, 1t, 1v – 1w, 1ac** were prepared by boiling an equimolar MeCN or Acetone solution of the corresponding substituted picoline and α-bromoacetophenone with $NaHCO_3$ (2.0 eq.) for 16 h, according to a literature method.[1] Indolizines **1e – 1m, 1r, 1u** were prepared according to the literature.[2,3] Indolizines **1s** was prepared according to the literature.[4] Indolizines **1x – 1ab** were prepared according to the literature.[5] 4-azido-[2,2'-bipyridine] 1-oxide was prepared according to a published method.[6] Ru(II) precomplexes were prepared by heating an *o*DCB solution of ligand and $[Ru(COD)Cl_2]_n$

at 140°C for 3 h, according to literature.[7] Phthalimide-SCF_3[8] and Saccharin-SCF_3[9] were prepared according to a published method.

Reference 1

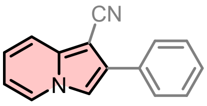

1a

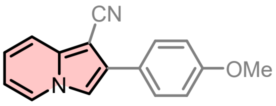

1b

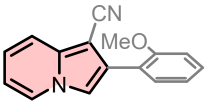

1c

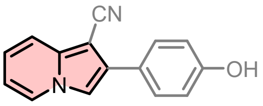

1d

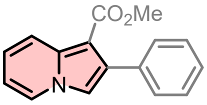

1n

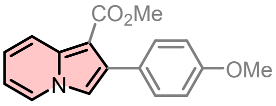

1o

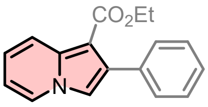

1p

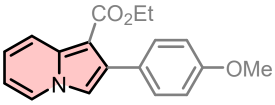

1q

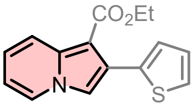

1t

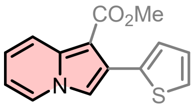

1v

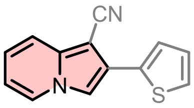

1w

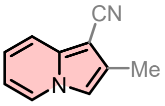

1y

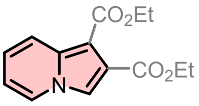

1ac

Reference 2,3

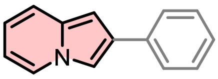

1e

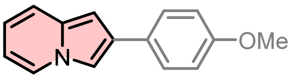

1f

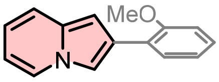

1g

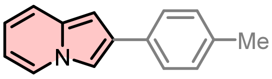

1h

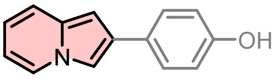

1i

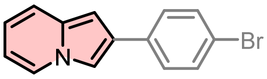

1j

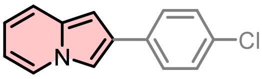

1k

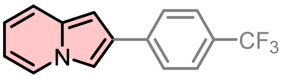

1l

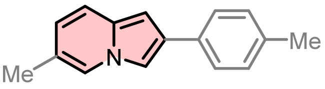

1m

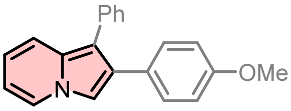

1r

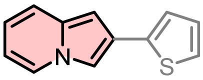

1u

Reference 4

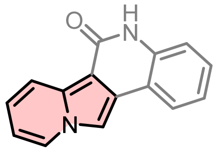

1s

Reference 5

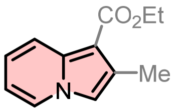

1x

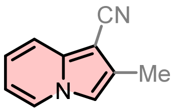

1y

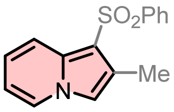

1z

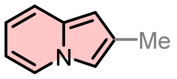

1aa

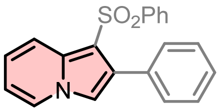

1ab

The synthesis of **PC1**, as previously reported, is described here below.[10] Reproduced from *J. Org. Chem.* **2025**, *90*, 6491-6503. Copyright 2025 American Chemical Society.

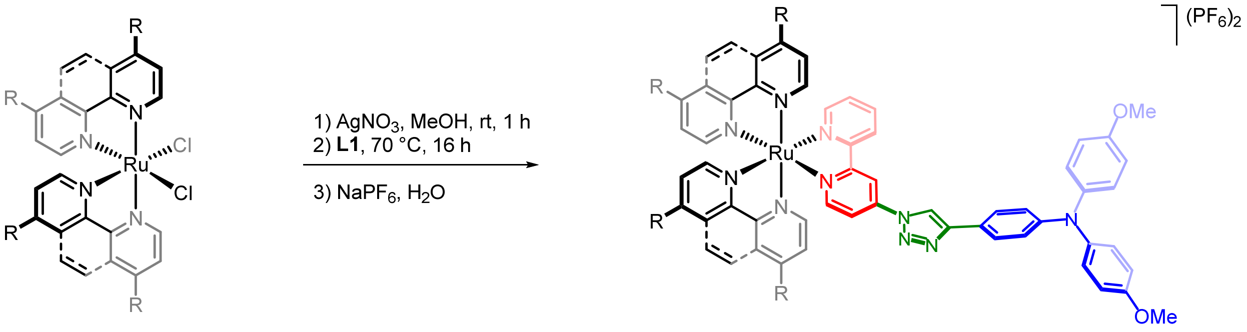

Ru(II) precomplex (86.0 µmol, 1.0 eq.) was mixed with $AgNO_3$ (29.3 mg, 173.0 µmol, 2.0 eq.) in MeOH (6 mL, 0.015 M) and the reaction mixture was stirred for 1 h at rt. The resulting suspension was filtered and then mixed with **L1** (50.0 mg, 95.0 µmol, 1.1 eq.). The mixture was heated at reflux for 16 h and later it was cooled down to rt. The solvent was evaporated under reduced pressure and the resulting solid was dissolved in the minimum amount of MeOH. The product is precipitated by addition of a $NaPF_6$ solution in distilled H_2O, washed sequentially with distilled H_2O/hexane/Et_2O and dried in vacuo.

[Ru(dpp)$_2$(dMeOTPA-Tz-bpy)](PF$_6$)$_2$ – PC1 Reproduced from *J. Org. Chem.* **2025**, *90*, 6491-6503. Copyright 2025 American Chemical Society.

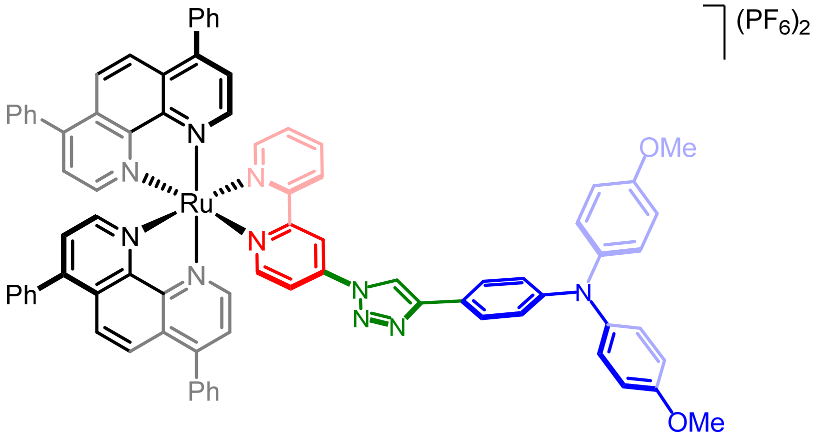

PC1

Complex **Ru-II** was obtained following the general procedure, using *cis*-dichlorobis(4,7-diphenyl-1,10-phenanthroline)ruthenium (72.2 mg, 86.0 µmol, 1.0 eq.), as a deep red solid: 0.104 g, 65.6 µmol, 76%. **^{1}H NMR** (600 MHz, CD_3CN) δ 9.02 (d, J = 2.3 Hz, 1H), 8.79 (d, J = 13.3 Hz, 2H), 8.43 (d, J = 5.5 Hz, 1H), 8.33 (d, J = 5.5 Hz, 1H), 8.29 (d, J = 5.5 Hz, 1H), 8.25 – 8.19 (m, 5H), 8.17 (d, J = 8.1 Hz, 2H), 8.13 (dd, J = 5.5, 2.5 Hz, 3H), 8.01 (d, J = 6.3 Hz, 2H), 7.93 (d, J = 5.2 Hz, 1H), 7.83 (dd, J = 6.3, 2.3 Hz, 1H), 7.78 (d, J = 3.6 Hz, 3H), 7.73 (d, J = 8.7 Hz, 3H), 7.65 – 7.61 (m, 18H), 7.46 – 7.41 (m, 2H), 7.09 (d, J = 9.0 Hz, 4H), 6.96 – 6.85 (m, 6H), 3.78 (s, 6H).**^{13}C{^{1}H} NMR** (150 MHz, CD_3CN) δ 140.7, 130.4, 130.4, 130.3, 129.7, 127.9, 127.1, 126.7, 115.5, 55.7. **^{19}F{^{1}H} NMR** (377 MHz, CD_3CN) δ -71.60 (d, 12F). **IR** (neat, cm^{-1}): 3061, 2931, 2909, 2835, 1615, 1502, 1504, 1239, 1031, 827, 702, 556. **ESI-HRMS**: m/z calcd. for $C_{80}H_{58}N_{10}O_2Ru$ $[M]^{2+}$ 646.1893, found 646.1894.

4. Hammett-plot study

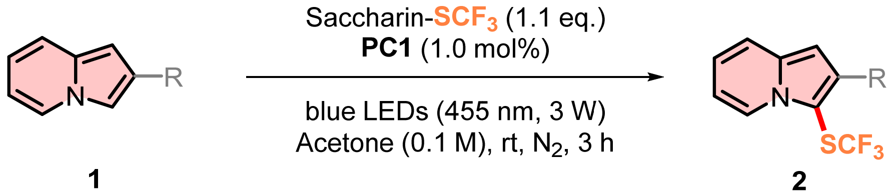

In a glovebox, to a vial filled with the indolizine **1** (0.20 mmol, 1.00 eq.), Saccharin-SCF_3 (62.3 mg, 0.22 mmol, 1.1 eq.), PC1 (3.2 mg, 2.0 µmol, 1.0 mol%) was added Acetone (2.0 mL, 0.1 M). The vial was sealed and the mixture was irradiated with a blue LED (3 W, 455 nm) for 3 h at rt outside of the glovebox. The reaction mixture was partitioned between H_2O and Et_2O and the organic phase was extracted. The water phase was extracted with Et_2O 3 times and the combined organic phases were washed with H_2O and brine. The organic phase was dried over Na_2SO_4 and the solvent was removed under reduced pressure. To the crude reaction mixture, CH_2Br_2 (34.7 mg, 14.0 µL, 0.2 mmol, 1.00 equiv.) was added as an internal standard. The reaction mixture was diluted with $CDCl_3$, and the yield was determined by ^1H NMR spectroscopy by integration of the peak at δ 4.95 ppm of the internal standard (s, 2H).

Table S1. Hammett-plot parameters for the photocatalytic trifluoromethylation of indolizines.

**Indolizine**	**Substituent**	**Ratio Yield X/H**	**log(k_X/k_H)**	**σ**[11]	**ρ**
**2e**	C_6H_5	1.00	0.000	-0.01	0
**2f**	C_6H_4-4-MeO	1.09	0.038	-0.08	-0.475
**2j**	C_6H_4-4-Br	0.99	-0.005	0.12	-0.042
**2k**	C_6H_4-4-Cl	0.92	-0.036	0.12	-0.300
**2h**	C_6H_4-4-Me	1.09	0.038	-0.03	-1.267
**2u**	2-Thienyl	1.06	0.024	0.05	0.480
**2l**	C_6H_4-4-CF_3	0.90	-0.047	0.25	-0.188

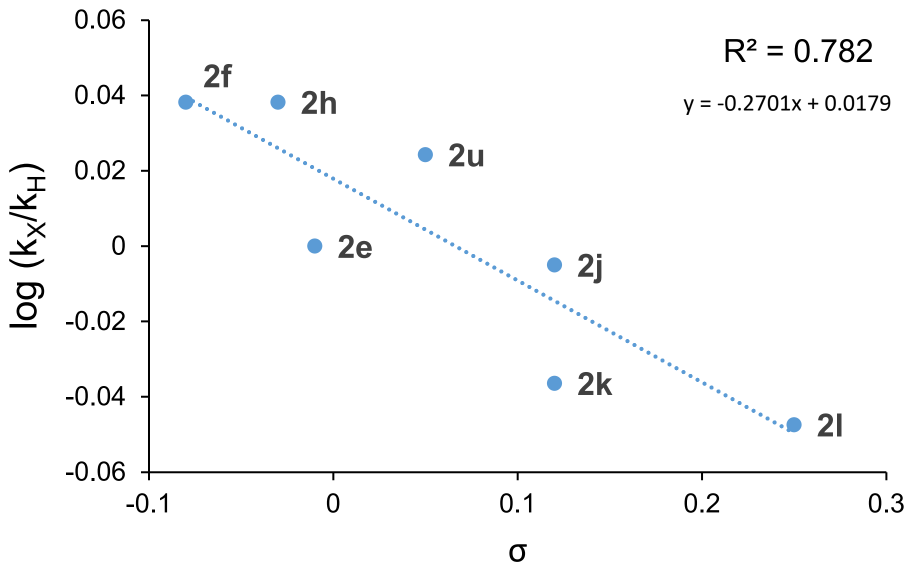

Figure S3. Hammett-plot for the photocatalytic trifluoromethylation of indolizines, electrophilic substituent constants according to the literature.[11]

Based on the results obtained, $\rho = -0.27$. As the latter value is $\rho < 0$, the reaction builds positive charge.

5. Reaction optimization

Table S2. Optimization studies for the synthesis of indolizine **2a**[a]

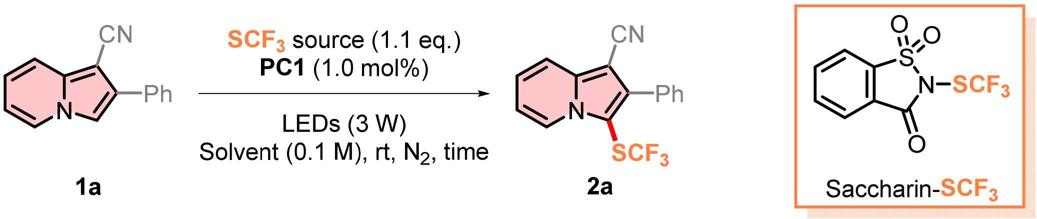

Entry	Photoredox catalyst	Time	LED	Yield
1	PC1	3 h	Blue (455 nm)	96% (92%)
2[b]	PC1	3 h	Blue (455 nm)	79%
3[c]	PC1	16 h	Blue (455 nm)	0%
4[d]	PC1	16 h	Blue (455 nm)	0%
5[e]	PC1	16 h	Blue (455 nm)	0%
6	PC1	3 h	Cyan (495 nm)	49%
7	PC1	16 h	Green (515 nm)	56%
8	PC1	1 h	Blue (455 nm)	39%
9	PC1	2 h	Blue (455 nm)	68%

[a] Reactions were carried out under N_2 atmosphere at 25 ° C using **1a** (0.2 mmol), $SCF_3^{\cdot}$ source (1.1 eq.), PC (1.0 mol%), Acetone (0.1 M of **1a**), irradiated by LEDs (3 W). Isolated yields after column chromatography are given in parentheses; [b] Using MeCN as solvent (0.1 M of **1a**); [c] Phthalimide-SCF_3 (1.1 eq.) was used instead; [d] In darkness; [e] Under air.

Acetone was selected as solvent to develop this protocol, as MeCN afforded a lower yield (79% vs 96%) when using our designed photocatalyst **PC1** (entry 1 - 2, Table S1). The use of a different $SCF_3^{\cdot}$ source (*e.g.*, Phthalimide-SCF_3) led to partial conversion of **1a** but no desired trifluoromethylthiolated product was detected (entry 3, Table S1). Both control reaction conducted in darkness (without light irradiation) and under air showed no product **2a** formation, with the latter experiment leading to the complete decomposition of indolizine **1a** (entry 4 - 5, Table S1). The choice of LEDs proved to be crucial and the best result was obtained with a common blue LED (*i.e.*, 455 nm, 3 W). Upon increase of the LEDs emission wavelength from cyano to green, yield of **2a** and conversion of **1a** dropped significantly (entries 6 - 7, Table S1). Optimum reaction time was chosen to be 3 h, as full conversion of **1a** could not be achieved with a shorter reaction time (entries 8 - 9, Table S1).

6. Trifluoromethylthiolation of indolizines

6.1. General procedure for screening experiments

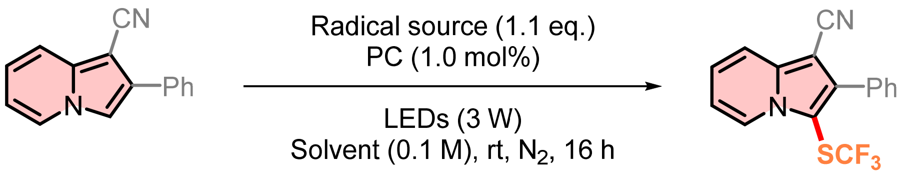

In a glovebox, to a vial filled with the indolizine **1a** (0.20 mmol, 1.00 eq.), SCF_3 radical source (0.22 mmol, 1.1 eq.), photoredox catalyst (2.0 µmol, 1.0 mol%) was added the selected solvent (2.0 mL, 0.1 M). The vial was sealed and the mixture was irradiated with a LED (3 W) for 16 h at rt outside of the glovebox. The reaction mixture was partitioned between H_2O and Et_2O and the organic phase was extracted. The water phase was extracted with Et_2O 3 times and the combined organic phases were washed with H_2O and brine. The organic phase was dried over Na_2SO_4 and the solvent was removed under reduced pressure. To the crude reaction mixture, CH_2Br_2 (34.7 mg, 14.0 µL, 0.2 mmol, 1.00 equiv.) was added as an internal standard. The reaction mixture was diluted with $CDCl_3$, and the yield was determined by ^1H NMR spectroscopy by integration of the peak at δ 4.95 ppm of the internal standard (s, 2H).

6.2. General procedure A for trifluoromethylthiolation of indolizines

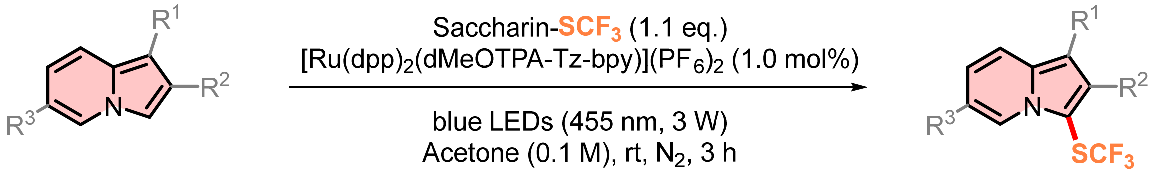

In a glovebox, to a vial filled with the indolizine **1** (0.20 mmol, 1.00 eq.), Saccharin-SCF_3 (62.3 mg, 0.22 mmol, 1.1 eq.), PC1 (3.2 mg, 2.0 µmol, 1.0 mol%) was added Acetone (2.0 mL, 0.1 M). The vial was sealed and the mixture was irradiated with a blue LED (3 W, 455 nm) for 3 h at rt outside of the glovebox. The reaction mixture was partitioned between H_2O and Et_2O and the organic phase was extracted. The water phase was extracted with Et_2O 3 times and the combined organic phases were washed with H_2O and brine. The organic phase was dried over Na_2SO_4 and the solvent was removed under reduced pressure. The residue was purified by flash column chromatography on silica gel to afford the trifluoromethylthiolated products **2**.

2-Phenyl-3-((trifluoromethyl)thio)indolizine-1-carbonitrile (2a)

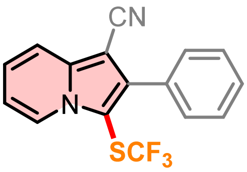

2a

Product **2a** was obtained following the general procedure **A** using 2-(phenyl)indolizine-1-carbonitrile **1a** (43.6 mg, 0.2 mmol, 1.00 eq.). The crude was purified by column chromatography on silica (PE:EtOAc, 4:1) to get the pure product as a brown wax: 58.5 mg, 0.18 mmol, 92%. **^{1}H NMR** (400 MHz, $CDCl_3$) δ 8.66 (dd, J = 7.2, 1.1 Hz, 1H), 7.78 (dt, J = 8.9, 1.2 Hz, 1H), 7.70 – 7.62 (m, 2H), 7.56 – 7.45 (m, 3H), 7.37 (ddd, J = 8.9, 6.8, 1.1 Hz, 1H), 7.05 (td, J = 6.9, 1.3 Hz, 1H). **^{13}C{^{1}H} NMR** (100 MHz, $CDCl_3$) δ 143.2, 141.0, 130.8, , 130.0, 129.1, 128.8, 128.5, 128.5 (q, J = 315 Hz), 126.1, 125.4, 118.0, 115.4, 114.7, 101.6, 85.1. **^{19}F{^{1}H} NMR** (377 MHz, $CDCl_3$): δ -43.49 (s, 3F). **IR** (neat, cm^{-1}): 3083, 3054, 2923, 2853, 2214, 1507, 1161, 1138, 1124, 1101, 1074, 1026, 747, 696, 652, 492, 468, 446. **ESI-HRMS**: m/z calcd. for $C_{16}H_9F_3N_2SNa$ $[M+Na]^+$ 341.0336, found 341.0331.

2-(4-Methoxyphenyl)-3-((trifluoromethyl)thio)indolizine-1-carbonitrile (2b)

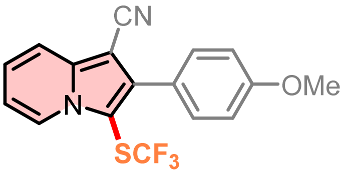

2b

Product **2b** was obtained following the general procedure **A** using 2-(4-methoxyphenyl)indolizine-1-carbonitrile **1b** (49.6 mg, 0.2 mmol, 1.00 eq.). The crude was purified by column chromatography on silica (PE:EtOAc, 9:1) to get the pure product as a yellowish wax: 18.1 mg, 0.05 mmol, 26%. **^{1}H NMR** (400 MHz, $CDCl_3$) δ 8.63 (d, J = 7.0 Hz, 1H), 7.75 (dt, J = 8.9, 1.2 Hz, 1H), 7.67 – 7.52 (m, 2H), 7.34 (ddd, J = 8.9, 6.8, 1.1 Hz, 1H), 7.14 – 6.86 (m, 3H), 3.88 (s, 3H). **^{13}C{^{1}H} NMR** (100 MHz, $CDCl_3$) δ 160.3, 142.9, 140.9, 130.0, 128.5 (q, J = 319 Hz), 125.9, 125.3, 123.0, 117.8, 115.6, 114.4, 114.2, 113.9, 101.1, 84.8, 55.4. **^{19}F{^{1}H} NMR** (377 MHz, $CDCl_3$): δ -43.51 (s, 3F). **IR** (neat, cm^{-1}): 3093, 2934, 2839, 2214, 1611, 1507, 1464, 1360, 1295, 1249, 1095, 1027, 837, 751, 735, 563, 505, 445. **ESI-HRMS**: m/z calcd. for $C_{17}H_{11}F_3N_2OSNa$ $[M+Na]^+$ 371.0442, found 371.0436.

2-(2-Methoxyphenyl)-3-((trifluoromethyl)thio)indolizine-1-carbonitrile (2c)

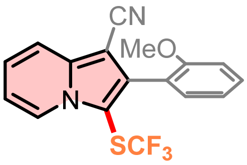

2c

Product **2c** was obtained following the general procedure **A** using 2-(2-methoxyphenyl)indolizine-1-carbonitrile **1c** (49.6 mg, 0.2 mmol, 1.00 eq.). The crude was purified by column chromatography on silica (PE:EtOAc, 9:1) to get the pure product as a yellowish wax: 35.5 mg, 0.10 mmol, 51%. **^{1}H NMR** (400 MHz, $CDCl_3$) δ 8.60 (d, J = 7.0 Hz, 1H), 7.75 (dd, J = 8.9, 1.3 Hz, 1H), 7.45 (ddd, J = 8.6, 7.6, 1.8 Hz, 1H), 7.40 – 7.28 (m, 2H), 7.16 – 6.90 (m, 3H), 3.84 (s, 3H). **^{13}C{^{1}H} NMR** (100 MHz, $CDCl_3$) δ 157.0, 140.5, 140.2, 131.9, 130.7, 128.5 (q, J = 315 Hz), 125.4, 125.2, 120.6, 119.7, 117.9, 115.3, 114.2, 111.3, 102.8, 86.3, 55.6. **^{19}F{^{1}H} NMR** (377 MHz, $CDCl_3$): δ -43.50 (s, 3F). **IR** (neat, cm^{-1}): 2922, 2851, 2209, 1603, 1509, 1476, 1360, 1245, 1149, 1099, 1016, 749, 460. **ESI-HRMS**: m/z calcd. for $C_{17}H_{11}F_3N_2OSNa$ $[M+Na]^+$ 371.0442, found 371.0436.

2-(4-Hydroxyphenyl)-3-((trifluoromethyl)thio)indolizine-1-carbonitrile (2d)

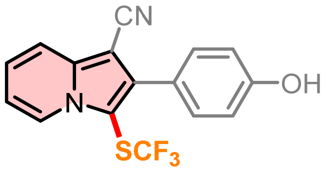

2d

Product **2d** was obtained following the general procedure **A** using 2-(4-hydroxyphenyl) indolizine-1-carbonitrile **1d** (46.8 mg, 0.2 mmol, 1.00 eq.). The crude was purified by column chromatography on silica (PE:EtOAc, 3:1) to get the pure product as a white wax: 19.8 mg, 0.06 mmol, 29%. **^{1}H NMR** (400 MHz, Acetone-d^{6}) δ 8.72 (d, J = 7.0 Hz, 1H), 8.68 (s, 1H), 7.67 (dt, J = 8.9, 1.2 Hz, 1H), 7.46 – 7.36 (m, 3H), 7.12 (td, J = 6.9, 1.3 Hz, 1H), 6.94 – 6.87 (m, 2H). **^{13}C{^{1}H} NMR** (100 MHz, Acetone-d^{6}) δ 158.4, 158.2, 142.8, 140.6, 131.2, 128.8 (q, J = 313 Hz), 126.7, 125.8, 122.0, 117.2, 115.5, 115.4, 114.9, 114.7, 84.4. **^{19}F{^{1}H} NMR** (377 MHz, Acetone-d^{6}): δ -43.64 (s, 3F). **IR** (neat, cm^{-1}): 3326, 2922, 2214, 1611, 1507, 1467, 1360, 1270, 1226, 1177, 1100, 837, 750, 734, 636, 507, 449. **ESI-HRMS**: m/z calcd. for $C_{16}H_9F_3N_2OSNa$ $[M+Na]^+$ 357.0285, found 357.0280.

2-Phenyl-3-((trifluoromethyl)thio)indolizine (2e)

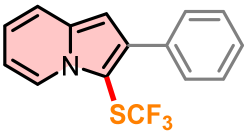

2e

Product **2e** was obtained following the general procedure **A** using 2-(phenyl)indolizine **1e** (38.6 mg, 0.2 mmol, 1.00 eq.). The crude was purified by column chromatography on silica (PE, 100%) to get the pure product as a white wax: 44.1 mg, 0.15 mmol, 75%. **^{1}H NMR** (400 MHz, $CDCl_3$) δ 8.57 (d, J = 7.1 Hz, 1H), 7.86 – 7.63 (m, 2H), 7.62 – 7.33 (m, 4H), 6.99 (dd, J = 8.9, 6.5 Hz, 1H), 6.79 - 6.76 (m, 2H). **^{13}C{^{1}H} NMR** (100 MHz, $CDCl_3$) δ 139.9, 137.4, 134.7, 129.4, 129.0 (q, J = 317 Hz), 128.5, 127.7, 124.3, 121.4, 119.0, 112.0, 101.8, 97.5. **^{19}F{^{1}H} NMR** (377 MHz, $CDCl_3$): δ -44.32 (s, 3F). **IR** (neat, cm^{-1}): 3069, 2924, 2854, 1505, 1451, 1351, 1241, 1098, 789, 759, 735, 691, 569, 503, 457, 427. **ESI-HRMS**: m/z calcd. for $C_{30}H_{19}F_6N_2S_2$ $[2M+H]^+$ 585.0894, found 585.0891.

2-(4-Methoxyphenyl)-3-((trifluoromethyl)thio)indolizine (2f)

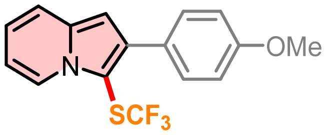

2f

Product **2f** was obtained following the general procedure **A** using 2-(4-methoxyphenyl)indolizine **1f** (44.6 mg, 0.2 mmol, 1.00 eq.). The crude was purified by column chromatography on silica (PE:EtOAc, 99:1) to get the pure product as a white wax: 54.8 mg, 0.17 mmol, 85%. **^{1}H NMR** (400 MHz, $CDCl_3$) δ 1H NMR (400 MHz, CDCl3) δ 8.50 (d, J = 7.1 Hz, 1H), 7.72 – 7.52 (m, 2H), 7.40 (dt, J = 8.9, 1.2 Hz, 1H), 7.02 – 6.95 (m, 2H), 6.92 (ddd, J = 8.8, 6.6, 1.1 Hz, 1H), 6.70 (td, J = 6.8, 1.3 Hz, 1H), 6.67 (s, 1H), 3.84 (s, 3H). **^{13}C{^{1}H} NMR** (100 MHz, $CDCl_3$) δ 159.3, 139.6, 137.4, 133.7, 130.6, 129.0 (q, J = 317 Hz), 127.1, 124.3, 121.3, 118.8, 114.0, 111.8, 101.4, 97.1, 55.4. **^{19}F{^{1}H} NMR** (377 MHz, $CDCl_3$): δ -44.37 (s, 3F). **IR** (neat, cm^{-1}): 2967, 2925, 2841, 1610, 1508, 1463, 1369, 1329, 1289, 1251, 1180, 1104, 1031, 829, 779, 728, 557, 463, 444. **ESI-HRMS**: m/z calcd. for $C_{16}H_{12}F_3NOSNa$ $[M+Na]^+$ 346.0489, found 346.0484.

2-(2-Methoxyphenyl)-3-((trifluoromethyl)thio)indolizine (2g)

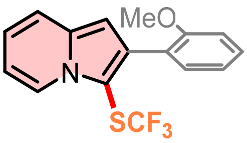

2g

Product **2g** was obtained following the general procedure **A** using methyl 2-(2-methoxyphenyl)indolizine **1g** (44.6 mg, 0.2 mmol, 1.00 eq.). The crude was purified by column chromatography on silica (PE:EtOAc, 98:2) to get the pure product as a white wax: 46.7 mg, 0.14 mmol, 72%. **^{1}H NMR** (400 MHz, $CDCl_3$) δ 8.54 (d, J = 7.1 Hz, 1H), 7.54 – 7.34 (m, 3H), 7.17 – 7.01 (m, 2H), 6.96 (ddd, J = 8.9, 6.6, 1.1 Hz, 1H), 6.83 – 6.66 (m, 2H), 3.83 (s, 3H). **^{13}C{^{1}H} NMR** (100 MHz, $CDCl_3$) δ 157.1, 137.1, 136.6, 133.7, 132.3, 129.3, 129.0 (q, J = 315 Hz), 124.3, 124.2, 123.8, 120.8, 120.5, 119.1, 111.7, 111.1, 103.1, 99.3, 55.6. **^{19}F{^{1}H} NMR** (377 MHz, $CDCl_3$): δ -44.28 (s, 3F). **IR** (neat, cm^{-1}): 3004, 2931, 2835, 1505, 1465, 1242, 1098, 1026, 777, 753, 735, 464, 428. **ESI-HRMS**: m/z calcd. for $C_{16}H_{12}F_3NOSNa$ $[M+Na]^+$ 346.0489, found 346.0483.

2-(*p*-Tolyl)-3-((trifluoromethyl)thio)indolizine (2h)

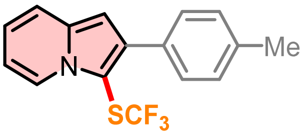

2h

Product **2h** was obtained following the general procedure **A** using 2-(*p*-tolyl)indolizine **1h** (41.4 mg, 0.2 mmol, 1.00 eq.). The crude was purified by column chromatography on silica (PE, 100%) to get the pure product as a white wax: 48.8 mg, 0.16 mmol, 80%. **^{1}H NMR** (400 MHz, $CDCl_3$) δ 8.56 (d, J = 7.1 Hz, 1H), 7.70 – 7.57 (m, 2H), 7.46 (dt, J = 8.9, 1.3 Hz, 1H), 7.31 (d, J = 7.8 Hz, 2H), 6.98 (ddd, J = 9.0, 6.6, 1.1 Hz, 1H), 6.82 – 6.70 (m, 2H), 2.46 (s, 3H). **^{13}C{^{1}H} NMR** (100 MHz, $CDCl_3$) δ 139.9, 137.5, 137.4, 133.7, 131.7, 129.3, 129.2, 129.0 (q, J = 316 Hz), 124.3, 121.3, 118.9, 111.8, 101.7, 97.3, 97.3, 21.4. **^{19}F{^{1}H} NMR** (377 MHz, $CDCl_3$): δ -44.32 (s, 3F). **IR** (neat, cm^{-1}): 2923, 1506, 1332, 1100, 825, 770, 736, 570, 506, 460. **ESI-HRMS**: m/z calcd. for $C_{32}H_{23}F_6N_2S_2$ $[2M+H]^+$ 613.1208, found 613.1202.

4-(3-((Trifluoromethyl)thio)indolizin-2-yl)phenol + 4-(1,3-bis((trifluoromethyl)thio)indolizin-2-yl)phenol (2i + 2i')

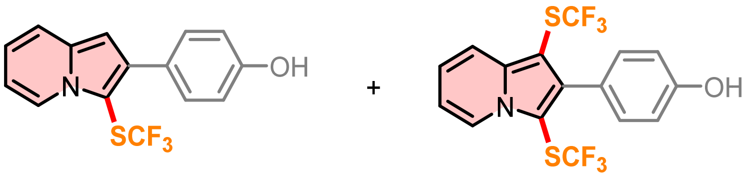

2i + 2i'

Product **2i** was obtained following the general procedure **A** using 4-(indolizin-2-yl)phenol **1i** (41.8 mg, 0.2 mmol, 1.00 eq.). The crude was purified by column chromatography on silica (PE:EtOAc, 4:1) to get the pure product as an off-white wax: 18.3 mg, 0.06 mmol, 30% (desired compound **2i**). **^{1}H NMR** (400 MHz, Acetone-d^6) δ 8.63 (d, J = 7.1 Hz, 1H), 8.55 (s, 1H), 7.67 – 7.50 (m, 3H), 7.07 (ddd, J = 8.9, 6.7, 1.1 Hz, 1H), 6.99 – 6.92 (m, 2H), 6.89 (td, J = 6.8, 1.2 Hz, 1H), 6.78 (d, J = 0.9 Hz, 1H). **^{13}C{^{1}H} NMR** (100 MHz, Acetone-d^6) δ 157.3, 139.8, 137.6, 130.3, 129.0 (q, J = 316 Hz), 125.6, 124.1, 121.6, 118.8, 115.3, 115.2, 112.0, 101.3. **^{19}F{^{1}H} NMR** (377 MHz, Acetone-d^6): δ -44.28 (s, 3F). **IR** (neat, cm^{-1}): 3297, 2923, 2853, 1611, 1507, 1450, 1331, 1242, 1094, 836, 778, 732, 569, 463. **ESI-HRMS**: m/z calcd. for $C_{15}H_{11}F_3NOS$ [M+H]$^+$ 310.0513, found 310.0508.

The bis-trifluoromethylthiolated by-product **2i'** was isolated as a colorless oil: 2.0 mg, 0.005 mmol, 2%. **^{1}H NMR** (400 MHz, $CDCl_3$) δ 8.63 (d, J = 7.0 Hz, 1H), 7.85 (d, J = 8.9 Hz, 1H), 7.36 – 7.28 (m, 2H), 7.04 – 6.89 (m, 3H), 4.92 (s, 1H). **^{13}C{^{1}H} NMR** (100 MHz, $CDCl_3$) δ 155.7, 132.4, 124.8, 117.9, 115.1, 113.9. **^{19}F{^{1}H} NMR** (377 MHz, $CDCl_3$): δ -43.99 (s, 3F), -44.84 (s, 3F). **ESI-HRMS**: m/z calcd. for $C_{16}H_{10}F_6NOS_2$ [M+H]$^+$ 410.0108, found 410.0111.

Mixed fractions of **2i + 2i'** (2:1) were also collected, yielding a yellowish oil: 29.3 mg, 45% combined. The mixture was analyzed as such.

2-(4-Bromophenyl)-3-((trifluoromethyl)thio)indolizine (2j)

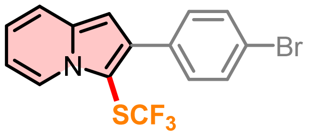

2j

Product **2j** was obtained following the general procedure **A** using 2-(4-bromophenyl)indolizine **1j** (54.4 mg, 0.2 mmol, 1.00 eq.). The crude was purified by column chromatography on silica (PE:EtOAc, 99:1) to get the pure product as a white wax: 55.7 mg, 0.15 mmol, 75%. **^{1}H NMR** (400 MHz, $CDCl_3$) δ 8.55 (d, J = 7.1 Hz, 1H), 7.69 – 7.51 (m, 4H), 7.46 (dt, J = 8.9, 1.2 Hz, 1H), 7.00 (ddd, J = 8.9, 6.6, 1.1 Hz,

1H), 6.78 (td, J = 6.9, 1.3 Hz, 1H), 6.72 (s, 1H). **^{13}C{^{1}H} NMR** (100 MHz, $CDCl_3$) δ 138.5, 137.4, 133.6, 133.6, 131.6, 130.9, 128.9 (q, J = 316 Hz), 124.3, 122.0, 121.6, 119.0, 112.2, 101.6, 97.4. **^{19}F{^{1}H} NMR** (377 MHz, $CDCl_3$): δ -44.34 (s, 3F). **IR** (neat, cm^{-1}): 2923, 1631, 1500, 1407, 1350, 1242, 1096, 1008, 827, 775, 733, 569, 503, 459. **ESI-HRMS**: m/z calcd. for $C_{15}H_9F_3NS$ [M-^{81}Br] 290.0428, found 290.0436.

2-(4-Chlorophenyl)-3-((trifluoromethyl)thio)indolizine (2k)

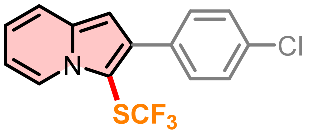

2k

Product **2k** was obtained following the general procedure **A** using 2-(4-chlorophenyl)indolizine **1k** (45.5 mg, 0.2 mmol, 1.00 eq.). The crude was purified by column chromatography on silica (PE:EtOAc, 99:1) to get the pure product as a white wax: 32.5 mg, 0.10 mmol, 50%. **^{1}H NMR** (400 MHz, $CDCl_3$) δ 8.55 (d, J = 7.1 Hz, 1H), 7.80 – 7.53 (m, 2H), 7.53 – 7.36 (m, 3H), 7.00 (ddd, J = 8.8, 6.6, 1.1 Hz, 1H), 6.78 (td, J = 6.8, 1.3 Hz, 1H), 6.72 (s, 1H). **^{13}C{^{1}H} NMR** (100 MHz, $CDCl_3$) δ 138.5, 137.4, 133.8, 133.1, 130.4, 128.9 (q, J = 316 Hz), 128.7, 124.3, 121.6, 119.0, 112.2, 101.7, 97.5. **^{19}F{^{1}H} NMR** (377 MHz, $CDCl_3$): δ -44.35 (s, 3F). **IR** (neat, cm^{-1}): 1503, 1463, 1409, 1350, 1182, 1128, 1094, 828, 777, 736, 723, 569, 461. **ESI-HRMS**: m/z calcd. for $C_{30}H_{17}F_6N_2S_2Cl_2$ $[2M+H]^+$ 653.0114, found 653.0105.

2-(4-(Trifluoromethyl)phenyl)-3-((trifluoromethyl)thio)indolizine (2l)

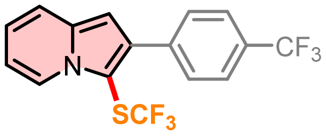

2l

Product **2l** was obtained following the general procedure **A** using 2-(4-(trifluoromethyl)phenyl)indolizine **1l** (52.2 mg, 0.2 mmol, 1.00 eq.). The crude was purified by column chromatography on silica (PE:EtOAc, 99:1) to get the pure product as a white wax: 47.2 mg, 0.13 mmol, 65%. **^{1}H NMR** (400 MHz, $CDCl_3$) δ 8.57 (d, J = 7.1 Hz, 1H), 7.83 (d, J = 8.2 Hz, 2H), 7.73 (d, J = 8.2 Hz, 2H), 7.49 (dt, J = 8.9, 1.3 Hz, 1H), 7.02 (ddd, J = 8.9, 6.6, 1.1 Hz, 1H), 6.81 (td, J = 6.9, 1.3 Hz, 1H), 6.77 (s, 1H). **^{13}C{^{1}H} NMR** (100 MHz, $CDCl_3$) δ 138.3, 138.2, 137.5, 133.5, 129.6, 129.6 (q, J = 32 Hz), 128.9 (q, J = 315 Hz), 128.5, 125.8, 125.4 (q, J = 4 Hz), 124.3, 124.1, 123.1, 121.8, 119.2, 112.5, 101.9, 97.8, 97.7. **^{19}F{^{1}H} NMR** (377 MHz, $CDCl_3$): δ -44.34 (s, 3F), -62.93 (s, 3F). **IR** (neat, cm^{-1}):

2924, 1618, 1500, 1416, 1320, 1097, 1065, 10177, 845, 777, 734, 689, 604, 445. **ESI-HRMS**: m/z calcd. for $C_{15}H_9F_3NSNa$ [M-CF_3] 292.0408, found 292.0399.

6-Methyl-2-(*p*-tolyl)-3-((trifluoromethyl)thio)indolizine (2m)

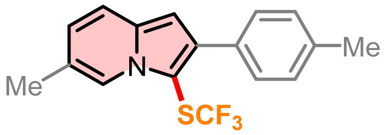

2m

Product **2m** was obtained following the general procedure **A** using 6-methyl-2-(*p*-tolyl)indolizine **1m** (44.2 mg, 0.2 mmol, 1.00 eq.). The crude was purified by column chromatography on silica (PE, 100%) to get the pure product as a yellow wax: 50.9 mg, 0.16 mmol, 80%. **^{1}H NMR** (400 MHz, $CDCl_3$) δ 1H NMR (400 MHz, CDC13) δ 8.36 (s, 1H), 7.76 – 7.55 (m, 2H), 7.37 (d, J = 9.0 Hz, 1H), 7.30 (d, J = 7.9 Hz, 2H), 6.85 (dd, J = 9.0, 1.5 Hz, 1H), 6.69 (s, 1H), 2.45 (s, 3H), 2.37 (s, 3H). **^{13}C{^{1}H} NMR** (100 MHz, $CDCl_3$) δ 139.3, 137.3, 136.2, 133.7, 131.9, 129.2, 129.2, 129.0 (q, J = 316 Hz), 124.6, 124.3, 122.0, 121.4, 118.4, 101.3, 96.7, 21.4, 18.7. **^{19}F{^{1}H} NMR** (377 MHz, $CDCl_3$): δ -44.40 (s, 3F). **IR** (neat, cm^{-1}): 2920, 2857, 1525, 1415, 1329, 1101, 824, 799, 578, 509, 462. **ESI-HRMS**: m/z calcd. for $C_{34}H_{27}F_6N_2S_2$ $[2M+H]^+$ 641.1520, found 641.1511.

Methyl 2-phenyl-3-((trifluoromethyl)thio)indolizine-1-carboxylate (2n)

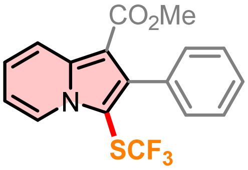

2n

Product **2n** was obtained following the general procedure **A** using methyl 2-(phenyl)indolizine-1-carboxylate **1n** (50.2 mg, 0.2 mmol, 1.00 eq.). The crude was purified by column chromatography on silica (PE:EtOAc, 9:1) to get the pure product as an off-white wax: 37.0 mg, 0.11 mmol, 53%. **^{1}H NMR** (400 MHz, $CDCl_3$) δ 8.65 (d, J = 7.0 Hz, 1H), 8.39 (dt, J = 9.0, 1.3 Hz, 1H), 7.50 – 7.41 (m, 3H), 7.39 – 7.31 (m, 3H), 7.01 (td, J = 6.9, 1.3 Hz, 1H), 3.71 (s, 3H). **^{13}C{^{1}H} NMR** (100 MHz, $CDCl_3$) δ 164.6, 143.6, 139.4, 133.7, 130.2, 128.5 (q, J = 315 Hz), 127.9, 127.0, 125.8, 124.8, 120.4, 114.1, 104.7, 103.2, 51.0. **^{19}F{^{1}H} NMR** (377 MHz, $CDCl_3$): δ -43.84 (s, 3F). **IR** (neat, cm^{-1}): 3085, 2952, 2923, 2853, 1693, 1496, 1438, 1411, 1327, 1231, 1173, 1140, 1101, 789, 768, 752, 702, 473, 451. **ESI-HRMS**: m/z calcd. for $C_{17}H_{12}F_3NO_2SNa$ $[M+Na]^+$ 374.0439, found 374.0433.

Methyl 2-(4-methoxyphenyl)-3-((trifluoromethyl)thio)indolizine-1-carboxylate (2o)

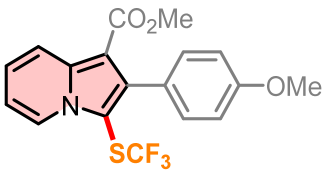

2o

Product **2o** was obtained following the general procedure **A** using methyl 2-(4-methoxyphenyl)indolizine-1-carboxylate **1o** (56.3 mg, 0.2 mmol, 1.00 eq.). The crude was purified by column chromatography on silica (PE:EtOAc, 9:1) to get the pure product as a white wax: 46.6 mg, 0.12 mmol, 61%. **^{1}H NMR** (400 MHz, $CDCl_3$) δ 8.64 (d, J = 7.0 Hz, 1H), 8.37 (dd, J = 8.9, 1.4 Hz, 1H), 7.39 – 7.22 (m, 3H), 7.06 – 6.89 (m, 3H), 3.89 (s, 3H), 3.74 (s, 3H). **^{13}C{^{1}H} NMR** (100 MHz, $CDCl_3$) δ 164.6, 159.3, 143.3, 139.4, 133.3, 131.6, 131.3, 128.6 (q, J = 315 Hz), 125.7, 125.7, 124.8, 123.9, 120.3, 114.0, 113.0, 104.6, 103.1, 103.1, 55.3, 51.0. **^{19}F{^{1}H} NMR** (377 MHz, $CDCl_3$): δ -43.84 (s, 3F). **IR** (neat, cm^{-1}): 3010, 2952, 2834, 1696, 1536, 1494, 1439, 1355, 1327, 1293, 1243, 1145, 1094, 1027, 836, 785, 752, 629, 565, 525, 451. **ESI-HRMS**: m/z calcd. for $C_{18}H_{14}F_3NO_3SNa$ $[M+Na]^+$ 404.0544, found 404.0539.

Ethyl 2-phenyl-3-((trifluoromethyl)thio)indolizine-1-carboxylate (2p)

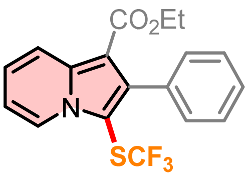

2p

Product **2p** was obtained following the general procedure **A** using ethyl 2-phenylindolizine-1-carboxylate **1p** (53.0 mg, 0.2 mmol, 1.00 eq.). The crude was purified by column chromatography on silica (PE:EtOAc, 9:1) to get the pure product as a colorless oil: 53.1 mg, 0.15 mmol, 73%. **^{1}H NMR** (400 MHz, $CDCl_3$) δ 8.64 (d, J = 7.0 Hz, 1H), 8.41 (dt, J = 9.0, 1.2 Hz, 1H), 7.44 (dd, J = 5.1, 2.1 Hz, 3H), 7.40 – 7.28 (m, 3H), 7.00 (td, J = 6.9, 1.3 Hz, 1H), 4.18 (q, J = 7.1 Hz, 2H), 1.09 (t, J = 7.1 Hz, 3H). **^{13}C{^{1}H} NMR** (100 MHz, $CDCl_3$) δ 164.2, 143.5, 139.4, 133.9, 133.3, 130.2, 128.6 (q, J = 314 Hz), 127.4, 127.0, 125.7, 124.8, 123.9, 120.2, 114.1, 105.0, 102.9, 59.7, 14.1. **^{19}F{^{1}H} NMR** (377 MHz, $CDCl_3$): δ -43.88 (s, 3F). **IR** (neat, cm^{-1}): 3065, 2981, 2933, 1683, 1502, 1424, 1365, 1284, 1094, 1049, 788, 744, 697, 637, 451. **ESI-HRMS**: m/z calcd. for $C_{18}H_{14}F_3NO_2SNa$ $[M+Na]^+$ 388.0595, found 388.0591.

Ethyl 2-(4-methoxyphenyl)-3-((trifluoromethyl)thio)indolizine-1-carboxylate (2q)

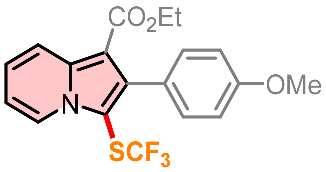

2q

Product **2q** was obtained following the general procedure **A** using ethyl 2-(4-methoxyphenyl)indolizine-1-carboxylate **1q** (59.0 mg, 0.2 mmol, 1.00 eq.). The crude was purified by column chromatography on silica (PE:EtOAc, 9:1) to get the pure product as a colorless oil: 43.5 mg, 0.11 mmol, 55%. **^{1}H NMR** (400 MHz, $CDCl_3$) δ 1H NMR (400 MHz, CDC13) δ 8.63 (d, J = 7.0 Hz, 1H), 8.38 (dt, J = 9.1, 1.2 Hz, 1H), 7.41 – 7.19 (m, 3H), 7.07 – 6.89 (m, 3H), 4.21 (q, J = 7.1 Hz, 2H), 3.88 (s, 3H), 1.16 (t, J = 7.1 Hz, 3H). **^{13}C{^{1}H} NMR** (100 MHz, $CDCl_3$) δ 164.2, 159.3, 143.3, 139.4, 131.6, 128.6 (q, J = 314 Hz), 126.0, 125.6, 124.8, 120.3, 114.0, 113.0, 105.0, 103.0, 103.0, 59.7, 55.3, 14.3. **^{19}F{^{1}H} NMR** (377 MHz, $CDCl_3$): δ -43.38 (s, 3F). **IR** (neat, cm^{-1}): 2985, 2926, 2854, 1677, 1632, 1504, 1427, 1363, 1282, 1244, 1185, 1156, 1102, 1057, 857, 826, 790, 749, 628, 564, 453. **ESI-HRMS**: m/z calcd. for $C_{19}H_{16}F_3NO_3SNa$ $[M+Na]^+$ 418.0701, found 418.0695.

2-(4-Methoxyphenyl)-1-phenyl-3-((trifluoromethyl)thio)indolizine (2r)

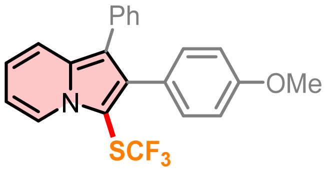

2r

Product **2r** was obtained following the general procedure **A** using 2-(4-methoxyphenyl)-1-phenylindolizine **1r** (59.8 mg, 0.2 mmol, 1.00 eq.). The crude was purified by column chromatography on silica (PE:EtOAc, 95:5) to get the pure product as a colorless oil: 37.7 mg, 0.09 mmol, 47%. **^{1}H NMR** (400 MHz, $CDCl_3$) δ 8.63 (d, J = 7.1 Hz, 1H), 7.67 (dt, J = 9.0, 1.2 Hz, 1H), 7.40 – 7.23 (m, 6H), 7.03 (ddd, J = 9.1, 6.6, 1.1 Hz, 1H), 6.97 – 6.89 (m, 2H), 6.83 (td, J = 6.8, 1.3 Hz, 1H), 3.87 (s, 3H). **^{13}C{^{1}H} NMR** (100 MHz, $CDCl_3$) δ 159.0, 138.2, 135.2, 134.2, 132.2, 130.5, 128.9 (q, J = 315 Hz), 128.5, 126.2, 125.9, 124.3, 121.8, 117.9, 115.8, 113.5, 112.4, 99.4, 99.3, 55.3. **^{19}F{^{1}H} NMR** (377 MHz, $CDCl_3$): δ -44.15 (s, 3F). **IR** (neat, cm^{-1}): 3043, 2936, 2835, 1612, 1530, 1462, 1351, 1244, 1095, 1031, 907, 834, 784, 760, 728, 699, 623, 563. **ESI-HRMS**: m/z calcd. for $C_{22}H_{16}F_3NOSNa$ $[M+Na]^+$ 422.0802, found 422.0797.

12-((Trifluoromethyl)thio)indolizino[1,2-c]quinolin-6(5*H*)-one (2s)

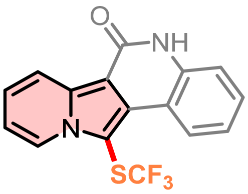

2s

Product **2s** was obtained following the general procedure **A** using indolizino[1,2-c]quinolin-6(5*H*)-one **1s** (46.8 mg, 0.2 mmol, 1.00 eq.) with a slight modification. The pure product was filtered from the reaction mixture, washed with Acetone, Et_2O and pentane and obtained as a brown solid: 46.9 mg, 0.15 mmol, 79%. **^{1}H NMR** (400 MHz, DMSO-d^6) δ 11.35 (s, 1H), 9.02 (d, J = 7.0 Hz, 1H), 8.90 (d, J = 8.1 Hz, 1H), 8.50 (d, J = 8.7 Hz, 1H), 7.58 (t, J = 7.8 Hz, 1H), 7.51 (t, J = 7.7 Hz, 1H), 7.43 (d, J = 8.2 Hz, 1H), 7.37 (t, J = 7.0 Hz, 1H), 7.28 (t, J = 7.6 Hz, 1H). **^{13}C{^{1}H} NMR** (100 MHz, DMSO-d^6) δ 159.2, 138.9, 136.1, 133.3, 130.1, 126.4, 125.1, 124.4, 122.1, 119.4, 116.9, 116.6, 114.5, 105.1, 93.4. **^{19}F{^{1}H} NMR** (377 MHz, DMSO-d^6): δ -44.41 (s, 3F). **IR** (neat, cm^{-1}): 3115, 2986, 2873, 1661, 1590, 1547, 1521, 1480, 1415, 1380, 1337, 1250, 1110, 848, 746, 708, 559, 461. **ESI-HRMS**: m/z calcd. for $C_{16}H_9F_3N_2OSNa$ [M+Na]$^+$ 357.0285, found 357.0280.

Ethyl 2-(thiophen-2-yl)-3-((trifluoromethyl)thio)indolizine-1-carboxylate (2t)

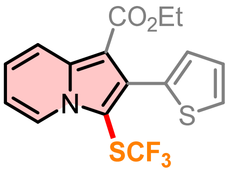

2t

Product **2t** was obtained following the general procedure **A** using ethyl 2-(thiophen-2-yl) indolizine-1-carboxylate **1t** (54.2 mg, 0.2 mmol, 1.00 eq.). The crude was purified by column chromatography on silica (PE, 100%) to get the pure product as a colorless oil: 70.1 mg, 0.19 mmol, 94%. **^{1}H NMR** (400 MHz, $CDCl_3$) δ 8.60 (d, J = 7.0 Hz, 1H), 8.36 (dq, J = 9.1, 0.9 Hz, 1H), 7.46 (ddd, J = 4.8, 1.6, 0.6 Hz, 1H), 7.34 – 7.27 (m, 1H), 7.16 – 7.09 (m, 2H), 7.01 – 6.92 (m, 1H), 4.23 (q, J = 7.1 Hz, 2H), 1.19 (t, J = 7.1 Hz, 3H). **^{13}C{^{1}H} NMR** (100 MHz, $CDCl_3$) δ 163.9, 139.4, 135.5, 133.4, 133.2, 129.3, 128.4 (q, J = 315 Hz), 126.8, 126.5, 125.7, 124.7, 123.8, 120.3, 114.3, 105.9, 104.1, 104.1, 59.9, 14.1. **^{19}F{^{1}H} NMR** (377 MHz, $CDCl_3$): δ -43.65 (s, 3F). **IR** (neat, cm^{-1}): 3107, 2981, 2904, 1685, 1503, 1431, 1366, 1282, 1238, 1096, 1035, 853, 784, 745, 695, 446. **ESI-HRMS**: m/z calcd. for $C_{16}H_{12}F_3NO_2S_2Na$ [M+Na]$^+$ 394.0159, found 394.0154.

2-(Thiophen-2-yl)-3-((trifluoromethyl)thio)indolizine (2u)

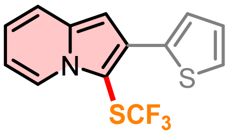

2u

Product **2u** was obtained following the general procedure **A** using 2-(thiophen-2-yl)indolizine **1u** (39.8 mg, 0.2 mmol, 1.00 eq.). The crude was purified by column chromatography on silica (PE, 100%) to get the pure product as a white wax: 51.5 mg, 0.17 mmol, 86%. **^{1}H NMR** (400 MHz, $CDCl_3$) δ 8.51 (d, J = 7.1 Hz, 1H), 7.61 (dd, J = 3.6, 1.1 Hz, 1H), 7.52 – 7.32 (m, 2H), 7.24 – 7.07 (m, 1H), 6.97 (ddd, J = 9.0, 6.6, 1.1 Hz, 1H), 6.81 (d, J = 1.0 Hz, 1H), 6.74 (td, J = 6.9, 1.3 Hz, 1H). **^{13}C{^{1}H} NMR** (100 MHz, $CDCl_3$) δ 137.5, 136.3, 133.6, 132.8, 128.9 (q, J = 317 Hz), 127.3, 126.1, 125.9, 124.2, 121.7, 118.8, 112.1, 100.7, 96.7. **^{19}F{^{1}H} NMR** (377 MHz, $CDCl_3$): δ -44.12 (s, 3F). **IR** (neat, cm^{-1}): 3074, 2923, 2853, 1502, 1318, 1091, 829, 776, 734, 696, 564, 457, 428. **ESI-HRMS**: m/z calcd. for $C_{26}H_{15}F_6N_2S_4$ $[2M+H]^+$ 597.0022, found 597.0023.

Methyl 2-(thiophen-2-yl)-3-((trifluoromethyl)thio)indolizine-1-carboxylate (2v)

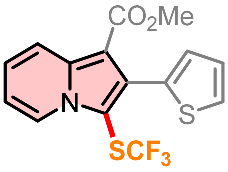

2v

Product **2v** was obtained following the general procedure **A** using methyl 2-(thiophen-2-yl)indolizine-1-carboxylate **1v** (51.5 mg, 0.2 mmol, 1.00 eq.). The crude was purified by column chromatography on silica (PE:EtOAc, 95:5) to get the pure product as a white wax: 55.5 mg, 0.16 mmol, 78%. **^{1}H NMR** (400 MHz, $CDCl_3$) δ 8.63 (d, J = 7.0 Hz, 1H), 8.36 (dt, J = 9.1, 1.3 Hz, 1H), 7.49 (dd, J = 5.0, 1.4 Hz, 1H), 7.33 (ddd, J = 9.2, 6.7, 1.1 Hz, 1H), 7.24 – 7.10 (m, 2H), 7.00 (td, J = 6.9, 1.3 Hz, 1H), 3.79 (s, 3H). **^{13}C{^{1}H} NMR** (100 MHz, $CDCl_3$) δ 164.2, 139.3, 135.5, 133.1, 133.1, 129.3, 128.4 (q, J = 317 Hz), 126.8, 126.5, 125.8, 124.7, 123.7, 120.2, 114.2, 105.4, 104.1, 104.1, 51.0. **^{19}F{^{1}H} NMR** (377 MHz, $CDCl_3$): δ -43.61 (s, 3F). **IR** (neat, cm^{-1}): 3087, 2923, 2853, 1695, 1496, 1439, 1319, 1215, 1093, 1006, 843, 782, 752, 685, 476, 446. **ESI-HRMS**: m/z calcd. for $C_{15}H_{10}F_3NO_2S_2Na$ $[M+Na]^+$ 380.0003, found 379.9997.

2-(Thiophen-2-yl)-3-((trifluoromethyl)thio)indolizine-1-carbonitrile (2w)

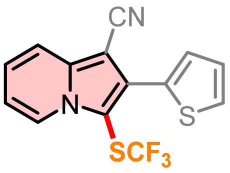

2w

Product **2w** was obtained following the general procedure **A** using 2-(thiophen-2-yl)indolizine-1-carbonitrile **1w** (44.9 mg, 0.2 mmol, 1.00 eq.). The crude was purified by column chromatography on silica (PE:EtOAc, 9:1) to get the pure product as a white wax: 50.1 mg, 0.16 mmol, 77%. **^{1}H NMR** (400 MHz, $CDCl_3$) δ 8.61 (d, J = 7.0 Hz, 1H), 7.84 (d, J = 3.8 Hz, 1H), 7.72 (d, J = 8.9 Hz, 1H), 7.51 (d, J = 5.1 Hz, 1H), 7.34 (dd, J = 8.9, 6.7 Hz, 1H), 7.20 (dd, J = 5.1, 3.7 Hz, 1H), 7.02 (t, J = 6.9 Hz, 1H).. **^{13}C{^{1}H} NMR** (100 MHz, $CDCl_3$) δ 141.3, 135.5, 131.5, 129.2, 128.4 (q, J = 316 Hz), 128.3, 126.9, 126.4, 125.2, 117.7, 115.7, 114.8, 100.6, 83.8. **^{19}F{^{1}H} NMR** (377 MHz, $CDCl_3$): δ -43.21 (s, 3F). **IR** (neat, cm^{-1}): 3092, 2924, 2853, 2206, 1505, 1468, 1359, 1241, 1093, 837, 750, 703, 488, 460. **ESI-HRMS**: m/z calcd. for $C_{14}H_7F_3N_2S_2Na$ $[M+Na]^+$ 346.9900, found 346.9895.

Ethyl 2-methyl-3-((trifluoromethyl)thio)indolizine-1-carboxylate (2x)

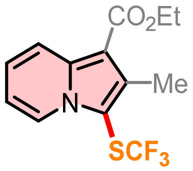

2x

Product **2x** was obtained following the general procedure **A** using ethyl 2-methylindolizine-1-carboxylate **1x** (40.6 mg, 0.2 mmol, 1.00 eq.). The crude was purified by column chromatography on silica (PE:EtOAc, 9:1) to get the pure product as a white wax: 60.7 mg, 0.20 mmol, quant.. **^{1}H NMR** (400 MHz, $CDCl_3$) δ 8.52 (d, J = 7.0 Hz, 1H), 8.26 (dt, J = 9.0, 1.1 Hz, 1H), 7.22 (ddd, J = 8.9, 6.7, 1.2 Hz, 1H), 6.95 – 6.80 (m, 1H), 4.40 (q, J = 7.1 Hz, 2H), 2.67 (s, 3H), 1.44 (t, J = 7.1 Hz, 3H). **^{13}C{^{1}H} NMR** (100 MHz, $CDCl_3$) δ 164.9, 140.3, 139.6, 133.5, 128.7 (q, J = 315 Hz), 125.2, 124.5, 124.1, 119.8, 113.4, 104.5, 102.4, 59.8, 14.7, 12.9. **^{19}F{^{1}H} NMR** (377 MHz, $CDCl_3$): δ -44.81 (s, 3F). **IR** (neat, cm^{-1}): 2983, 2927, 2855, 1681, 1496, 1422, 1385, 1324, 1216, 1164, 1098, 783, 750, 571, 485, 465, 435. **ESI-HRMS**: m/z calcd. for $C_{13}H_{12}F_3NO_2SNa$ $[M+Na]^+$ 326.0429, found 326.0433.

2-Methyl-3-((trifluoromethyl)thio)indolizine-1-carbonitrile (2y)

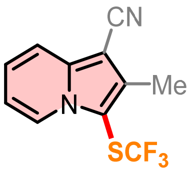

2y

Product **2y** was obtained following the general procedure **A** using 2-methylindolizine-1-carbonitrile **1y** (31.2 mg, 0.2 mmol, 1.00 eq.). The crude was purified by column chromatography on silica (PE:EtOAc, 9:1) to get the pure product as a white wax: 42.9 mg, 0.17 mmol, 84%. **^{1}H NMR** (400 MHz, $CDCl_3$) δ 8.52 (d, J = 7.0 Hz, 1H), 7.64 (dd, J = 8.8, 1.4 Hz, 1H), 7.37 – 7.21 (m, 1H), 7.05 – 6.88 (m, 1H), 2.55 (s, 3H). **$^{13}C\{^1H\}$ NMR** (100 MHz, $CDCl_3$) δ 140.5, 140.3, 133.2, 128.5 (q, J = 315 Hz), 125.5, 125.0, 123.8, 117.4, 115.2, 114.0, 102.0, 85.4, 11.5. **$^{19}F\{^1H\}$ NMR** (377 MHz, $CDCl_3$): δ -44.37 (s, 3F). **IR** (neat, cm^{-1}): 2926, 2855, 2211, 1632, 1507, 1357, 1239, 1155, 1106, 750, 502, 436, 434. **ESI-HRMS**: m/z calcd. for $C_{11}H_7F_3N_2SNa$ $[M+Na]^+$ 279.0180, found 279.0174 .

2-Methyl-1-(phenylsulfonyl)-3-((trifluoromethyl)thio)indolizine (2z)

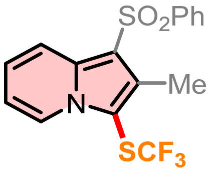

2z

Product **2z** was obtained following the general procedure **A** using 2-methyl-1-(phenylsulfonyl)indolizine **1z** (54.2 mg, 0.2 mmol, 1.00 eq.). The crude was purified by column chromatography on silica (PE:EtOAc, 6:1) to get the pure product as a white wax: 55.2 mg, 0.15 mmol, 74%. **^{1}H NMR** (400 MHz, $CDCl_3$) δ 8.53 (d, J = 7.0 Hz, 1H), 8.37 (d, J = 9.1 Hz, 1H), 7.95 (dd, J = 8.2, 1.4 Hz, 2H), 7.49 (ddd, J = 14.5, 8.0, 6.3 Hz, 3H), 7.39 – 7.28 (m, 1H), 6.97 (td, J = 6.9, 1.3 Hz, 1H), 2.61 (s, 3H). **$^{13}C\{^1H\}$ NMR** (100 MHz, $CDCl_3$) δ 144.0, 137.8, 132.7, 129.2, 128.5 (q, J = 316 Hz), 126.2, 126.2, 124.7, 123.8, 118.1, 114.2, 111.1, 103.5, 11.6. **$^{19}F\{^1H\}$ NMR** (377 MHz, $CDCl_3$): δ -44.25 (s, 3F). **IR** (neat, cm^{-1}): 3109, 2925, 2854, 1486, 1444, 1391, 1345, 1303, 1272, 1100, 764, 726, 689, 591, 548, 464. **ESI-HRMS**: m/z calcd. for $C_{16}H_{12}F_3NO_2S_2Na$ $[M+Na]^+$ 394.0159, found 394.0148.

2-Methyl-3-((trifluoromethyl)thio)indolizine (2aa)

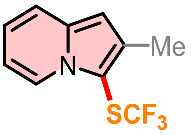

2aa

Product **2aa** was obtained following the general procedure **A** using 2-methylindolizine **1aa** (26.2 mg, 0.2 mmol, 1.00 eq.) with a slight modification. After the reaction time, the crude was immediately purified by column chromatography on silica (PE, 100%) to get the pure product as a colorless oil: 24.5 mg, 0.11 mmol, 53%. **^{1}H NMR** (400 MHz, $CDCl_3$) δ 8.42 (d, J = 7.0 Hz, 1H), 7.35 (dt, J = 8.9, 1.2 Hz, 1H), 6.90 (ddd, J = 8.9, 6.6, 1.1 Hz, 1H), 6.66 (td, J = 6.8, 1.3 Hz, 1H), 6.42 (s, 1H), 2.46 (s, 3H). **^{13}C{^{1}H} NMR** (100 MHz, $CDCl_3$) δ 137.3, 136.3, 133.7, 129.0 (q, J = 316 Hz), 124.3, 124.1, 120.8, 118.4, 117.1, 113.3, 111.1, 101.9, 98.9, 12.5. **^{19}F{^{1}H} NMR** (377 MHz, $CDCl_3$): δ -45.28 (s, 3F). **IR** (neat, cm^{-1}): 2926, 2855, 1390, 1273, 1102, 771, 731, 568, 462, 428. **ESI-HRMS**: m/z calcd. for $C_{20}H_{15}F_6N_2S_2$ $[2M+H]^+$ 461.0581, found 461.0573. *Note: 2-methylindolizine and potentially its derivatives are light-/heat-/air-sensitive compounds and therefore should be stored under inert conditions on cold.*

1-(Methylsulfonyl)-2-phenyl-3-((trifluoromethyl)thio)indolizine (2ab)

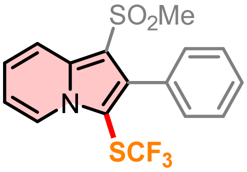

2ab

Product **2ab** was obtained following the general procedure **A** using 1-(methylsulfonyl)-2-phenylindolizine **1ab** (54.2 mg, 0.2 mmol, 1.00 eq.). The crude was purified by column chromatography on silica (PE:EtOAc, 4:1) to get the pure product as a colorless oil: 14.7 mg, 0.04 mmol, 20%. **^{1}H NMR** (400 MHz, $CDCl_3$) δ 8.66 (d, J = 7.0 Hz, 1H), 8.43 (dt, J = 9.2, 1.2 Hz, 1H), 7.52 – 7.42 (m, 5H), 7.38 (ddd, J = 9.1, 6.8, 1.1 Hz, 1H), 7.08 (td, J = 6.9, 1.3 Hz, 1H), 2.84 (s, 3H). **^{13}C{^{1}H} NMR** (100 MHz, $CDCl_3$) δ 141.0, 137.3, 131.0, 131.0, 129.0, 128.4 (q, J = 314 Hz), 128.0, 126.3, 124.8, 119.2, 114.7, 112.1, 104.2, 45.8. **^{19}F{^{1}H} NMR** (377 MHz, $CDCl_3$): δ -43.49 (s, 3F). **IR** (neat, cm^{-1}): 2926, 2854, 1491, 1350, 1296, 1168, 1123, 1099, 1022, 948, 785, 768, 748, 695, 630, 543, 510, 464, 447. **ESI-HRMS**: m/z calcd. for $C_{16}H_{12}F_3NO_2S_2Na$ $[M+Na]^+$ 394.0159, found 394.0154.

Diethyl 3-((trifluoromethyl)thio)indolizine-1,2-dicarboxylate (2ac)

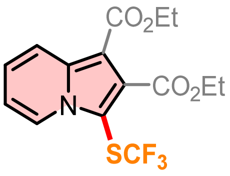

2ac

Product **2ac** was obtained following the general procedure **A** using diethyl indolizine-1,2-dicarboxylate **1ac** (52.2 mg, 0.2 mmol, 1.00 eq.). The crude was purified by column chromatography on silica (PE:EtOAc, 6:1) to get the pure product as a colorless oil: 15.7 mg, 0.04 mmol, 22%. **^{1}H NMR** (400 MHz, $CDCl_3$) δ 8.55 (d, J = 7.0 Hz, 1H), 8.33 (dt, J = 9.2, 1.4 Hz, 1H), 7.34 (ddd, J = 8.7, 6.4, 0.9 Hz, 1H), 7.03 (td, J = 6.9, 1.3 Hz, 1H), 4.48 (q, J = 7.1 Hz, 2H), 4.37 (q, J = 7.1 Hz, 2H), 1.44 – 1.37 (m, 6H). **^{13}C{^{1}H} NMR** (100 MHz, $CDCl_3$) δ 164.8, 162.9, 138.6, 134.8, 133.0, 128.3 (q, J = 315 Hz), 126.2, 124.6, 120.5, 115.0, 104.0, 101.6, 62.1, 60.5, 14.5, 14.3. **^{19}F{^{1}H} NMR** (377 MHz, $CDCl_3$): δ -43.53 (s, 3F). **IR** (neat, cm^{-1}): 2987, 2928, 2855, 1728, 1693, 1506, 1437, 1212, 1159, 1104, 1015, 788, 758, 573, 460. **ESI-HRMS**: m/z calcd. for $C_{15}H_{14}F_3NO_4SNa$ $[M+Na]^+$ 384.0493, found 384.0488.

6.3. Scale-up experiment for trifluoromethylthiolation of 1a

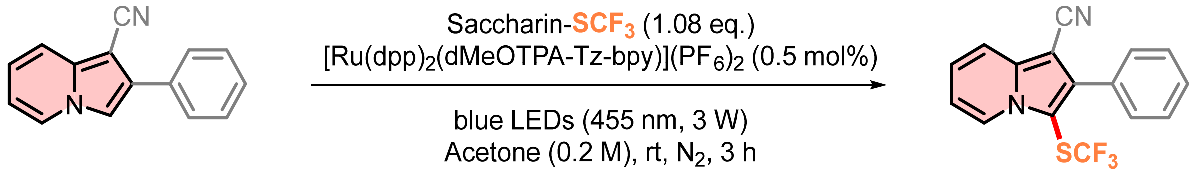

1a
1.8 mmol scale

2a

In a glovebox, to a vial filled with indolizine **1a** (392.9 mg, 1.80 mmol, 1.00 eq.), Saccharin-SCF_3 (550.0 mg, 1.94 mmol, 1.08 eq.), PC1 (14.2 mg, 9.0 µmol, 0.5 mol%) was added Acetone (9.0 mL, 0.2 M). The vial was sealed and the mixture was irradiated with a blue LED (3 W, 455 nm) for 3 h at rt outside of the glovebox. The reaction mixture was partitioned between H_2O and Et_2O and the organic phase was extracted. The water phase was extracted with Et_2O 3 times and the combined organic phases were washed with H_2O and brine. The organic phase was dried over Na_2SO_4 and the solvent was removed under reduced pressure. The residue was purified by flash column chromatography on silica gel (PE:EtOAc, 9:1) to afford the trifluoromethylthiolated products **2a** as an off-white wax: 513.2 mg, 1.61 mmol, 90%. Spectral data sets were in agreement with previous measurements.

6.4. Derivatization of 3-((trifluoromethyl)thio)indolizines

2-(4-(3-(Piperidin-1-yl)propoxy)phenyl)-3-((trifluoromethyl)thio)indolizine (3)

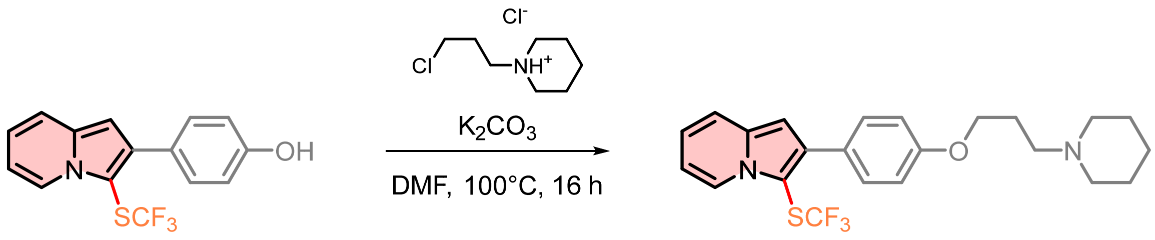

2i **3**

Indolizine **2i** (13.8 mg, 0.045 mmol, 1.00 eq.), 1-(3-chloropropyl)piperidin-1-ium chloride (10.6 mg, 0.054 mmol, 1.20 eq.) and K_2CO_3 (12.3 mg, 0.089 mmol, 2.00 eq.) are suspended in anhydrous DMF (1 mL, 0.05 M). The reaction mixture is stirred overnight under air at 100°C and it is later cooled to rt. The mixture is partitioned between DCM/H_2O and the organic phase is separated. The water phase was extracted with DCM 3 times and the combined organic phases were washed with H_2O and brine. The organic phase was dried over Na_2SO_4 and the solvent was removed under reduced pressure. The residue was purified by flash column chromatography on silica gel (DCM:MeOH, 97:3 to 9:1) to afford the trifluoromethylthiolated product **3** (20.0 mg, 0.045 mmol, quant.) as a pale yellow oil. **^{1}H NMR** (400 MHz, $CDCl_3$) δ 8.52 (d, J = 7.1 Hz, 1H), 7.73 – 7.54 (m, 2H), 7.49 – 7.37 (m, 1H), 7.06 – 6.84 (m, 3H), 6.84 – 6.61 (m, 2H), 4.10 (t, J = 5.9 Hz, 2H), 3.11 – 2.65 (m, 6H), 2.27 (dq, J = 11.9, 6.0 Hz, 2H), 1.88 (p, J = 5.7 Hz, 4H), 1.65 – 1.50 (m, 2H). **^{13}C{^{1}H} NMR** (100 MHz, $CDCl_3$) δ 158.3, 139.4, 137.4, 130.5, 125.8 (q, J = 314 Hz), 124.3, 118.8, 114.4, 111.8, 101.4, 97.1, 70.7, 65.8, 55.8, 54.1, 29.8, 25.3, 24.2, 23.3. **^{19}F{^{1}H} NMR** (377 MHz, $CDCl_3$): δ -44.41 (s, 3F). **IR** (neat, cm^{-1}): 3411, 2934, 2857, 2517, 1610, 1508, 1466, 1242, 1097, 832, 780, 728. **ESI-HRMS**: m/z calcd. for $C_{23}H_{26}F_3N_2OS$ [M+H]$^+$ 435.1718, found 435.1710.

7. Absorption, emission and electrochemical properties

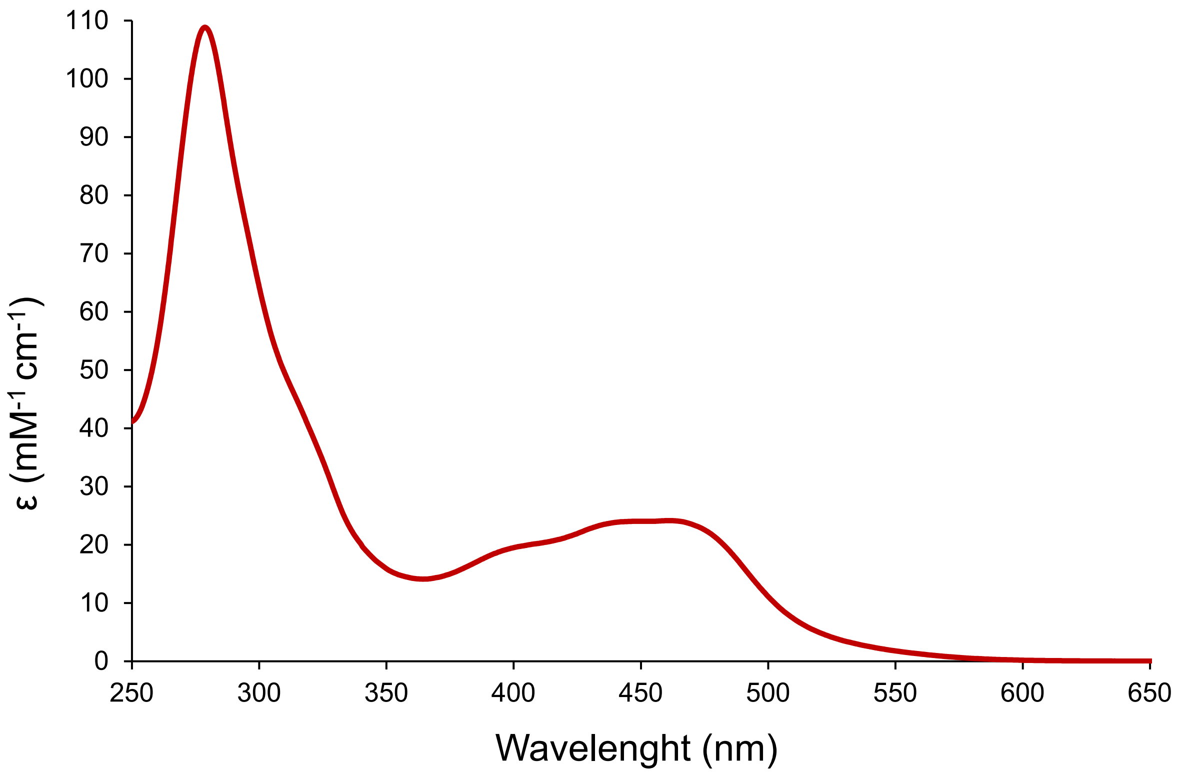

Figure S4. UV-vis spectrum of **PC1** (5 μM) in MeCN.[1]

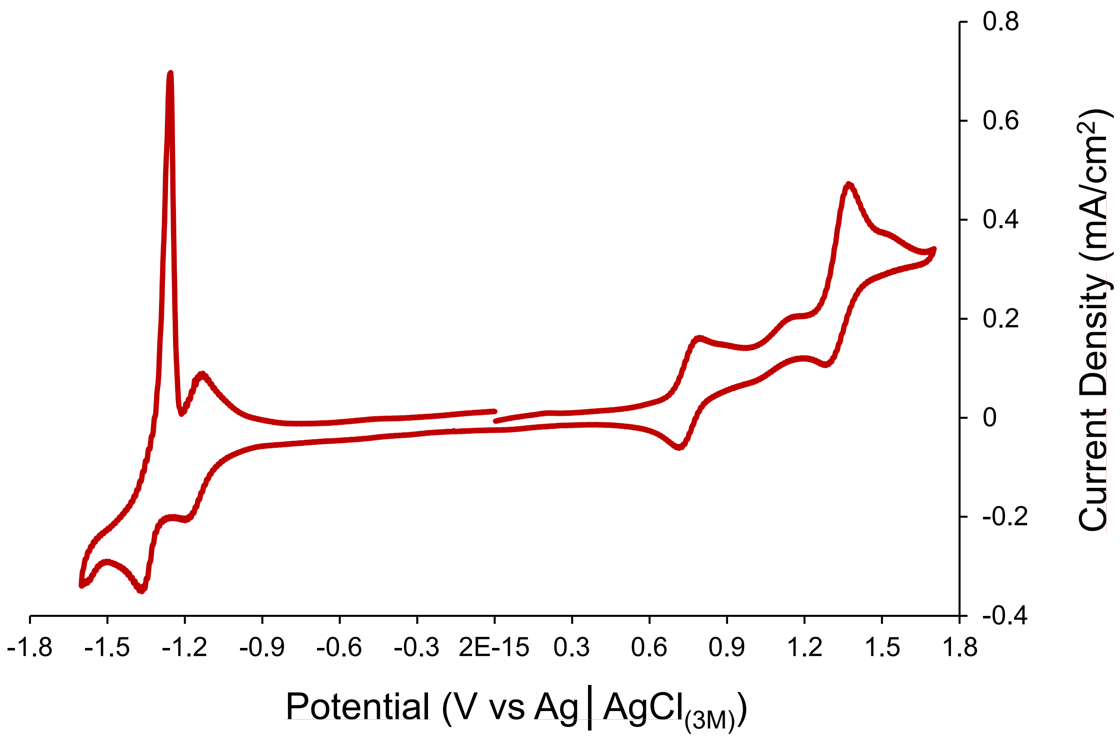

Figure S5. Cyclic voltammogram of **PC1** (1 mM) in a supporting electrolyte 0.1 M [Bu_4N][PF_6] in MeCN referenced to Ag/Ag^+ couple at 0.1 Vs^{-1} sweep rate.[1]

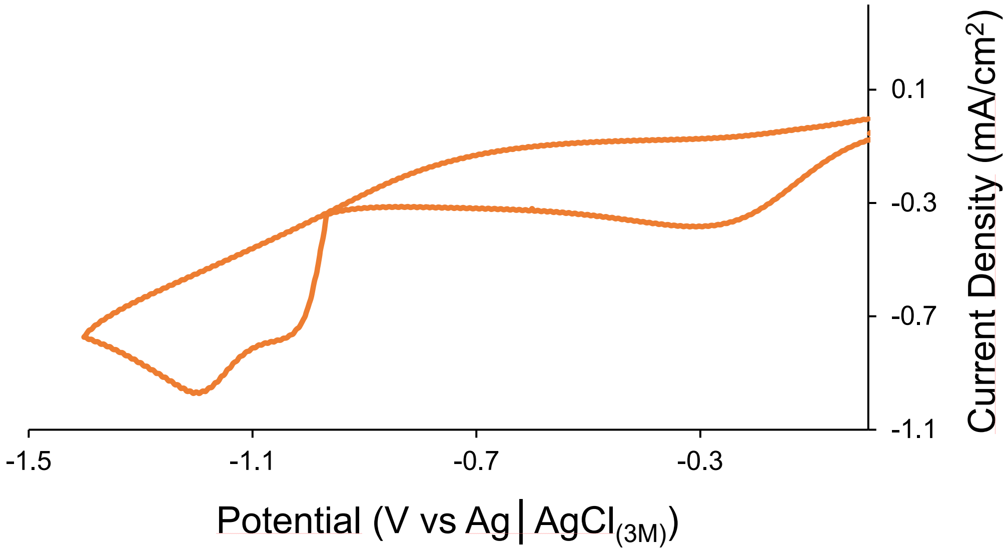

Figure S6. Cyclic voltammogram of **Saccharin-SCF$_3$** (2 mM) in a supporting electrolyte 0.1 M [Bu$_4$N][PF$_6$] in MeCN referenced to Ag/Ag$^+$ couple at 0.1 Vs^{-1} sweep rate.

8. Proposed reaction mechanism

Based on our previous work[10] and other literature reports,[12–14] a plausible reaction mechanism for the indolizine photocatalytic trifluoromethylthiolation can be proposed (Scheme S1). First, photoredox catalyst **PC1** is excited (from Ru^{2+} (**L1**) to Ru^{2+} *(**L1**) upon blue light irradiation). The interaction between Ru^{2+} *(**L1**) and the radical source, Saccharin-SCF_3 (E_p = −0.35 V vs SCE), can generate the SCF_3 radical via Single-Electron-Transfer (SET), because of the high reducing power of **PC1** (E^0 [Ru^{2+} (**L1**)$^{•+}$/Ru^{2+} *(**L1**)] = −1.30 V vs SCE). The formed SCF_3 radical can be intercepted by indolizine **1a** to produce **INT-I**. The resulting radical species can be then oxidized by Ru^{2+} (**L1**)$^{•+}$ to a carbocation **INT-II**, as the reduction potential of the oxidized catalyst is sufficiently high (E^0 [Ru^{2+} (**L1**)$^{•+}$/Ru^{2+} (**L1**)] = +0.84 V vs SCE). This process regenerates the catalyst and closes the photoredox cycle. Ultimately, the deprotonated Saccharin readily abstracts a proton from **INT-II**, thereby restoring aromaticity and yielding the desired product **2a**.

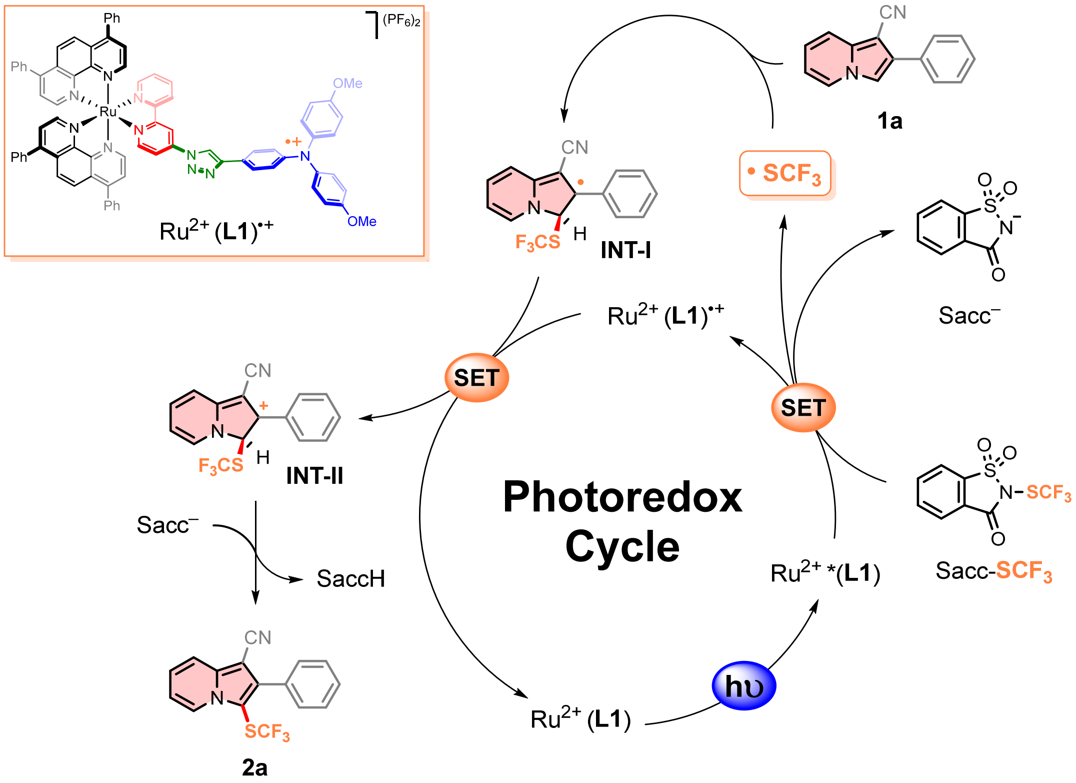

Scheme S1. Proposed reaction mechanism.

9. NMR spectra

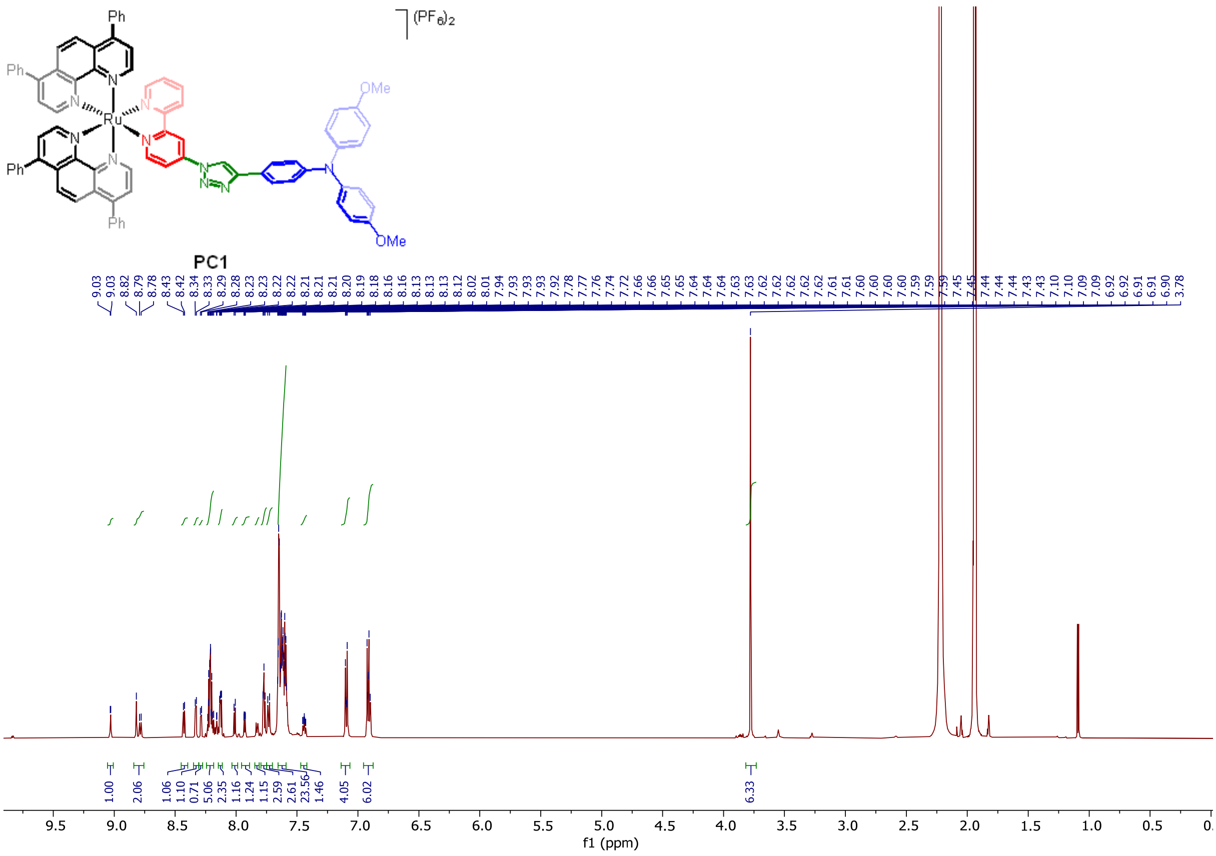

^{1}H NMR (600 MHz, CD_3CN) of **PC1**.

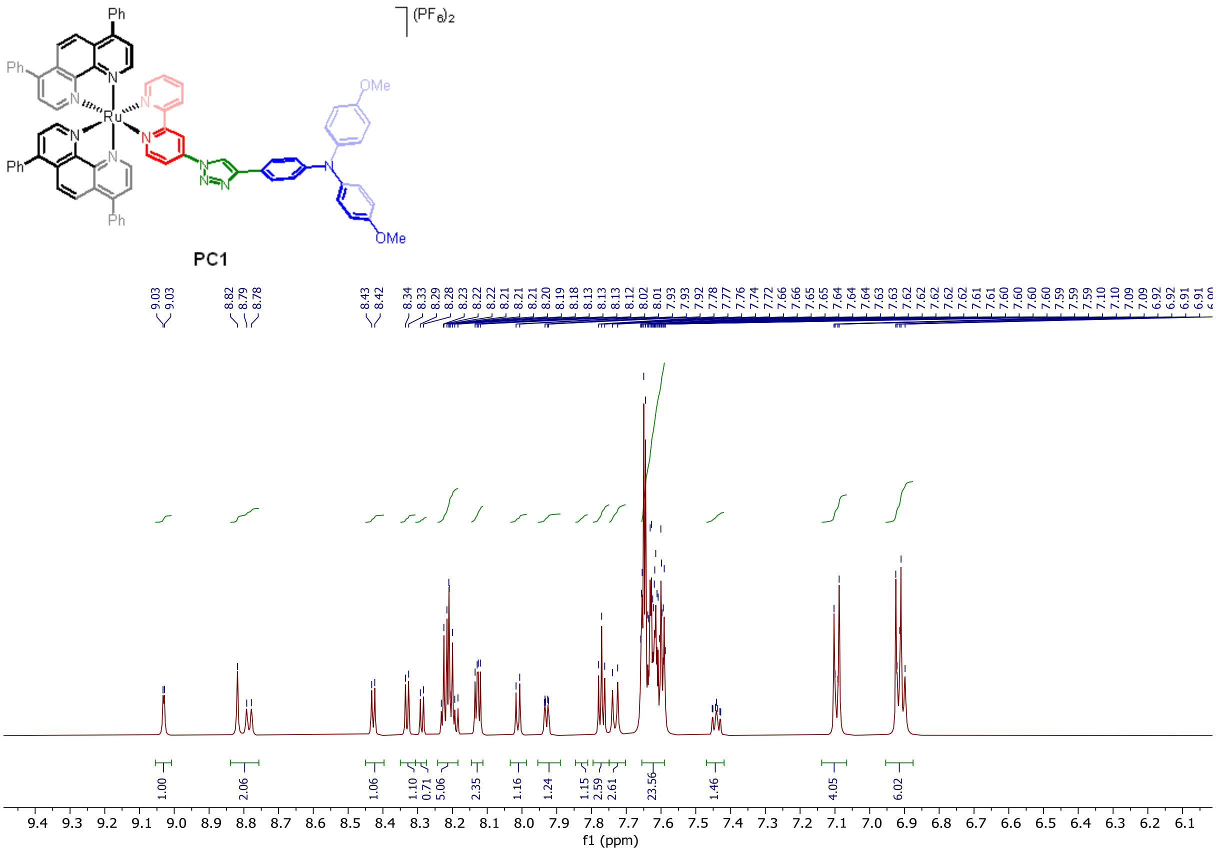

^{1}H NMR (600 MHz, CD_3CN) of **PC1** (expansion aromatic area 6.0 – 9.5 ppm).

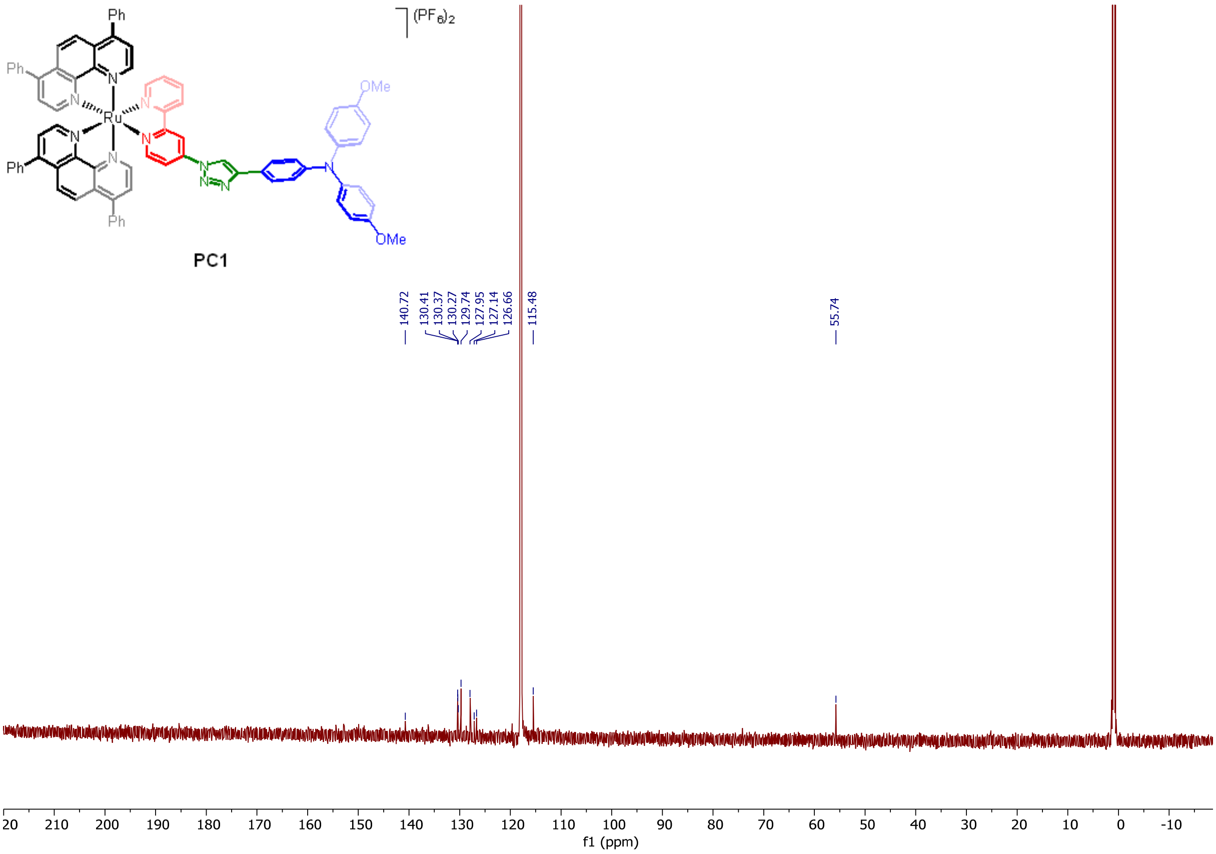

^{13}C{^{1}H} NMR (150 MHz, CD_3CN) of **PC1**.

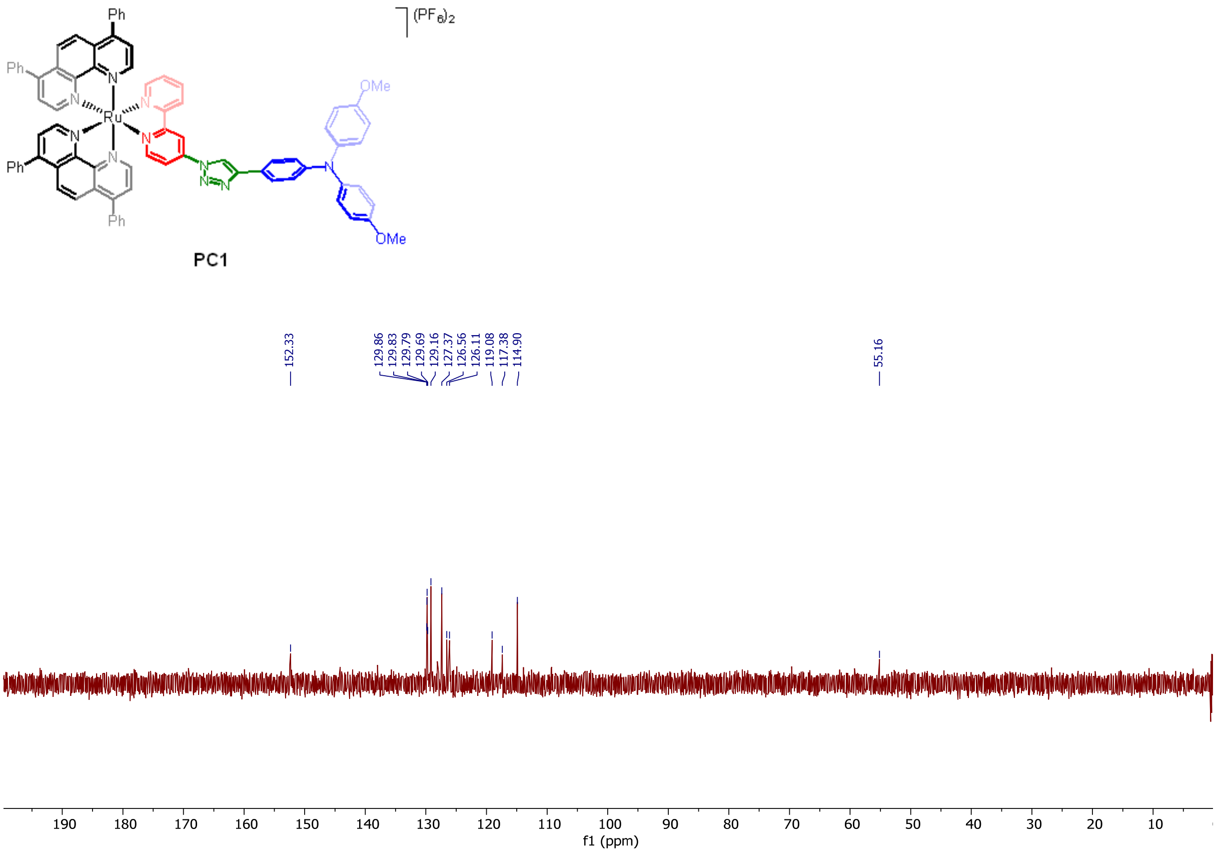

^{13}C-DEPT135 NMR (150 MHz, CD_3CN) of **PC1**.

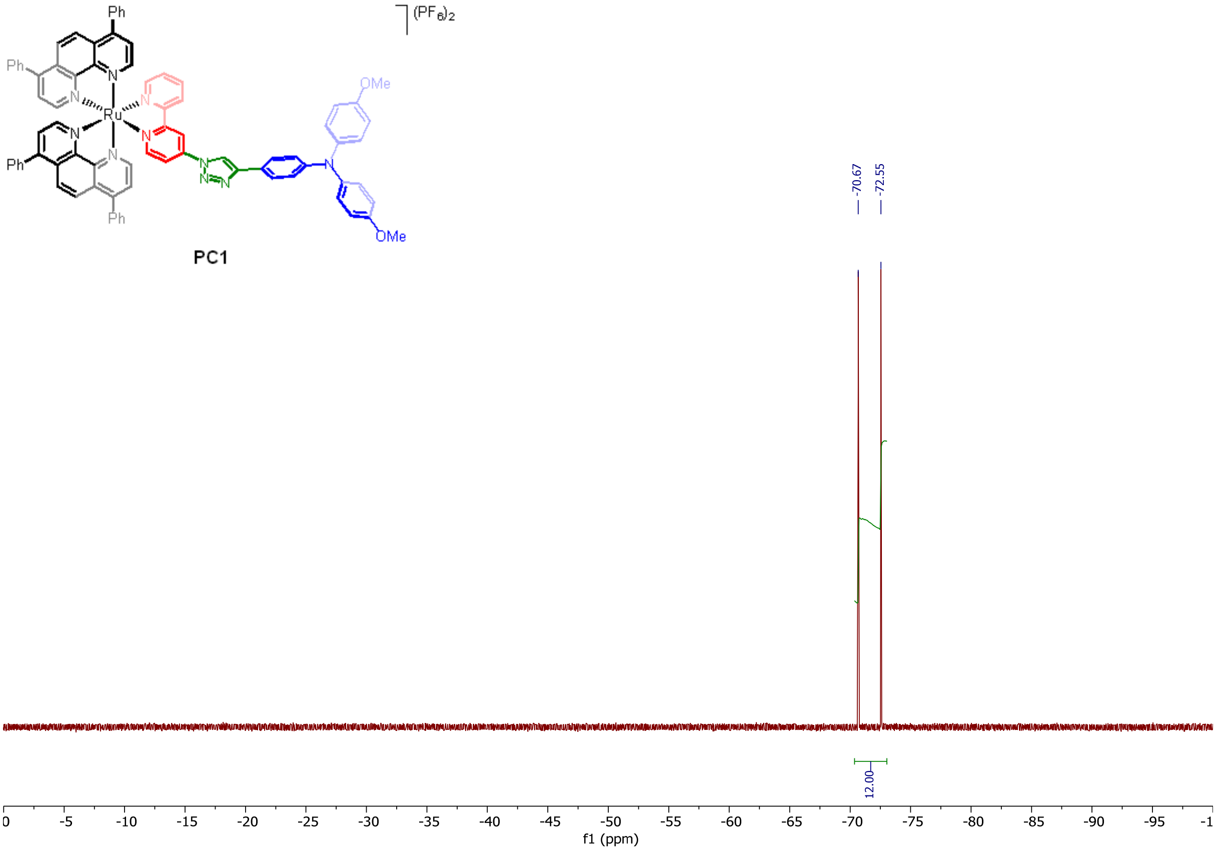

^{19}F{^{1}H} NMR (377 MHz, CD_3CN) of **PC1**.

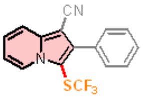

2a

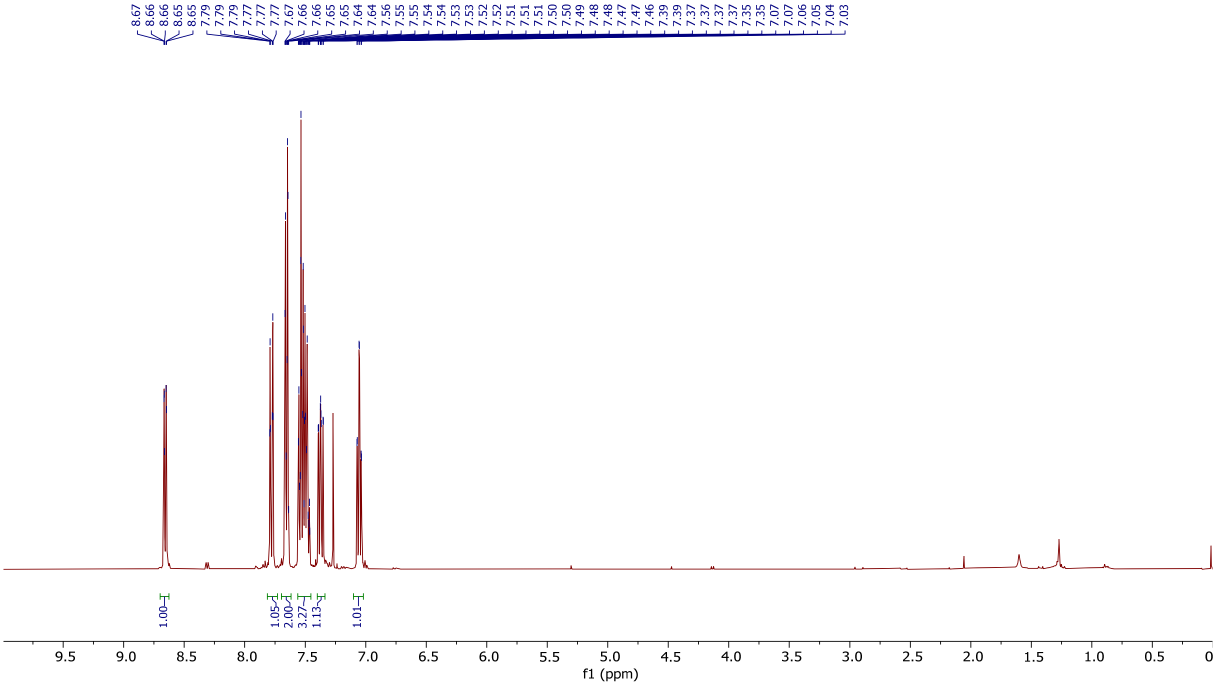

^{1}H NMR (400 MHz, $CDCl_3$) of **2a**.

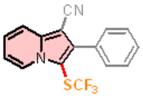

2a

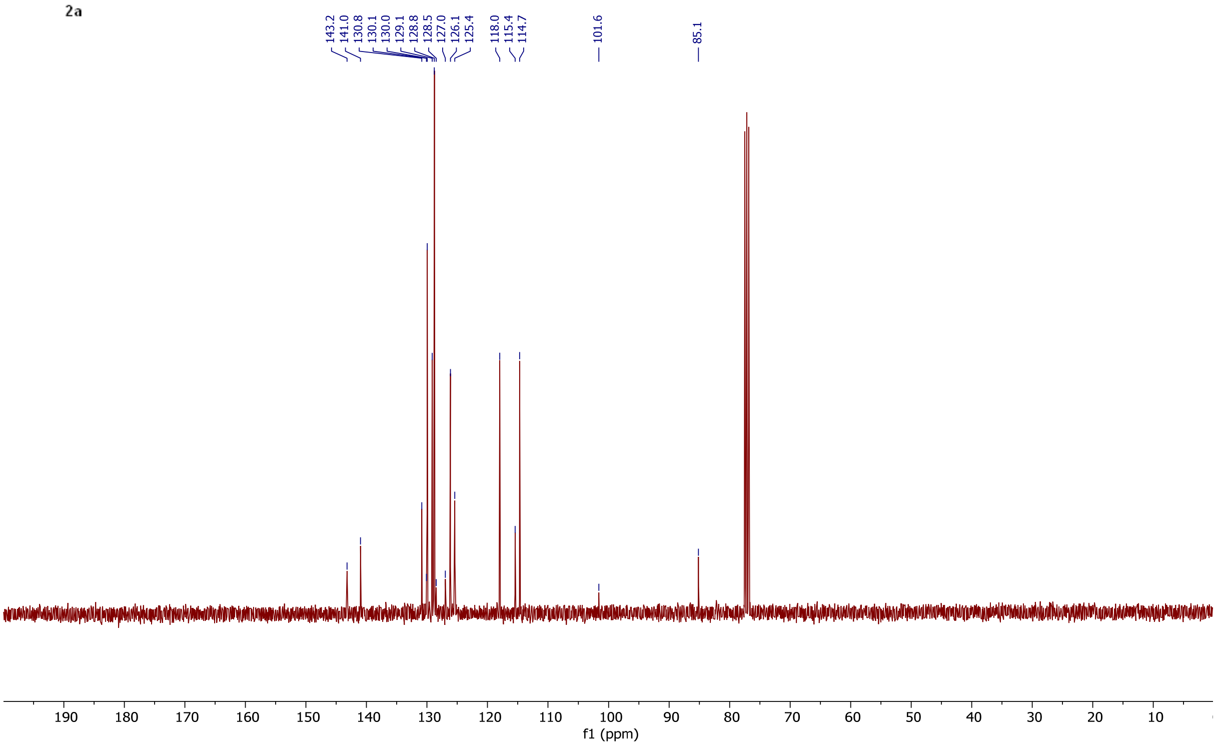

^{13}C{^{1}H} NMR (100 MHz, $CDCl_3$) of **2a**.

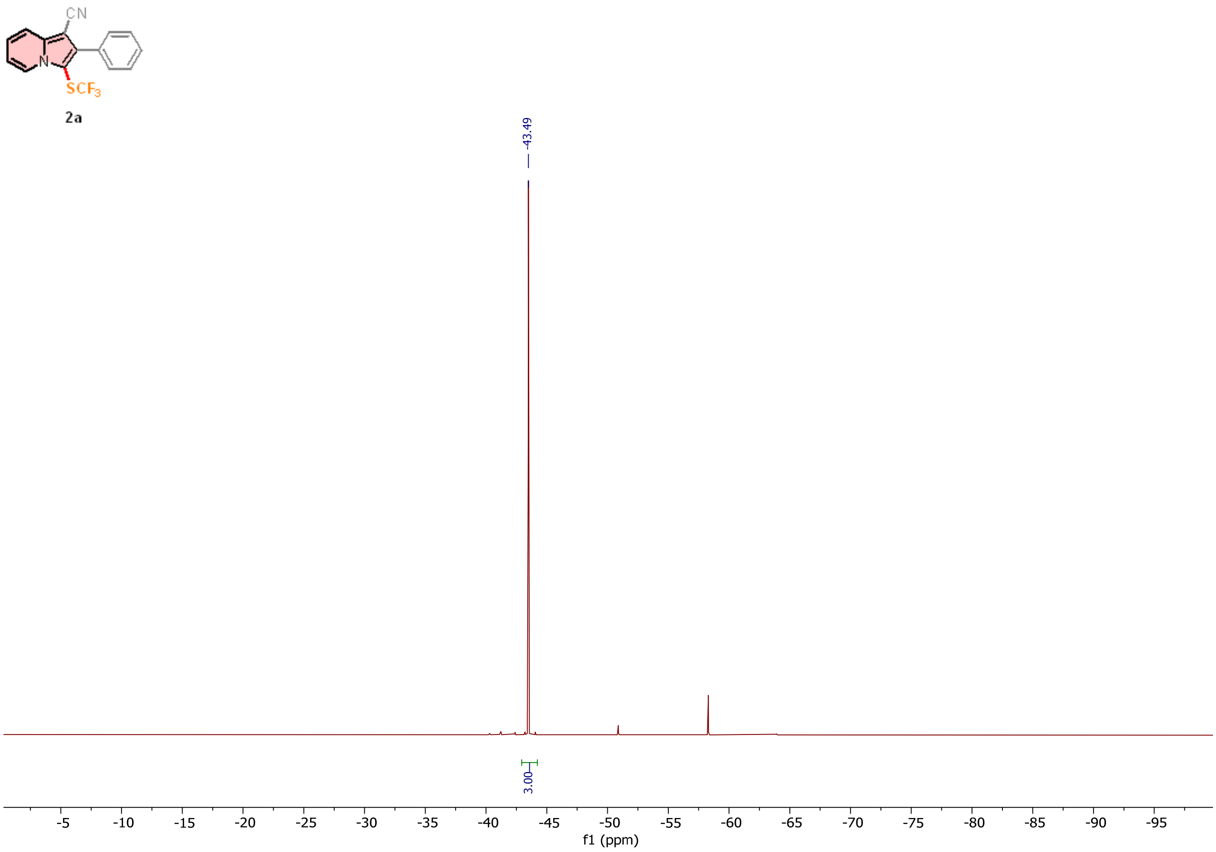

$^{19}F\{^1H\}$ NMR (377 MHz, $CDCl_3$) of **2a**.

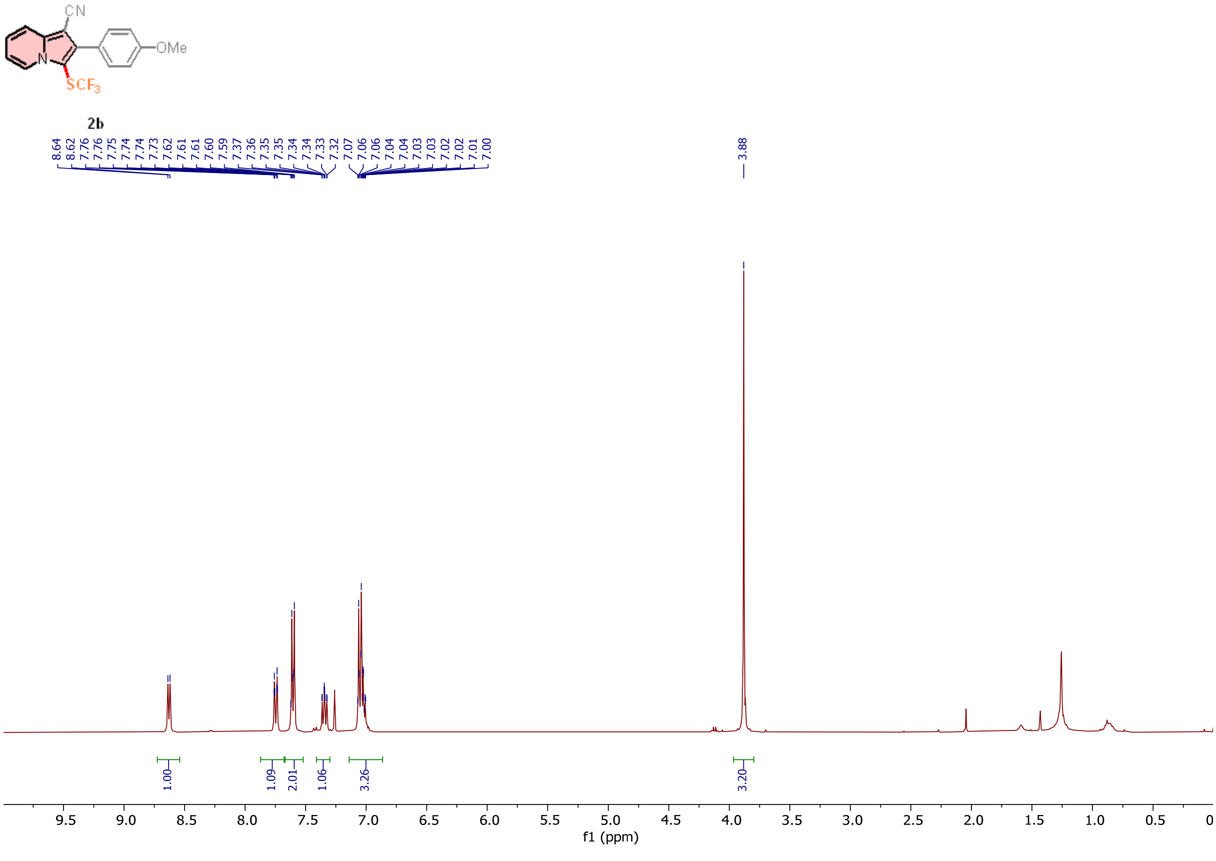

^1H NMR (400 MHz, $CDCl_3$) of **2b**.

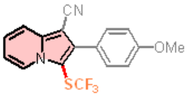

2b

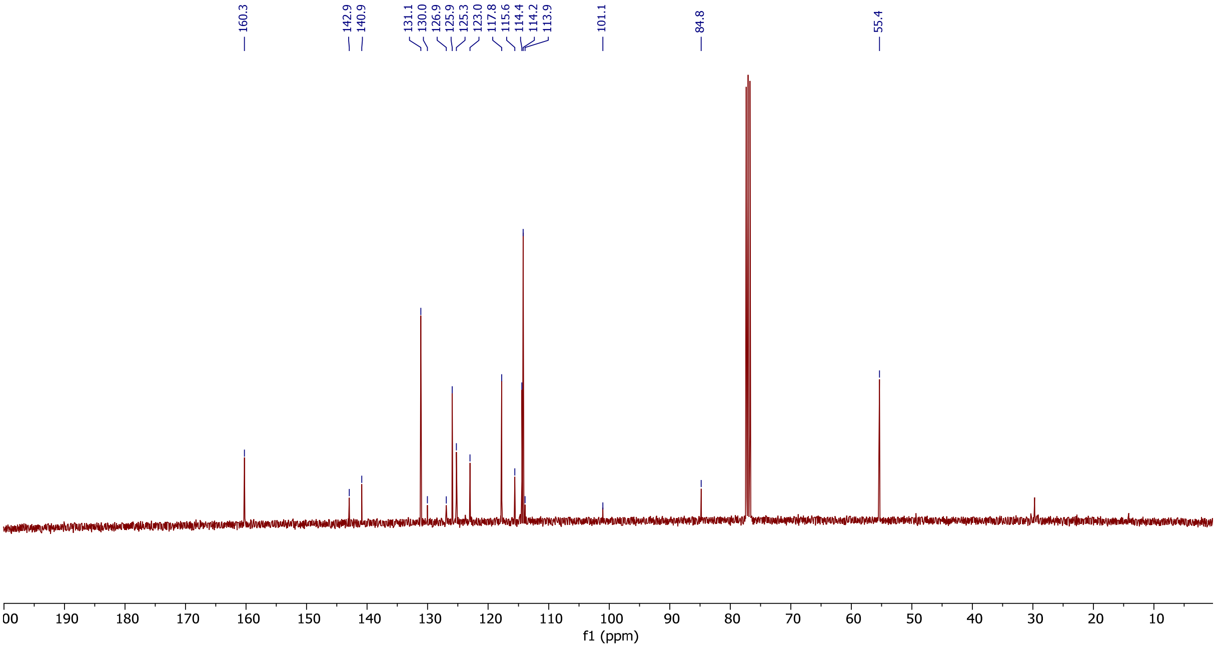

$^{13}C\{^1H\}$ NMR (100 MHz, $CDCl_3$) of **2b**.

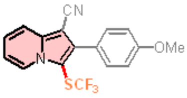

2b

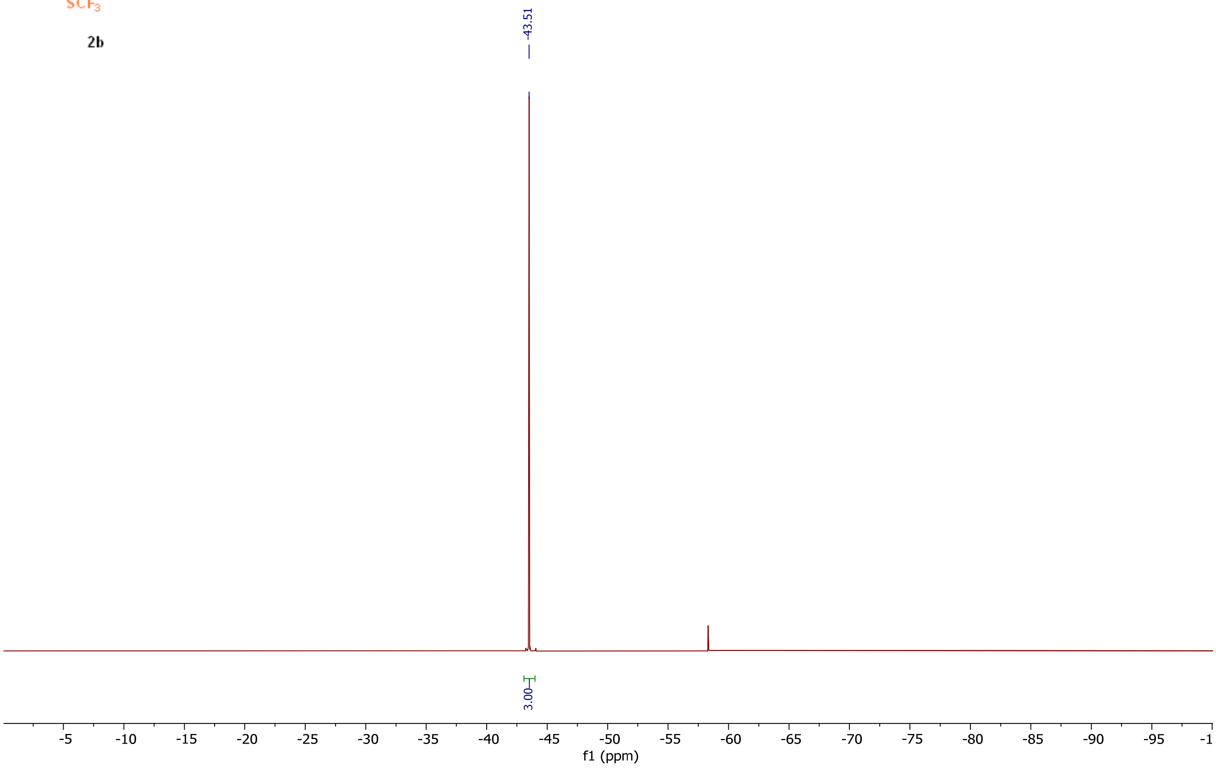

$^{19}F\{^1H\}$ NMR (377 MHz, $CDCl_3$) of **2b**.

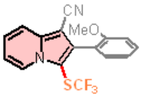

2c

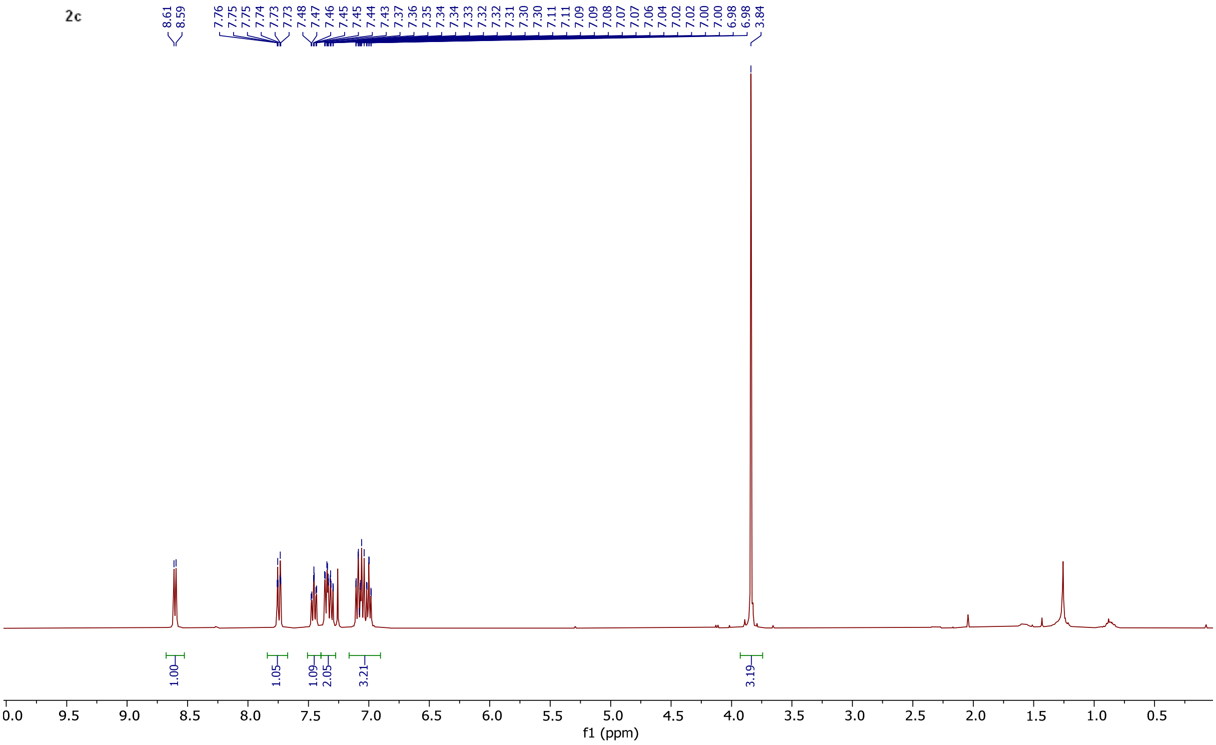

^{1}H NMR (400 MHz, $CDCl_3$) of **2c**.

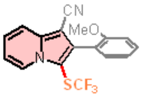

2c

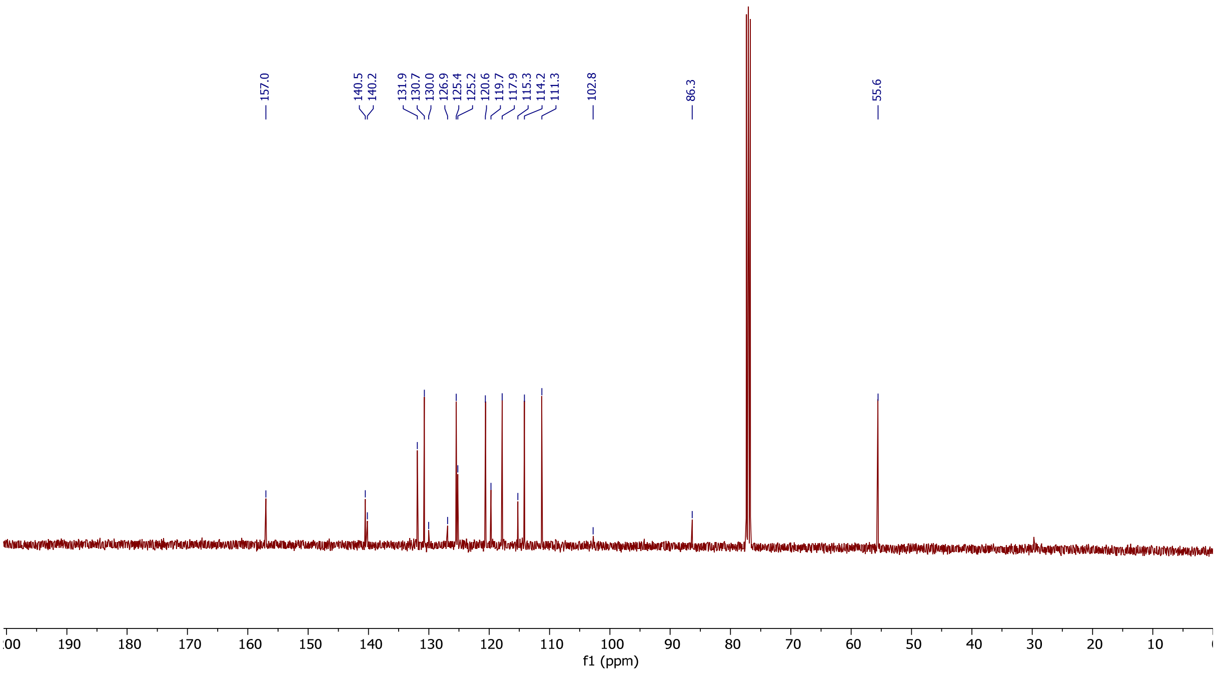

^{13}C{^{1}H} NMR (100 MHz, $CDCl_3$) of **2c**.

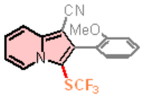

2c

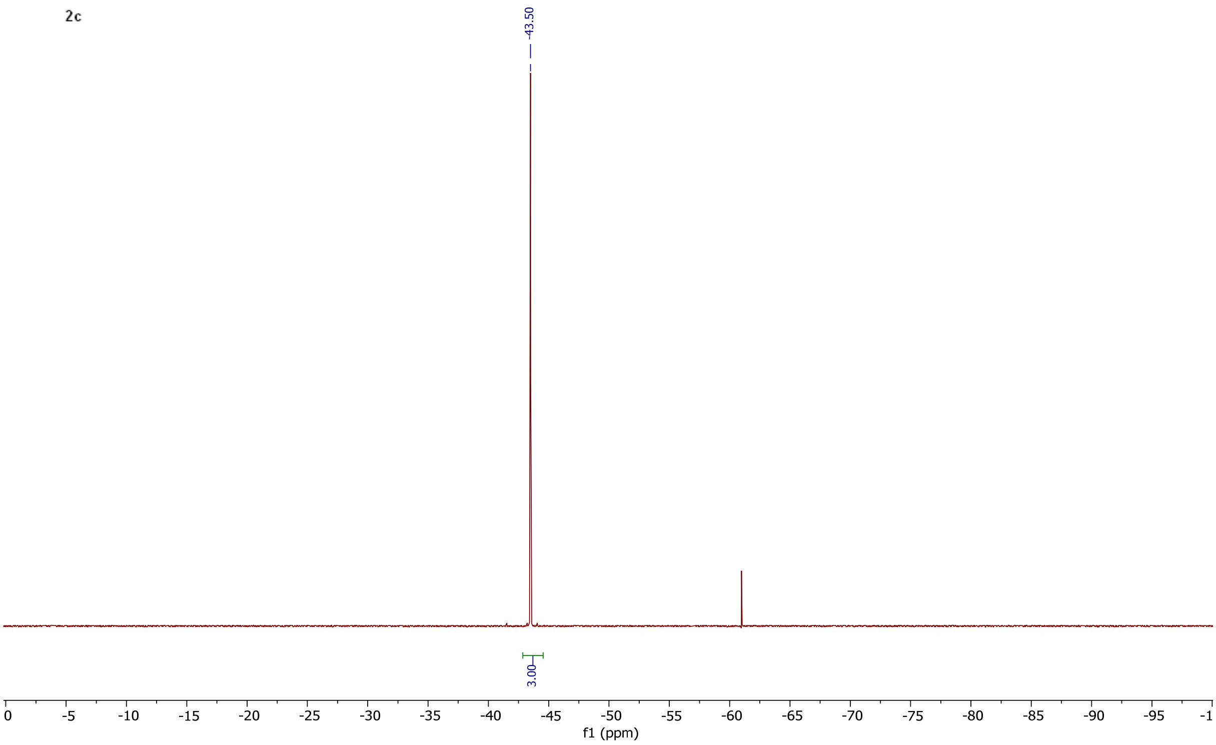

$^{19}F\{^{1}H\}$ NMR (377 MHz, $CDCl_3$) of **2c**.

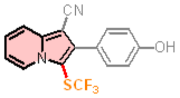

2d

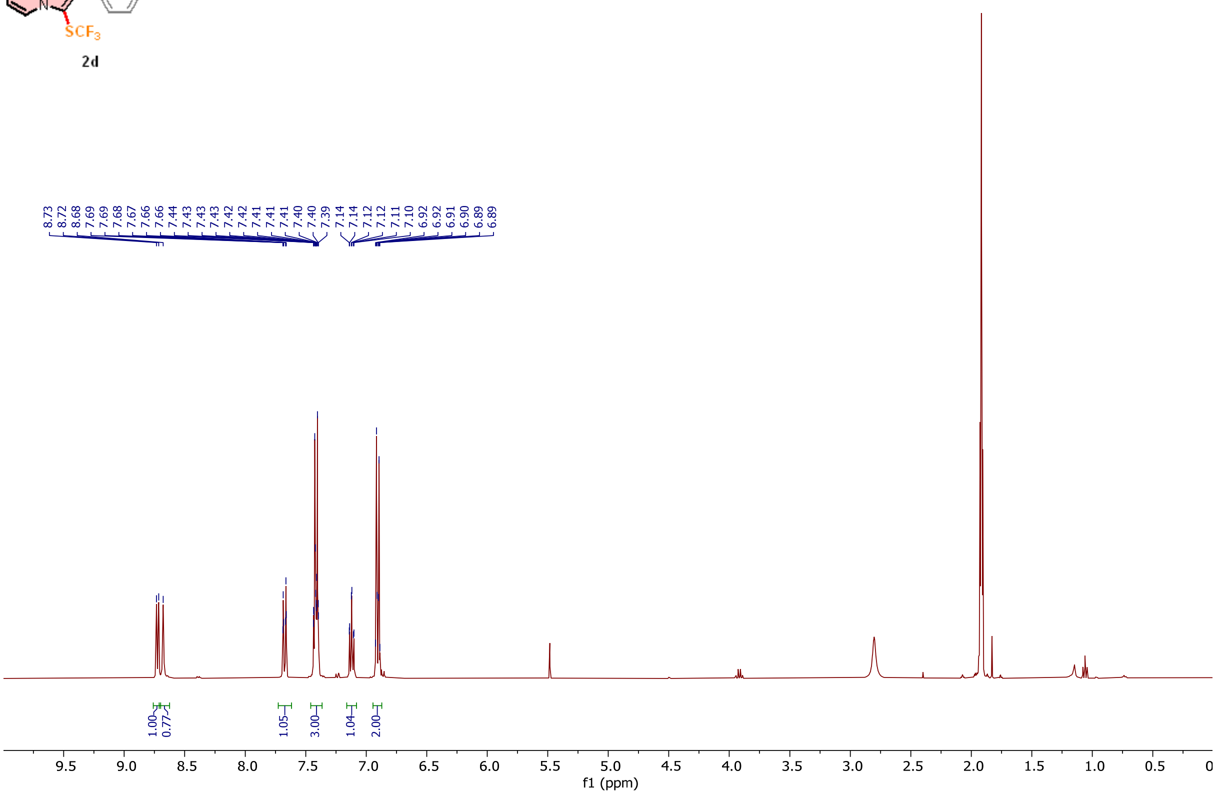

^{1}H NMR (400 MHz, Acetone-d^6) of **2d**.

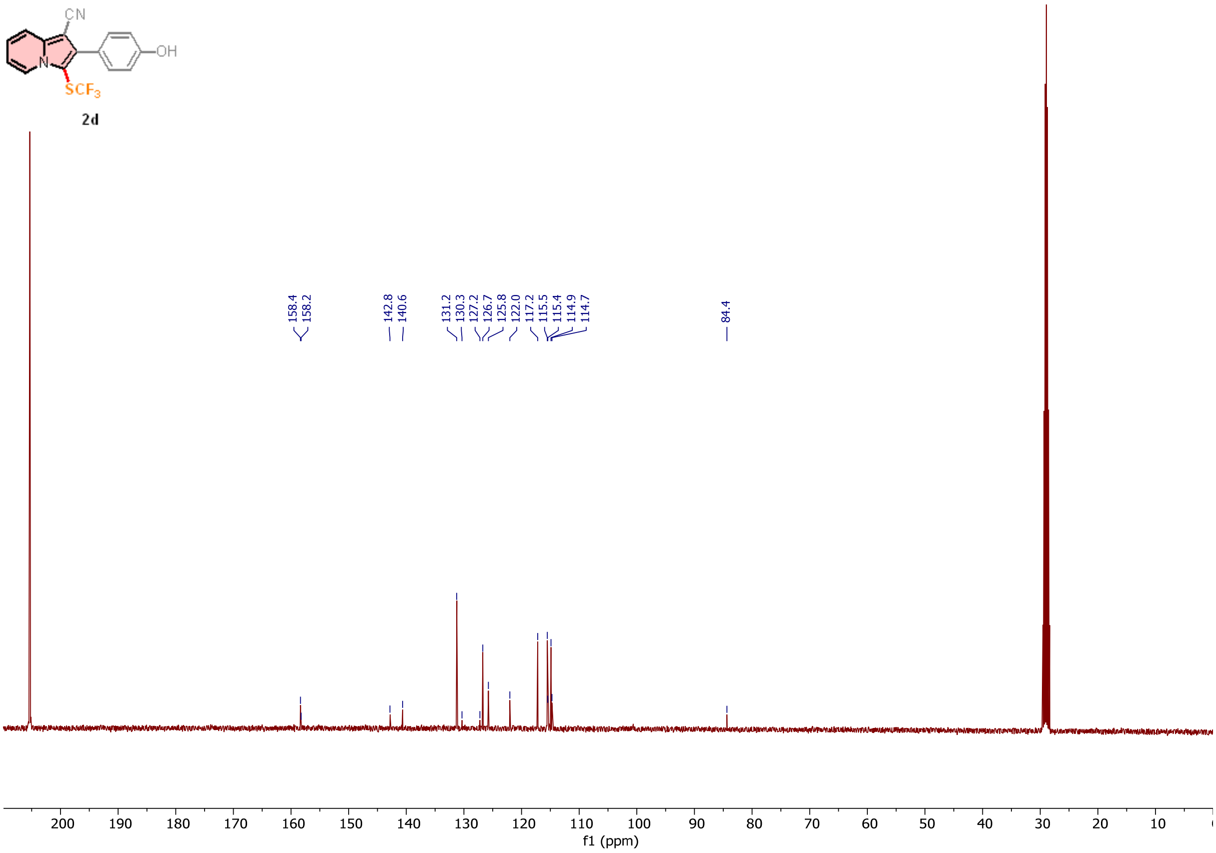

$^{13}C\{^{1}H\}$ NMR (100 MHz, Acetone-d^{6}) of **2d**.

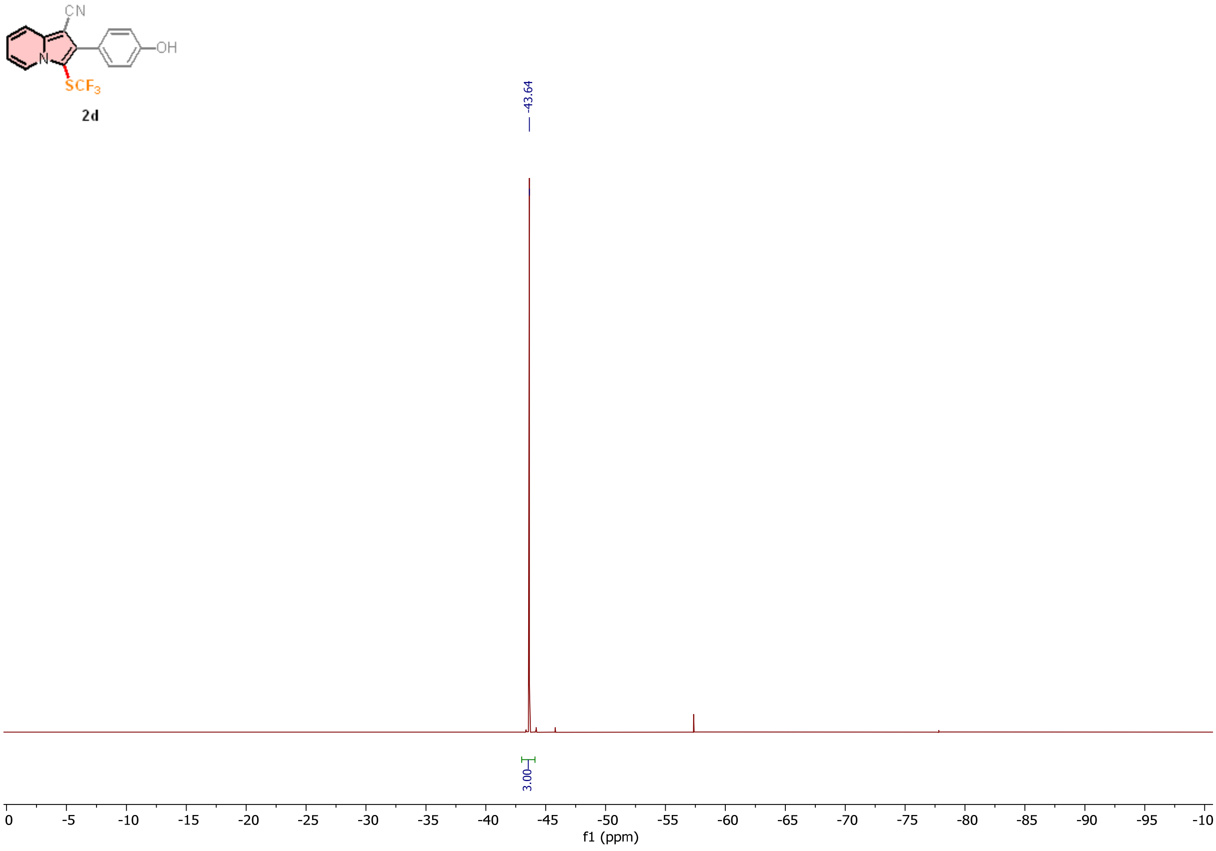

$^{19}F\{^{1}H\}$ NMR (377 MHz, Acetone-d^{6}) of **2d**.

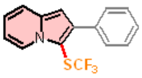

2e

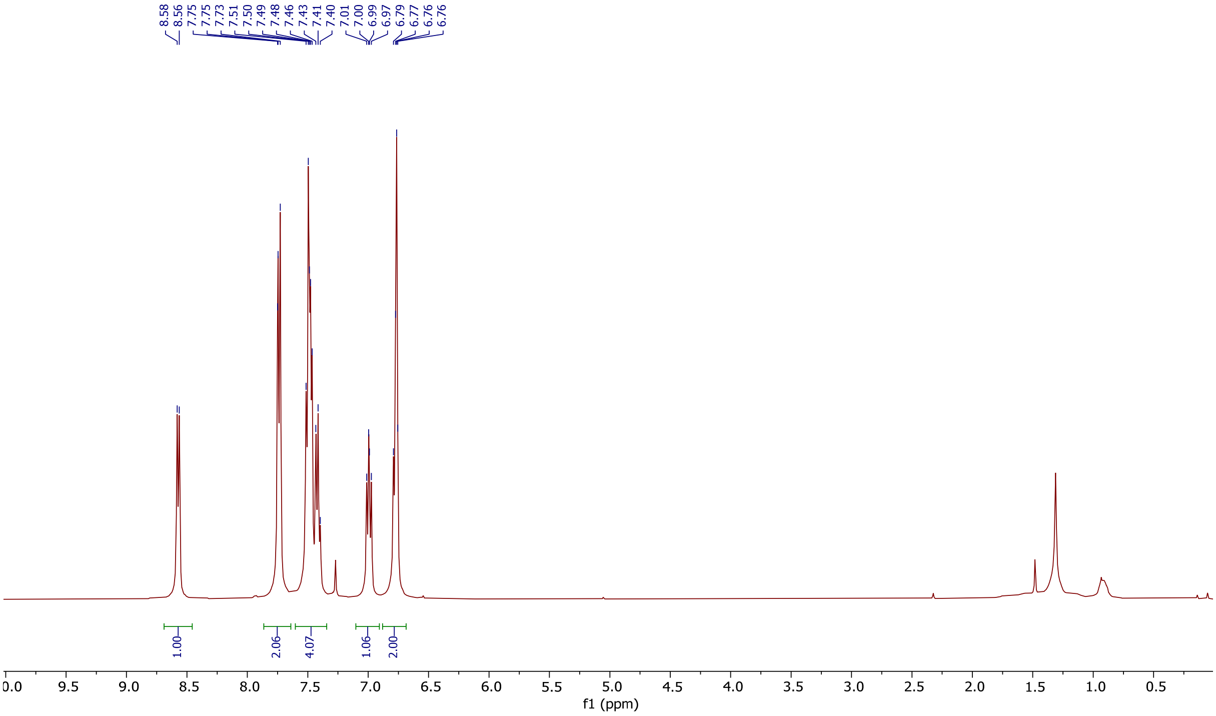

^{1}H NMR (400 MHz, $CDCl_3$) of **2e**.

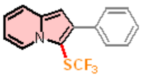

2e

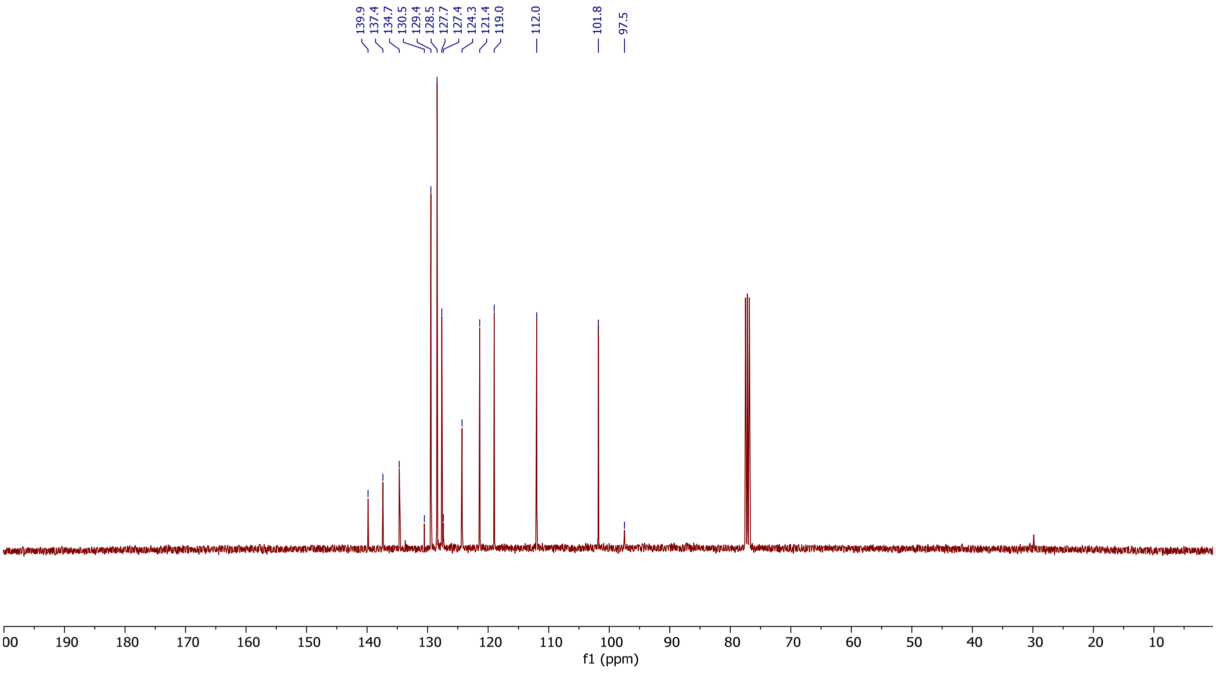

$^{13}C\{^1H\}$ NMR (100 MHz, $CDCl_3$) of **2e**.

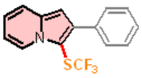

2e

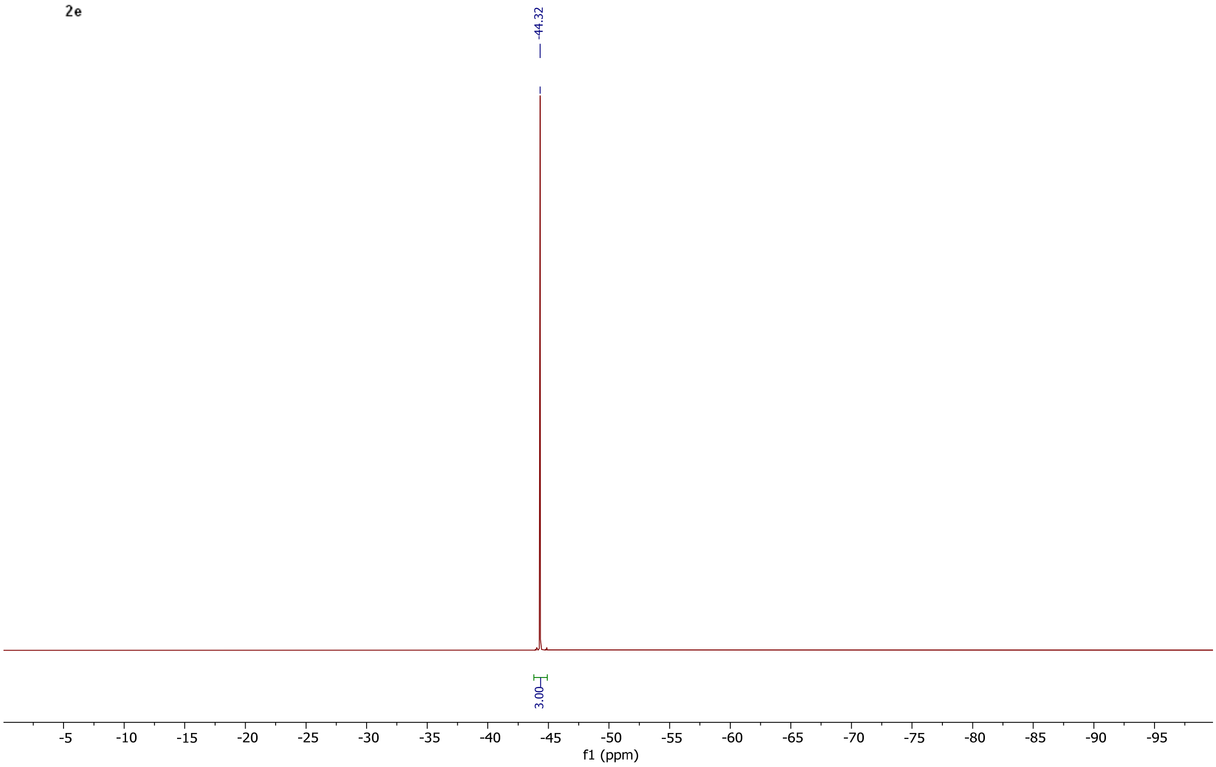

$^{19}F\{^{1}H\}$ NMR (377 MHz, $CDCl_3$) of **2e**.

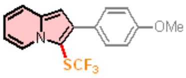

2f

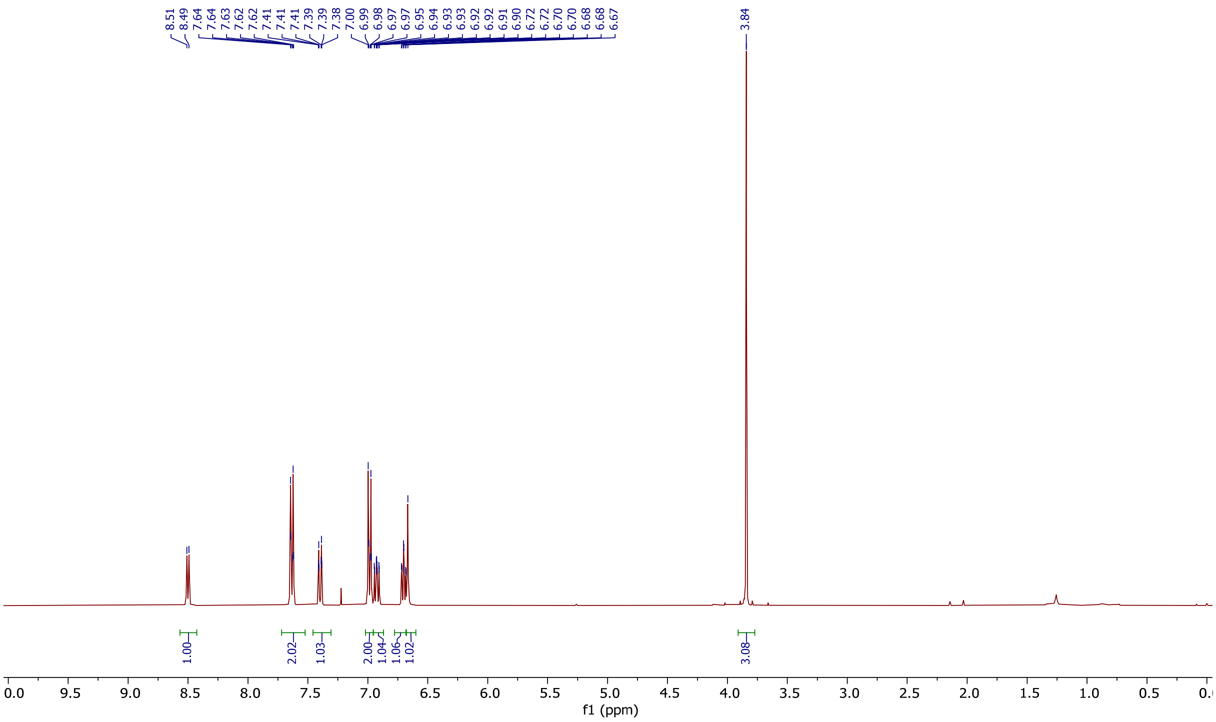

^{1}H NMR (400 MHz, $CDCl_3$) of **2f**.

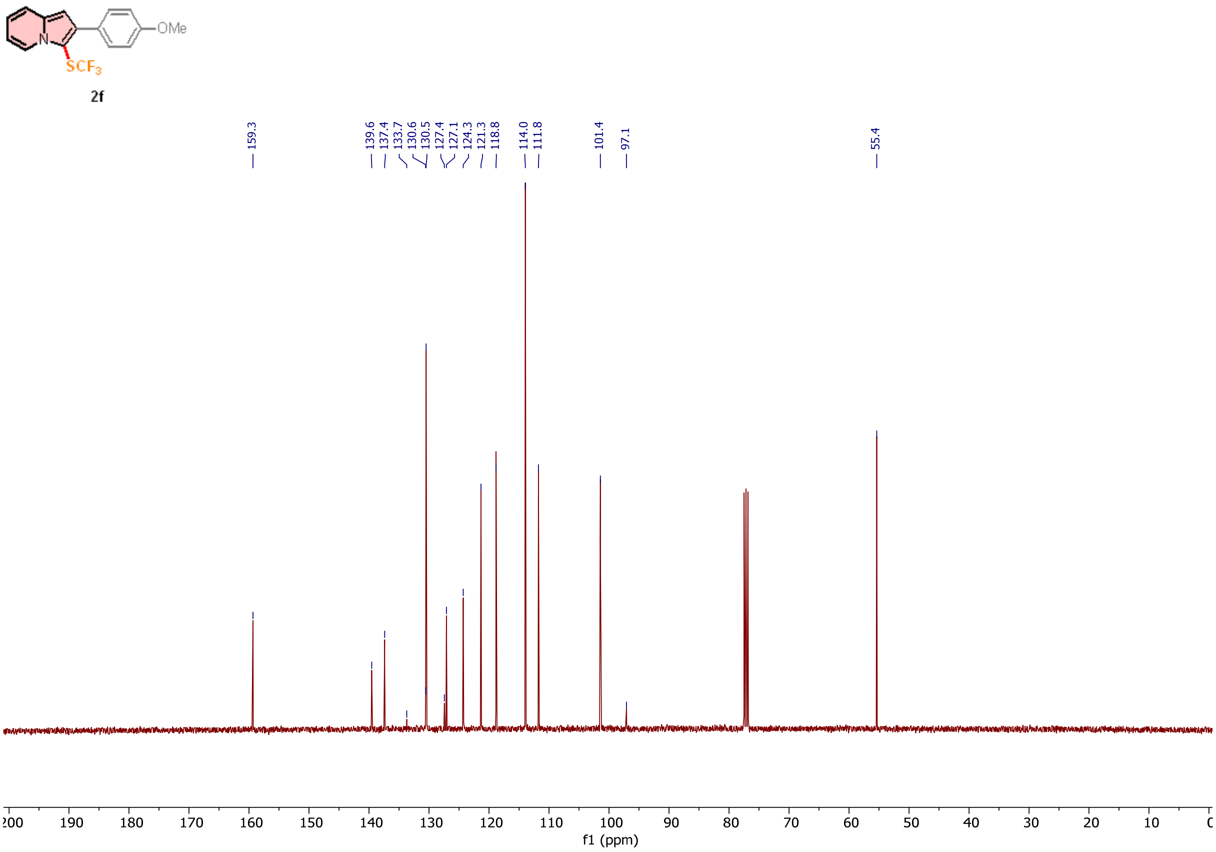

$^{13}C\{^{1}H\}$ NMR (100 MHz, $CDCl_3$) of **2f**.

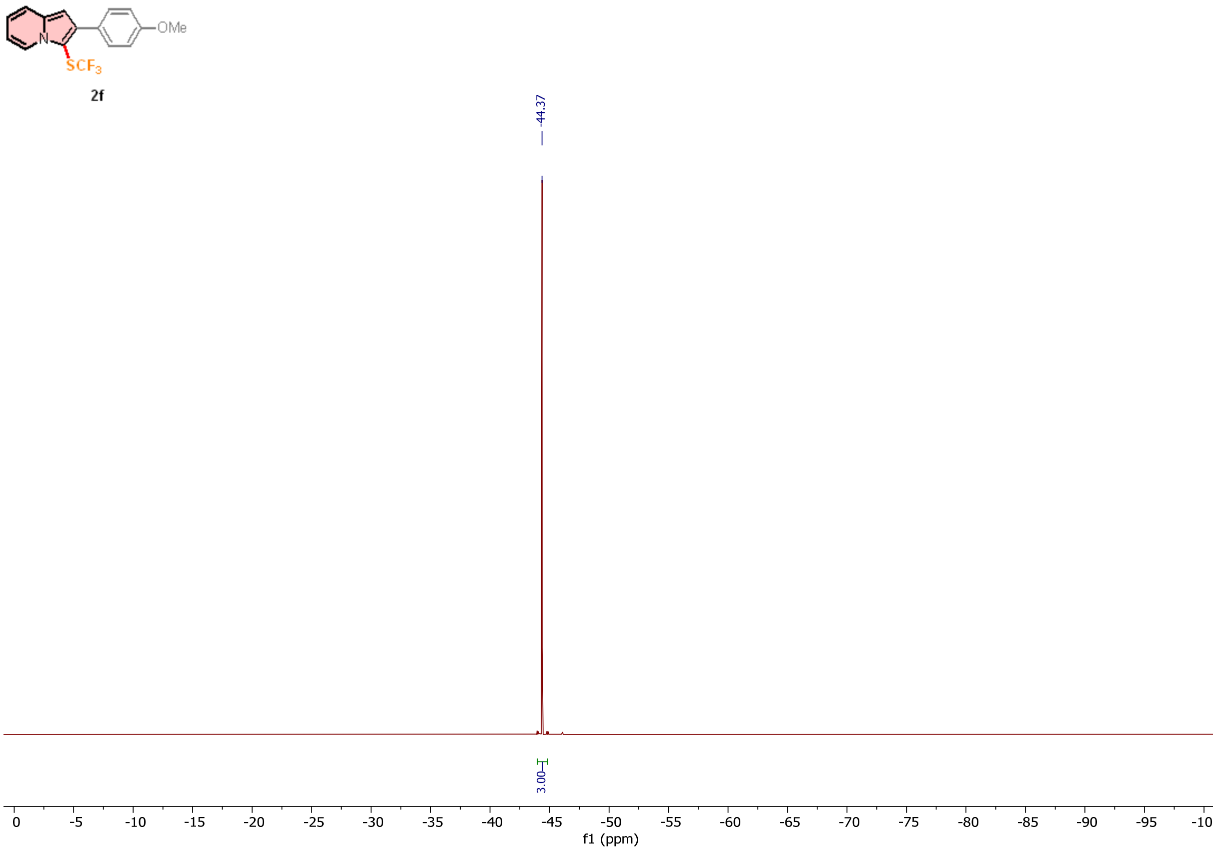

$^{19}F\{^{1}H\}$ NMR (377 MHz, $CDCl_3$) of **2f**.

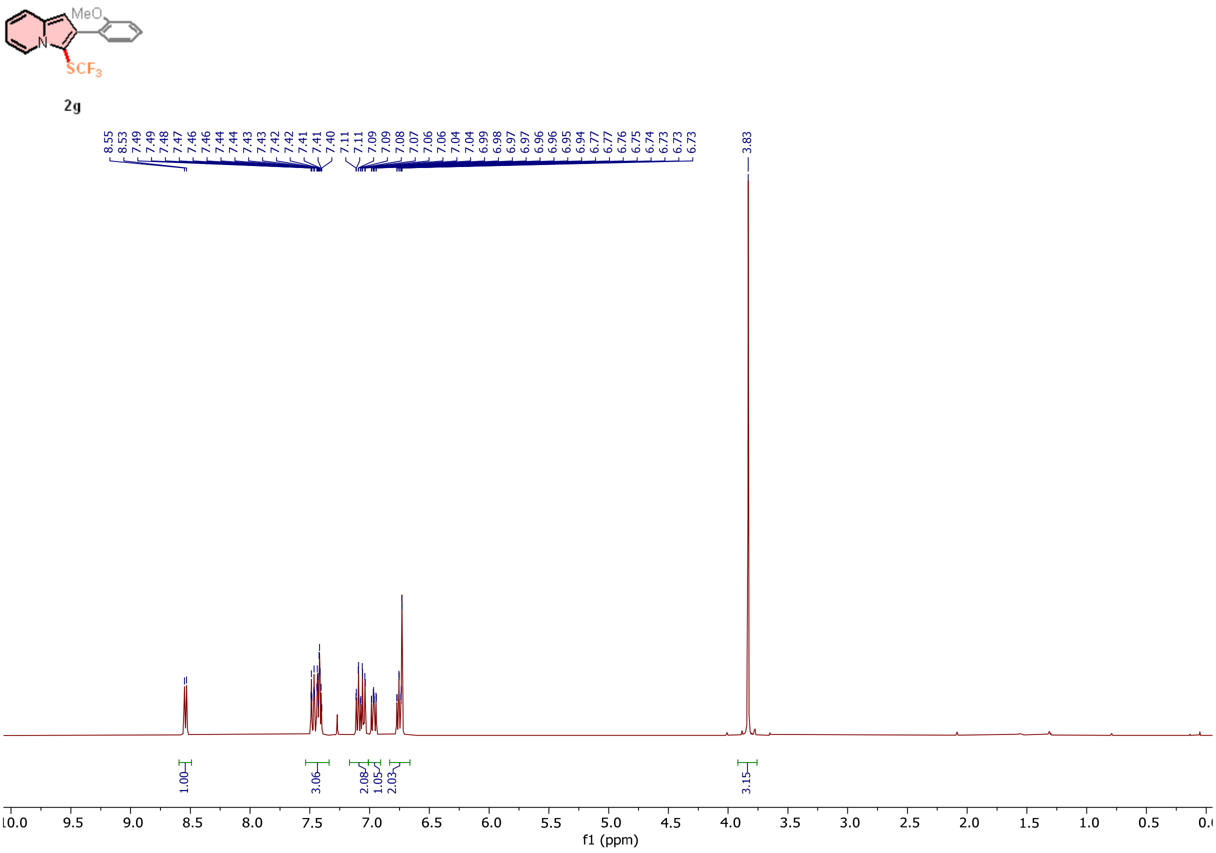

^{1}H NMR (400 MHz, $CDCl_3$) of **2g**.

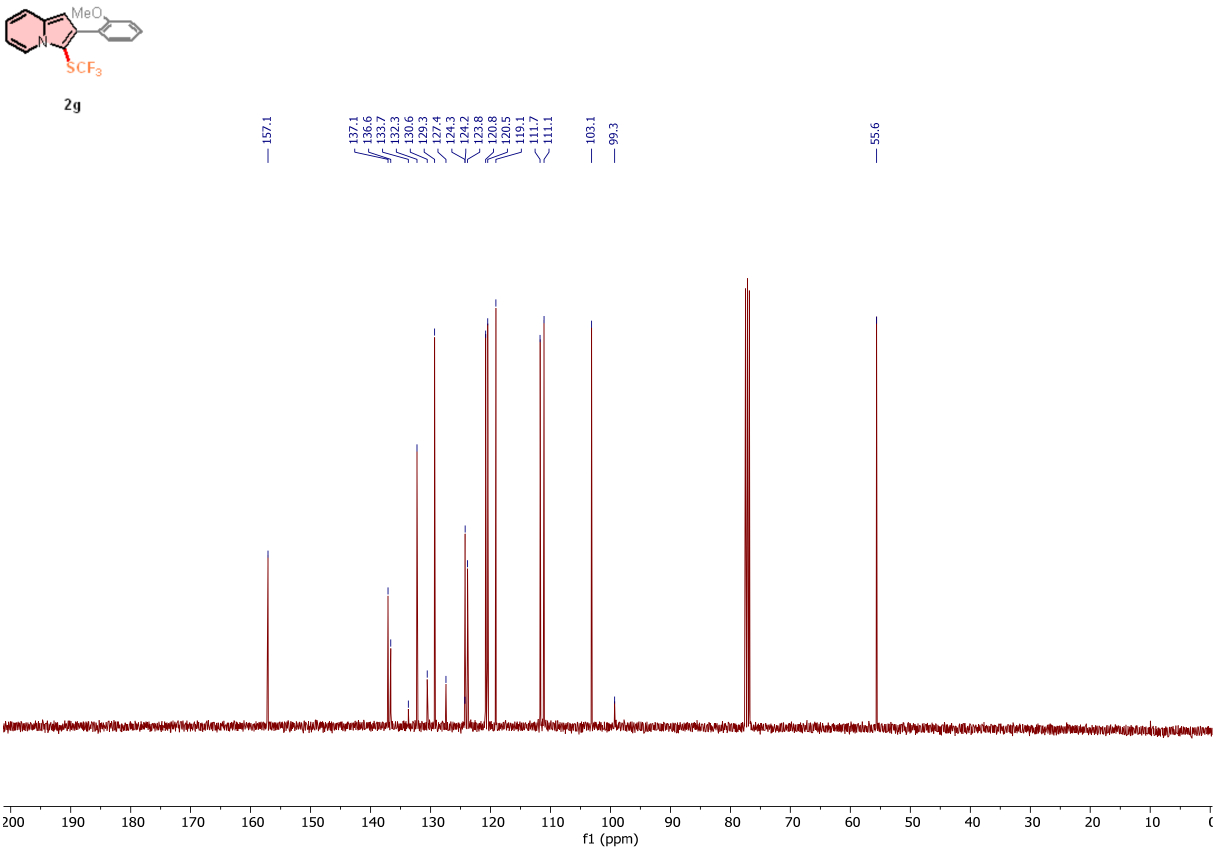

$^{13}C\{^{1}H\}$ NMR (100 MHz, $CDCl_3$) of **2g**.

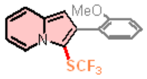

2g

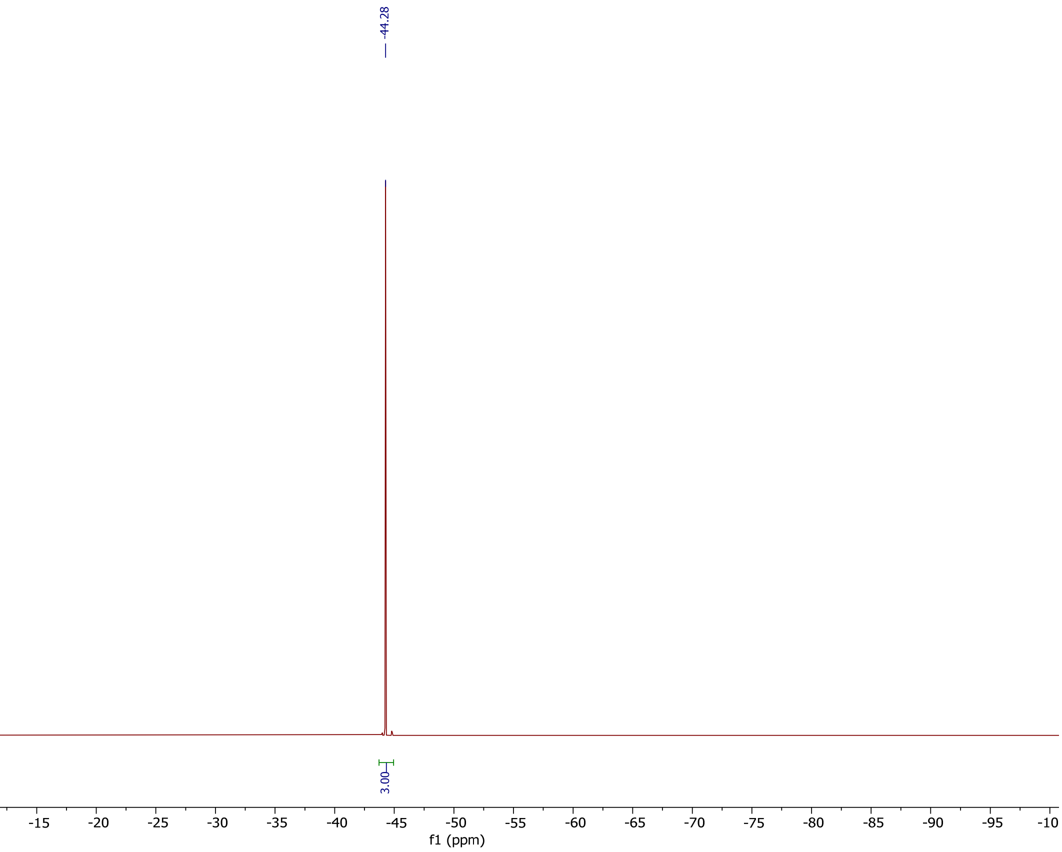

$^{19}F\{^{1}H\}$ NMR (377 MHz, $CDCl_3$) of **2g**.

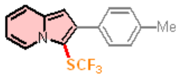

2h

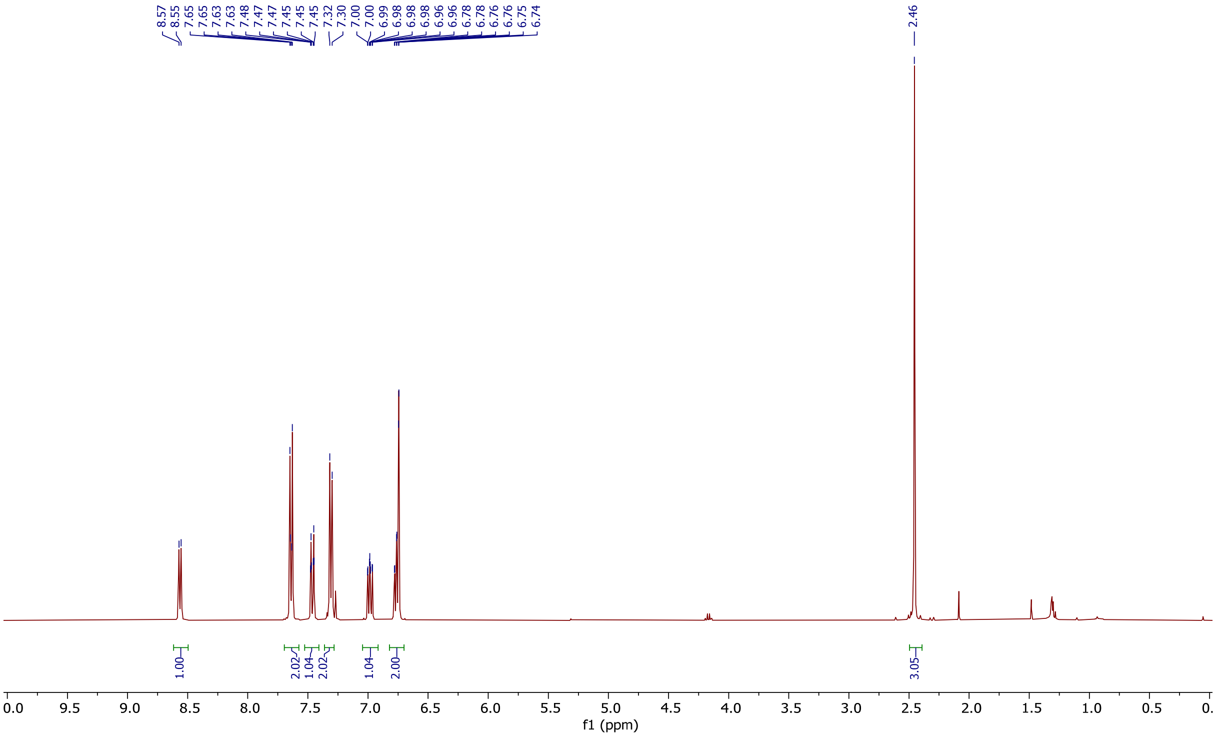

^{1}H NMR (400 MHz, $CDCl_3$) of **2h**.

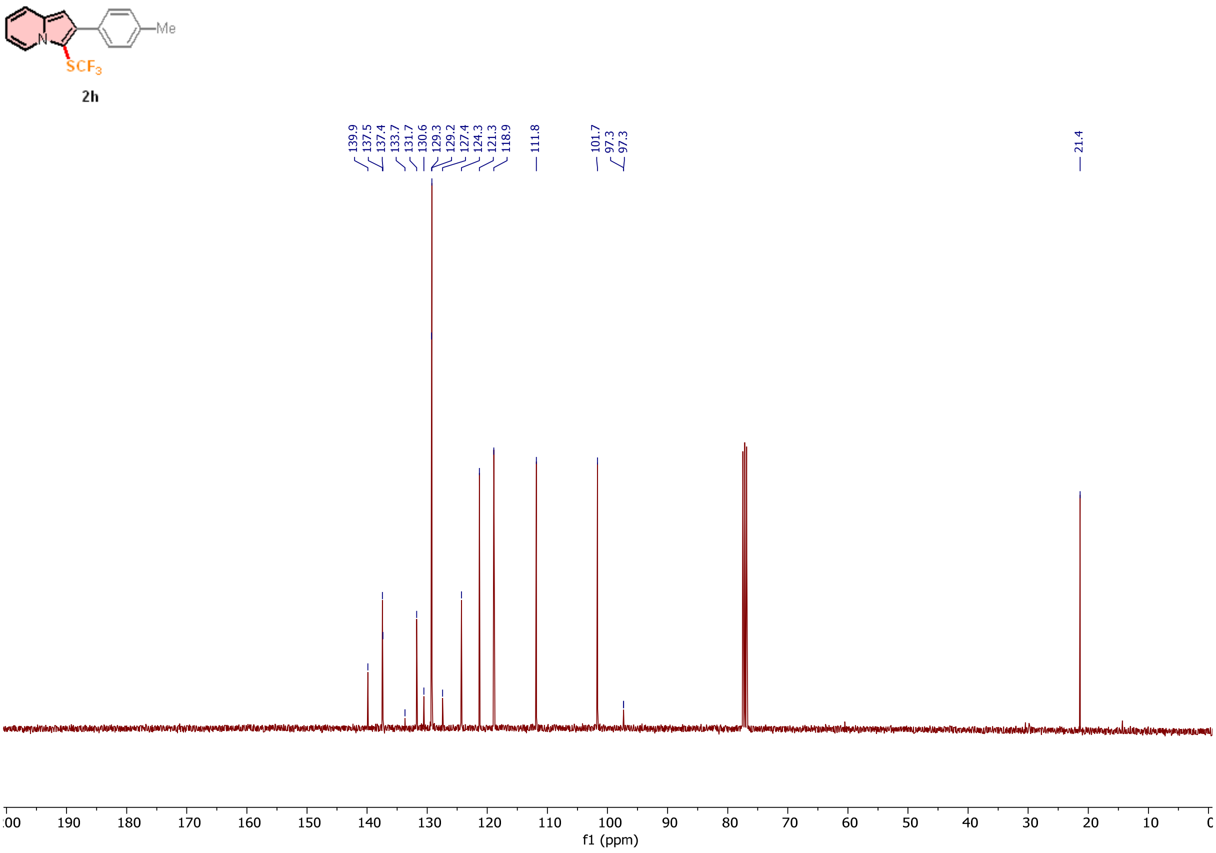

$^{13}C\{^{1}H\}$ NMR (100 MHz, $CDCl_3$) of **2h**.

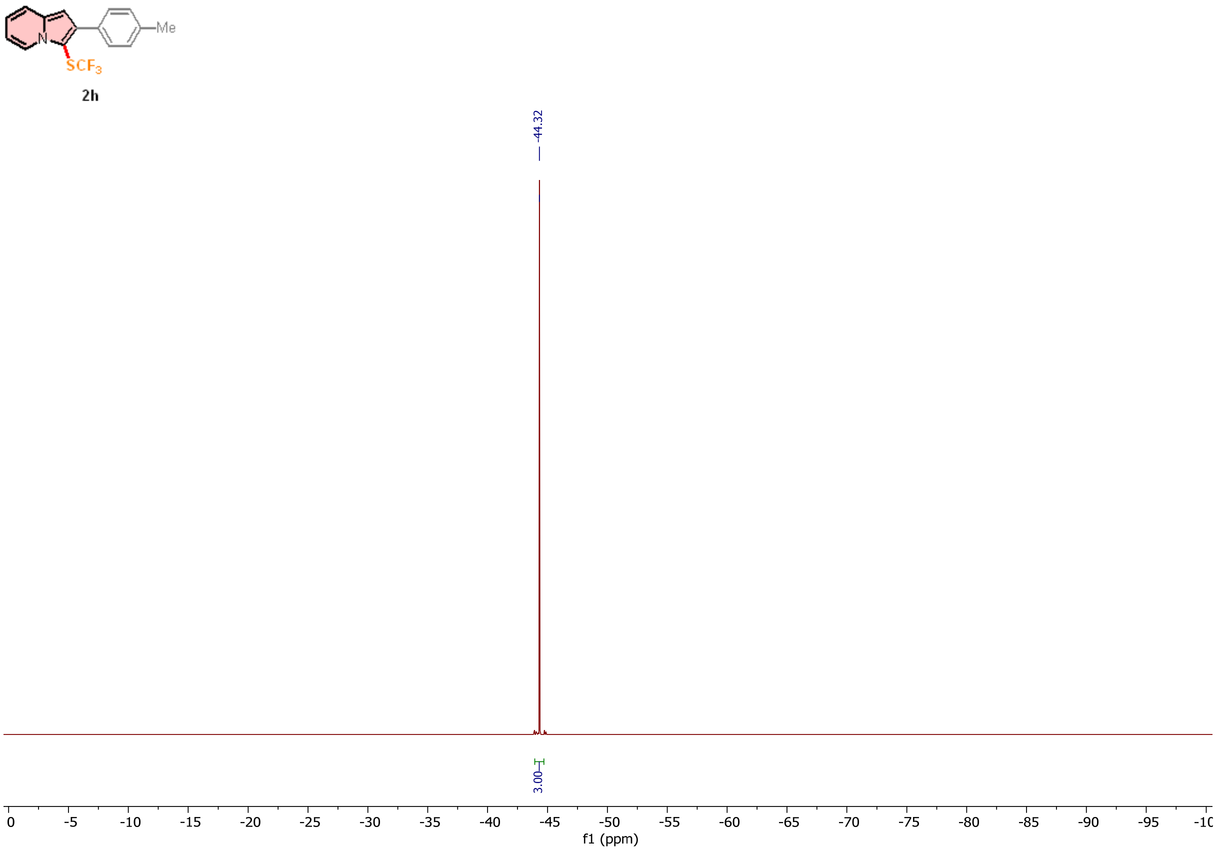

$^{19}F\{^{1}H\}$ NMR (377 MHz, $CDCl_3$) of **2h**.

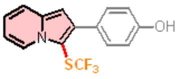

2i

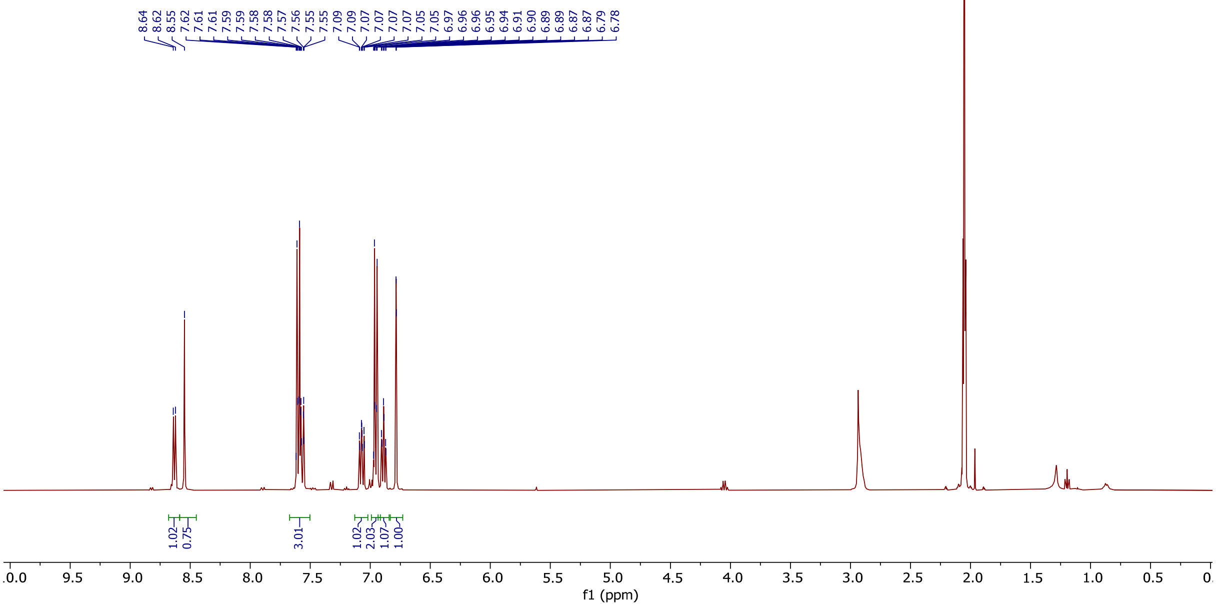

^{1}H NMR (400 MHz, Acetone-d^{6}) of **2i**.

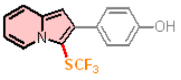

2i

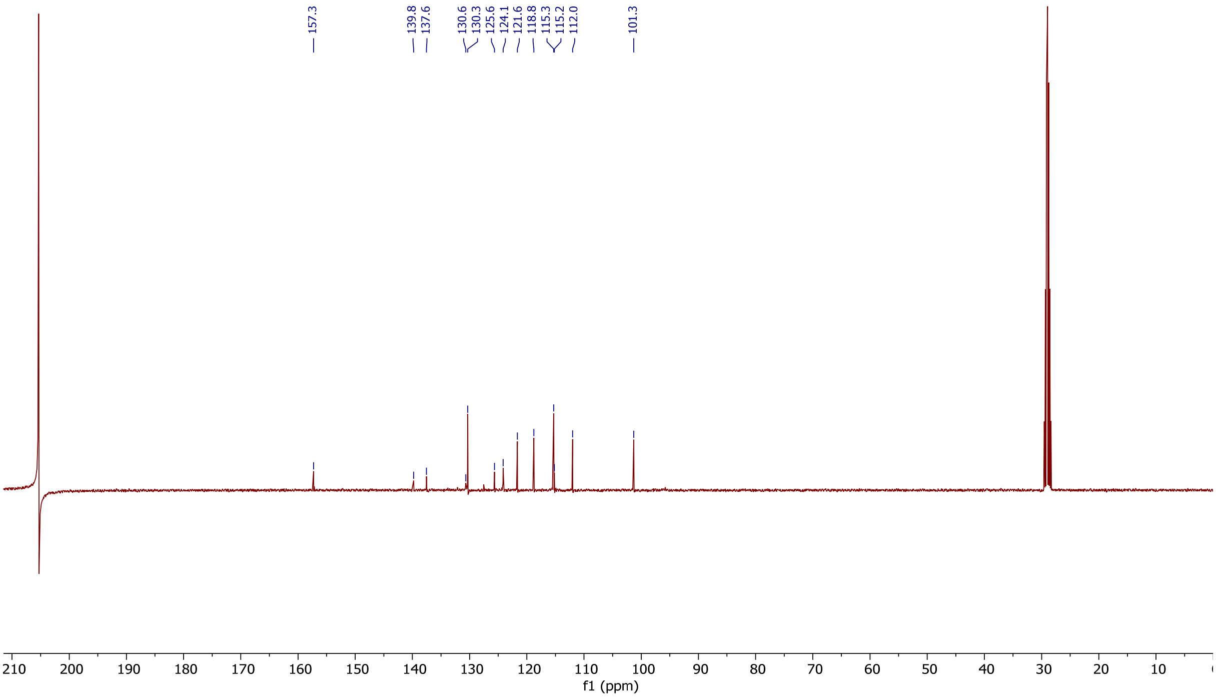

$^{13}C\{^{1}H\}$ NMR (100 MHz, Acetone-d^{6}) of **2i**.

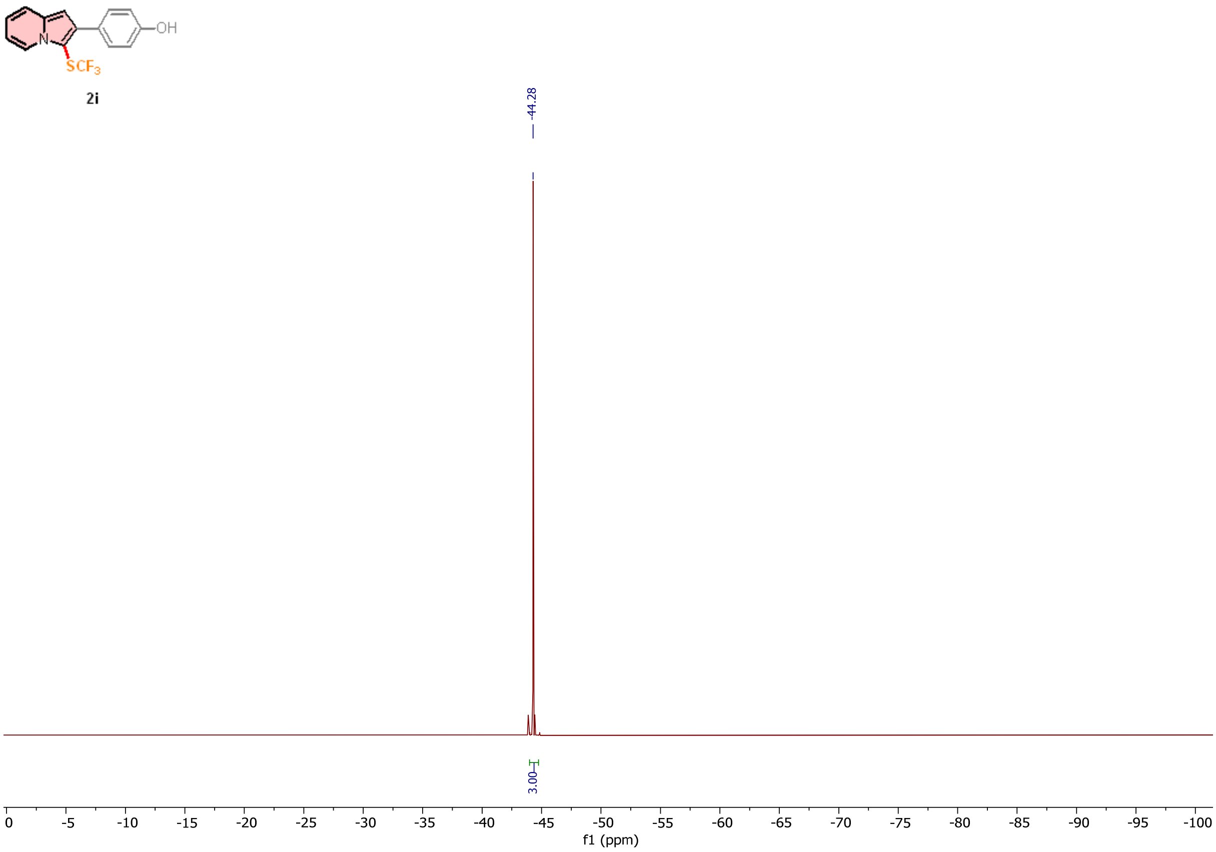

$^{19}F\{^{1}H\}$ NMR (377 MHz, Acetone-d[6]) of **2i**.

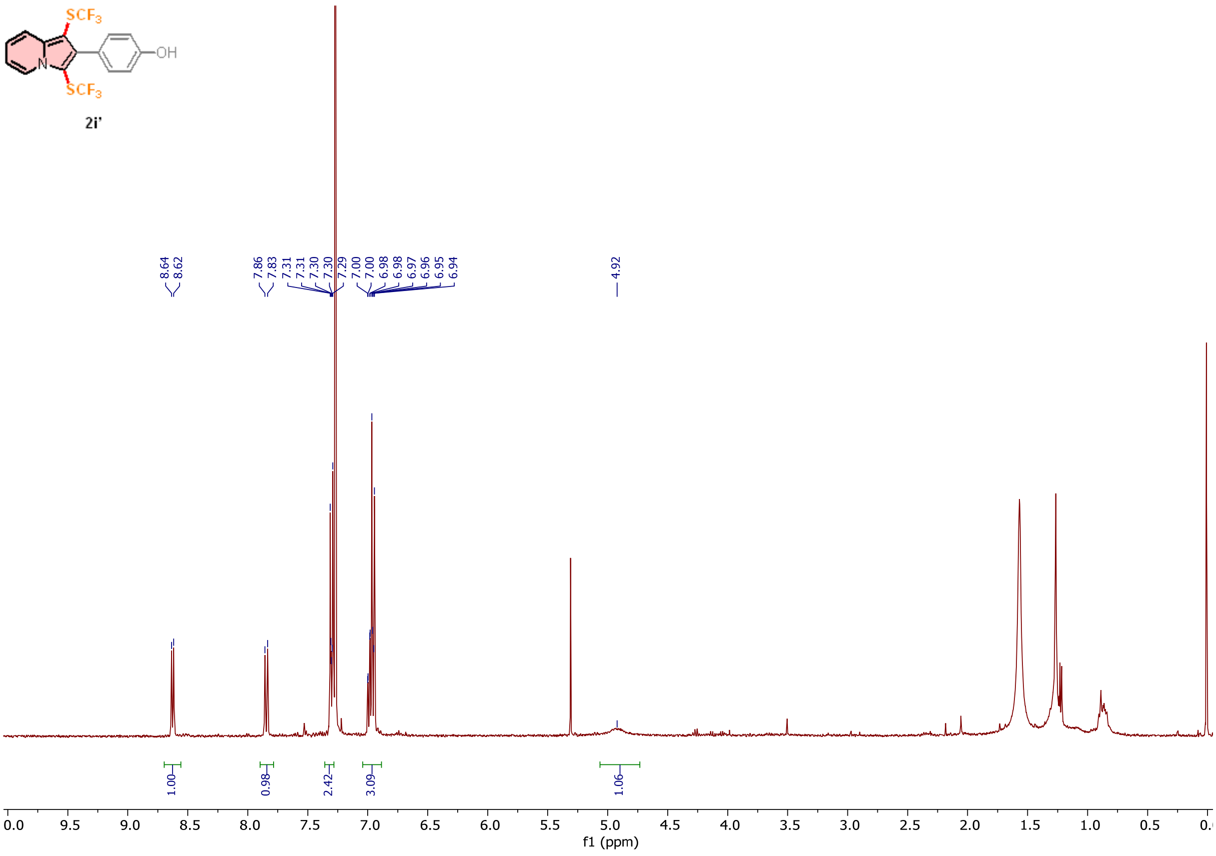

^{1}H NMR (400 MHz, $CDCl_3$) of **2i’**.

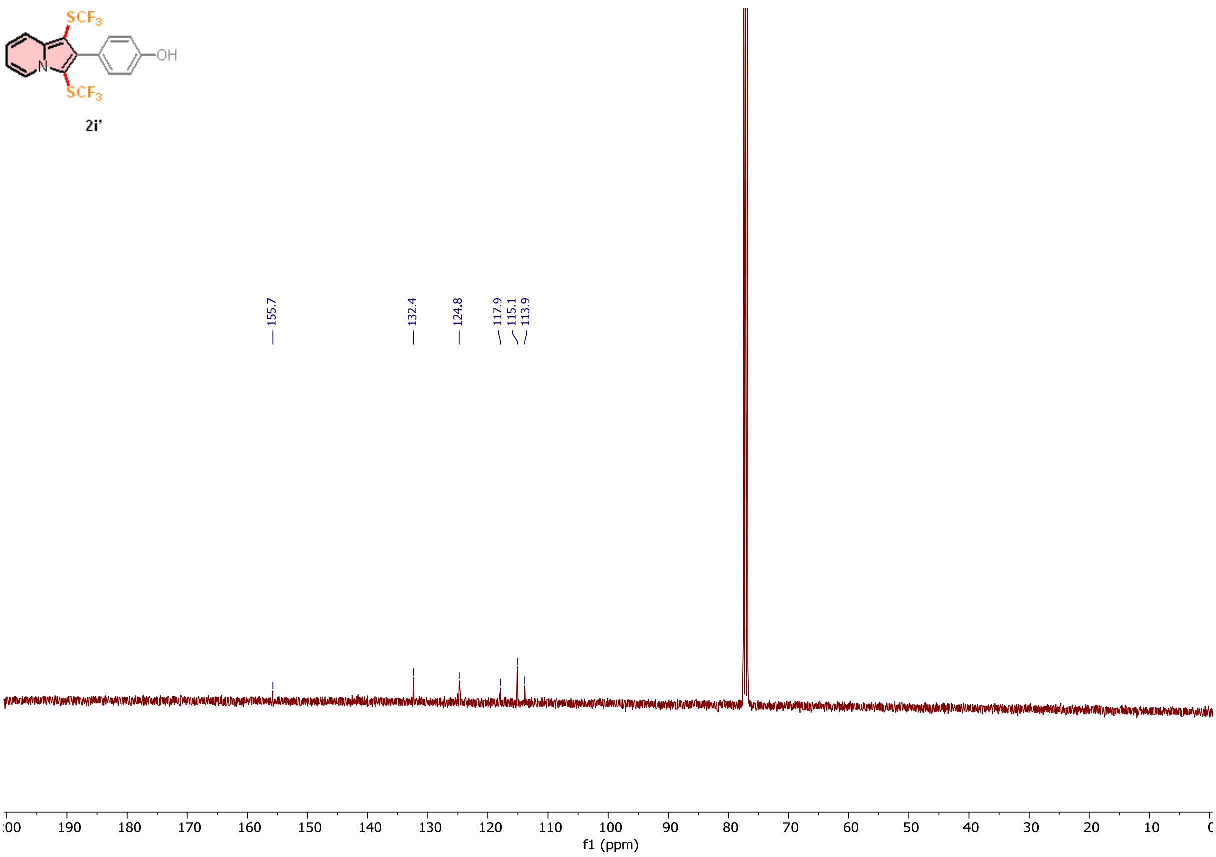

$^{13}C\{^{1}H\}$ NMR (100 MHz, $CDCl_3$) of **2i'**.

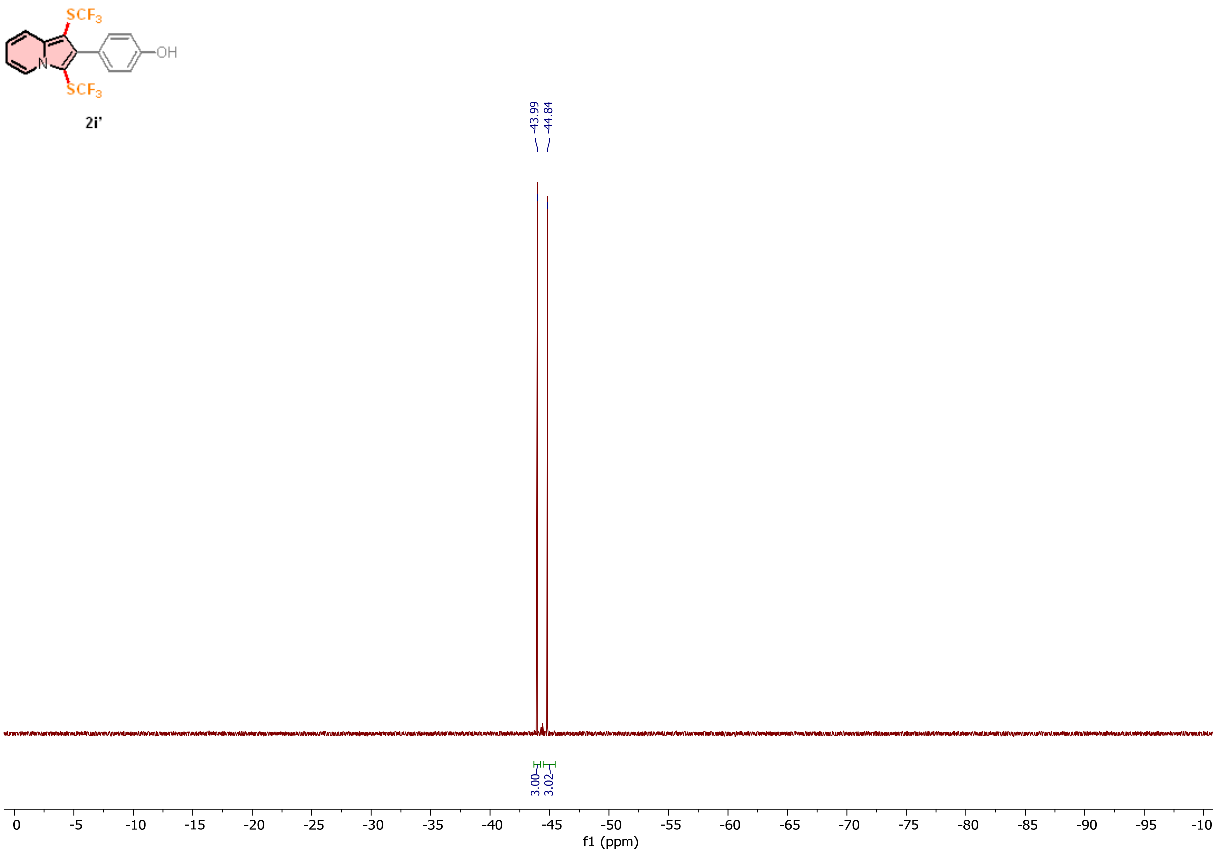

$^{19}F\{^{1}H\}$ NMR (377 MHz, $CDCl_3$) of **2i'**.

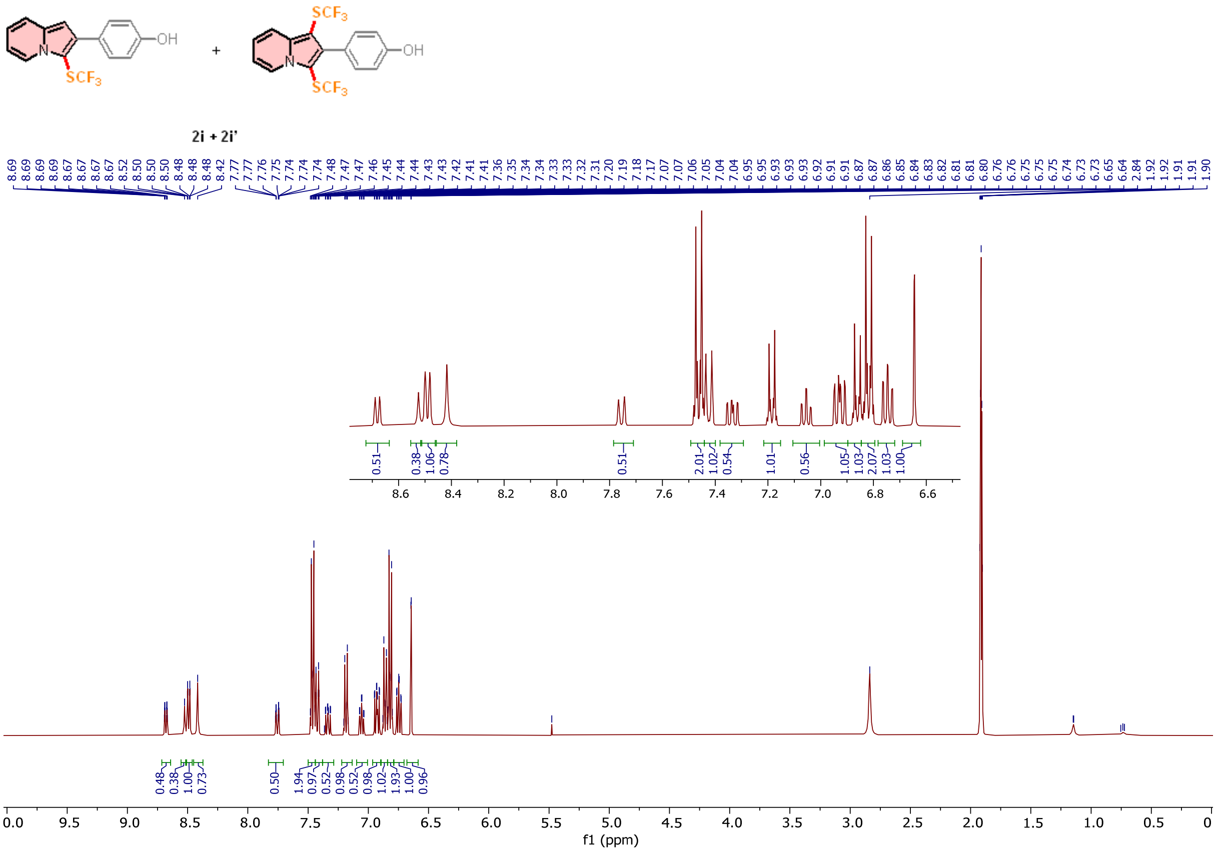

^{1}H NMR (400 MHz, Acetone-d^{6}) of **2i + 2i'**.

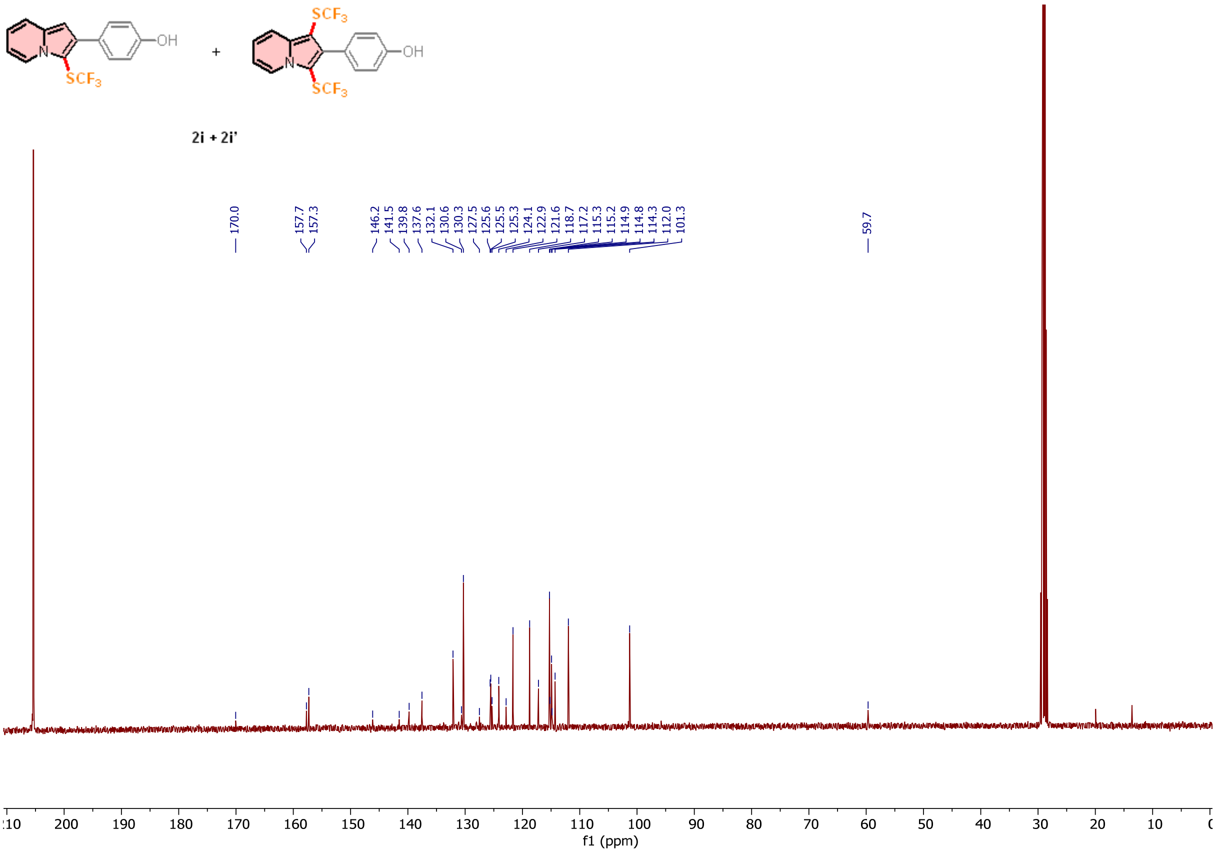

^{13}C{^{1}H} NMR (100 MHz, Acetone-d^{6}) of **2i + 2i'**.

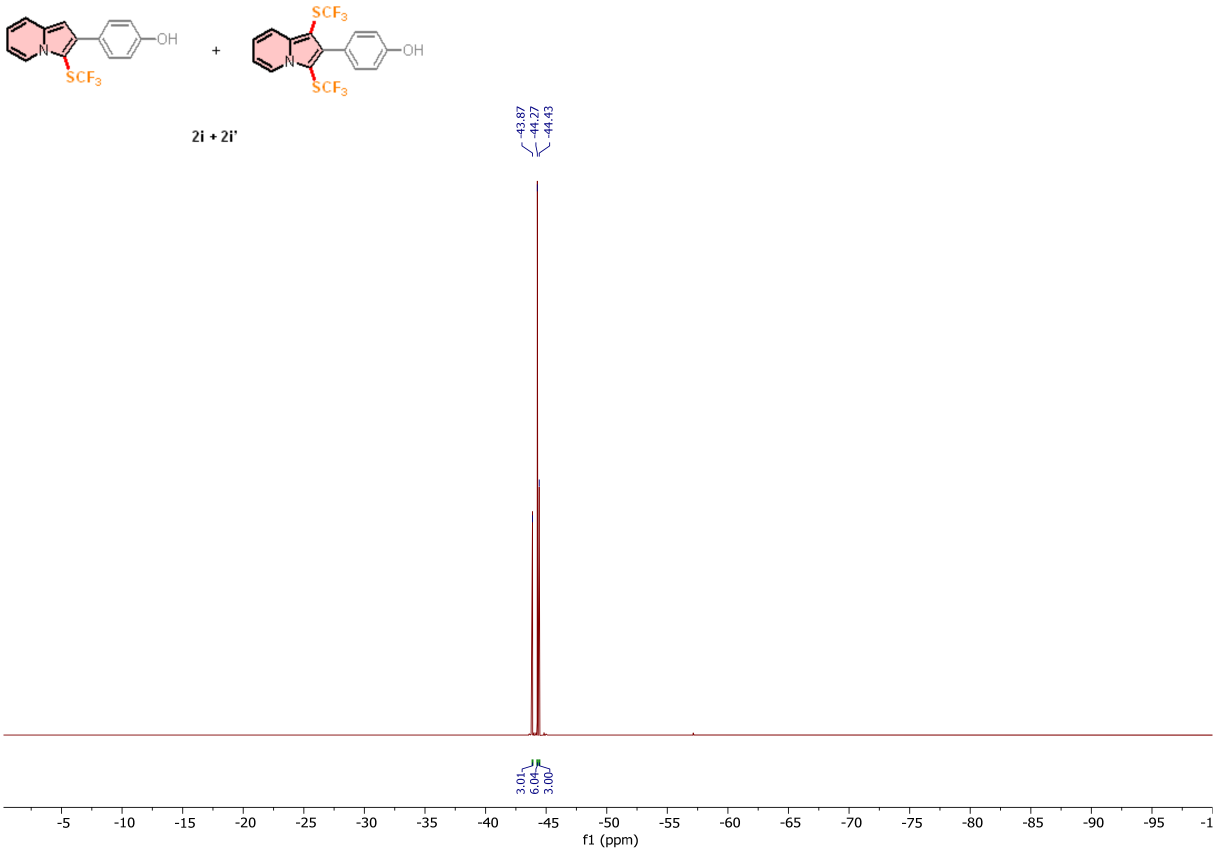

$^{19}F\{^1H\}$ NMR (377 MHz, Acetone-d[6]) of **2i + 2i'**.

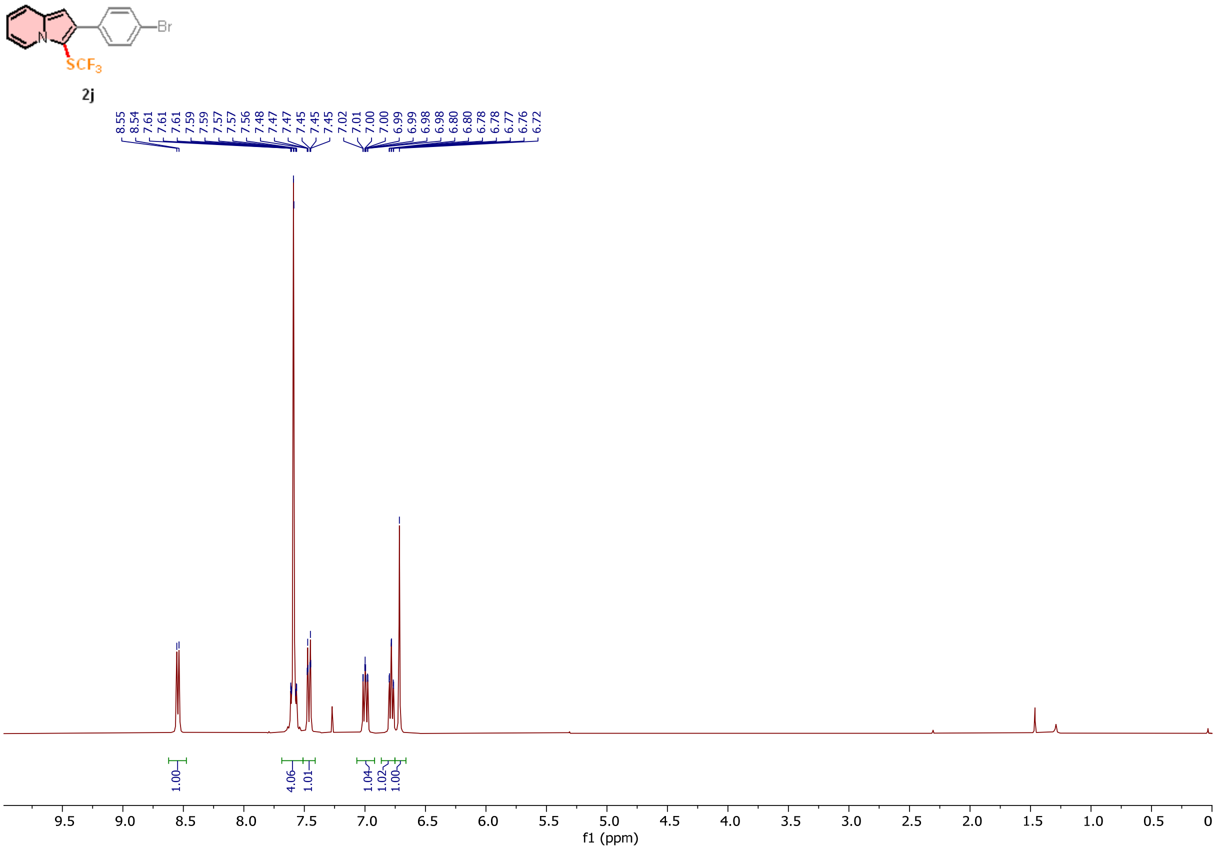

^1H NMR (400 MHz, $CDCl_3$) of **2j**.

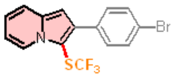

2j

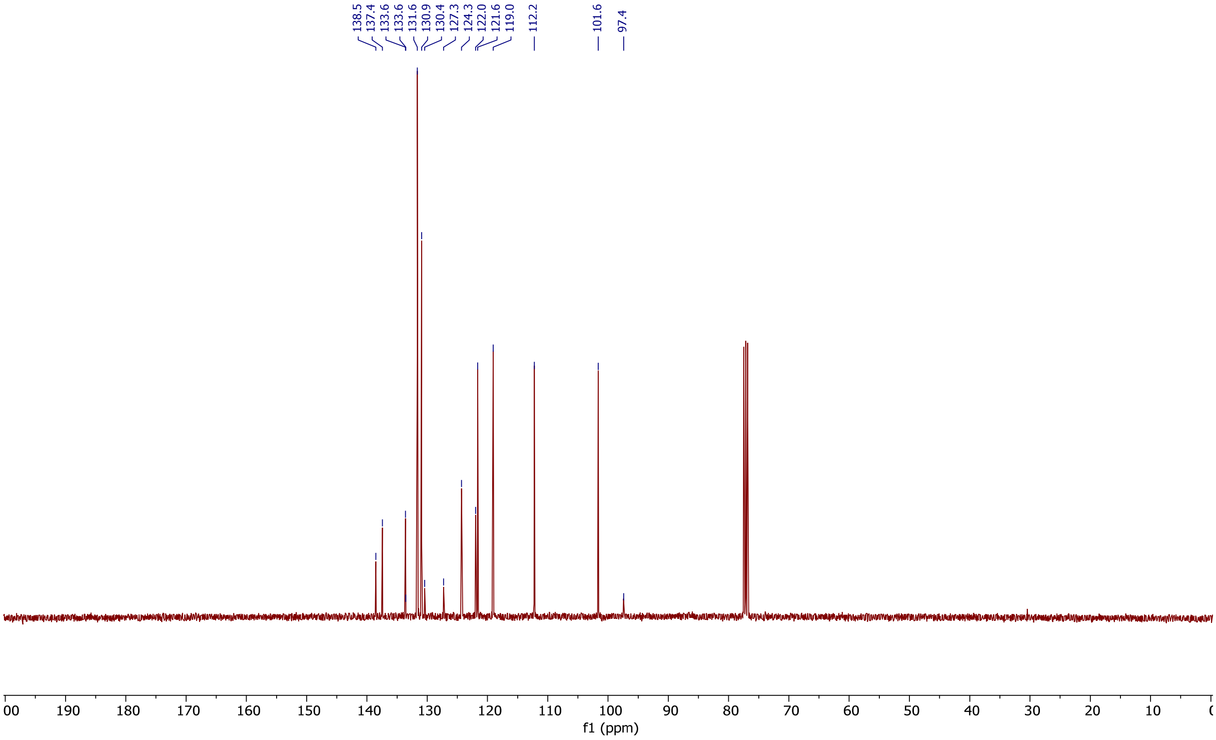

$^{13}C\{^1H\}$ NMR (100 MHz, $CDCl_3$) of **2j**.

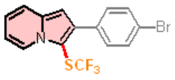

2j

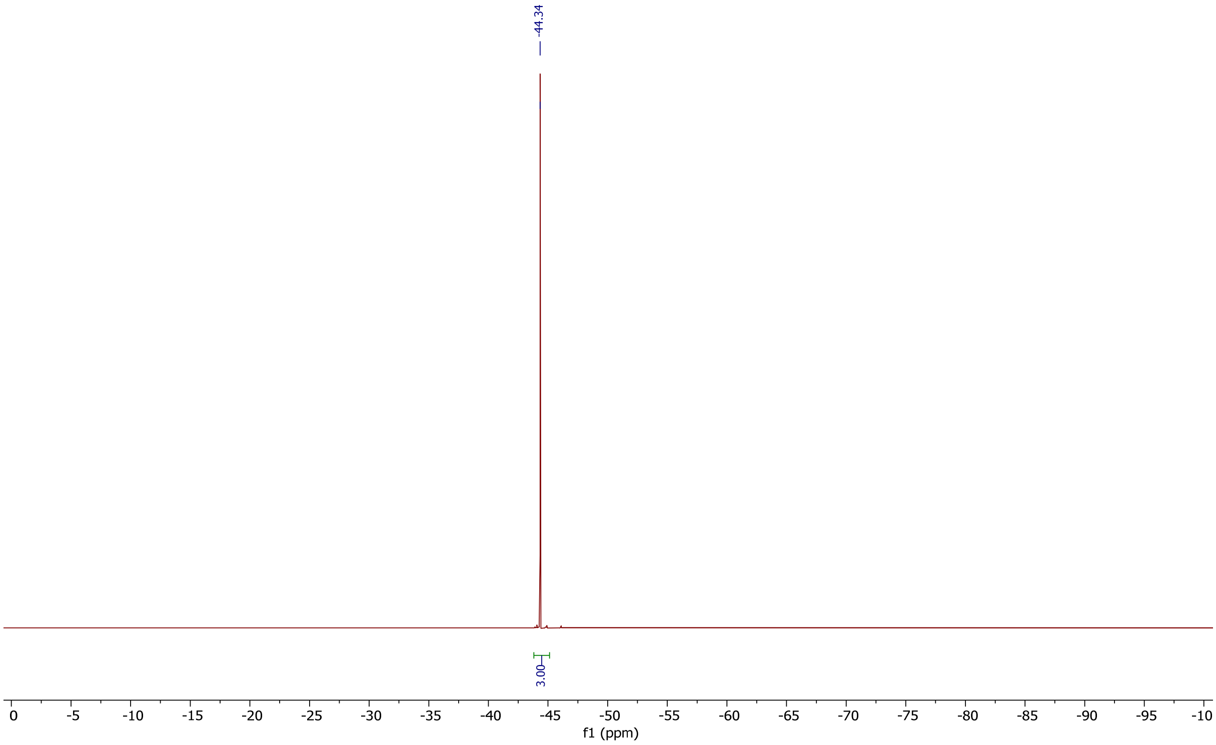

$^{19}F\{^1H\}$ NMR (377 MHz, $CDCl_3$) of **2j**.

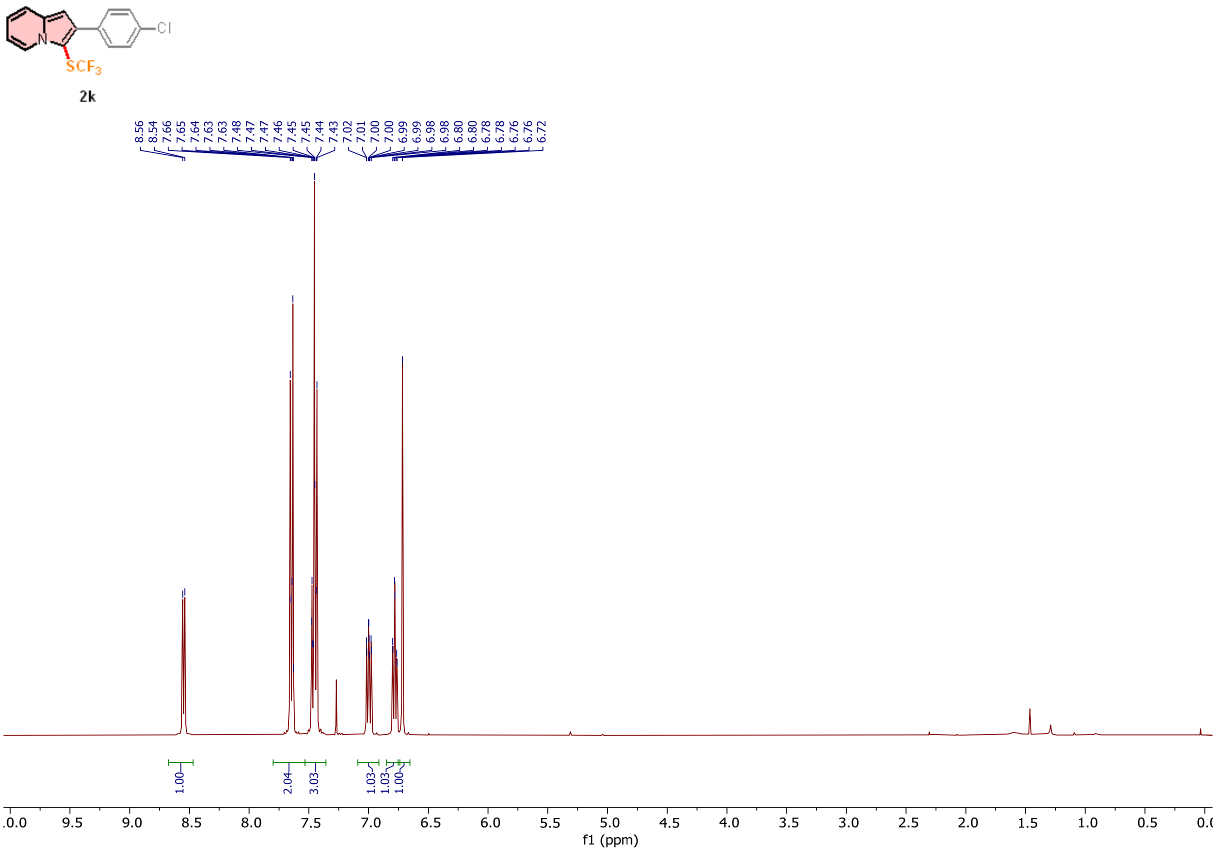

^1H NMR (400 MHz, $CDCl_3$) of **2k**.

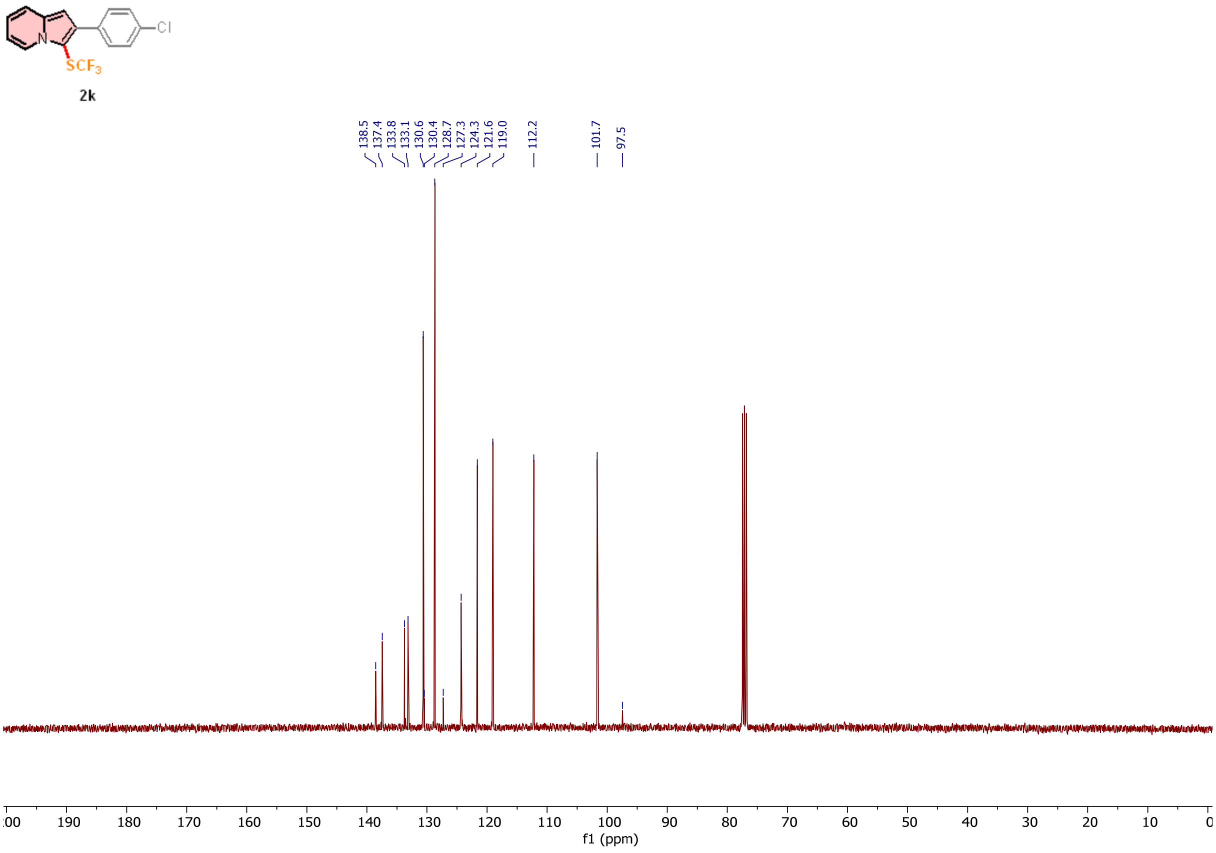

$^{13}C\{^1H\}$ NMR (100 MHz, $CDCl_3$) of **2k**.

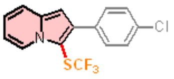

2k

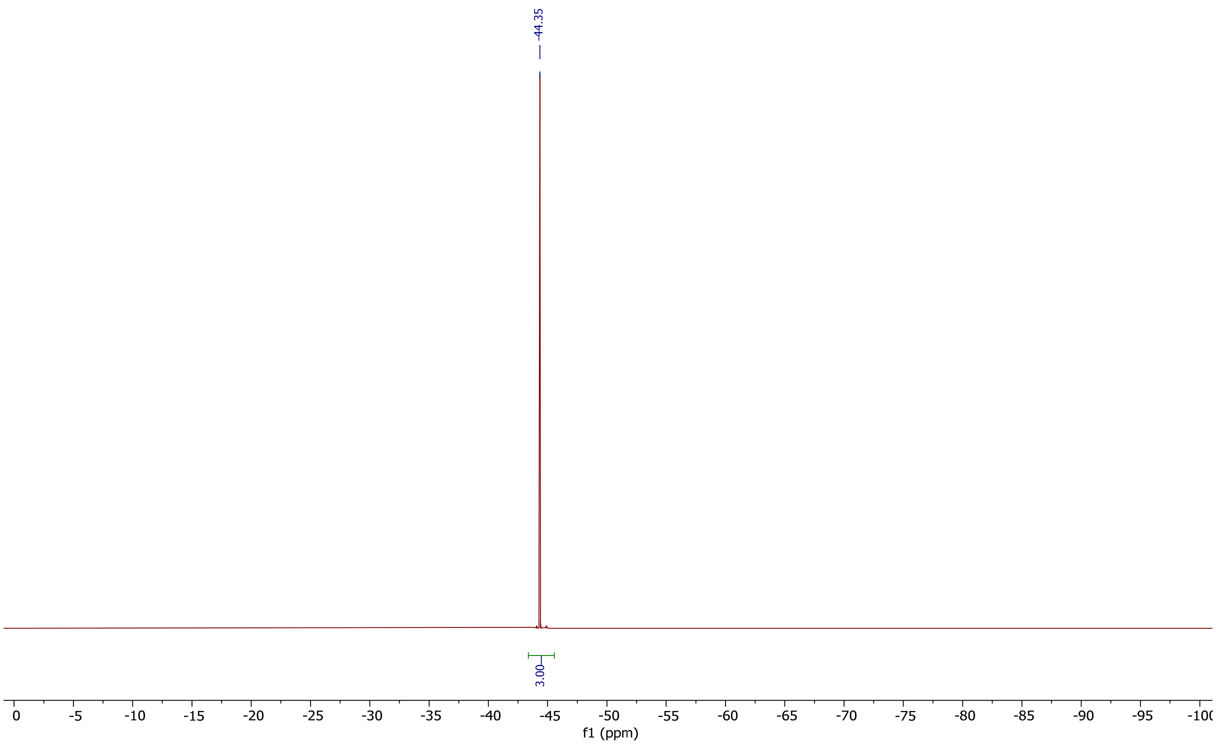

$^{19}F\{^{1}H\}$ NMR (377 MHz, $CDCl_3$) of **2k**.

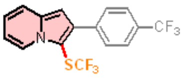

2l

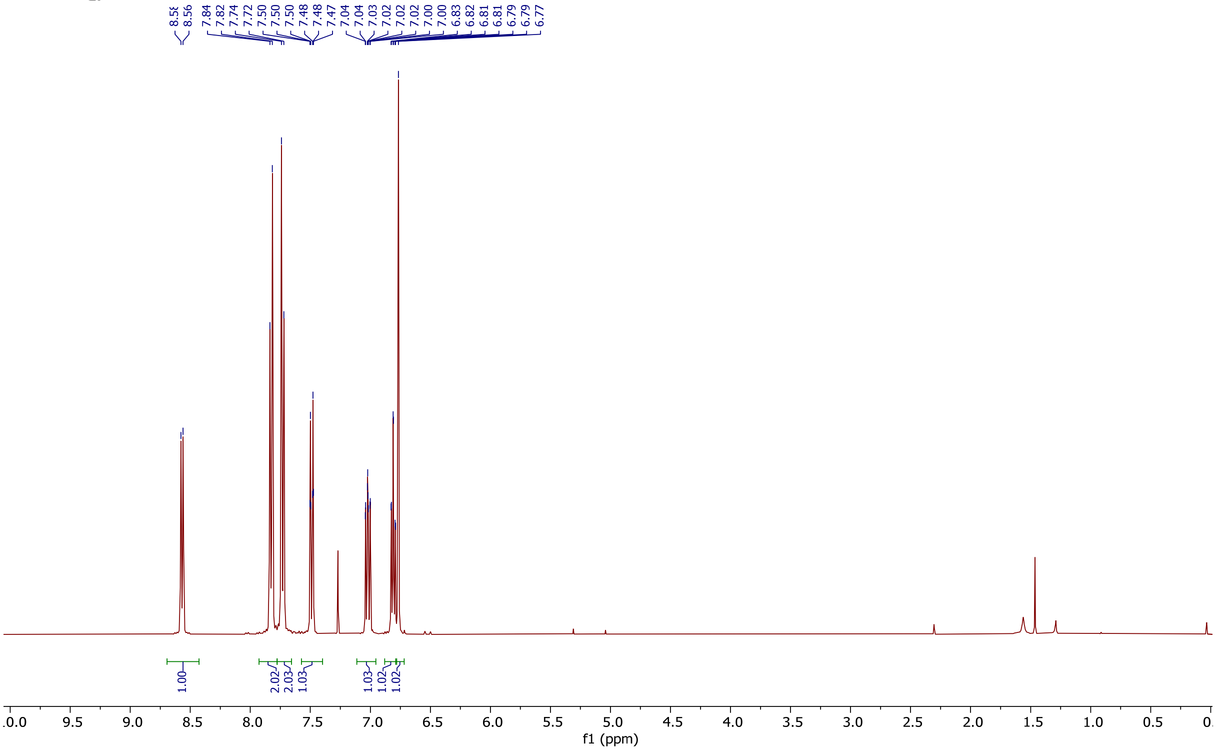

^{1}H NMR (400 MHz, $CDCl_3$) of **2l**.

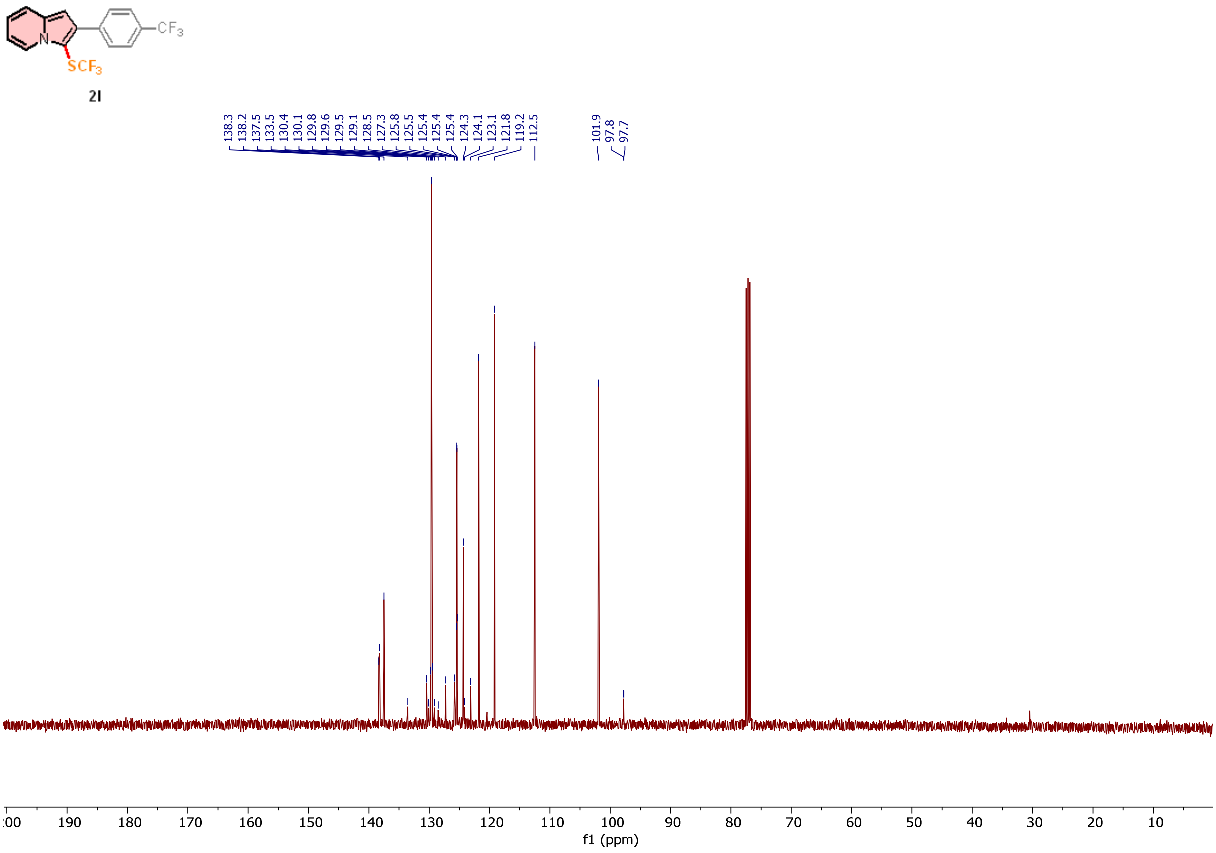

$^{13}C\{^{1}H\}$ NMR (100 MHz, $CDCl_3$) of **2l**.

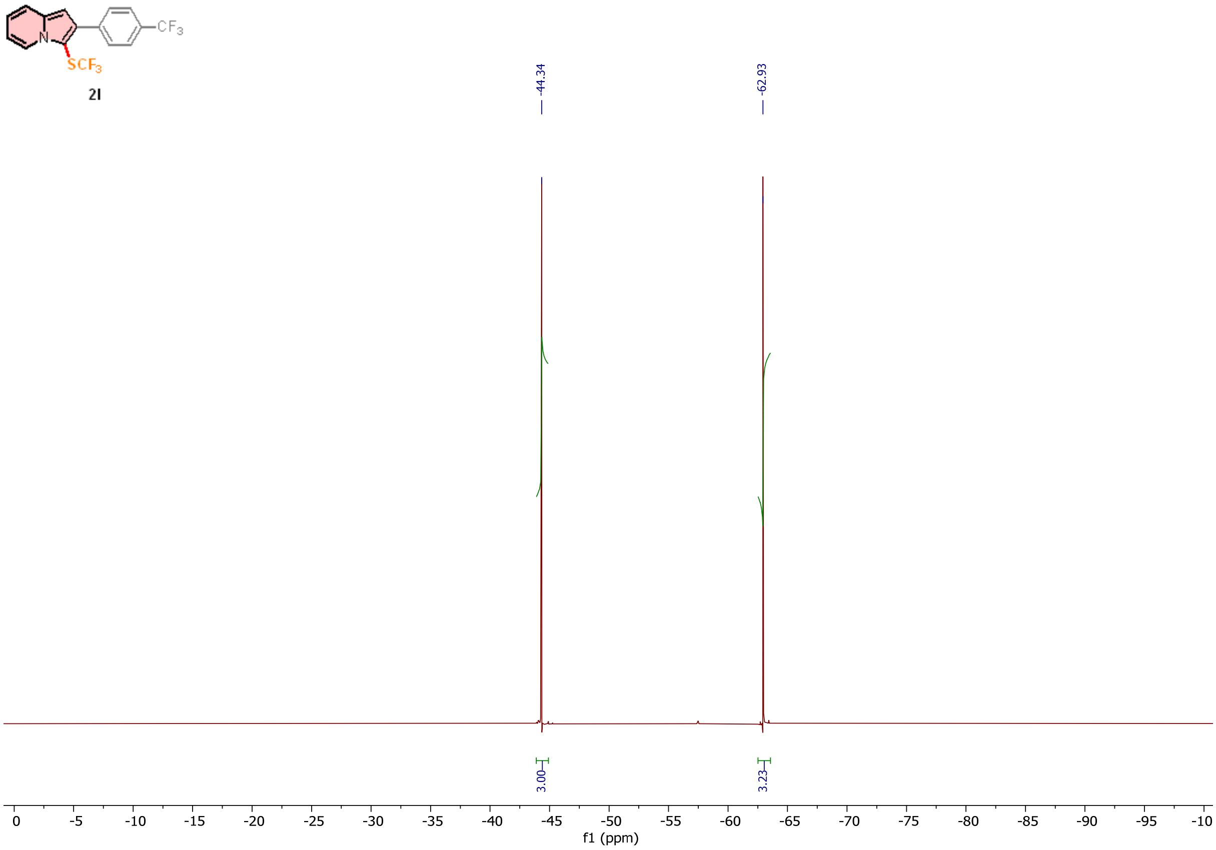

$^{19}F\{^{1}H\}$ NMR (377 MHz, $CDCl_3$) of **2l**.

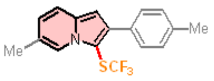

2m

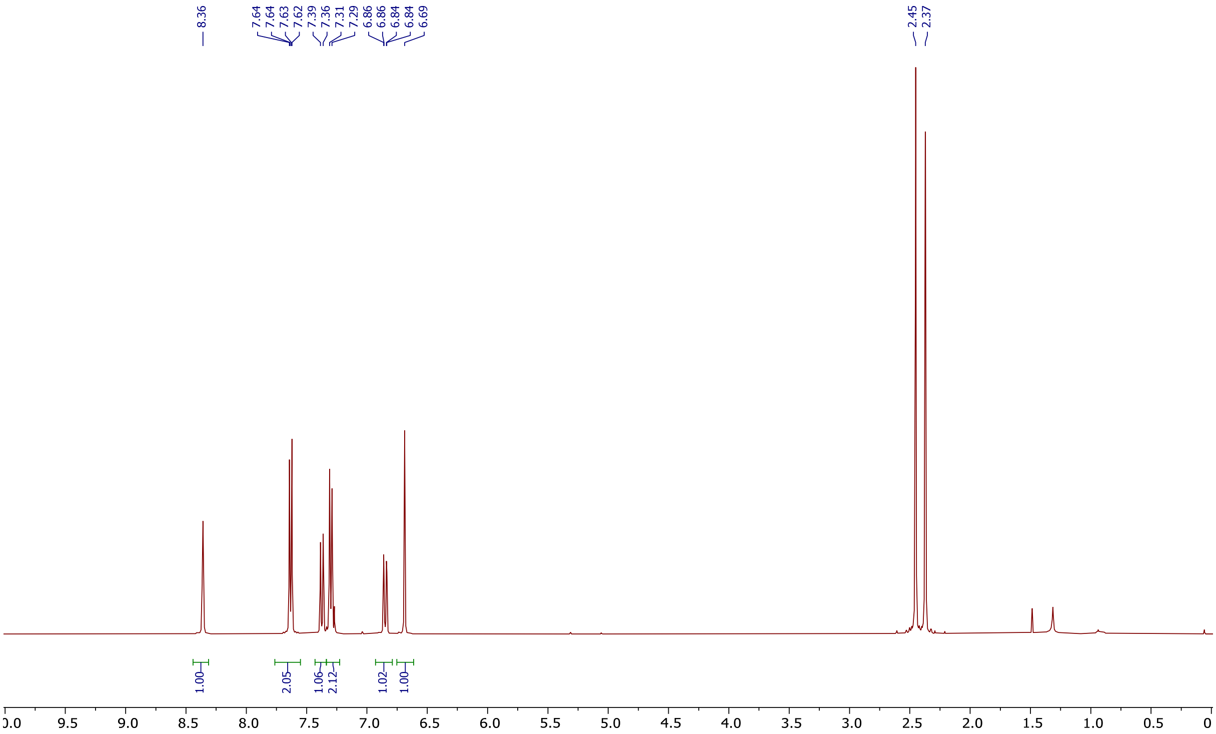

^{1}H NMR (400 MHz, $CDCl_3$) of **2m**.

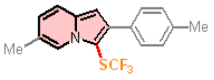

2m

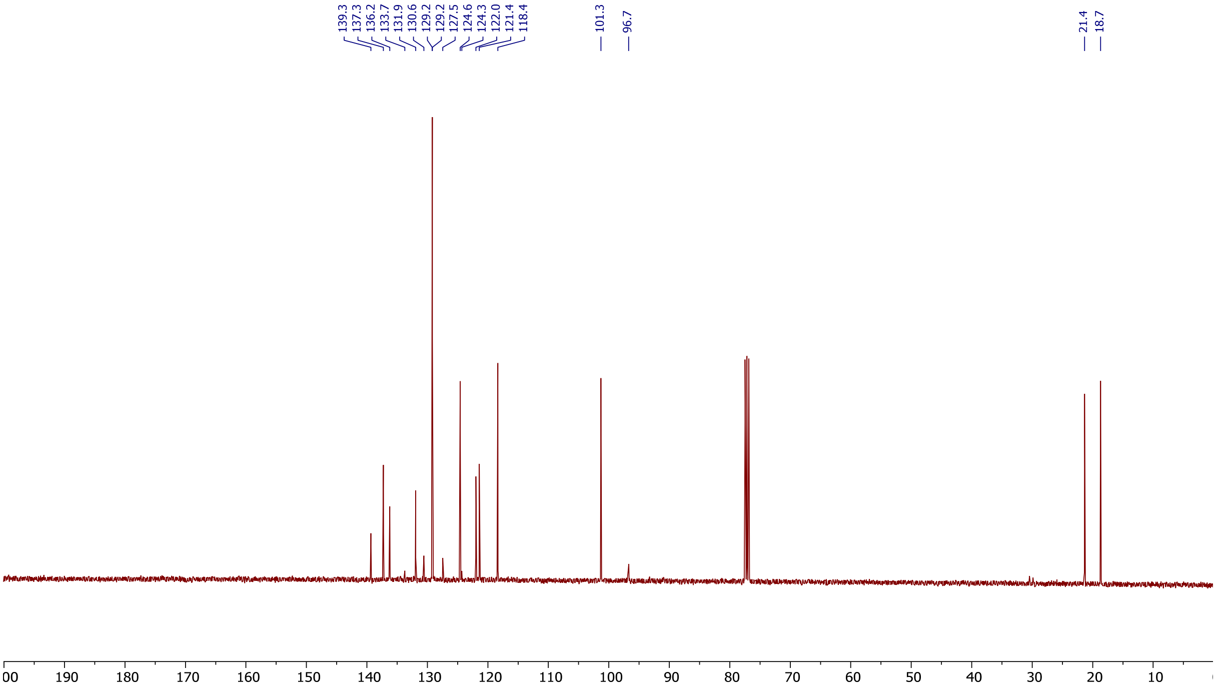

$^{13}C\{^1H\}$ NMR (100 MHz, $CDCl_3$) of **2m**.

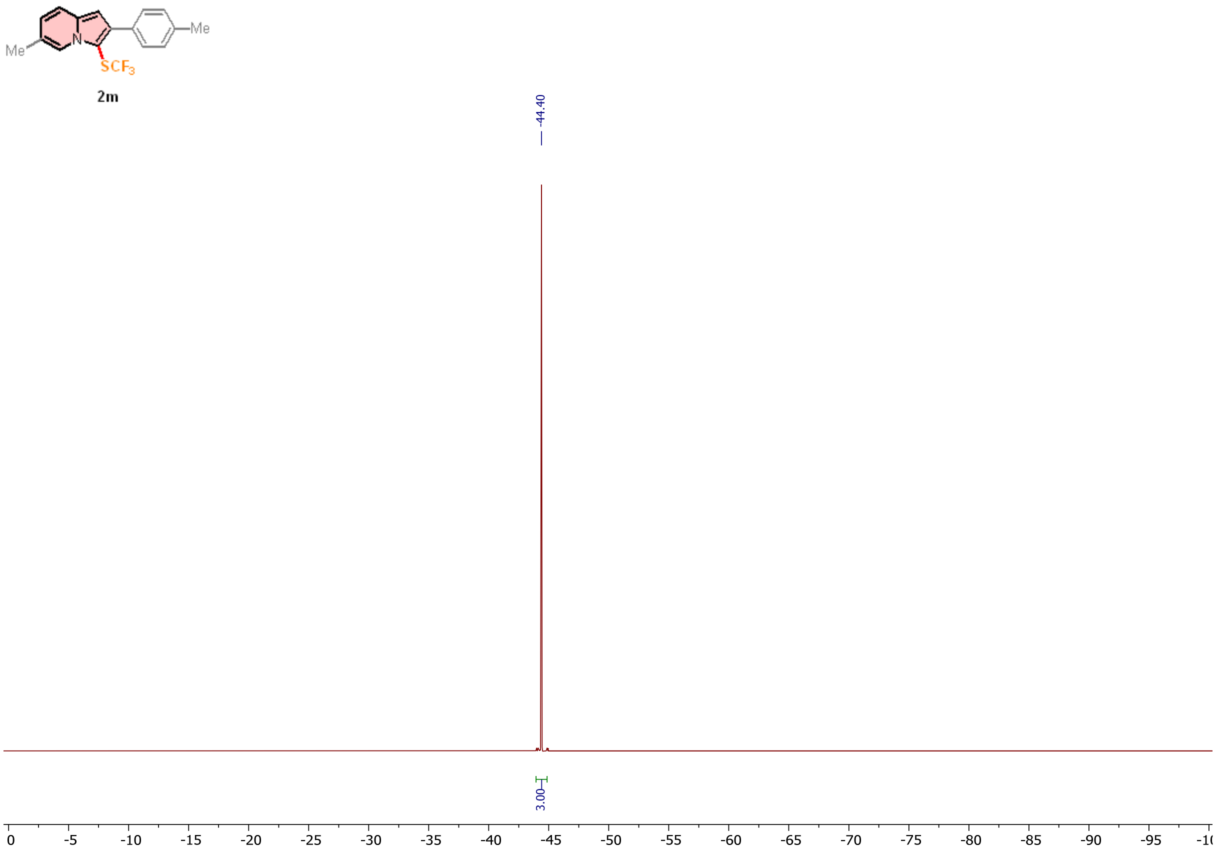

$^{19}F\{^{1}H\}$ NMR (377 MHz, $CDCl_3$) of **2m**.

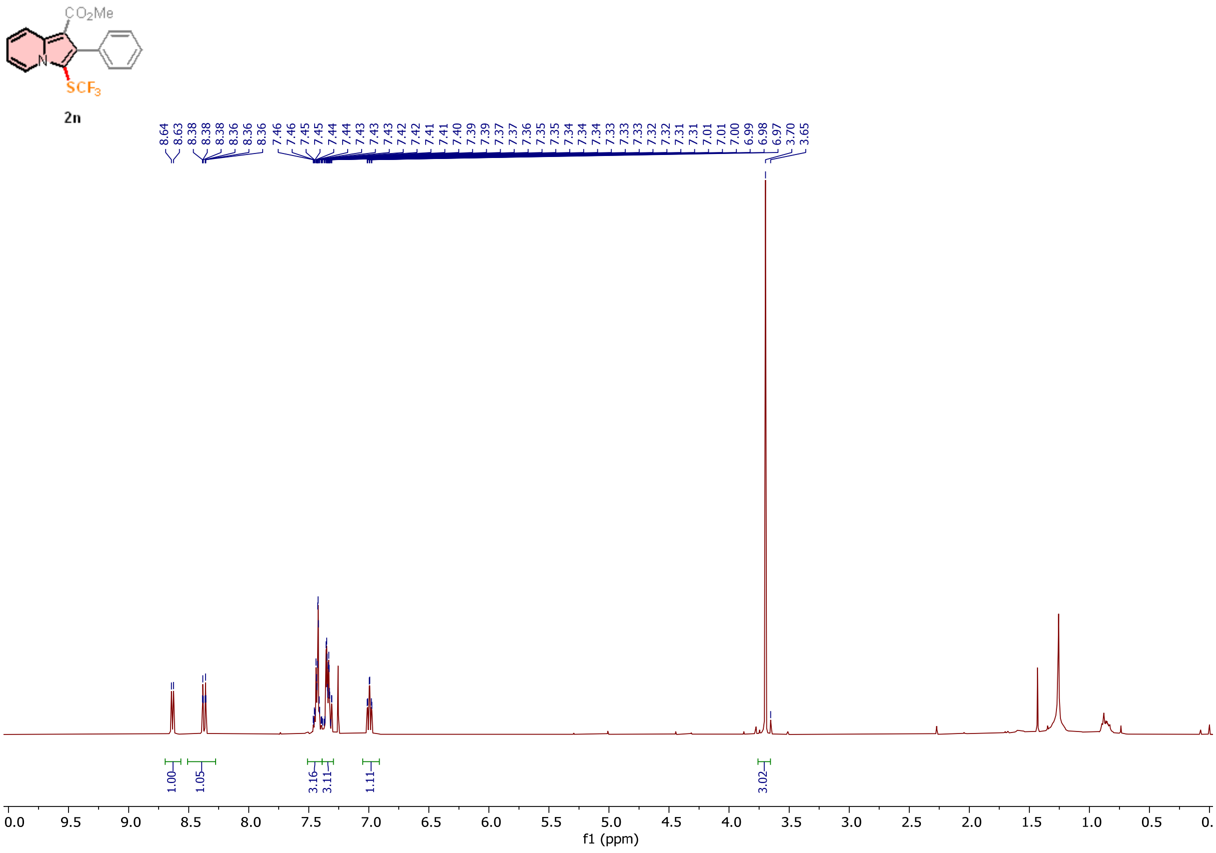

^{1}H NMR (400 MHz, $CDCl_3$) of **2n**.

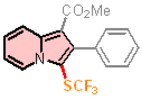

2n

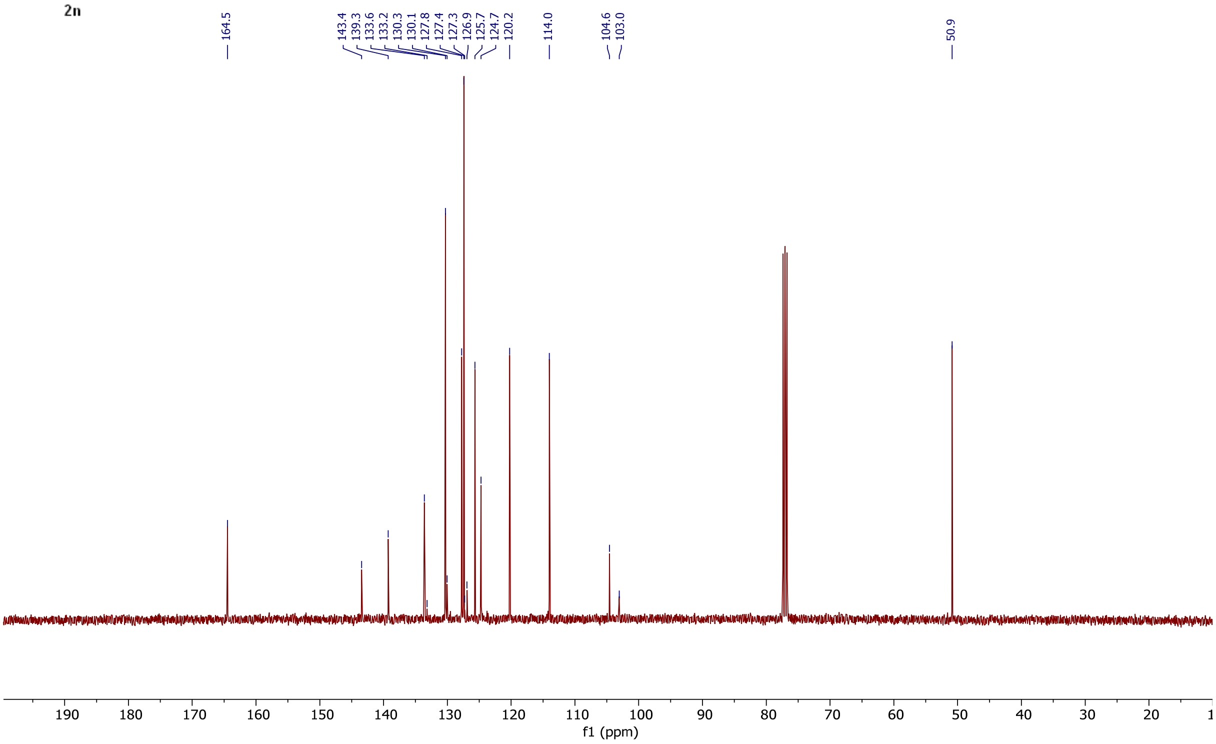

$^{13}C\{^1H\}$ NMR (100 MHz, $CDCl_3$) of **2n**.

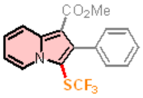

2n

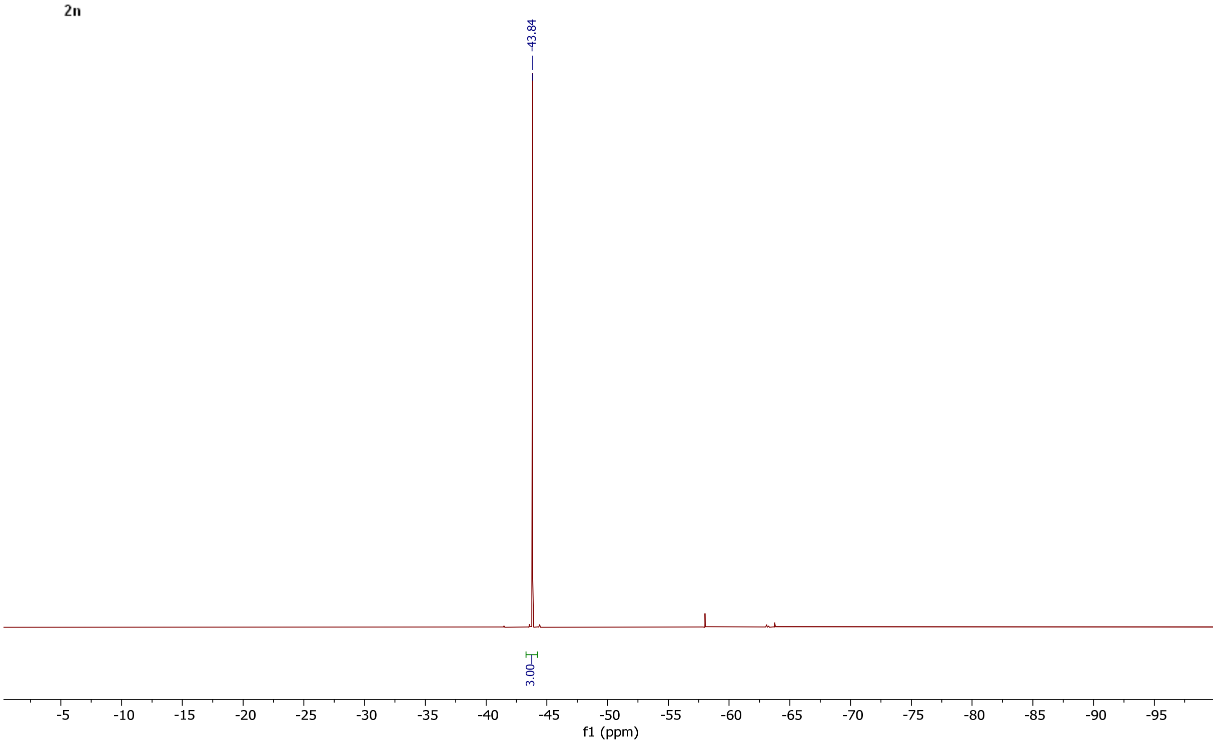

$^{19}F\{^1H\}$ NMR (377 MHz, $CDCl_3$) of **2n**.

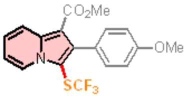

2o

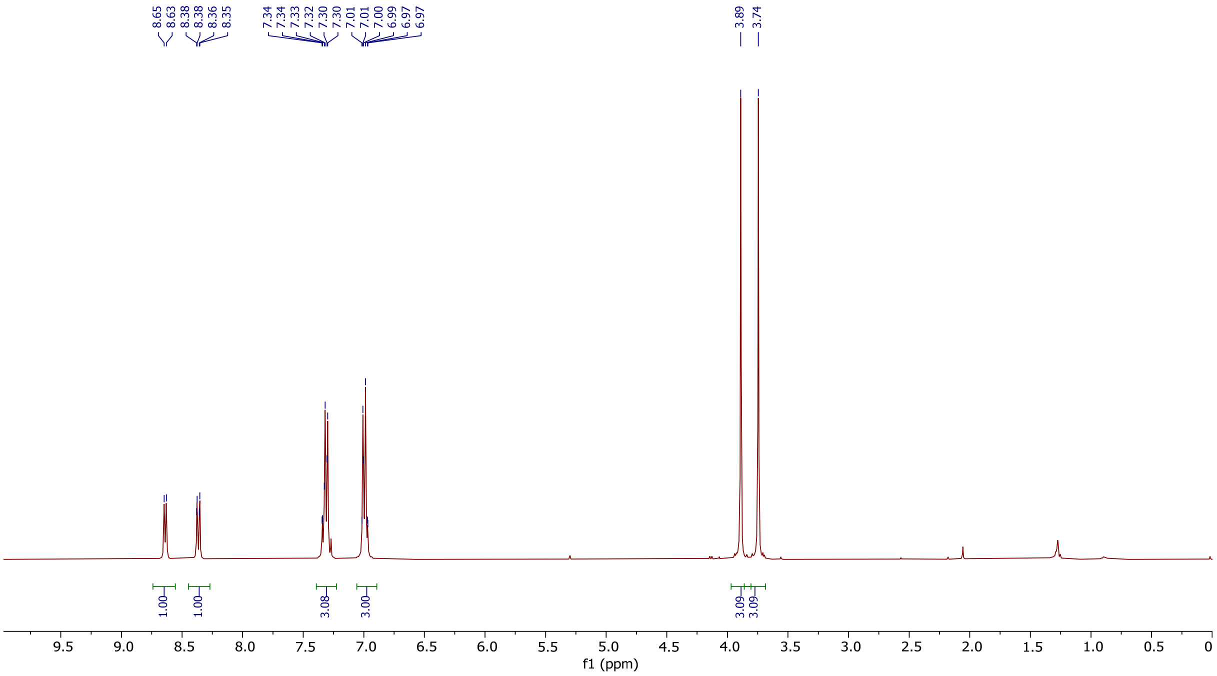

^{1}H NMR (400 MHz, $CDCl_3$) of **2o**.

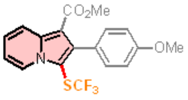

2o

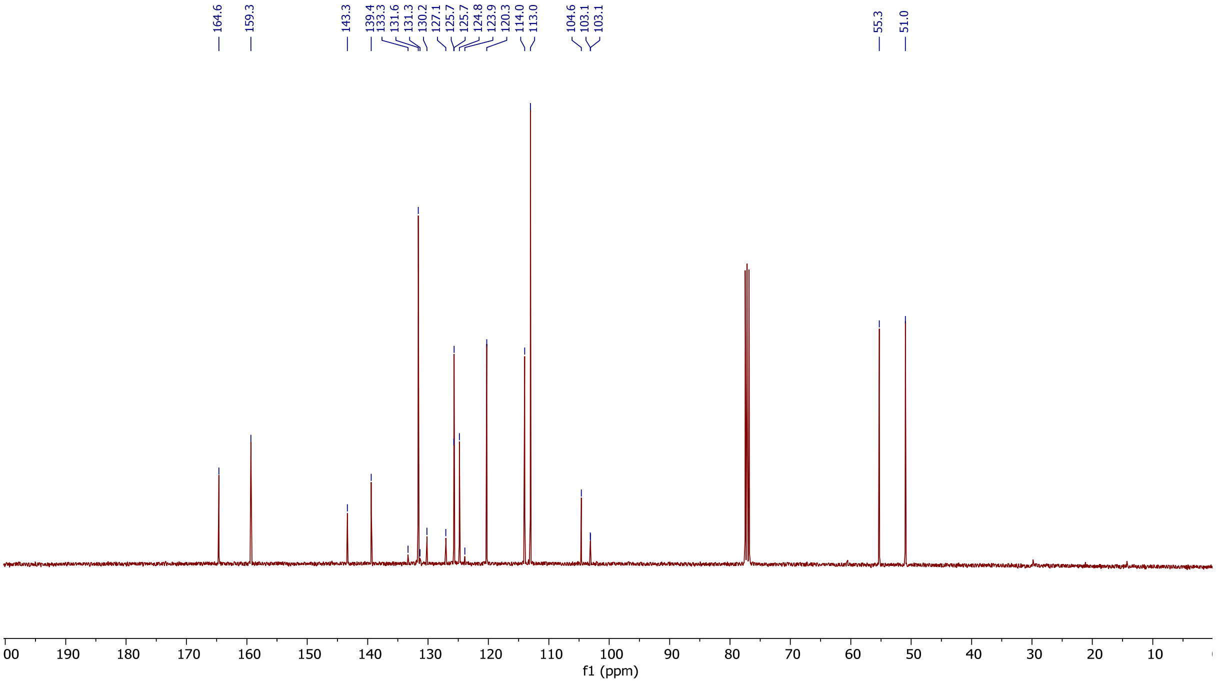

$^{13}C\{^{1}H\}$ NMR (100 MHz, $CDCl_3$) of **2o**.

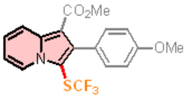

2o

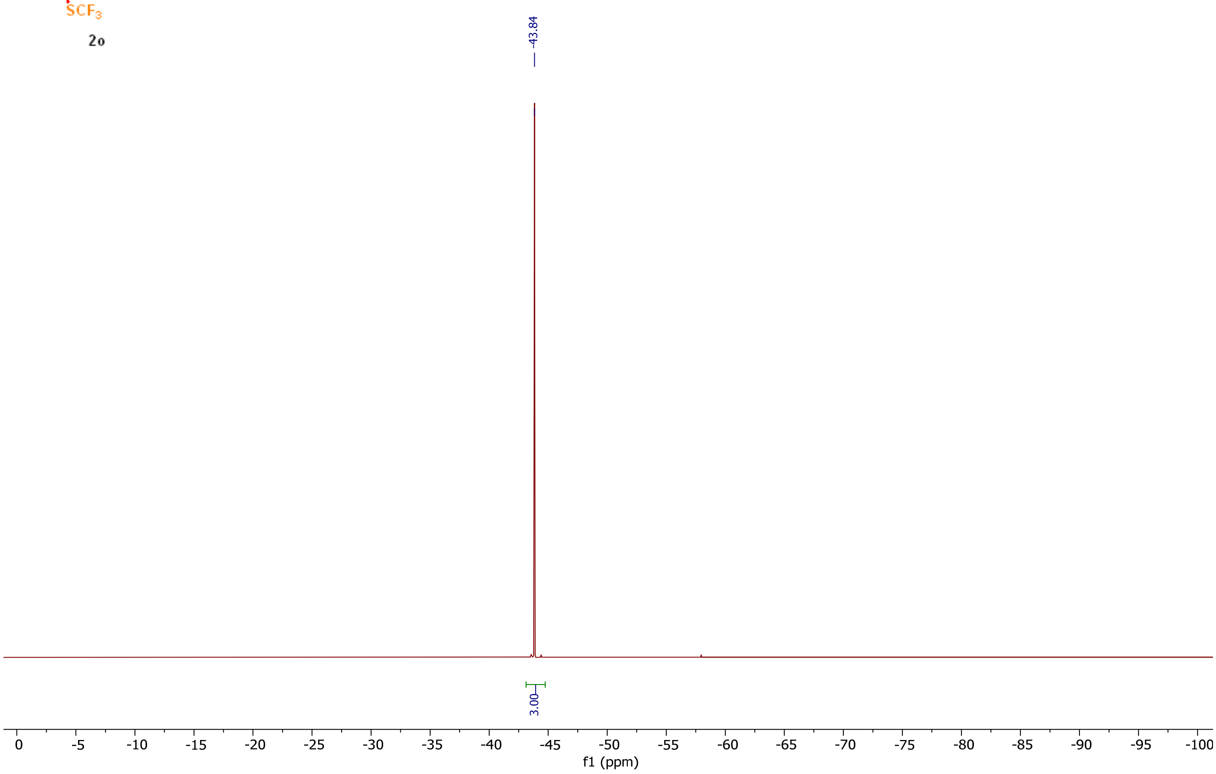

$^{19}F\{^{1}H\}$ NMR (377 MHz, $CDCl_3$) of **2o**.

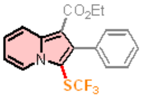

2p

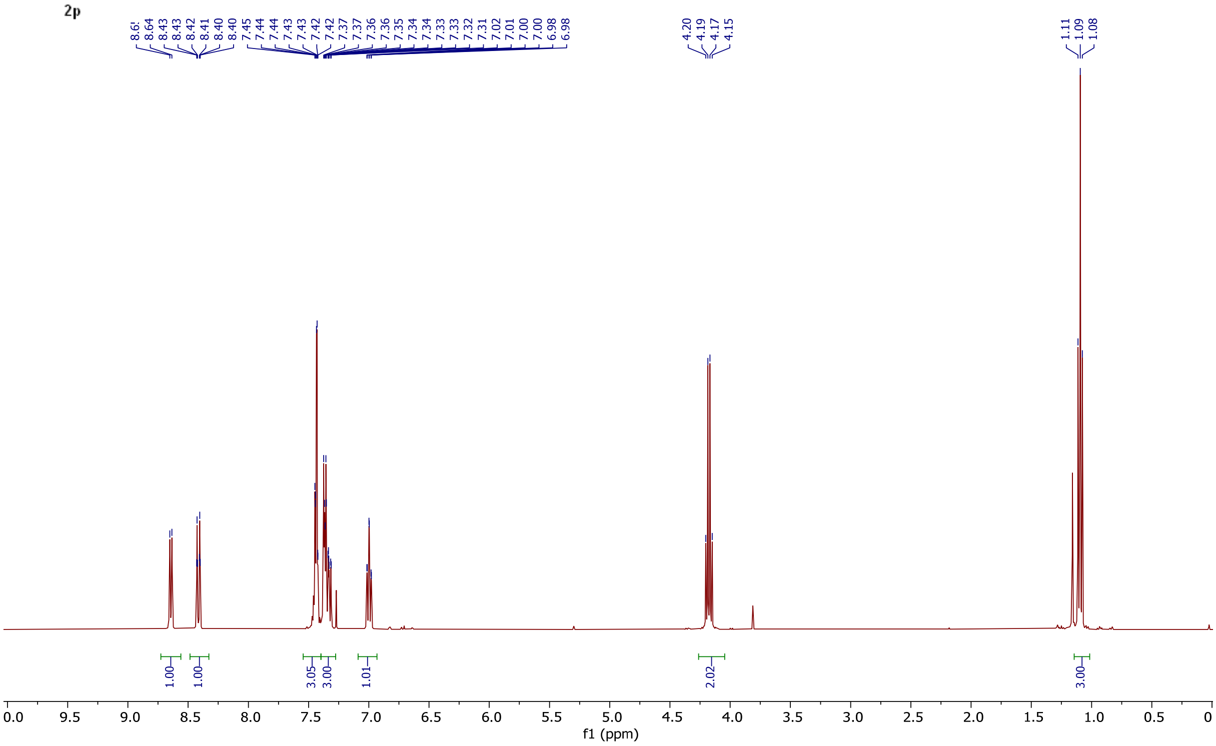

^{1}H NMR (400 MHz, $CDCl_3$) of **2p**.

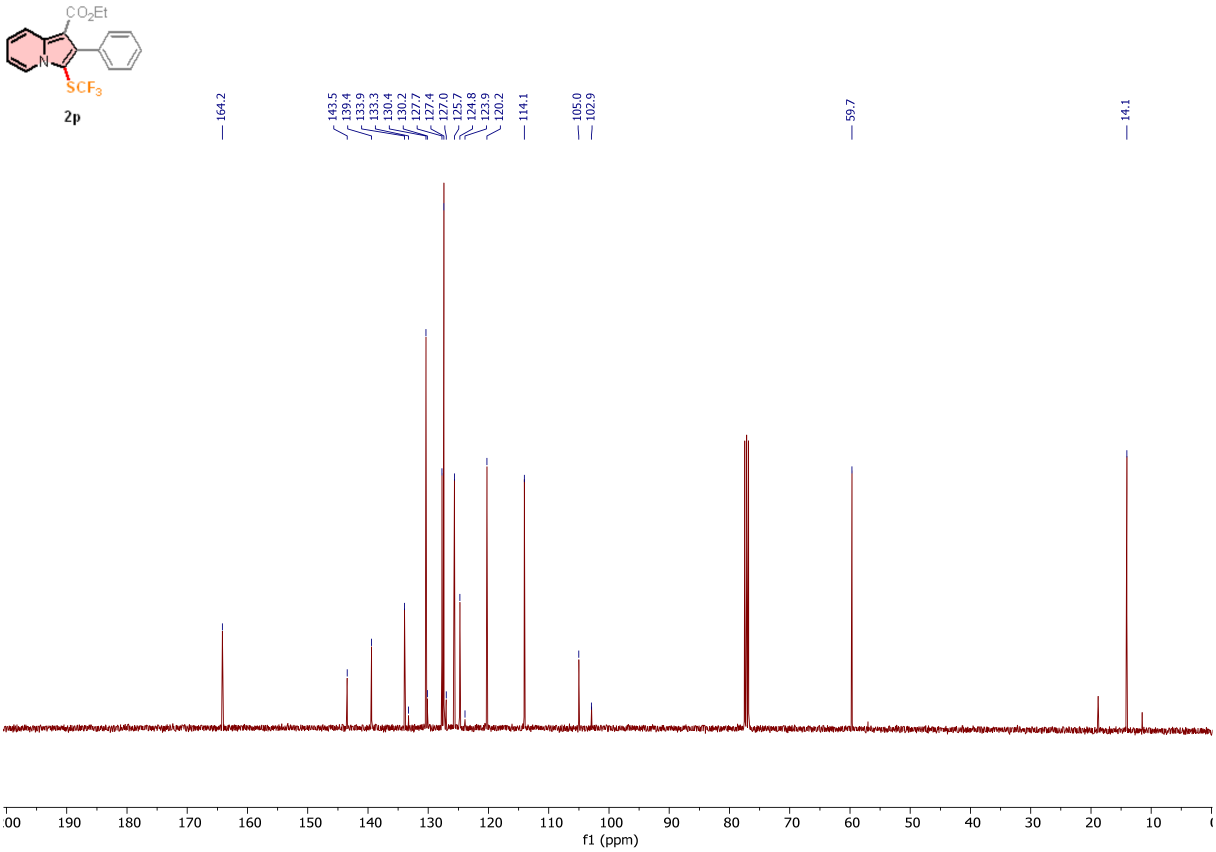

$^{13}C\{^{1}H\}$ NMR (100 MHz, $CDCl_3$) of **2p**.

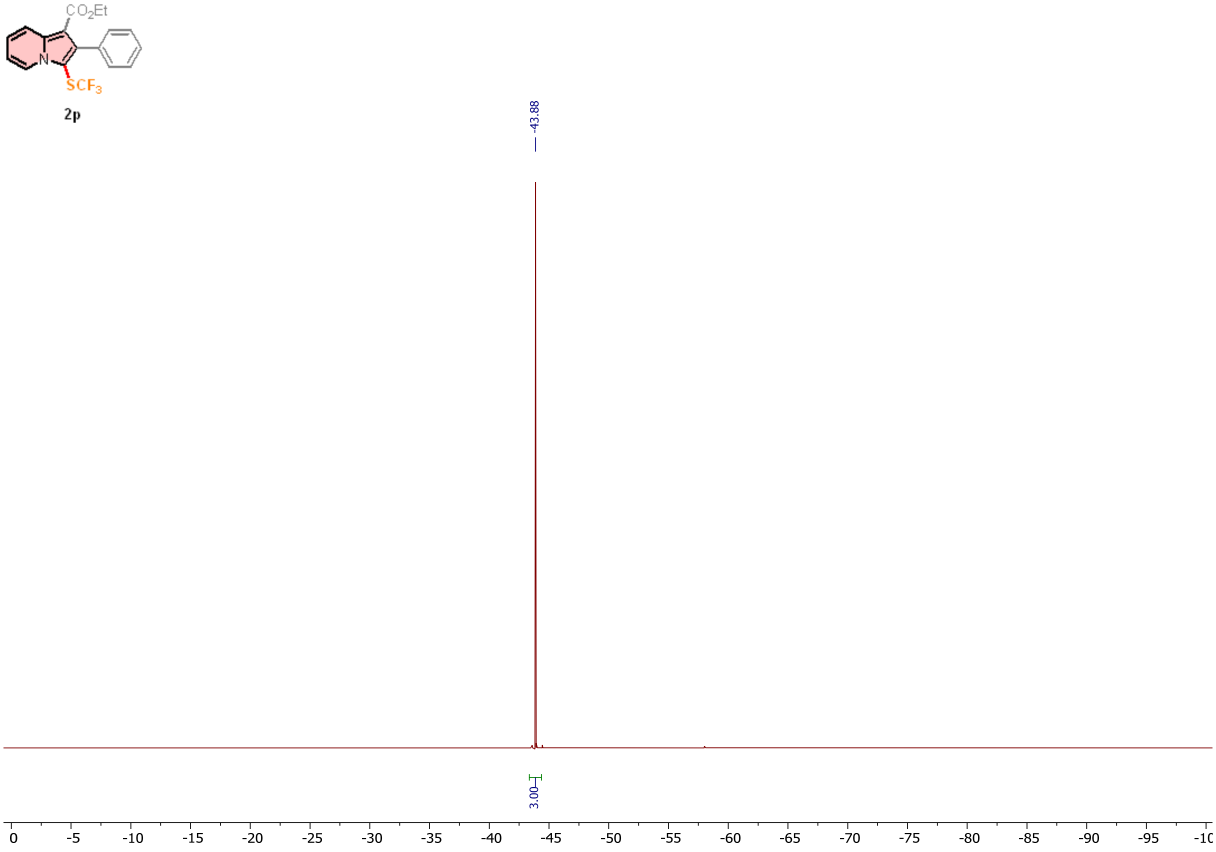

$^{19}F\{^{1}H\}$ NMR (377 MHz, $CDCl_3$) of **2p**.

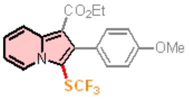

2q

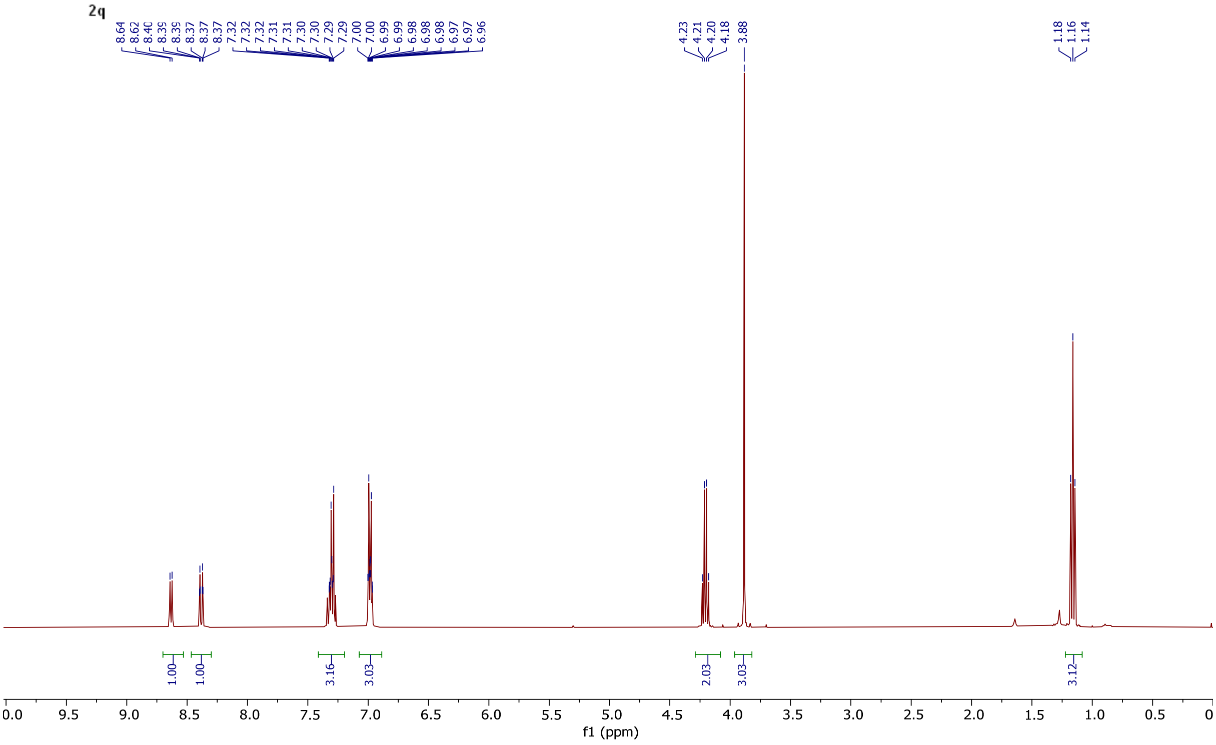

^{1}H NMR (400 MHz, $CDCl_3$) of **2q**.

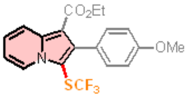

2q

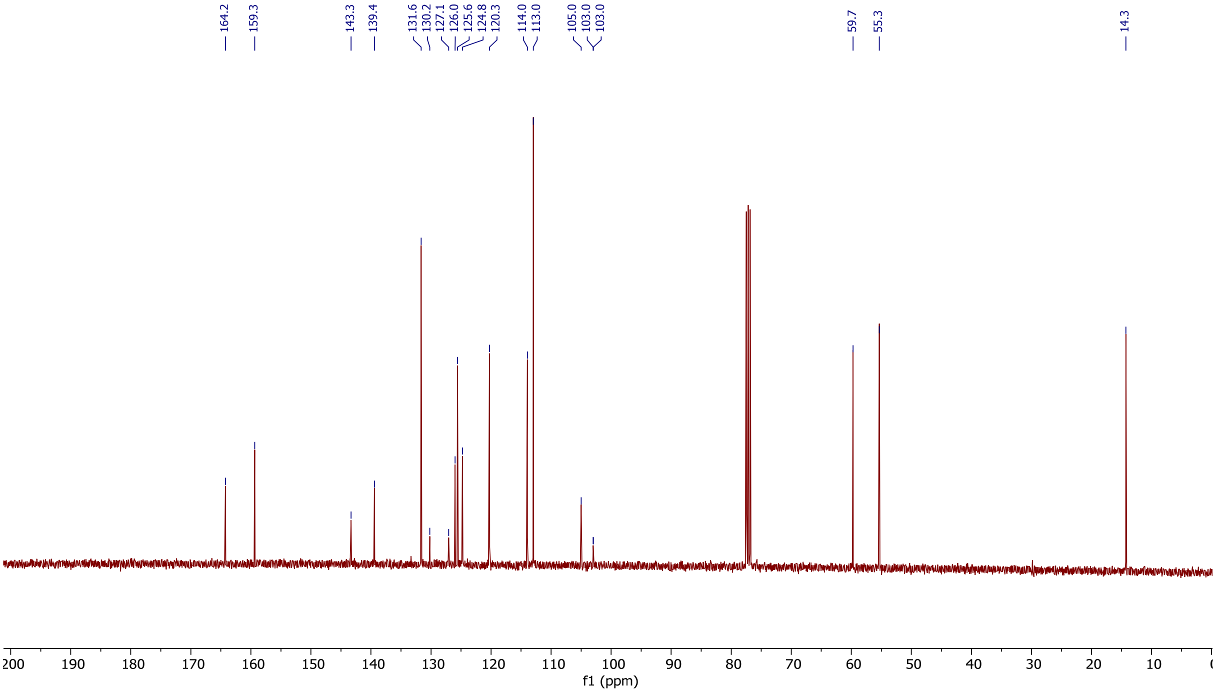

$^{13}C\{^1H\}$ NMR (100 MHz, $CDCl_3$) of **2q**.

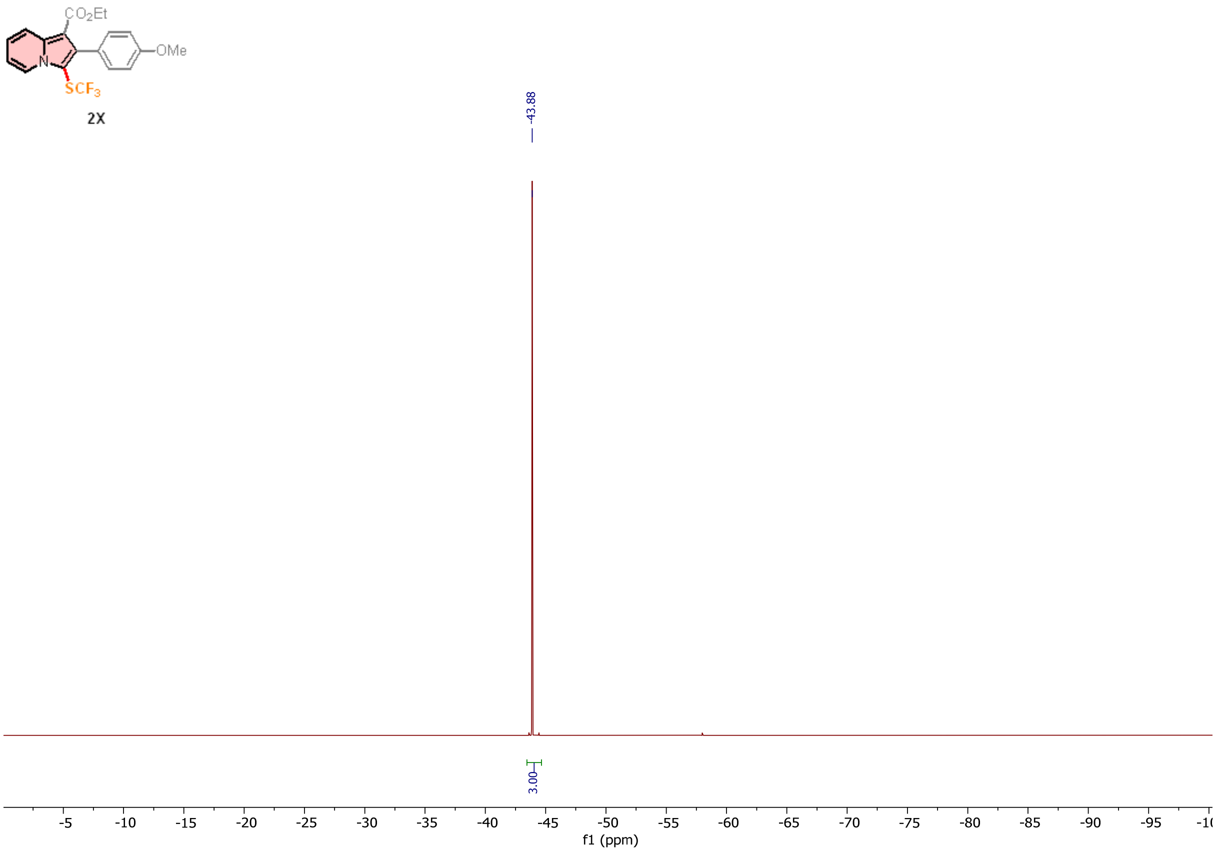

$^{19}F\{^1H\}$ NMR (377 MHz, $CDCl_3$) of **2q**.

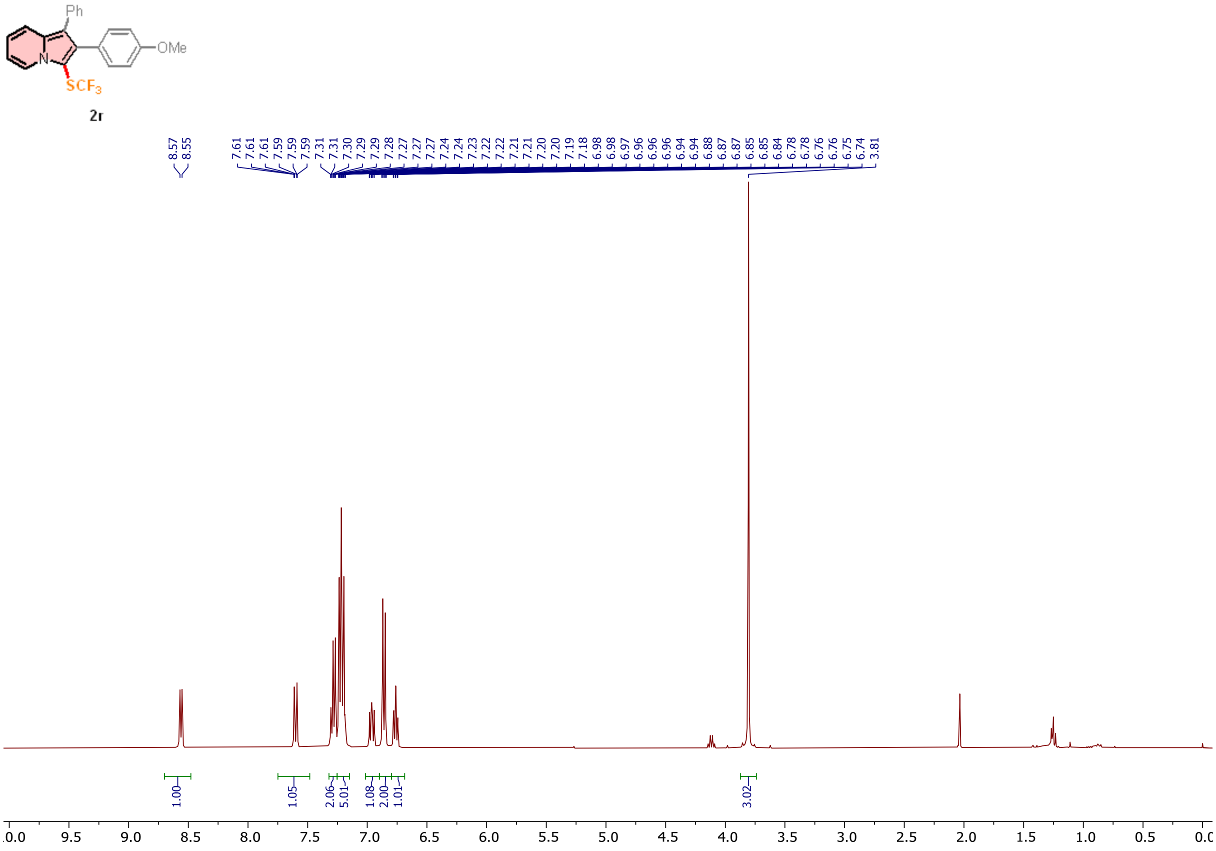

^1H NMR (400 MHz, $CDCl_3$) of **2r**.

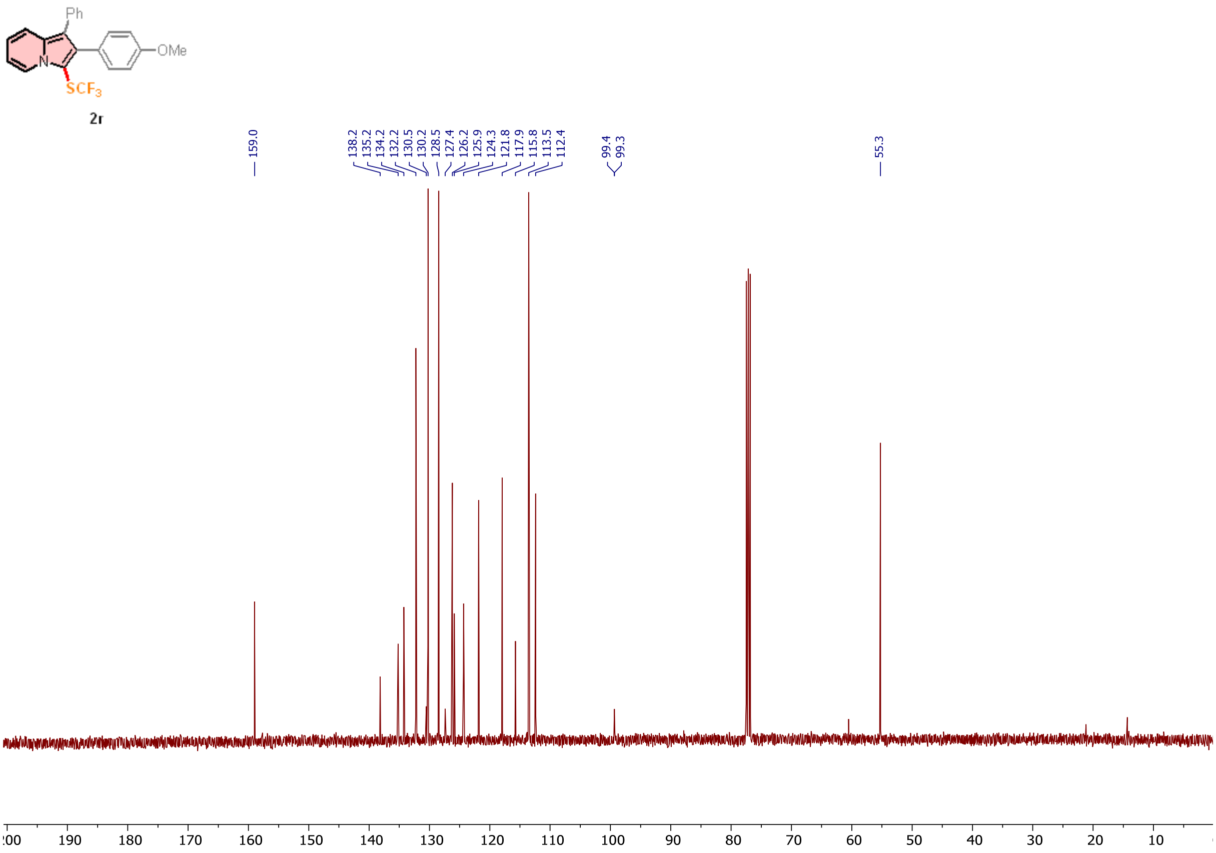

$^{13}C\{^{1}H\}$ NMR (100 MHz, $CDCl_3$) of **2r**.

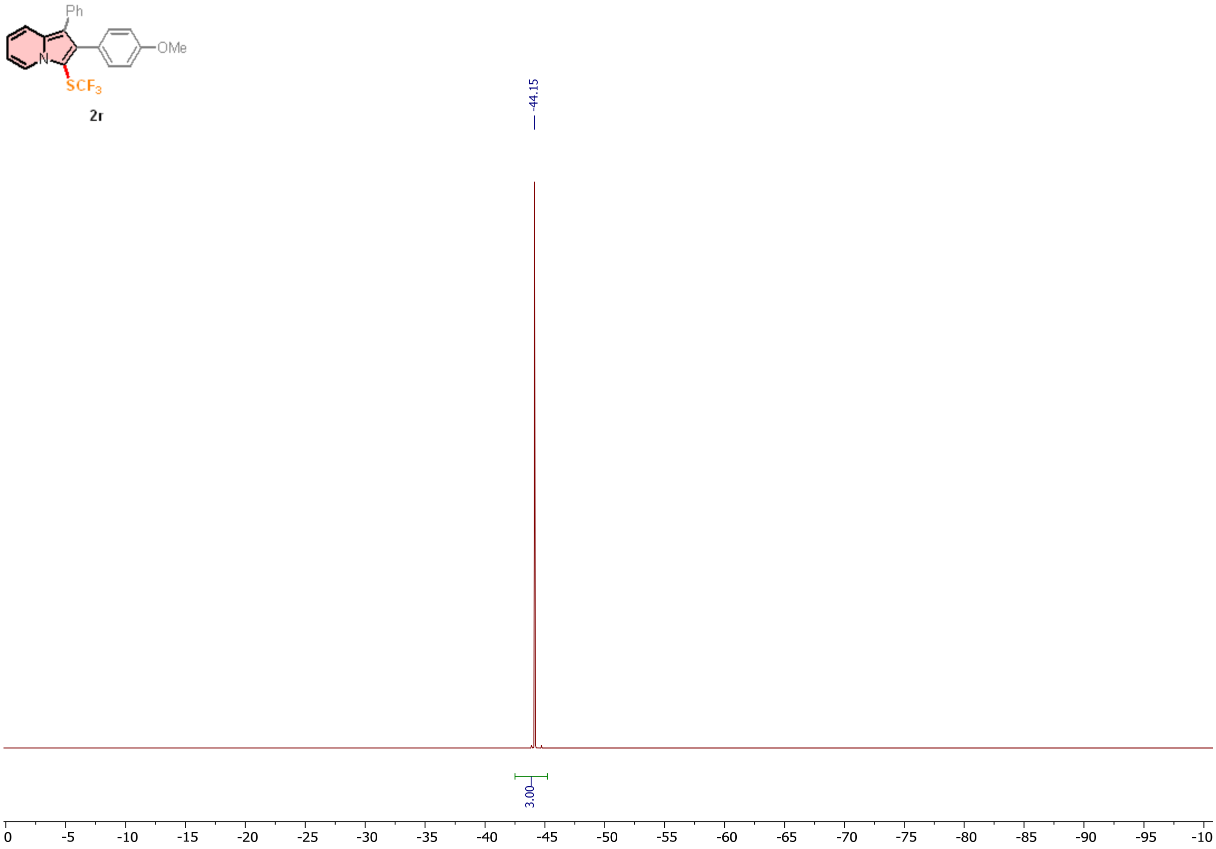

$^{19}F\{^{1}H\}$ NMR (377 MHz, $CDCl_3$) of **2r**.

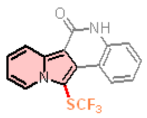

2s

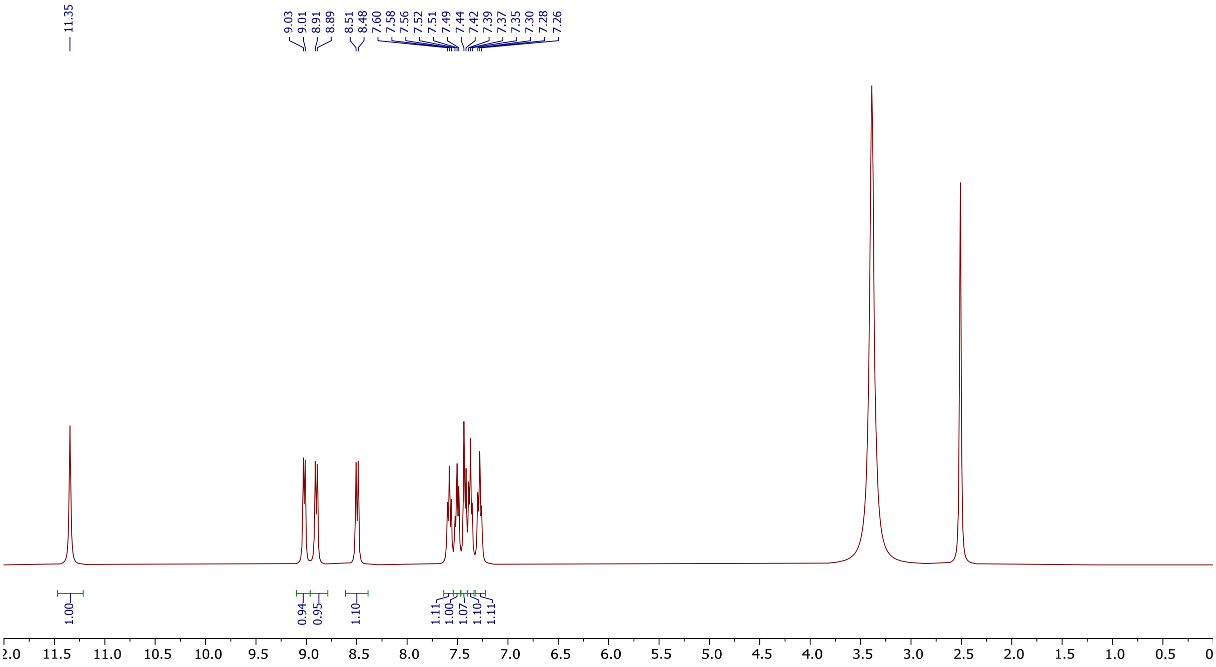

^{1}H NMR (400 MHz, DMSO-d[6]) of **2s**.

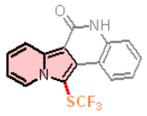

2s

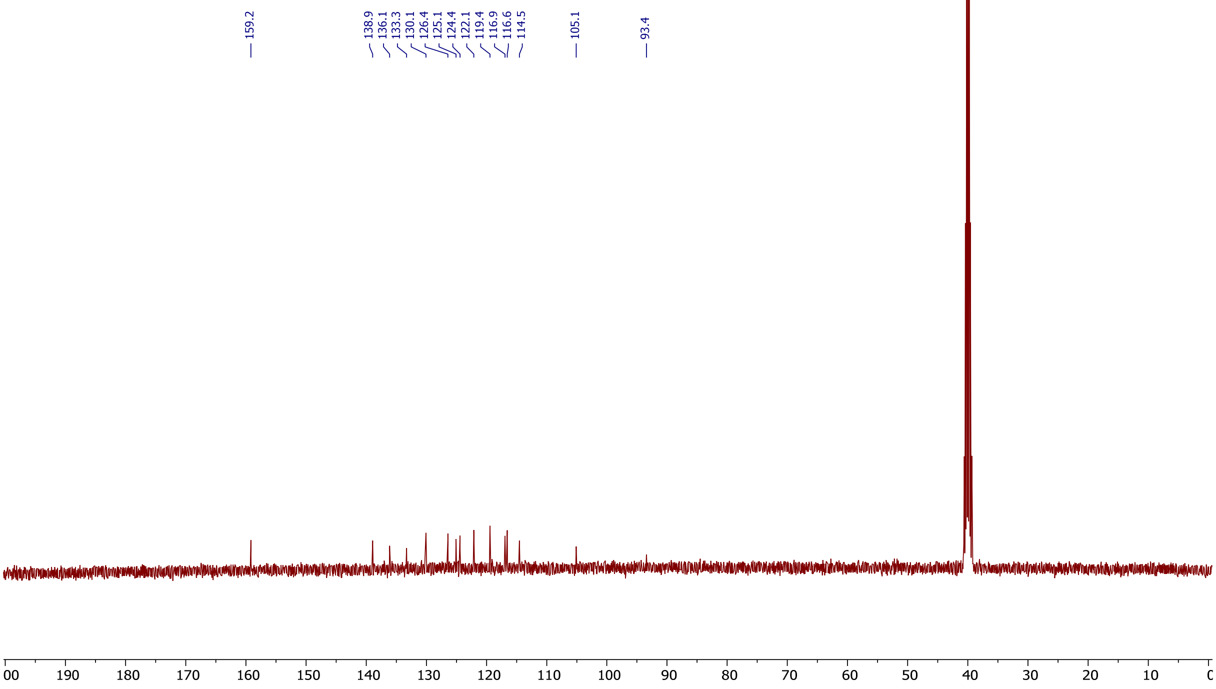

$^{13}C\{^{1}H\}$ NMR (100 MHz, DMSO-d[6]) of **2s**.

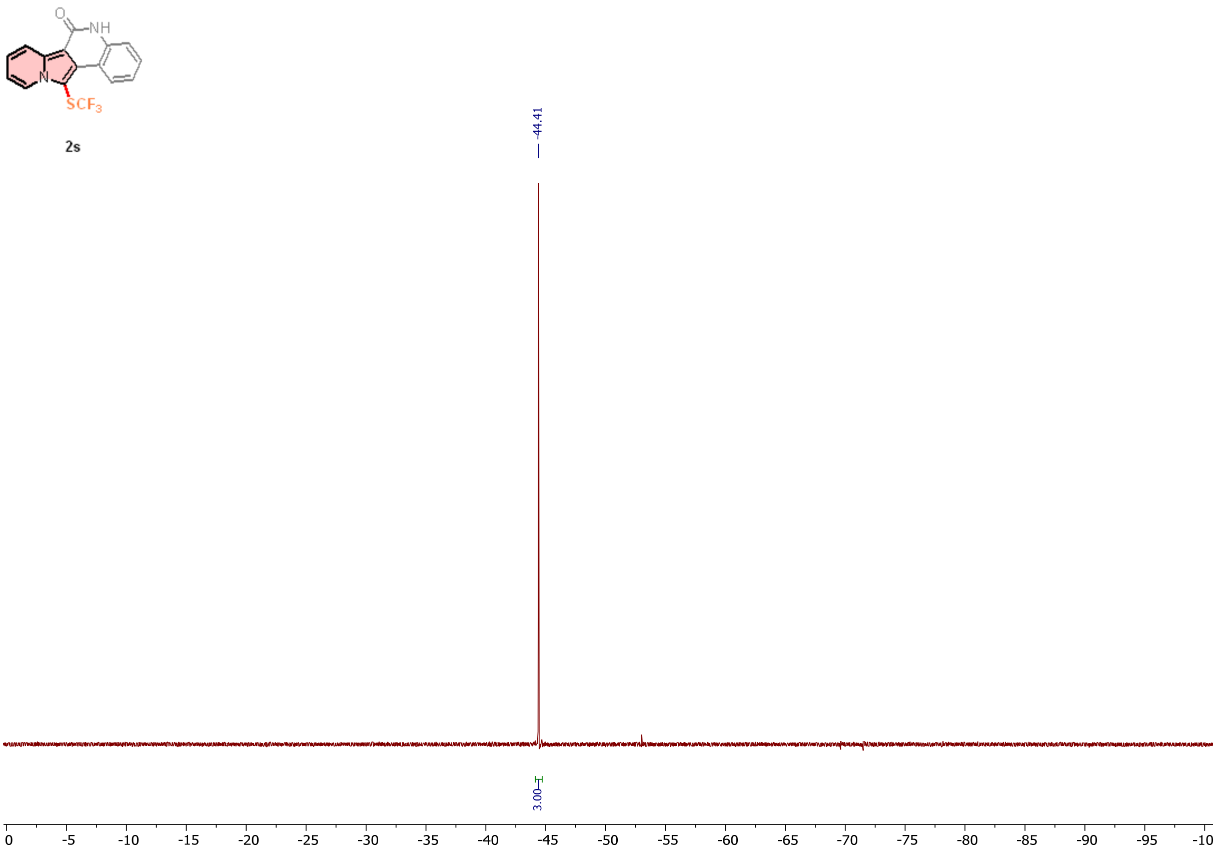

$^{19}F\{^{1}H\}$ NMR (377 MHz, DMSO-d^{6}) of **2s**.

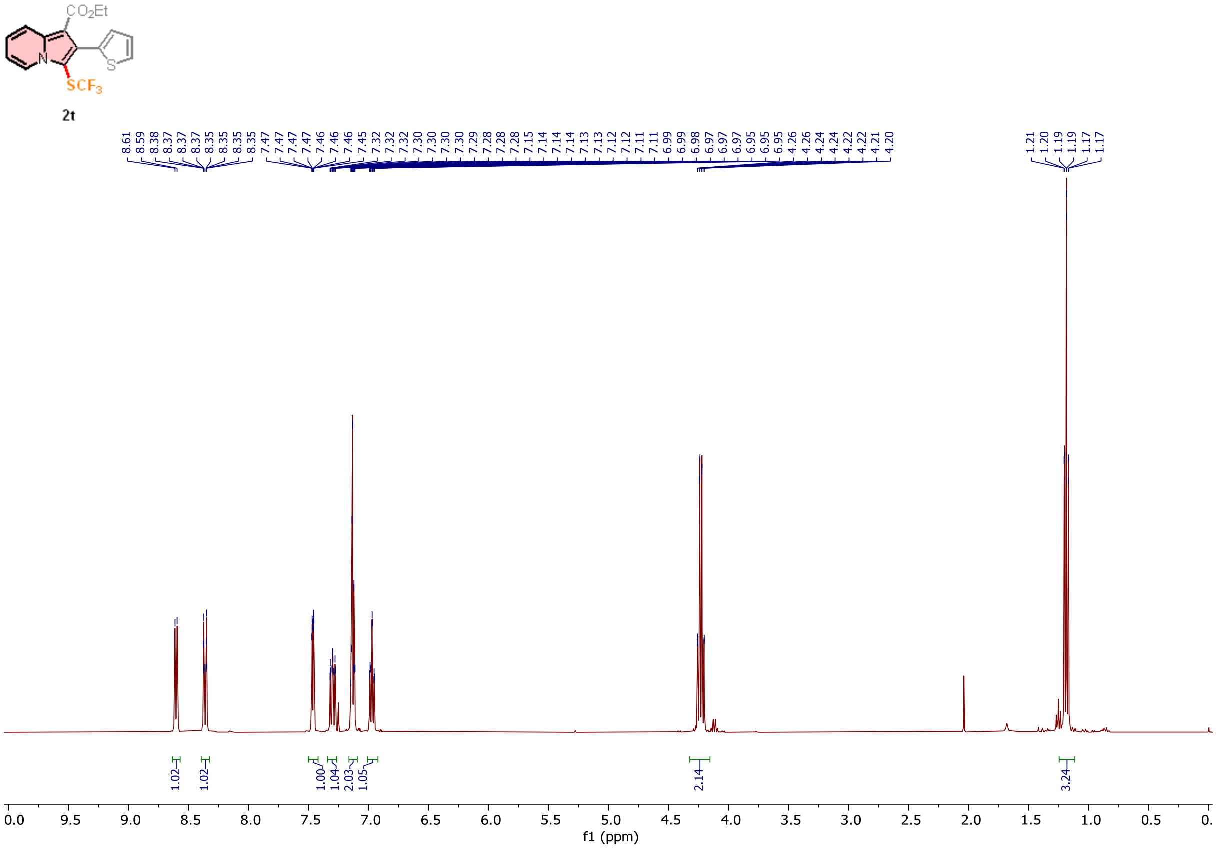

^{1}H NMR (400 MHz, $CDCl_3$) of **2t**.

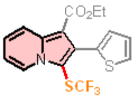

2t

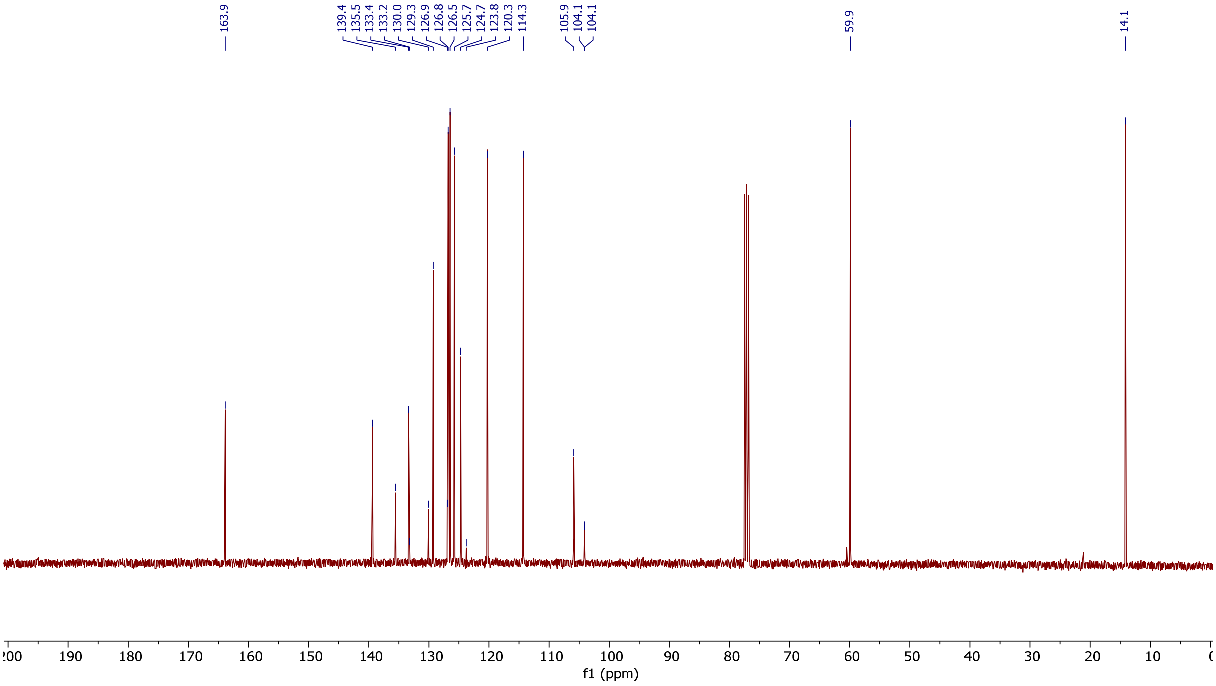

$^{13}C\{^1H\}$ NMR (100 MHz, $CDCl_3$) of **2t**.

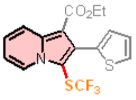

2t

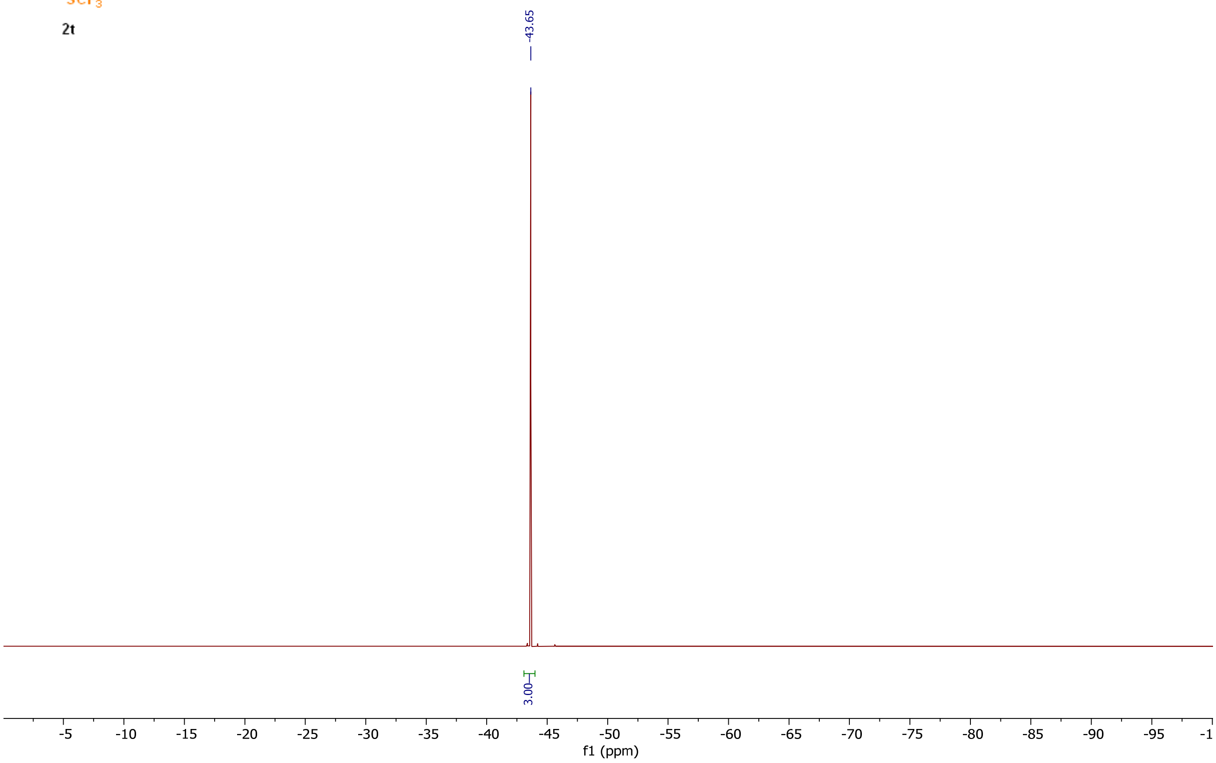

$^{19}F\{^1H\}$ NMR (377 MHz, $CDCl_3$) of **2t**.

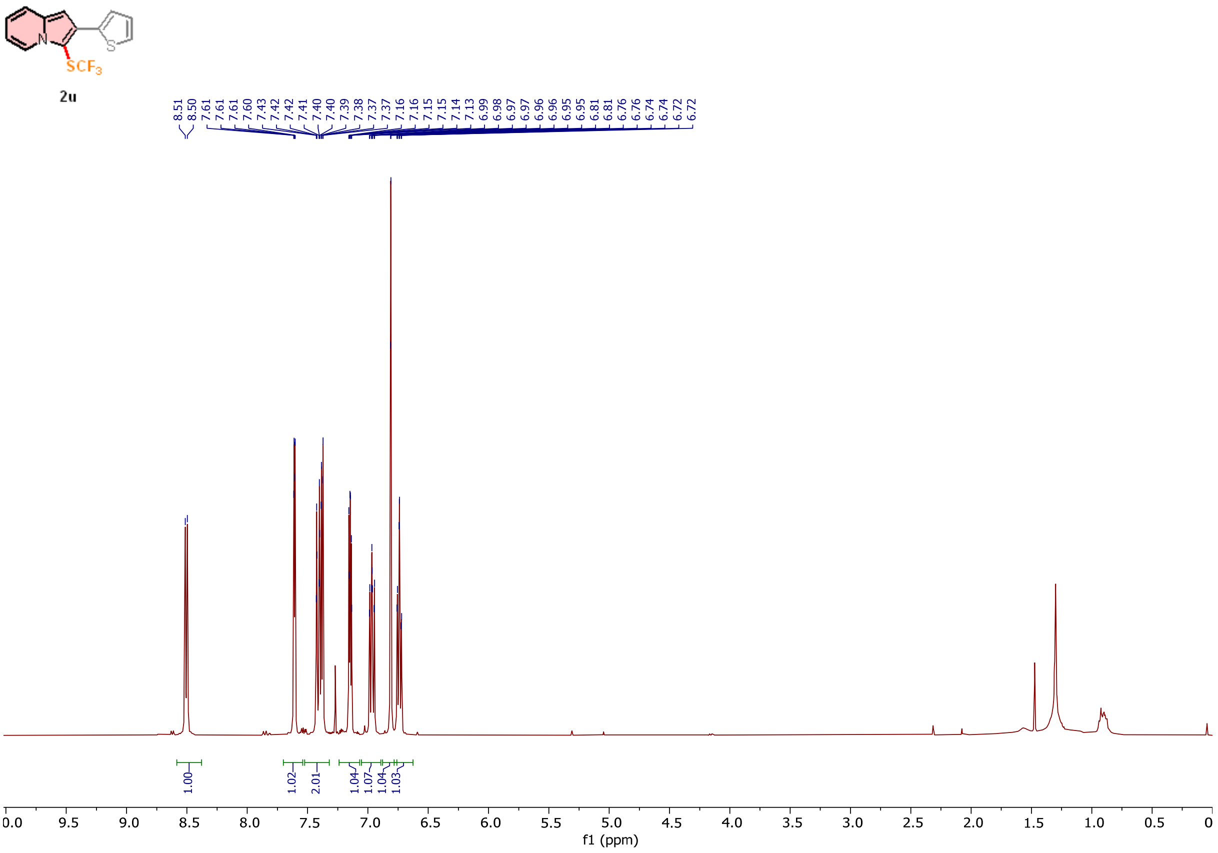

^{1}H NMR (400 MHz, $CDCl_3$) of **2u**.

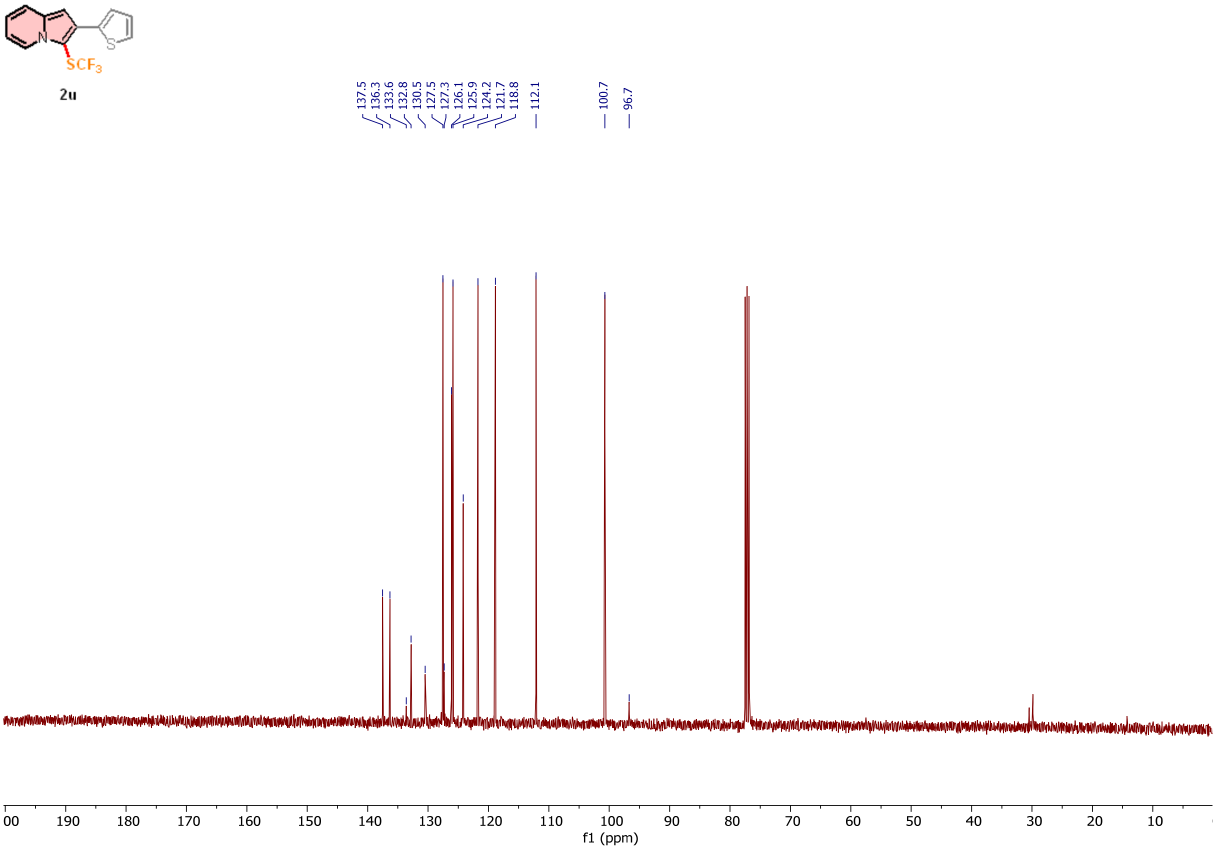

$^{13}C\{^{1}H\}$ NMR (100 MHz, $CDCl_3$) of **2u**.

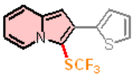

2u

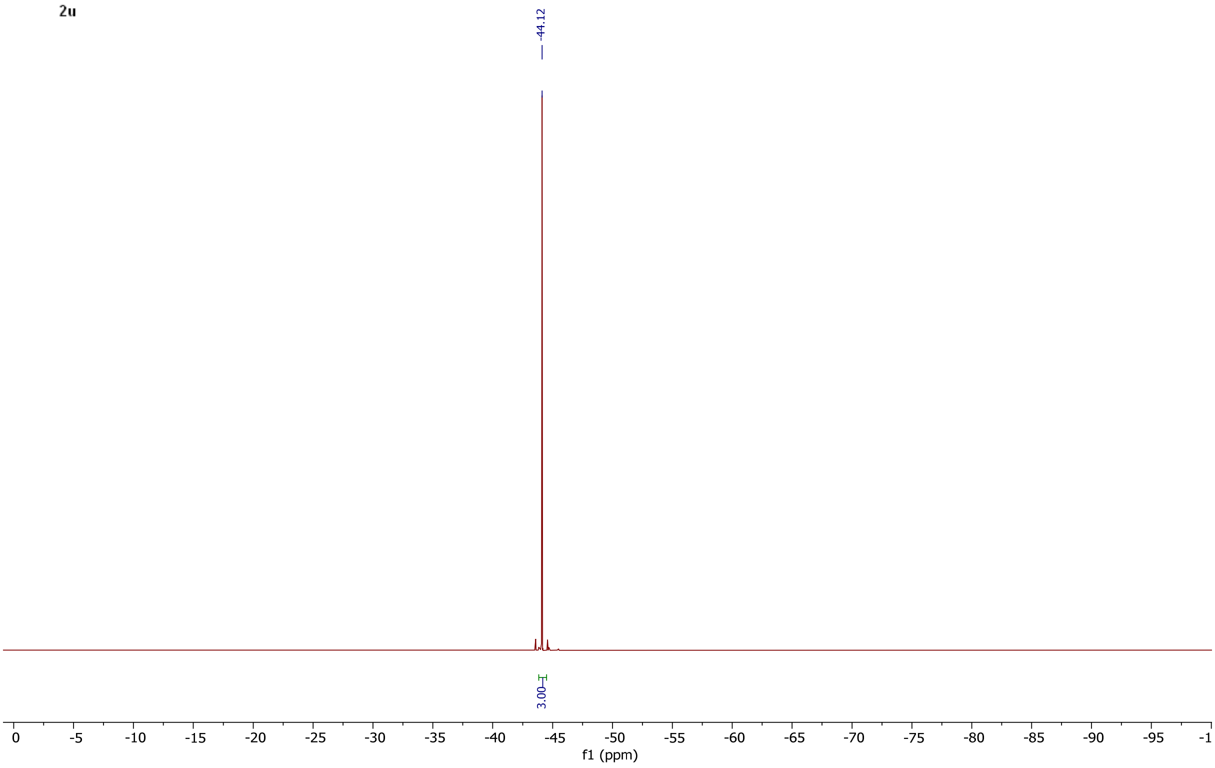

$^{19}F\{^{1}H\}$ NMR (377 MHz, $CDCl_3$) of **2u**.

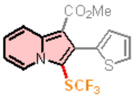

2v

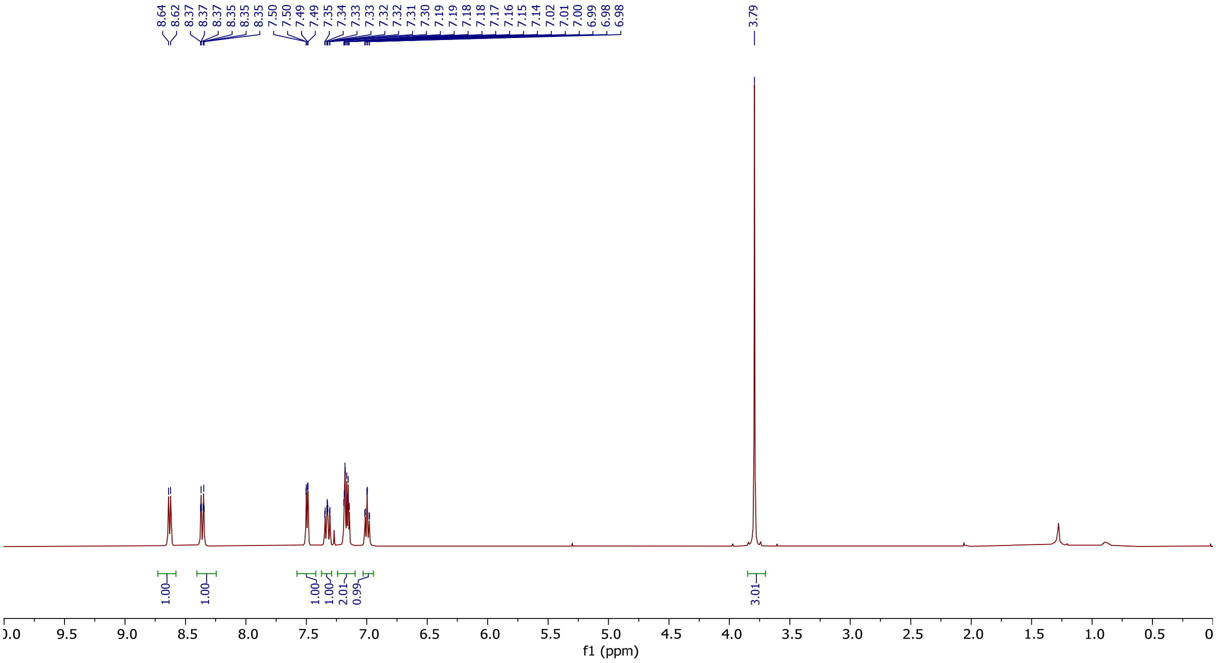

^{1}H NMR (400 MHz, $CDCl_3$) of **2v**.

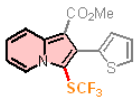

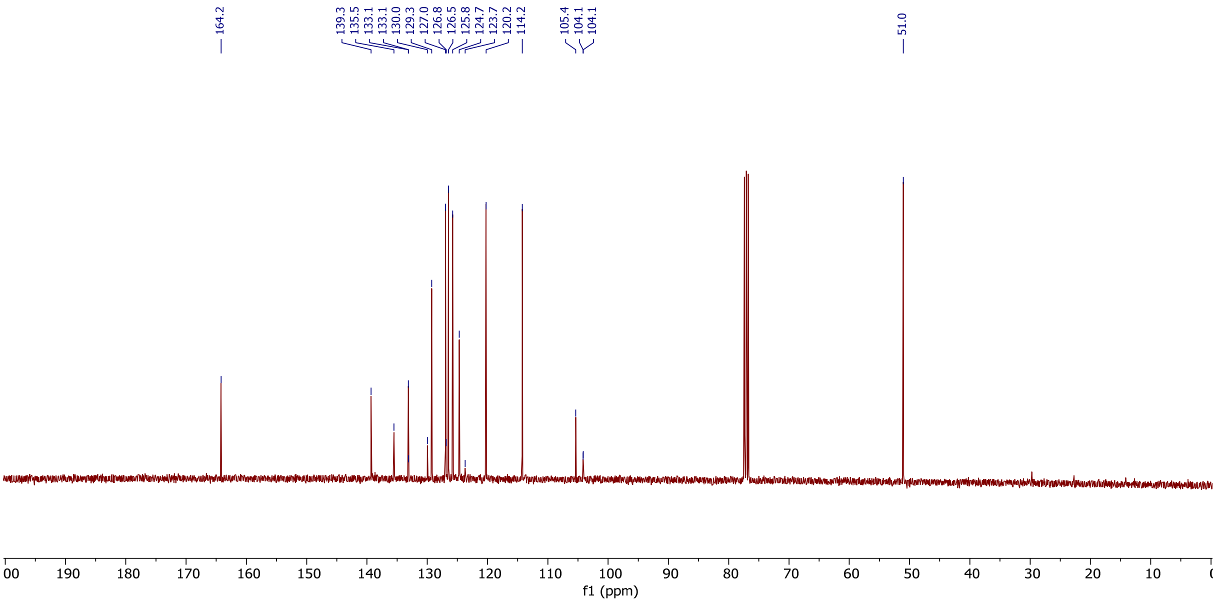

$^{13}C\{^{1}H\}$ NMR (100 MHz, $CDCl_3$) of **2v**.

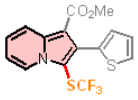

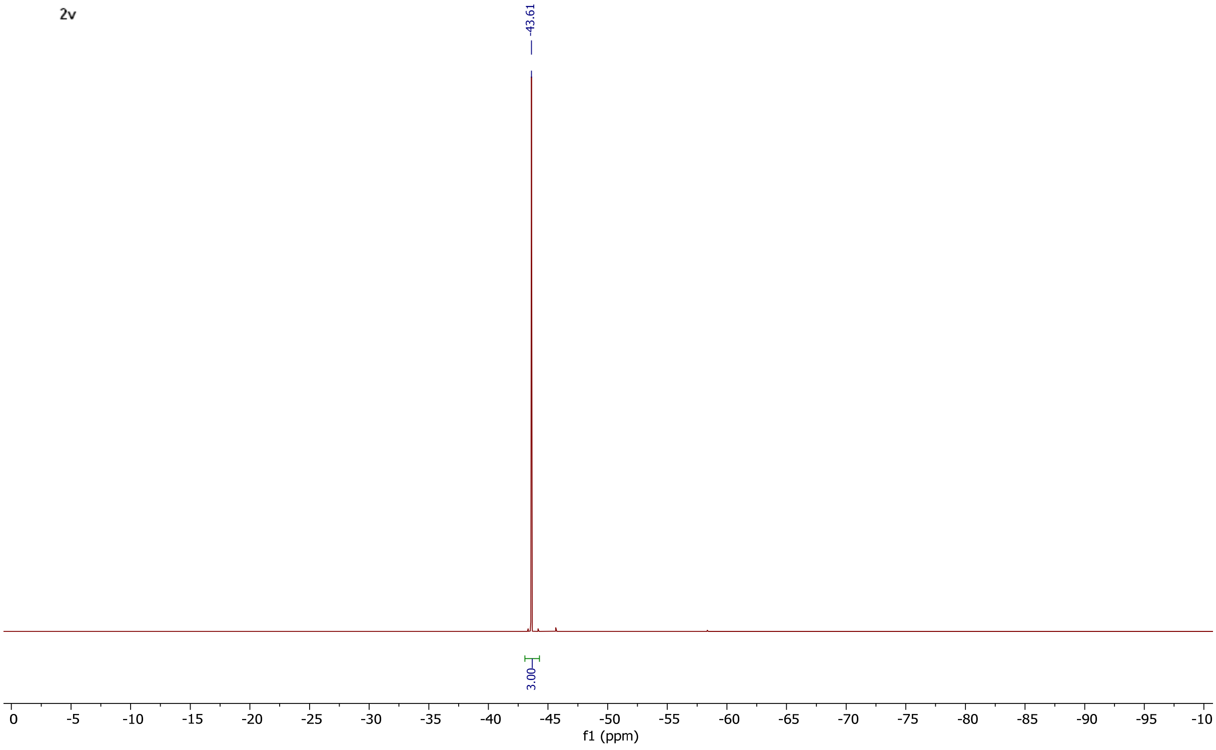

$^{19}F\{^{1}H\}$ NMR (377 MHz, $CDCl_3$) of **2v**.

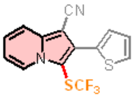

2w

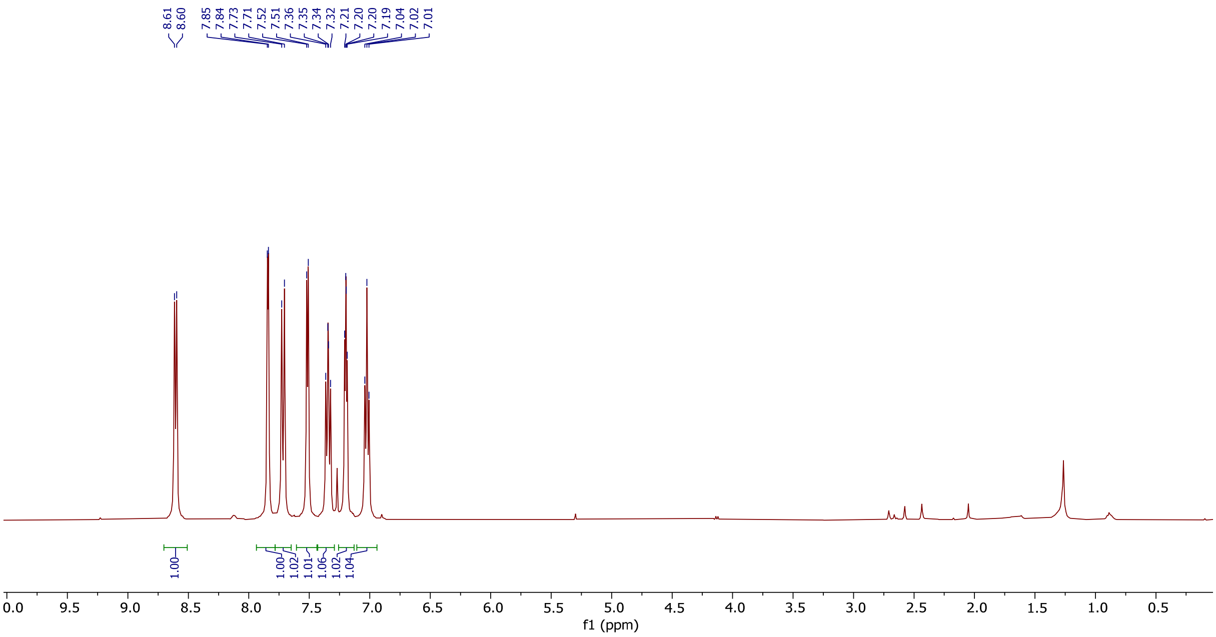

^{1}H NMR (400 MHz, $CDCl_3$) of **2w**.

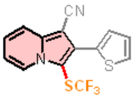

2w

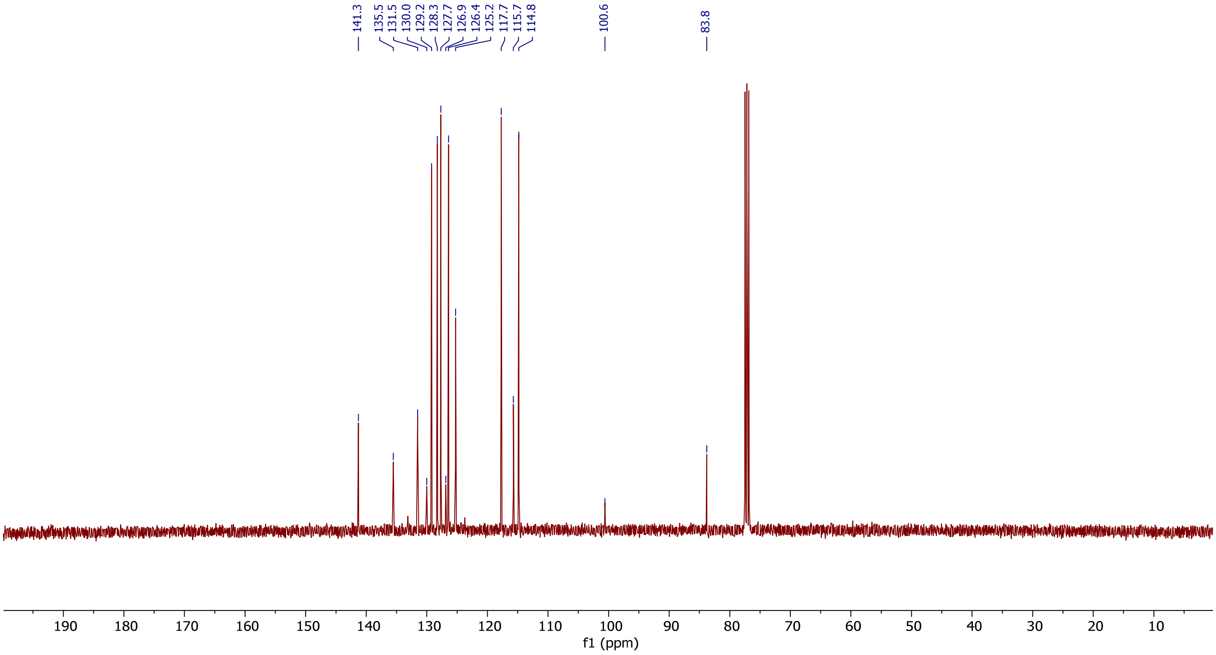

$^{13}C\{^{1}H\}$ NMR (100 MHz, $CDCl_3$) of **2w**.

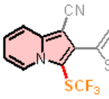

2w

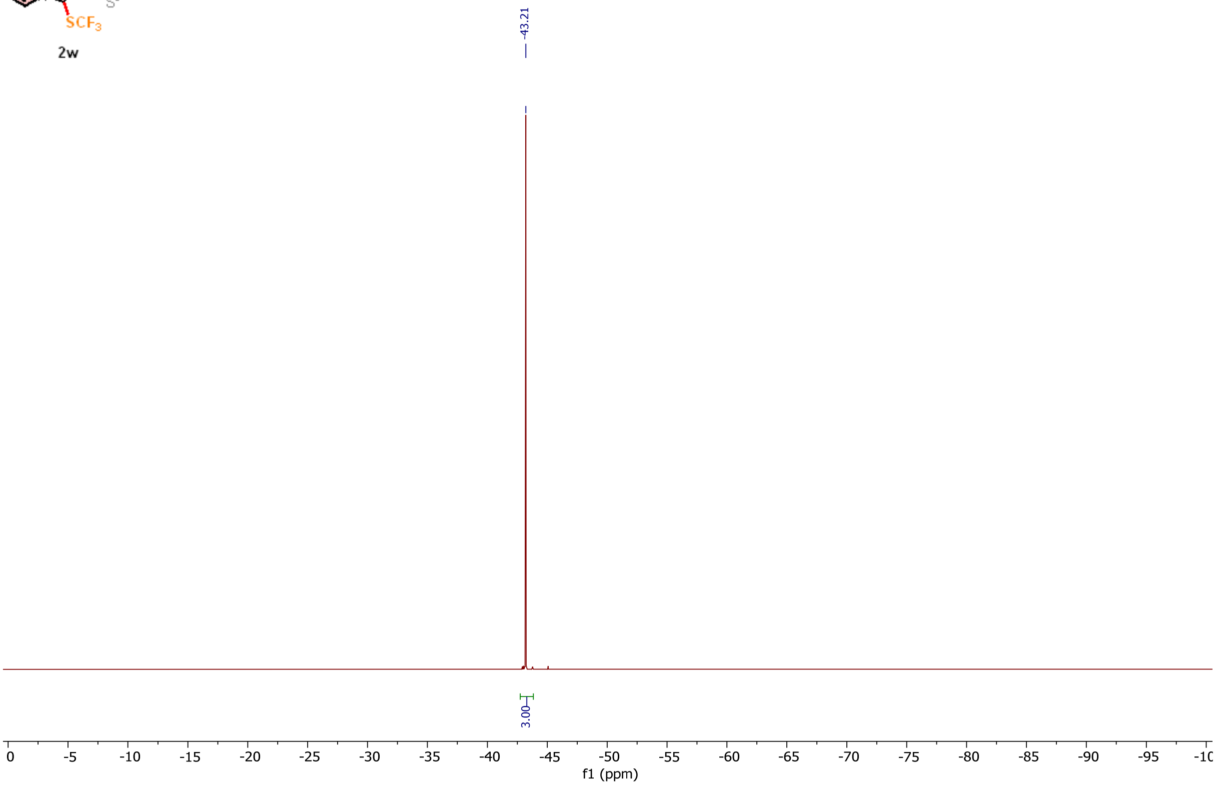

$^{19}F\{^{1}H\}$ NMR (377 MHz, $CDCl_3$) of **2w**.

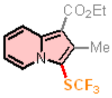

2x

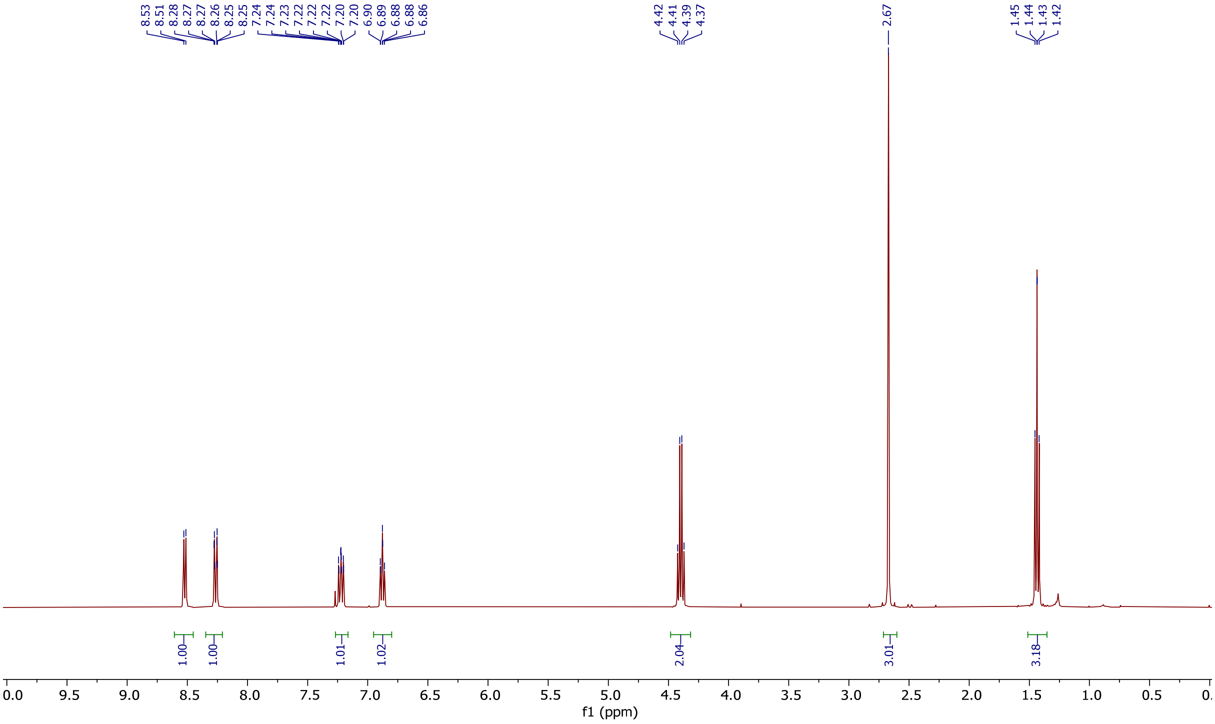

^{1}H NMR (400 MHz, $CDCl_3$) of **2x**.

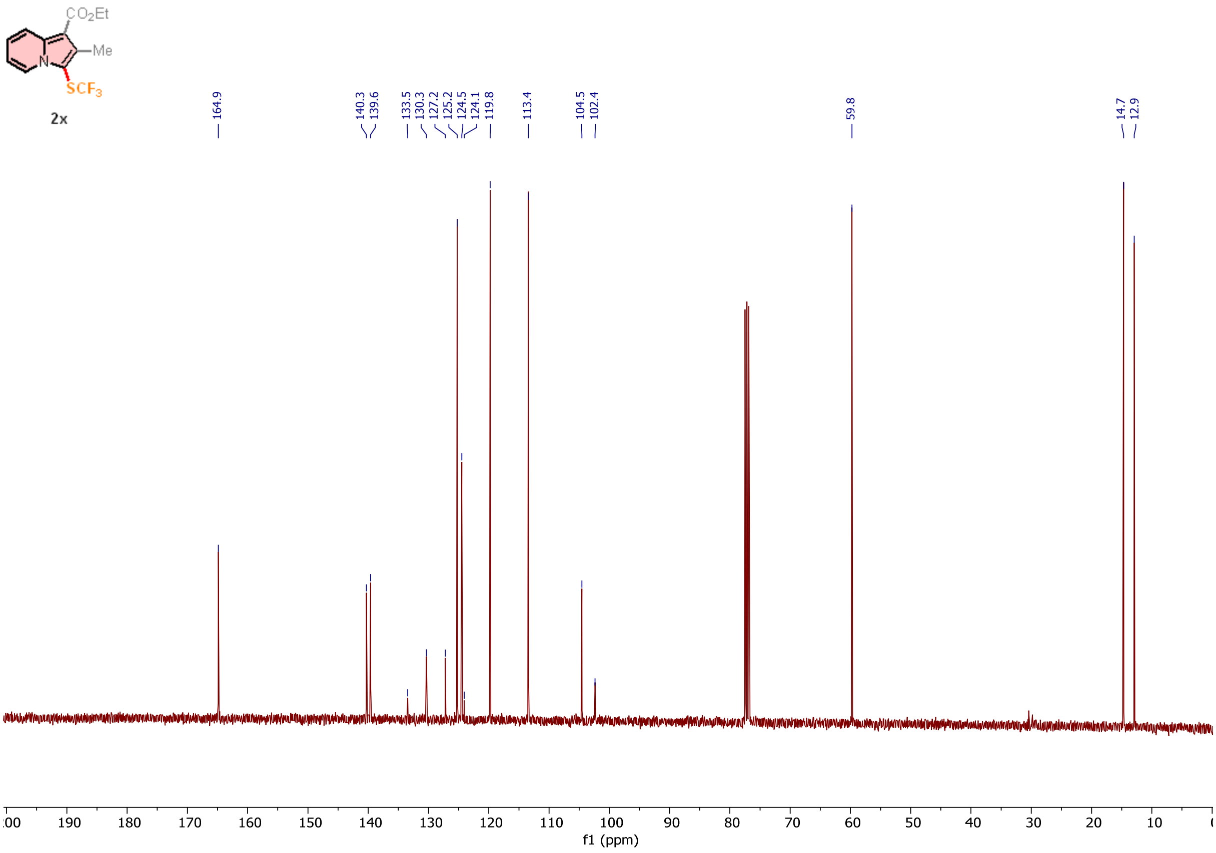

$^{13}C\{^{1}H\}$ NMR (100 MHz, $CDCl_3$) of **2x**.

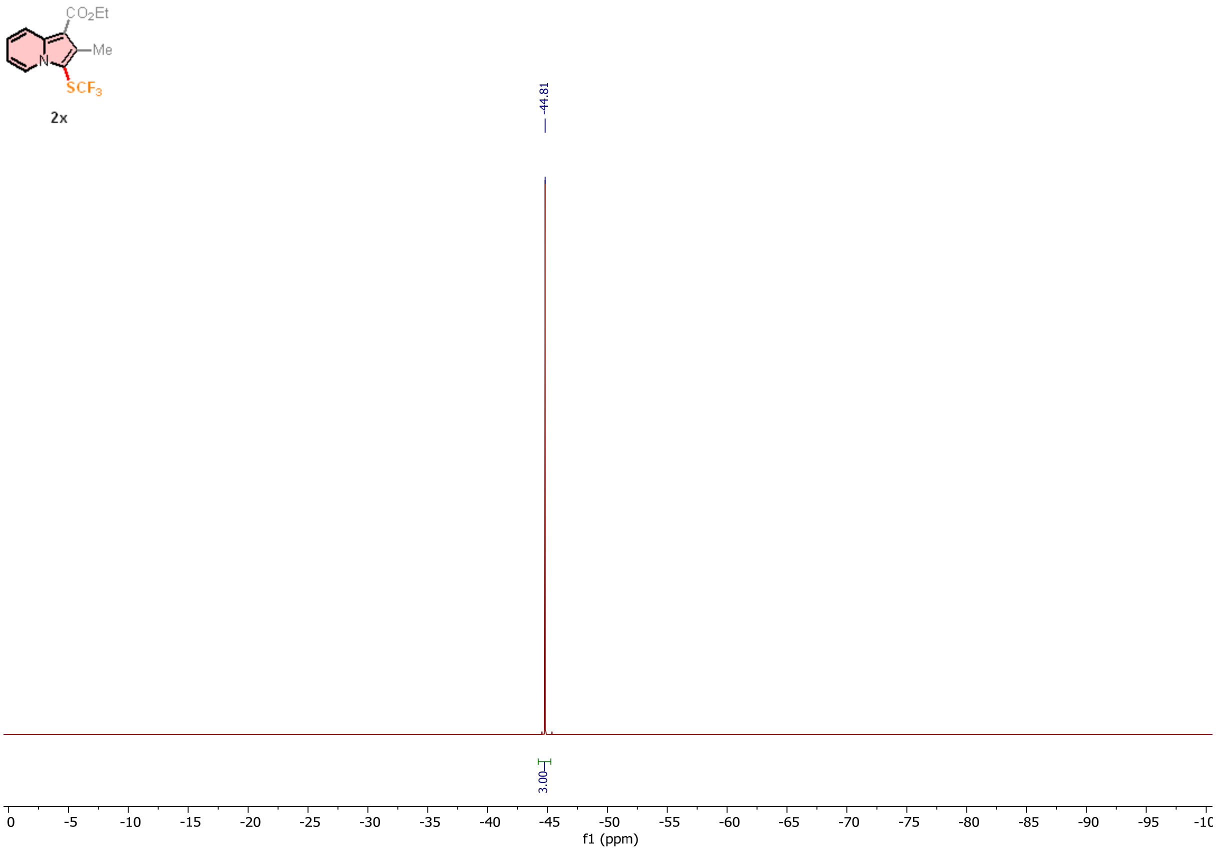

$^{19}F\{^{1}H\}$ NMR (377 MHz, $CDCl_3$) of **2x**.

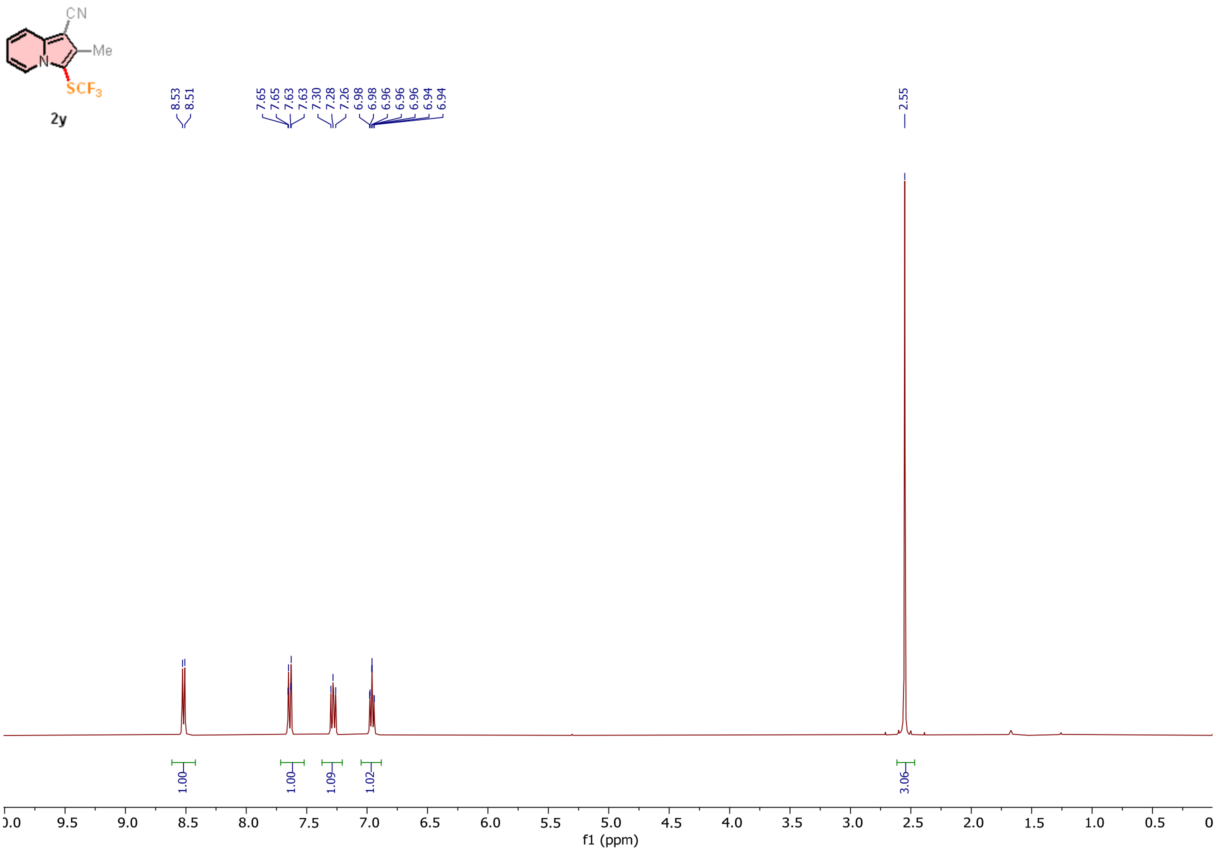

^{1}H NMR (400 MHz, $CDCl_3$) of **2y**.

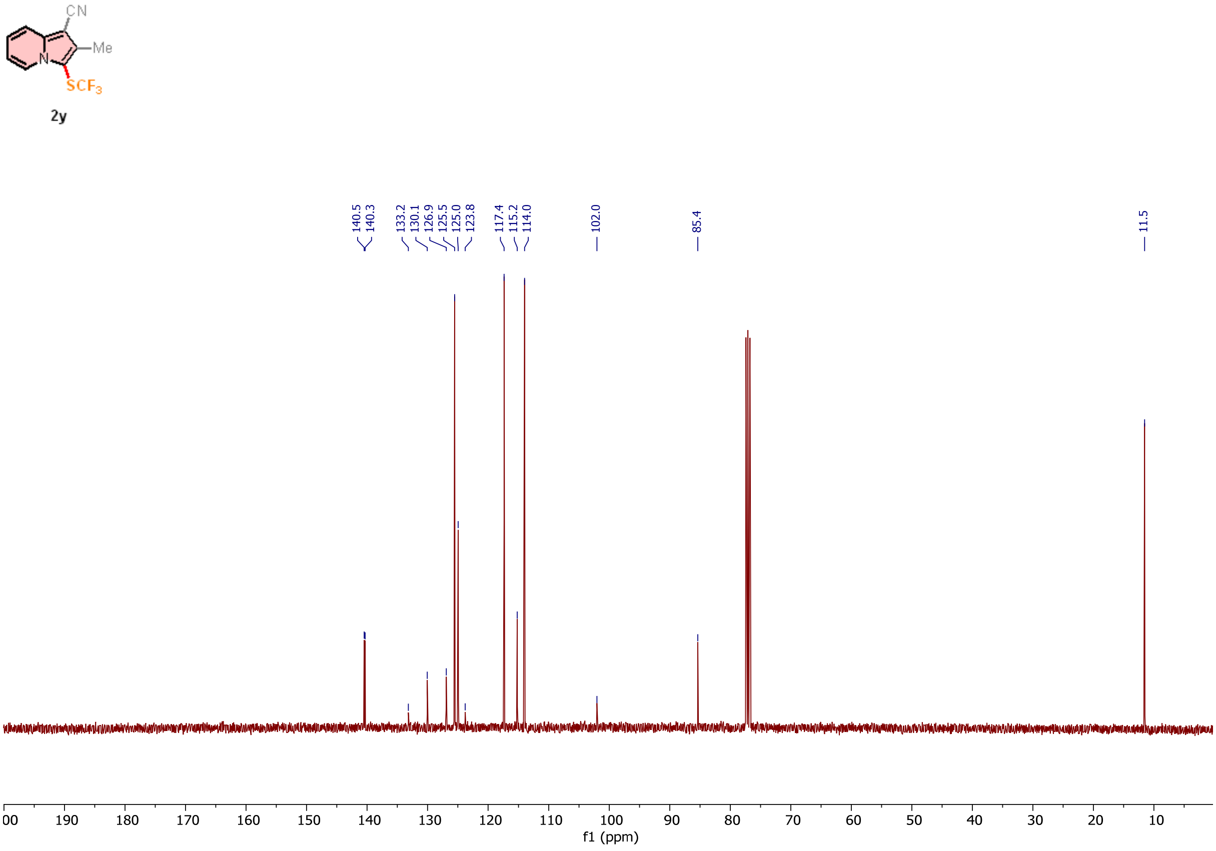

$^{13}C\{^{1}H\}$ NMR (100 MHz, $CDCl_3$) of **2y**.

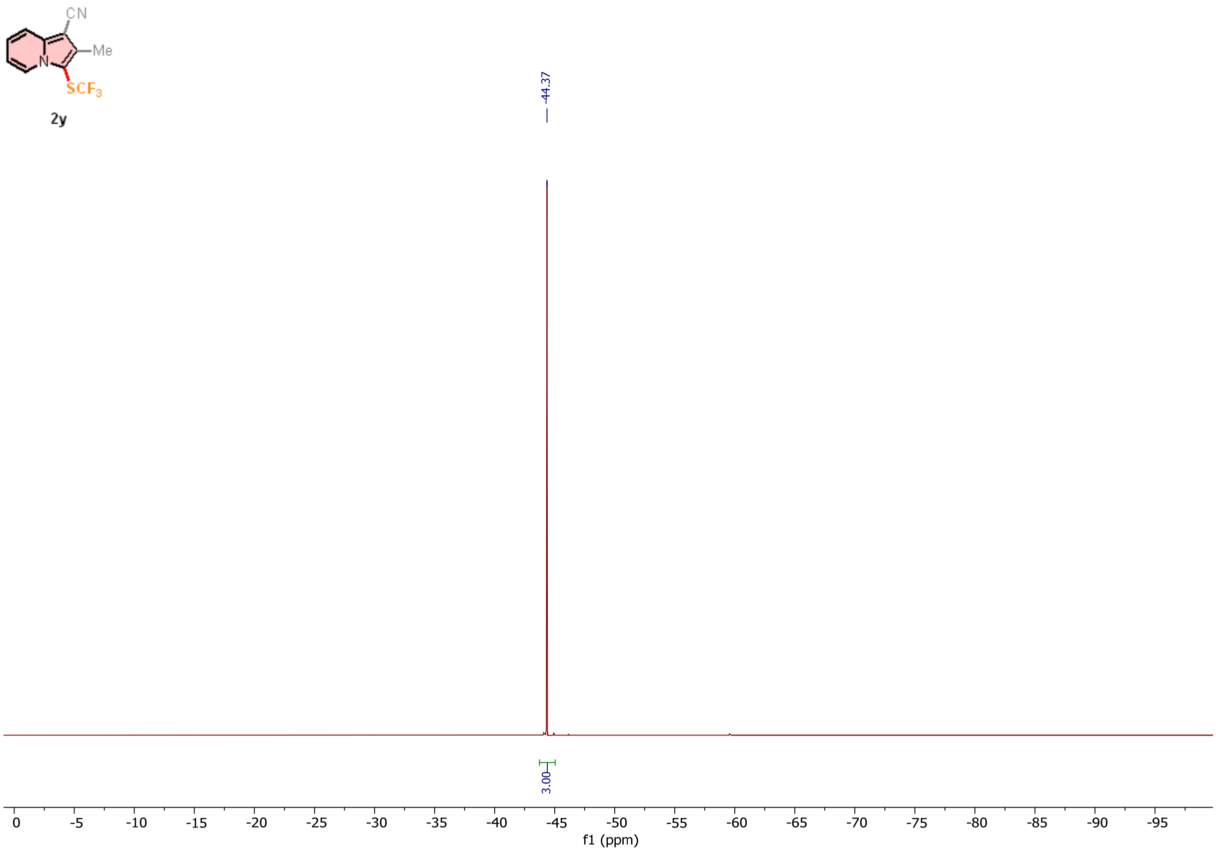

$^{19}F\{^{1}H\}$ NMR (377 MHz, $CDCl_3$) of **2y**.

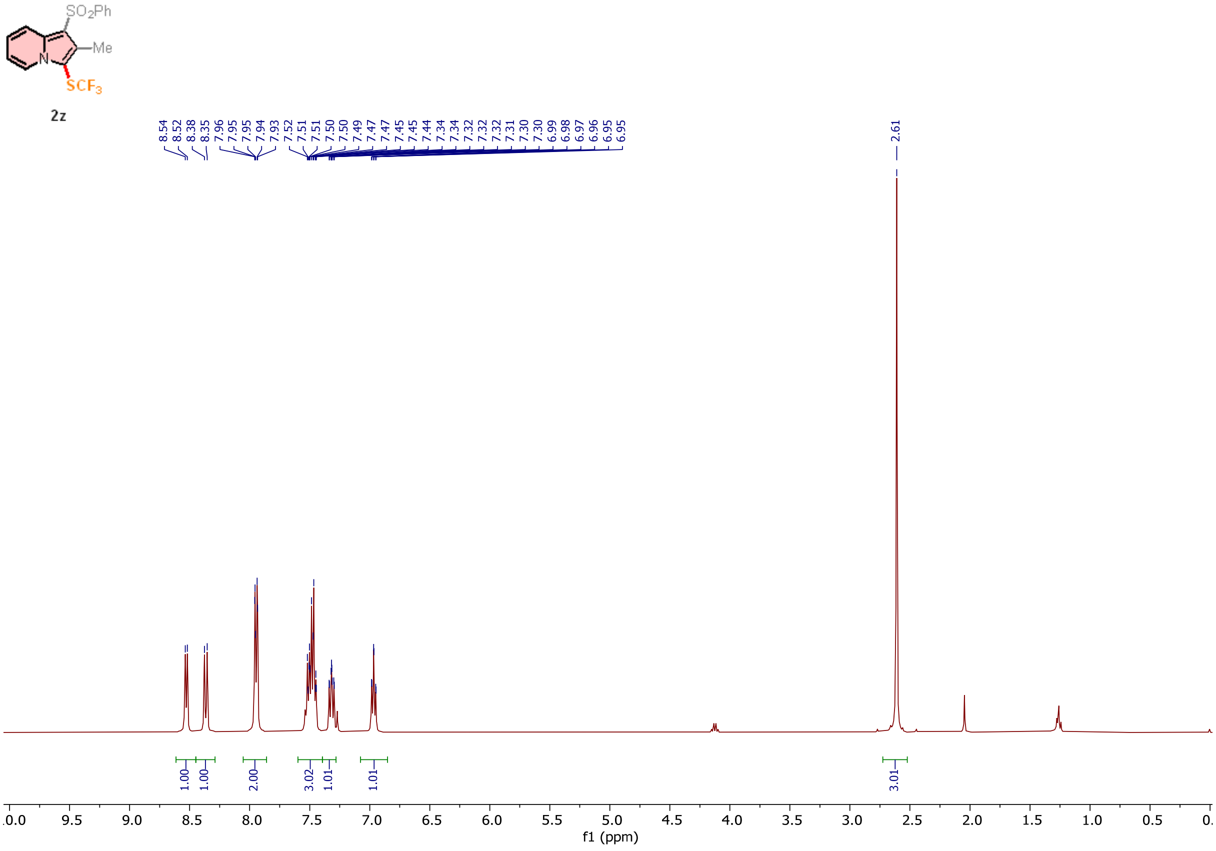

^{1}H NMR (400 MHz, $CDCl_3$) of **2z**.

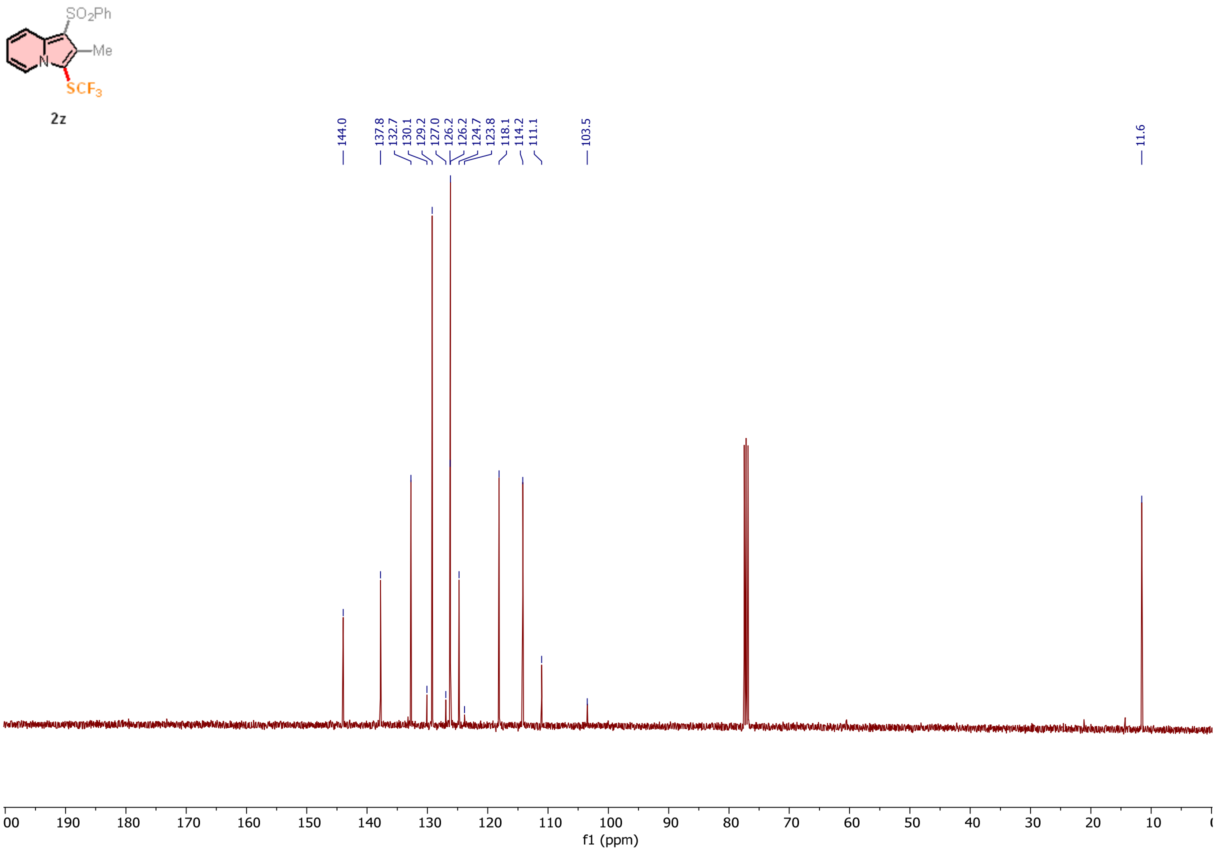

$^{13}C\{^1H\}$ NMR (100 MHz, $CDCl_3$) of **2z**

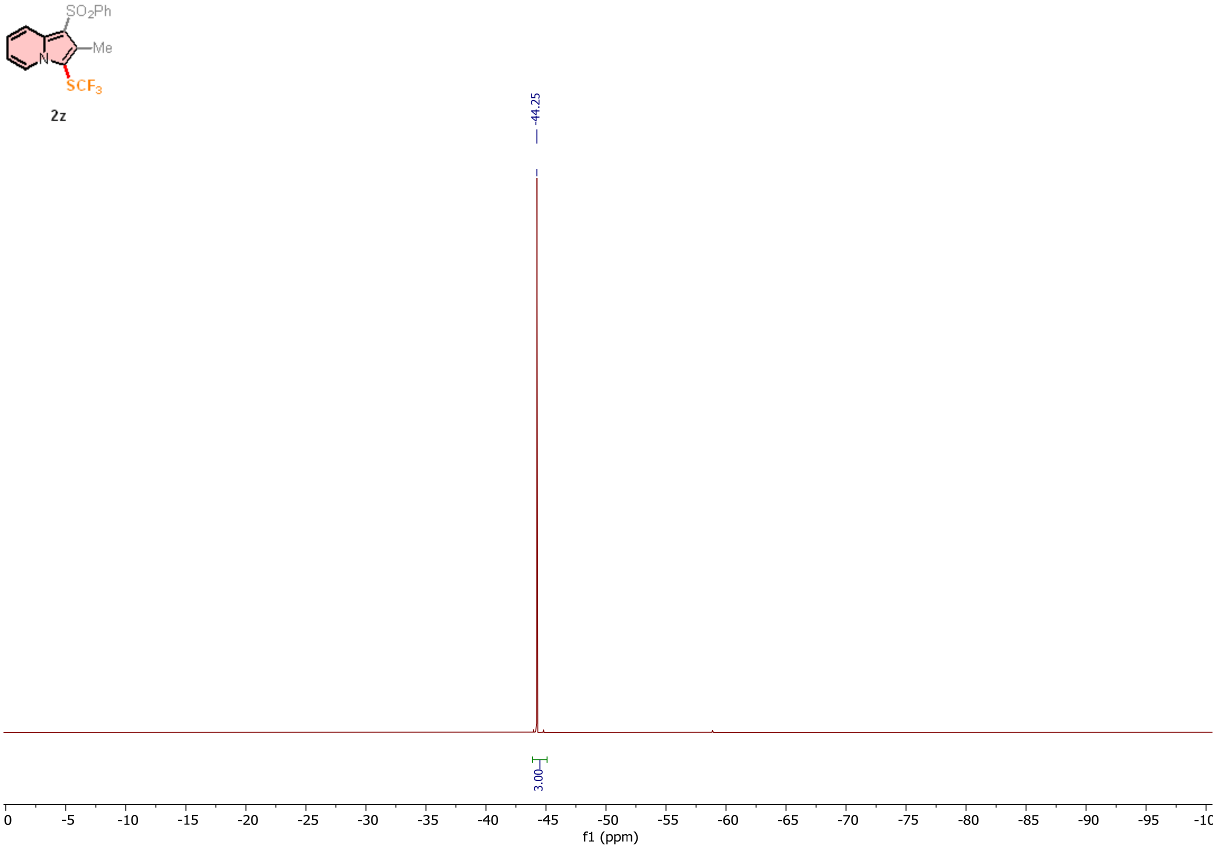

$^{19}F\{^1H\}$ NMR (377 MHz, $CDCl_3$) of **2z**.

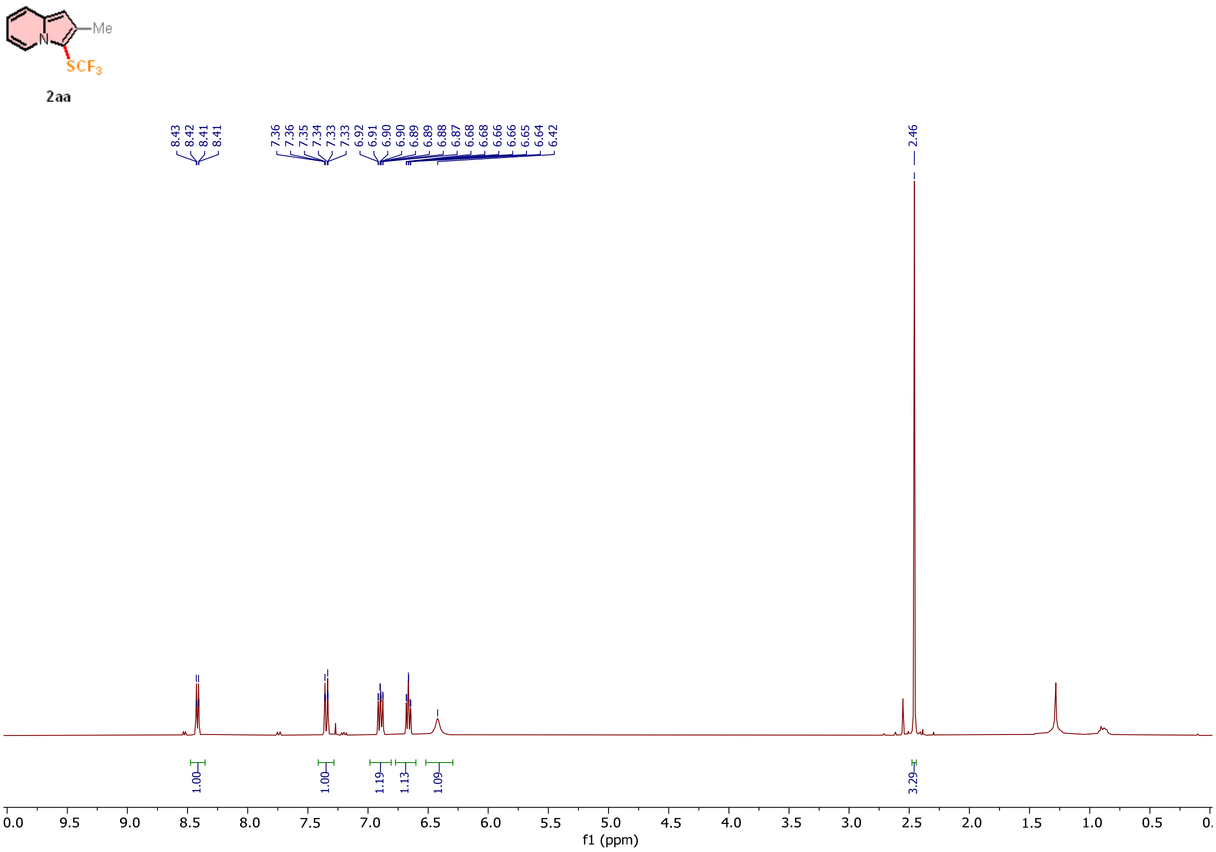

^{1}H NMR (400 MHz, $CDCl_3$) of **2aa**.

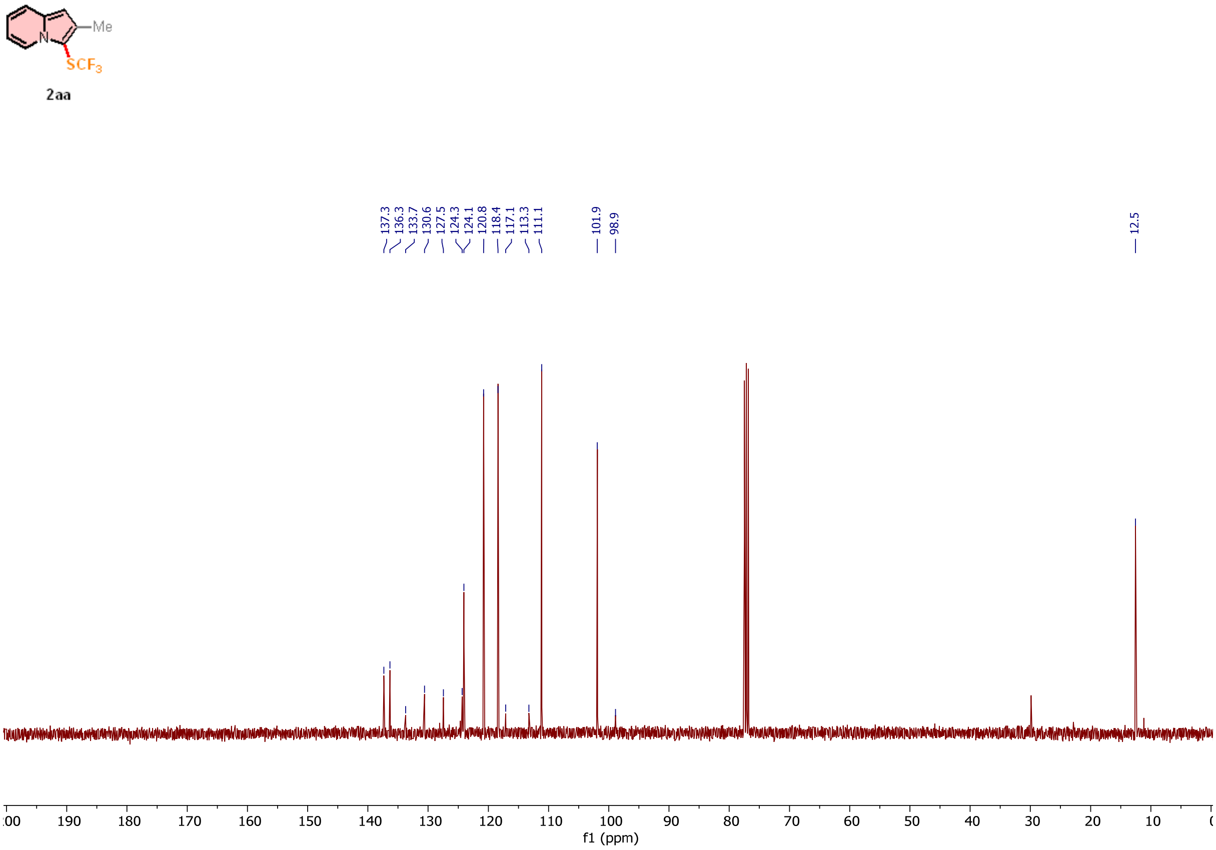

$^{13}C\{^{1}H\}$ NMR (100 MHz, $CDCl_3$) of **2aa**.

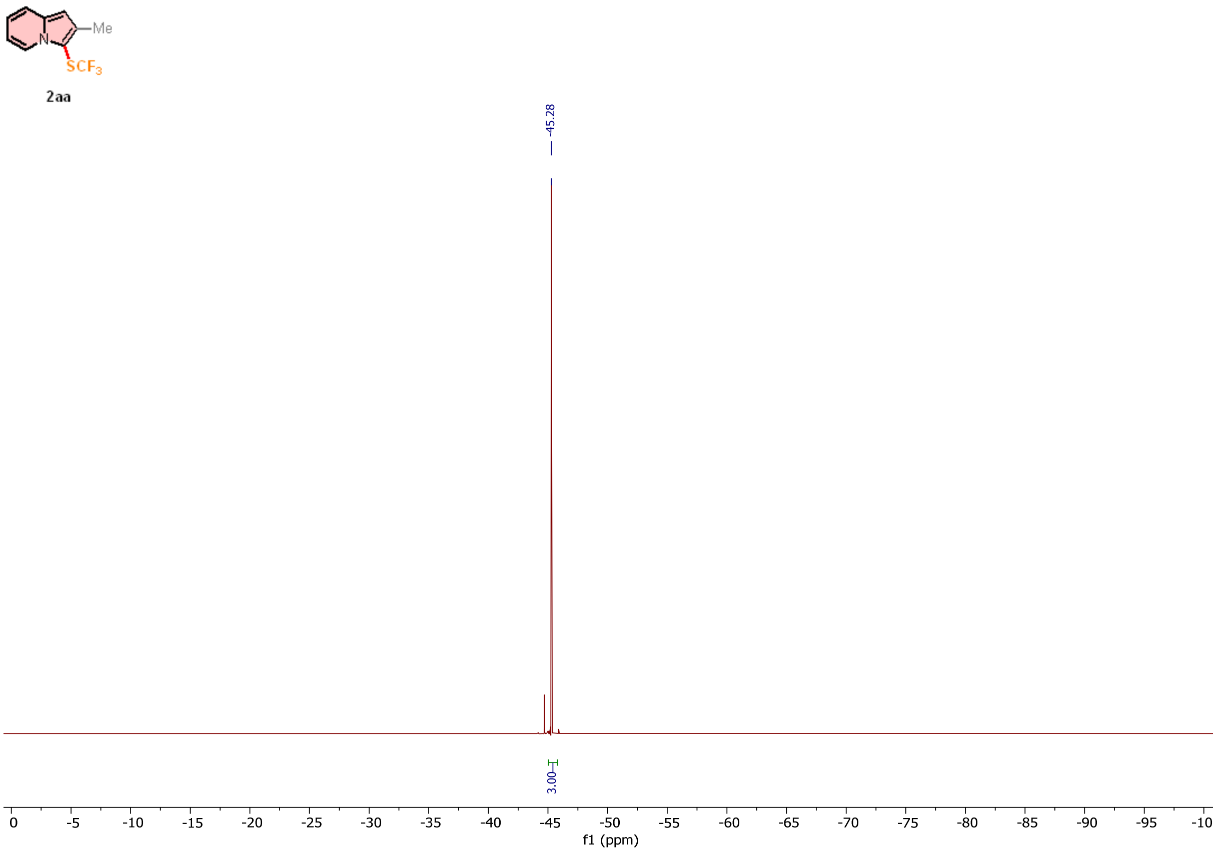

$^{19}F\{^{1}H\}$ NMR (377 MHz, $CDCl_3$) of **2aa**.

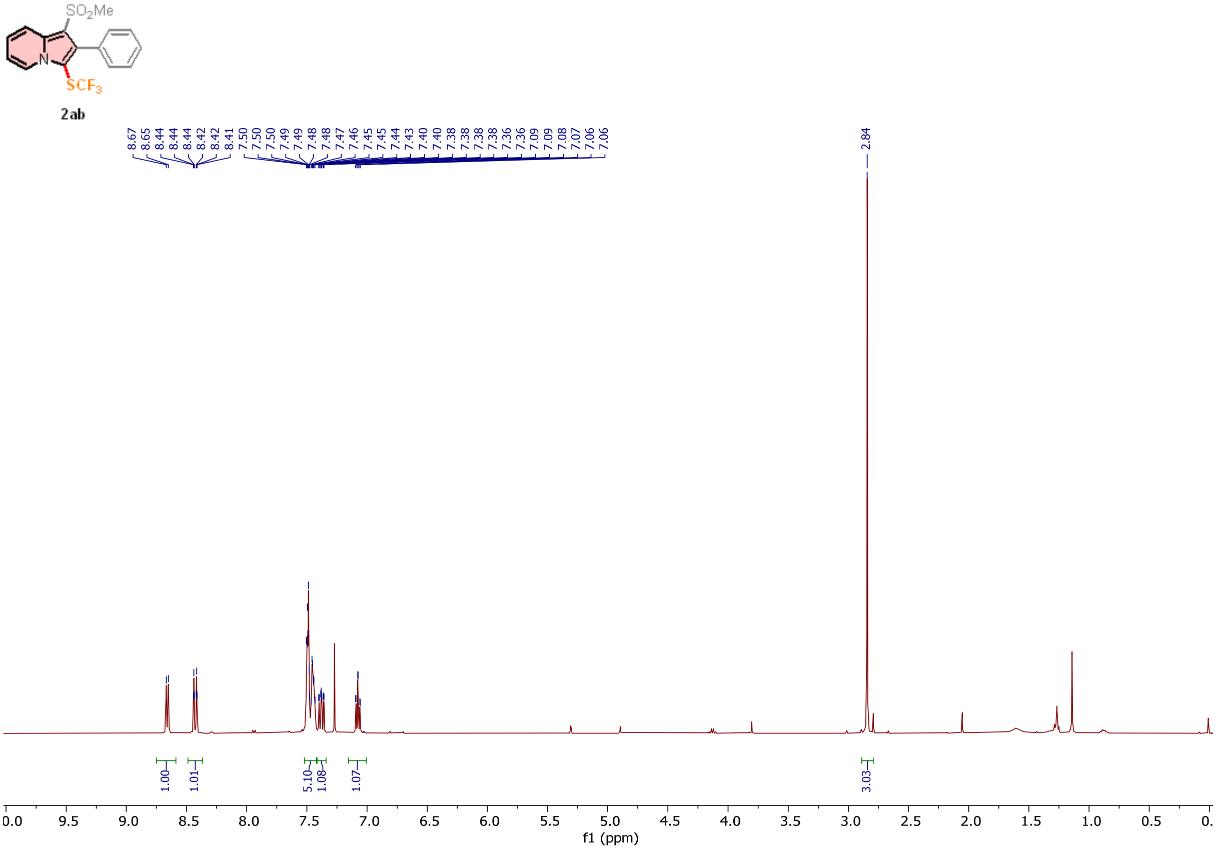

^{1}H NMR (400 MHz, $CDCl_3$) of **2ab**.

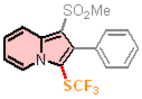

2ab

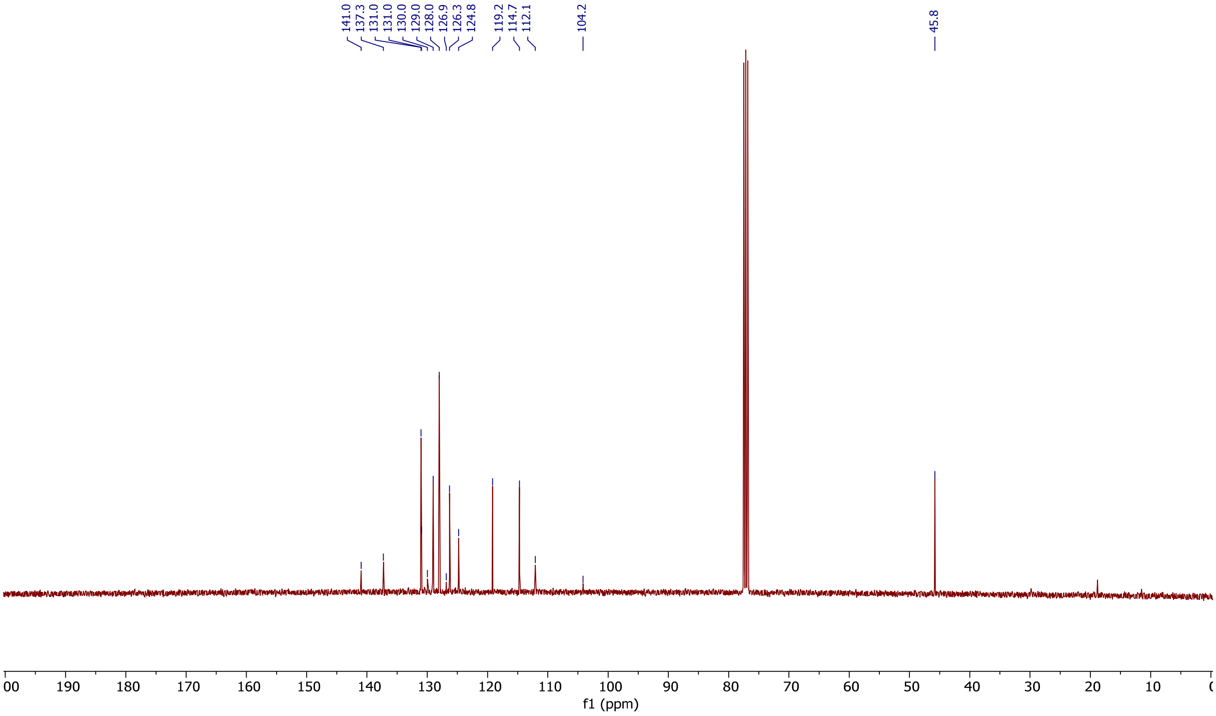

$^{13}C\{^1H\}$ NMR (100 MHz, $CDCl_3$) of **2ab**.

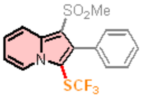

2ab

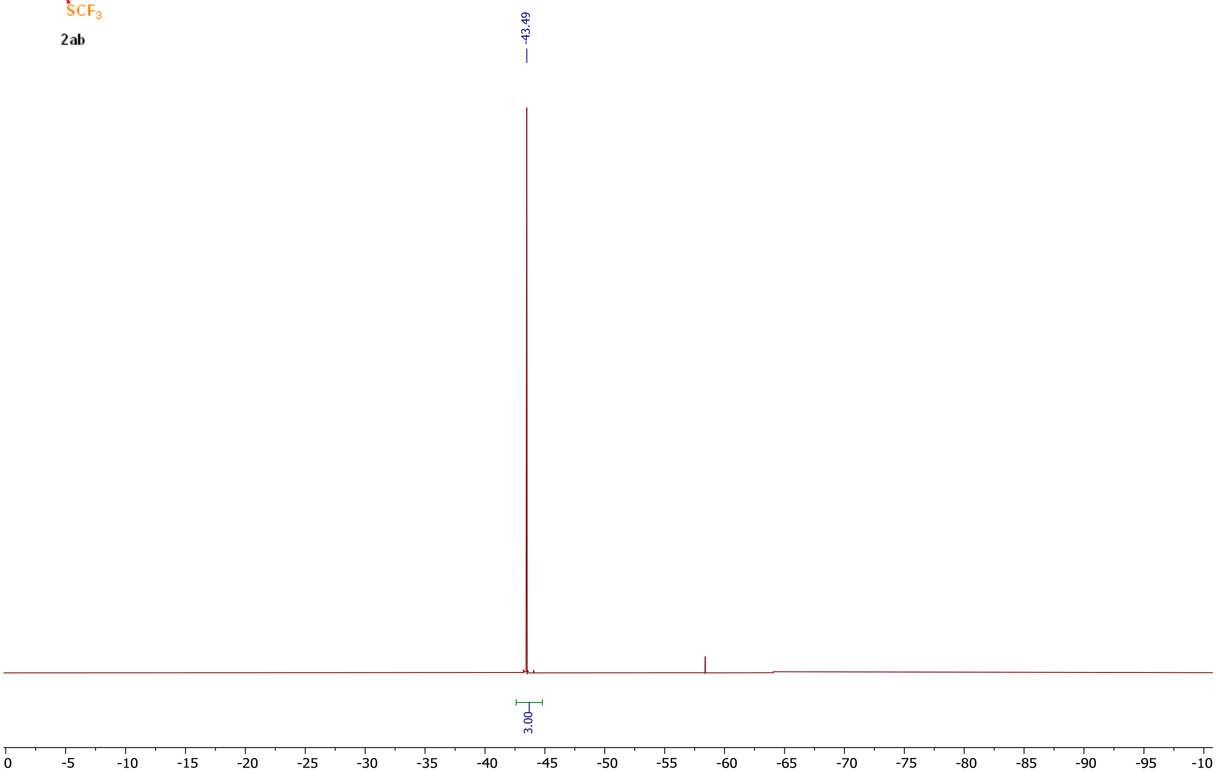

$^{19}F\{^1H\}$ NMR (377 MHz, $CDCl_3$) of **2ab**.

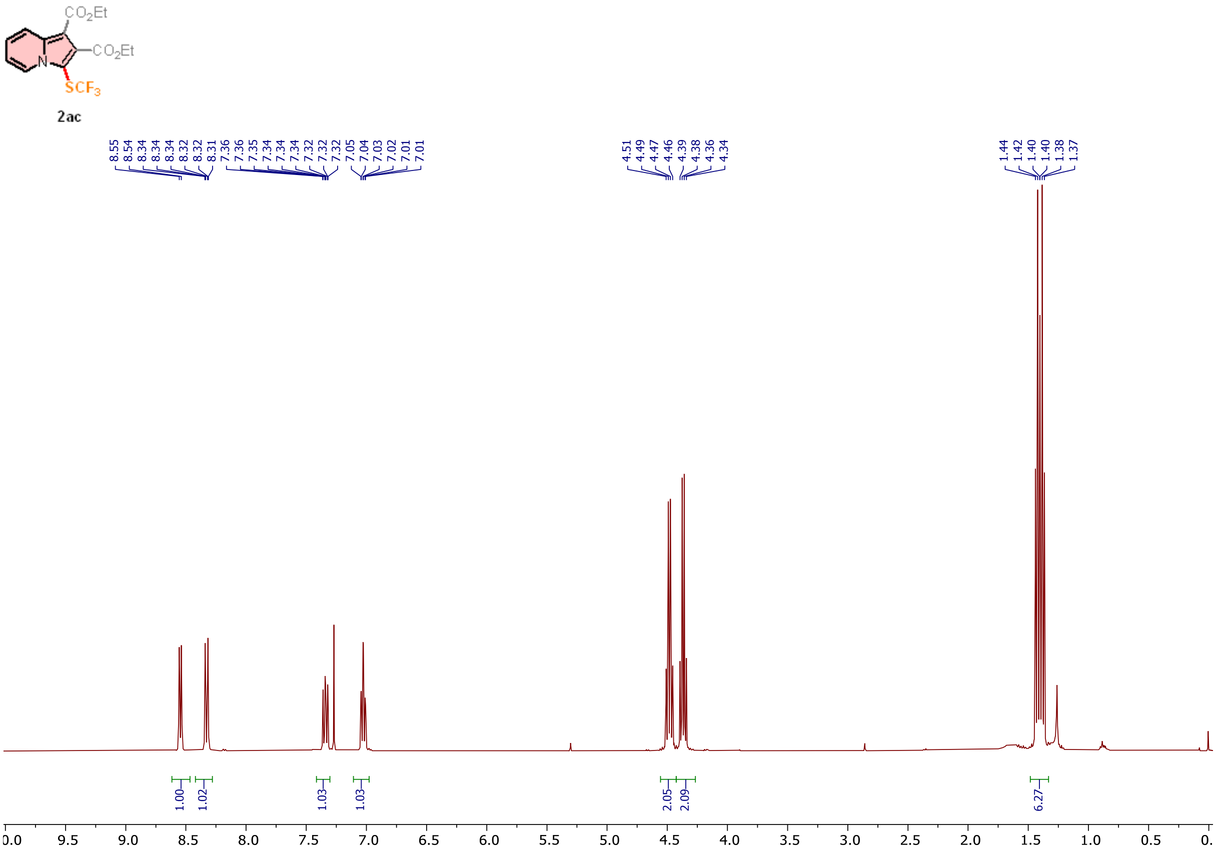

^{1}H NMR (400 MHz, $CDCl_3$) of **2ac**.

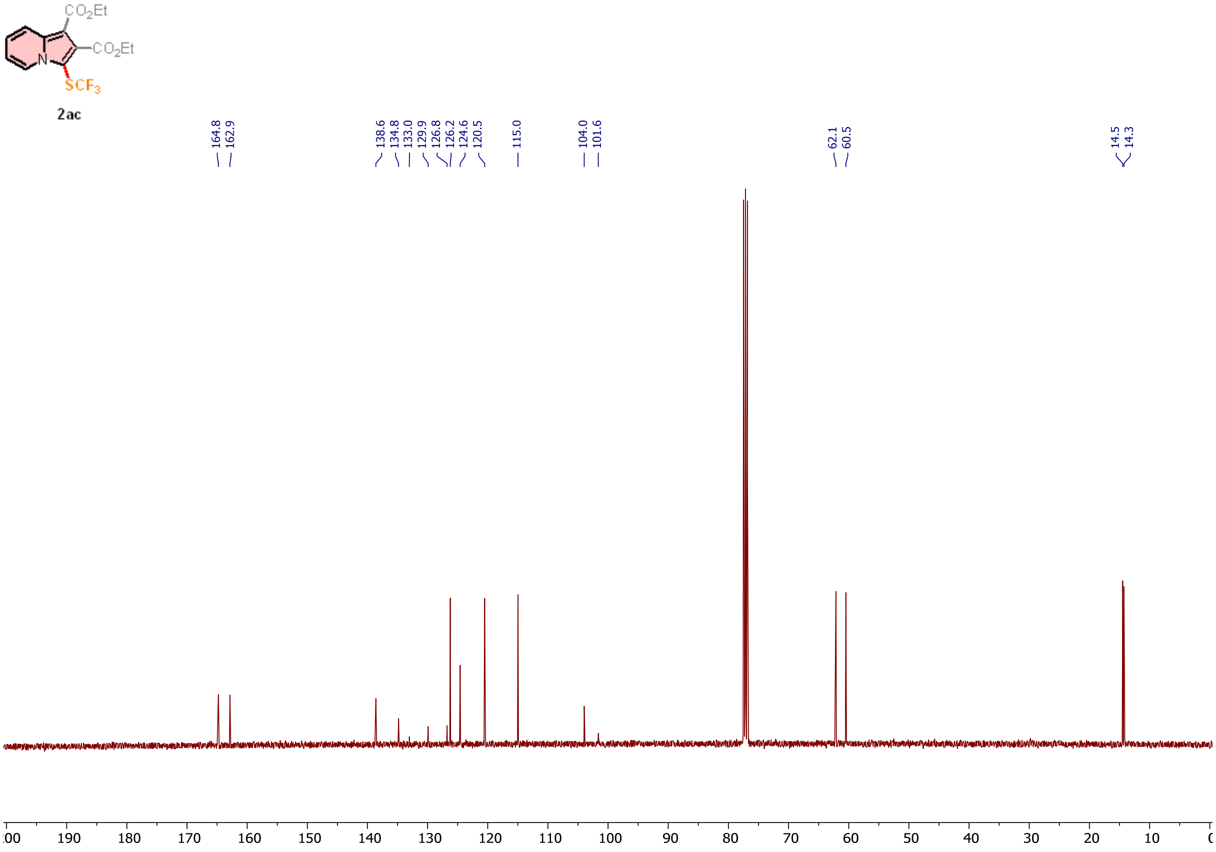

$^{13}C\{^{1}H\}$ NMR (100 MHz, $CDCl_3$) of **2ac**.

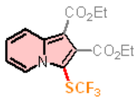

2ac

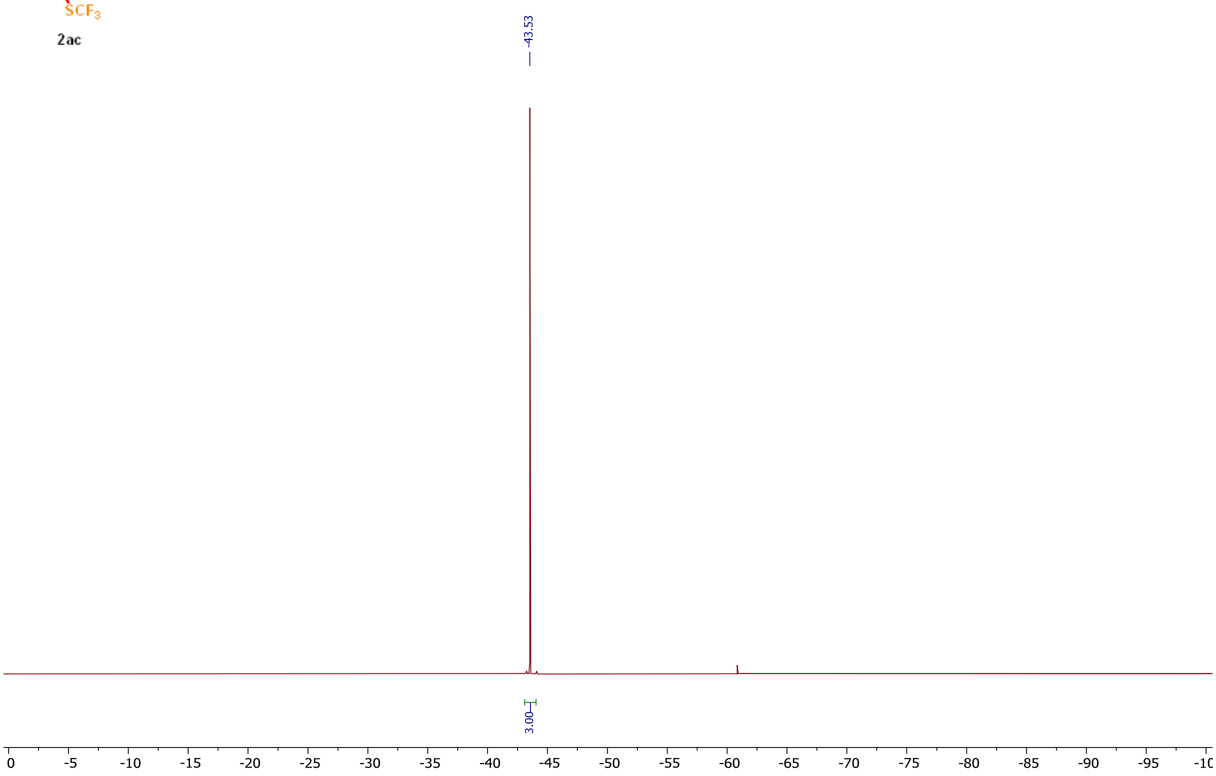

$^{19}F\{^{1}H\}$ NMR (377 MHz, $CDCl_3$) of **2ac**.

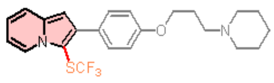

3

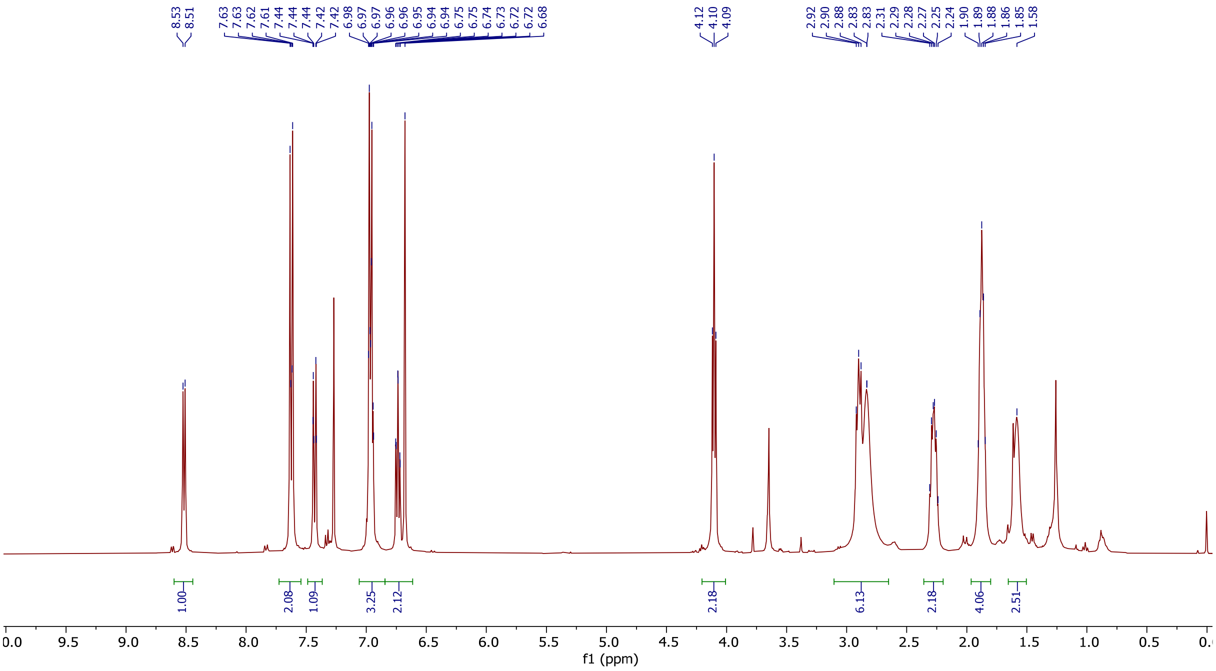

^{1}H NMR (400 MHz, $CDCl_3$) of **3**.

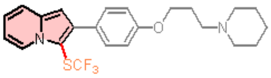

3

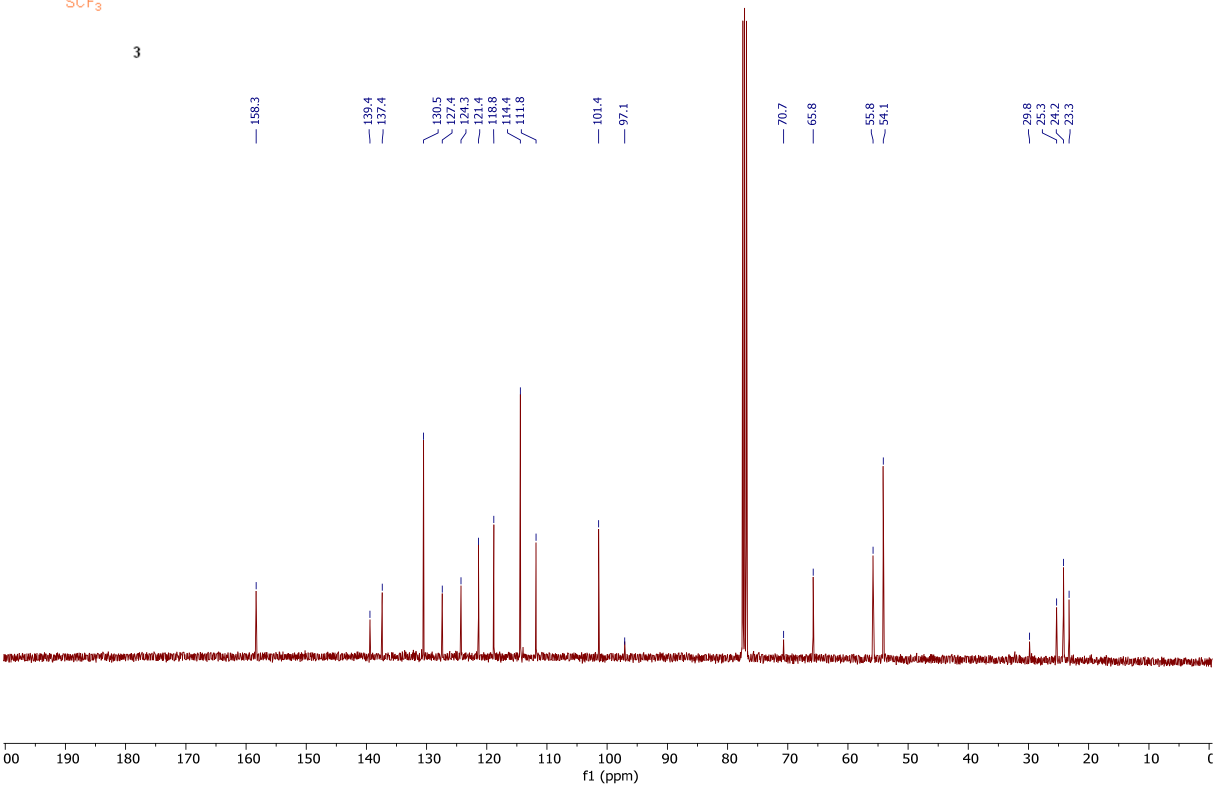

$^{13}C\{^{1}H\}$ NMR (100 MHz, $CDCl_3$) of **3**.

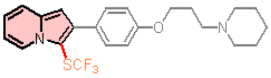

3

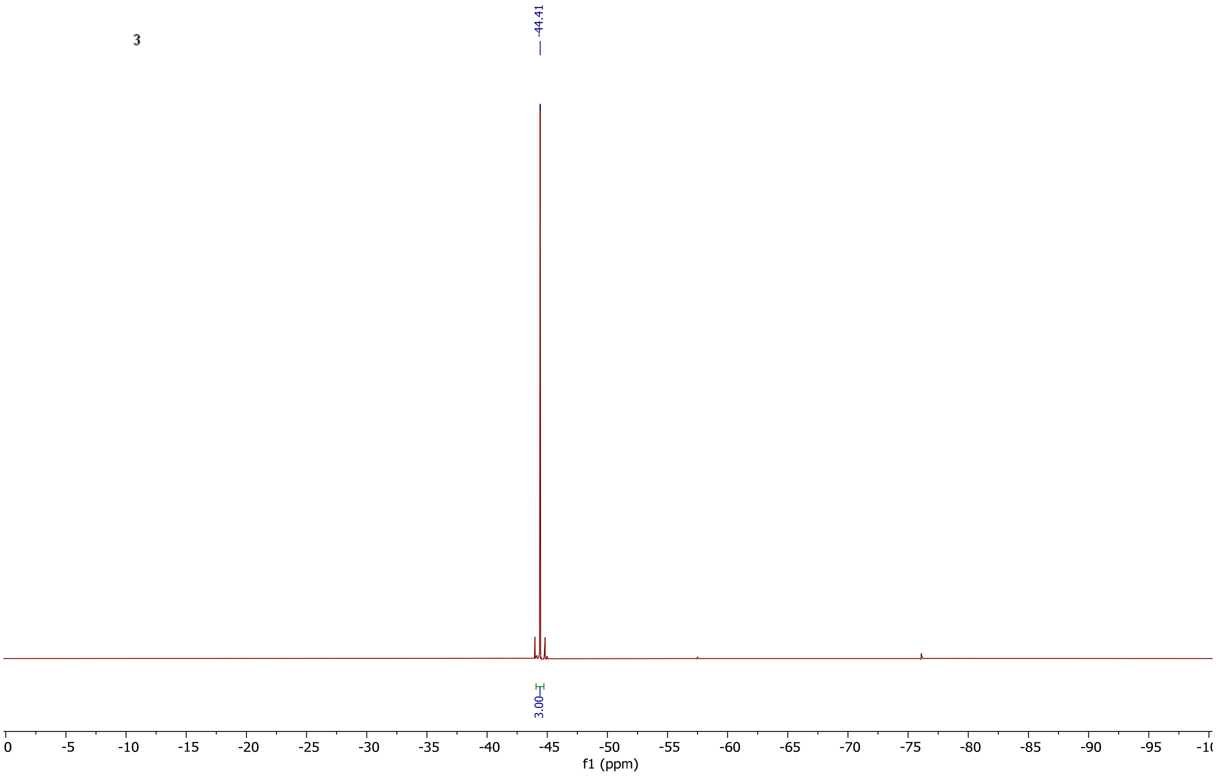

$^{19}F\{^{1}H\}$ NMR (377 MHz, $CDCl_3$) of **3**.

10. References

(1) Bragg, D. R.; Wibberley, D. G. 609. Indolizines. Part II. Preparation from Ethyl 2-Pyridylacetate and Related Compounds. *J. Chem. Soc. Resumed* **1963**, 3277. https://doi.org/10.1039/jr9630003277.

(2) Li, B.; Chen, Z.; Cao, H.; Zhao, H. Transition-Metal-Free Regioselective Cross-Coupling: Controlled Synthesis of Mono- or Dithiolation Indolizines. *Org. Lett.* **2018**, *20* (11), 3291–3295. https://doi.org/10.1021/acs.orglett.8b01168.

(3) Kim, W.; Kim, H. Y.; Oh, K. Oxidation Potential-Guided Electrochemical Radical–Radical Cross-Coupling Approaches to 3-Sulfonylated Imidazopyridines and Indolizines. *J. Org. Chem.* **2021**, *86* (22), 15973–15991. https://doi.org/10.1021/acs.joc.1c00873.

(4) Singh, D. K.; Kim, S.; Lee, J. H.; Lee, N. K.; Kim, J.; Lee, J.; Kim, I. 6-(Hetero)Arylindolizino[1,2-]Quinolines as Highly Fluorescent Chemical Space: Synthesis and Photophysical Properties. *J. Heterocycl. Chem.* **2020**, *57* (8), 3018–3028. https://doi.org/10.1002/jhet.4000.

(5) Bragg, D. R.; Wibberley, D. G. 609. Indolizines. Part II. Preparation from Ethyl 2-Pyridylacetate and Related Compounds. *J. Chem. Soc. Resumed* **1963**, No. 0, 3277–3281. https://doi.org/10.1039/JR9630003277.

(6) Baron, A.; Herrero, C.; Quaranta, A.; Charlot, M.-F.; Leibl, W.; Vauzeilles, B.; Aukauloo, A. Efficient Electron Transfer through a Triazole Link in Ruthenium(Ii) Polypyridine Type Complexes. *Chem. Commun.* **2011**, *47* (39), 11011. https://doi.org/10.1039/c1cc13683f.

(7) Norris, M. R.; Concepcion, J. J.; Glasson, C. R. K.; Fang, Z.; Lapides, A. M.; Ashford, D. L.; Templeton, J. L.; Meyer, T. J. Synthesis of Phosphonic Acid Derivatized Bipyridine Ligands and Their Ruthenium Complexes. *Inorg. Chem.* **2013**, *52* (21), 12492–12501. https://doi.org/10.1021/ic4014976.

(8) Xing, S.; Zhu, Y.-Y.; Liu, W.; Liu, Y.; Zhang, J.; Zhang, H.; Wang, Y.; Ni, S.-F.; Shao, X. C–H Fluoroalkylsulfinylation/Intramolecular Rearrangement for Precise Synthesis of Fluoroalkyl Sulfoxides. *Org. Lett.* **2022**, *24* (18), 3378–3383. https://doi.org/10.1021/acs.orglett.2c01151.

(9) Xu, C.; Ma, B.; Shen, Q. N-Trifluoromethylthiosaccharin: An Easily Accessible, Shelf-Stable, Broadly Applicable Trifluoromethylthiolating Reagent. *Angew. Chem. Int. Ed.* **2014**, *53* (35), 9316–9320. https://doi.org/10.1002/anie.201403983.

(10) Stefanoni, K. K.; Schmitz, M.; Treuheit, J.; Kerzig, C.; Wilhelm, R. Bichromophoric Ruthenium Complexes for Photocatalyzed Late-Stage Synthesis of Trifluoromethylated Indolizines. *J. Org. Chem.* **2025**, *90* (19), 6491–6503. https://doi.org/10.1021/acs.joc.5c00319.

(11) Hansch, Corwin.; Leo, A.; Taft, R. W. A Survey of Hammett Substituent Constants and Resonance and Field Parameters. *Chem. Rev.* **1991**, *91* (2), 165–195. https://doi.org/10.1021/cr00002a004.

(12) He, X.; Chen, Z.; Zhu, X.; Liu, H.; Chen, Y.; Sun, Z.; Chu, W. Photoredox-Catalyzed Trifluoromethylation of 2H-Indazoles Using TT-CF3+OTf− in Ionic Liquids. *Org. Biomol. Chem.* **2023**, *21* (8), 1814–1820. https://doi.org/10.1039/D3OB00096F.

(13) Jia, H.; Häring, A. P.; Berger, F.; Zhang, L.; Ritter, T. Trifluoromethyl Thianthrenium Triflate: A Readily Available Trifluoromethylating Reagent with Formal CF3+, CF3•, and CF3– Reactivity. *J. Am. Chem. Soc.* **2021**, *143* (20), 7623–7628. https://doi.org/10.1021/jacs.1c02606.

(14) Malpani, Y. R.; Biswas, B. K.; Han, H. S.; Jung, Y.-S.; Han, S. B. Multicomponent Oxidative Trifluoromethylation of Alkynes with Photoredox Catalysis: Synthesis of α-

Trifluoromethyl Ketones. *Org. Lett.* **2018**, *20* (7), 1693–1697. https://doi.org/10.1021/acs.orglett.8b00410.
