# Supplementary figures and images for: Access to SCF3‑Substituted Indolizines via a Photocatalytic Late-Stage Functionalization Protocol
Source: Org Lett. 2025 Jul 21;27(31):8389–93. doi: 10.1021/acs.orglett.5c02079 (PMC12340967; doi:10.1021/acs.orglett.5c02079)

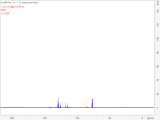

Supplement: Supplementary file 2 [file ol5c02079_si_002.zip › FID for Publication/2a/13C/pdata/1/thumb.png]

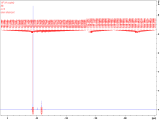

Supplement: Supplementary file 2 [file ol5c02079_si_002.zip › FID for Publication/2a/19F/pdata/1/thumb.png]

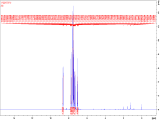

Supplement: Supplementary file 2 [file ol5c02079_si_002.zip › FID for Publication/2a/1H/pdata/1/thumb.png]

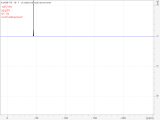

Supplement: Supplementary file 2 [file ol5c02079_si_002.zip › FID for Publication/2aa/19F/pdata/1/thumb.png]
